# Uridine Bisphosphonate-Carbagalactose: A Stabilized α‑1,4-Galactosyltransferase Inhibitor

**DOI:** 10.1021/acs.orglett.6c03808

**Published:** 2026-10-02

**Authors:** Jianyun Guo, Nicky de Koster, Bob van Puffelen, Roy Steneker, Vincent Verhoeks, Tim P. Ofman, Herman S. Overkleeft, Marta Artola, Dmitri V. Filippov, Jeroen D. C. Codée

**Affiliations:** Leiden Institute of Chemistry, 4496 Leiden University, Einsteinweg 55, 2333 CC Leiden, Netherlands

## Abstract

Methylene and difluoromethylene uridine bisphosphonate carbagalactose were designed and synthesized as stable uridine diphosphate-galactose (UDP-Gal) mimics for probing α-1,4-galactosyltransferase (A4GALT). In these analogues, the labile glycosidic and pyrophosphate linkages of UDP-Gal are replaced by chemically and enzymatically stable motifs, yielding compounds that retain key structural features essential for biological recognition. This work demonstrates the first successful nucleophilic substitution of a secondary triflate with a difluoromethylene bisphosphonate, providing a methodology that should enable efficient access to a range of sugar nucleotide mimics. The CH_2_ and CF_2_ UDP-Gal analogues **5** and **6** inhibited A4GALT, displaying IC_50_ values of 249 and 217 μM, respectively, in the presence of 500 μM UDP-Gal, consistent with efficient recognition by the A4GALT donor-binding site.

## Graphical abstract

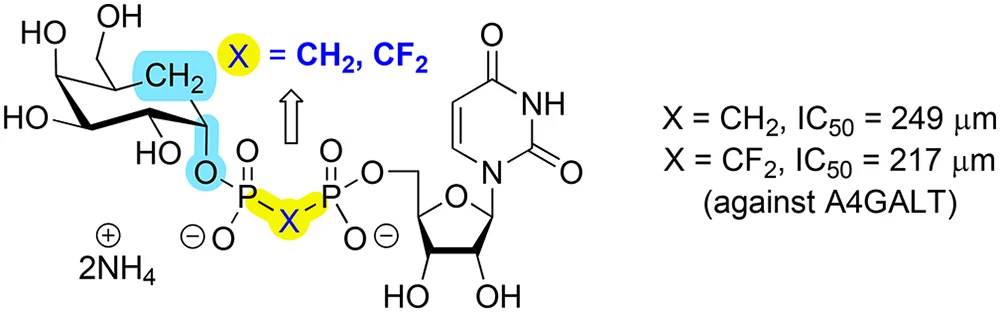

Glycosyltransferases (GTs) are a large family of enzymes responsible for the construction of glycosidic linkages through the transfer of a sugar moiety from an activated donor to an acceptor.^1^ These enzymes play an important role in post-translational modifications, shaping cell signaling, immune responses, and other essential biological processes.^2^ GT inhibitors can be valuable tools to understand and elucidate the mode of action of these enzymes and lead to the discovery of potential therapeutic agents.^3^ However, due to the lack of available three-dimensional structures for many GTs and their complex reaction mechanisms, which can involve significant conformational changes, the rational development of inhibitors remains a considerable challenge.^4^

α-1,4-Galactosyltransferase (A4GALT) is a member of the GT32 family (www.cazy.org) and is a retaining glycosyltransferase that transfers a galactose from UDP-galactose (UDP-Gal) to lactosylceramide (LacCer), forming an α-1,4-glycosidic linkage during the globotriaosylceramide (Gb3) biosynthesis. Fabry disease is a lysosomal storage disorder caused by impaired α-galactosidase A (GLA) activity, which leads to the accumulation of Gb3 in cells.^5^ Current therapies involve enzyme replacement therapy and the use of pharmacological chaperones, but their efficacy can be limited, and many patients continue to experience disease progression.^6^ An alternative strategy would be the inhibition of A4GALT to lower Gb3 biosynthesis.^7^ This makes A4GALT an attractive therapeutic target, and the conception of UDP-Gal mimics can provide useful chemical tools to better understand its catalytic mechanism.

A classical strategy for the development of GT inhibitors involves modifying the sugar-nucleotide donor. Donor mimics can bind to the enzyme active site and compete with the natural donor to achieve inhibition. The glycosidic bond and pyrophosphate linkage of UDP-galactose (**1**, Figure 1A) are both susceptible to enzymatic hydrolysis, and several studies have focused on mimicking these two regions to develop stable UDP-Gal analogues. Structural changes in the sugar moiety, such as replacing the endocyclic oxygen with a methylene group to give carbasugar **2**,^8^ or the introduction of electron-withdrawing groups, enhance the stability of the glycosidic linkage.^9^ Likewise, the substitution of the pyrophosphate with nonhydrolyzable isosteres such as methylene bisphosphonate **3** ^10^ or malonate **4** ^11^ has been reported for the inhibition of glycosyl transferases (Figure 1B). Building on our previous work on methylene bisphosphonate^12^ (P–CH_2_–P) and difluoromethylene bisphosphonate^13^ (P–CF_2_–P) mimics, we reasoned that a new class of methylene and difluoromethylene bisphosphonate UDP-carbagalactose could be within reach as nonhydrolyzable donor-type mimics (analogues **5** and **6**, Figure 1C). In particular, difluoromethylene bisphosphonate (P–CF_2_–P) analogues stand out since they closely mimic the natural pyrophosphates in terms of p*K*_a_ value, polarity, and size.^14^ This makes them promising candidates as stable donor-type inhibitors. Herein, we report the synthesis of (difluoro) methylene bisphosphonate UDP-carbagalactose analogues as stable donor-type inhibitors and their evaluation as recombinant human A4GALT inhibitors.

**Figure 1:**
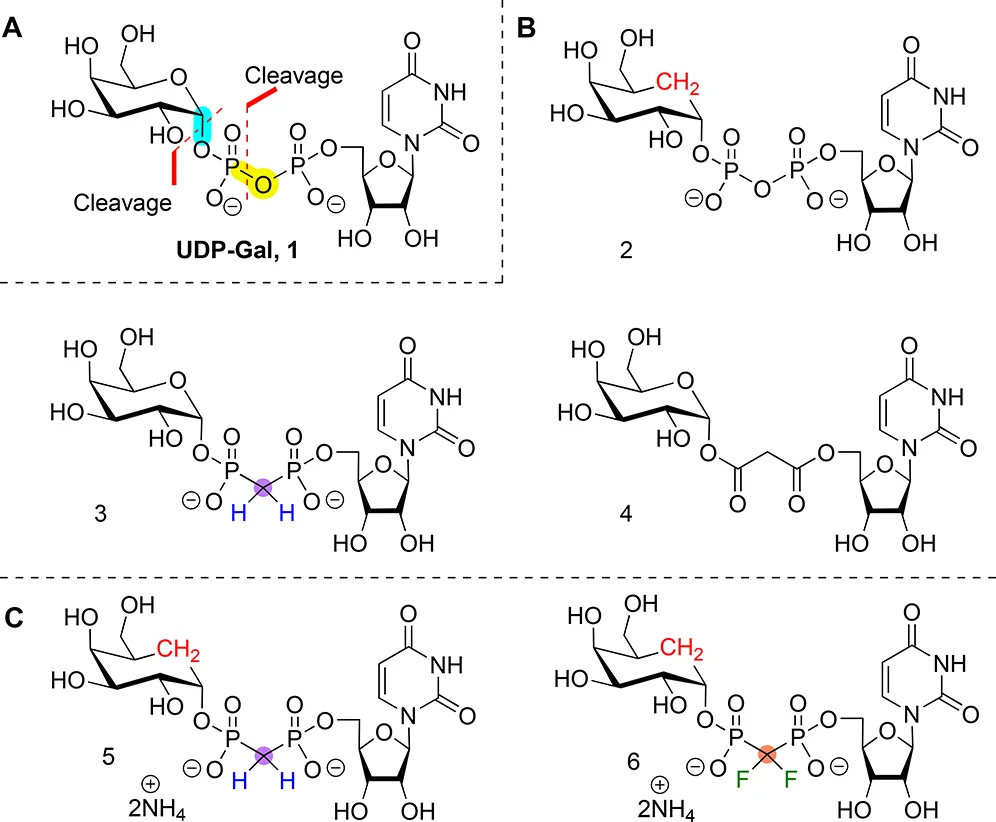
Natural UDP-Gal donor and structures of synthetic UDP-Gal inhibitors. (A) Structure of natural donor UDP-Gal **1**. (B) Carba UDP-Gal **2**, methylene phosphonate analogue **3** and malonate analogue **4**. (C) Methylene and difluoromethylene bisphosphonate analogues **5** and **6** developed in this work.

Our synthesis began with the preparation of α-carbagalactose (Scheme 1). Cyclohexene derivative **7** was synthesized in nine steps from d-xylose, essentially as described by Llebaria and co-workers (see SI for details).^15^ Unlike the original route, we employed acid-labile para-methoxybenzyl (PMB) protecting groups on the carbasugar building blocks, which allows for a straightforward global deprotection in a single acidic step at the final stage of the synthesis. Epoxidation of compound **7** using *m*-CPBA produced a separable mixture of epoxides **8** and **9**, with the β-epoxide **9** being the major product (61%). The selective opening of epoxide **8** and **9** using various reducing agents was investigated. However, all tested hydride reagents, including LiAlH_4_, LiHBEt_3_, L-selectride, and LiBH_4_, failed to achieve selective ring opening of α-epoxide **8**, with the starting material mainly being recovered and only minor decomposition and ring-opened products being formed (see SI for details). The low reactivity of the α-epoxide is likely due to steric hindrance of the PMB ethers at C3, C4, and C6 on the backside of the α-epoxide, preventing nucleophilic attack by the hydride reagents. We therefore turned our attention to β-epoxide **9**. The use of common hydride reagents resulted in poor selectivity (see SI), but treatment of **9** with the bulky L-selectride effected smooth ring opening to furnish compound **10** in 71% yield with only a small amount of compound **11** formed (**10**:**11** = 7:1). The regioselectivity in this reaction is remarkable as nucleophilic attack at C1 would lead to a favorable half-chair transition state (the Fürst–Plattner rule).^16^ Attack at the C7 position of β-galactose-configured cyclophellitol with L-selectride however proceeds through a twist-boat like transition state. The observed outcome may be due to steric effects, as the bulky L-selectride may encounter more severe interactions when approaching the C1 position in comparison to the C7 position. Finally, the α-anomer of carbagalactose **12** was obtained from the β-anomer **10** through a Mitsunobu reaction with nitrobenzoic acid and subsequent methanolysis in 76% yield over two steps.

**Scheme 1:**
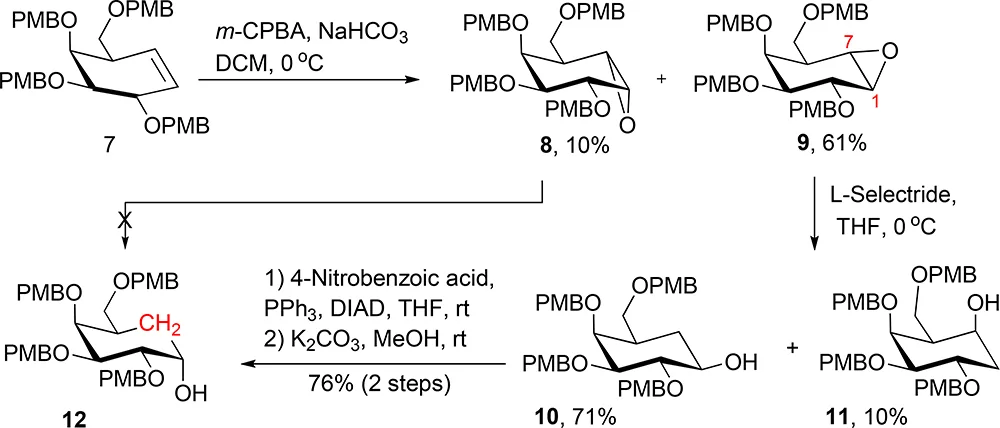
Synthesis of α-Carbagalactose **12**

With α-carbagalactose **12** in hand, we first set out to synthesize the P–CH_2_–P linked analogue **5** (Scheme 2). As a nonhydrolyzable pyrophosphate mimic, the P–CH_2_–P moiety introduces minimal structural changes while resisting enzymatic cleavage and it has been widely used in the design of inhibitors.^17^ To incorporate this mimic into our UDP-Gal mimetic designs, mixed phosphonamidite–phosphonodiester reagent **16** was generated in two steps from methylphosphonate **13** and chlorophosphorodiamidite **14** following a procedure previously developed by our group.^12a^ Thus, compound **13** was first treated with 1.0 equiv of LDA, and then reacted with compound **14** to afford intermediate **15** in 72% yield. Activation of compound **15** with 4,5-dicyanoimidazole (DCI, 0.6 equiv), followed by reaction with *tert*-butanol, afforded reagent **16** in 76% yield. Condensation of *a*-carbagalactose **12** with phosphoramidite **16** was accomplished using DCI as an activator. Subsequent in situ oxidation of the phosphonite-phosphonate intermediate with *t*BuOOH yielded methylene bisphosphonate derivative **17** in 62% yield over two steps. Next, selective removal of one of the methyl groups was achieved using thiophenol in a mixture of MeCN/Et_3_N, which was followed by a condensation reaction with partially protected uridine **18** using Wada’s reagent (PyNTP), which was selected based on our previous studies^12,13^ that demonstrated its efficiency in the coupling of bisphosphonates with primary alcohols. This afforded compound **19** in 44% yield over two steps. Global deprotection of **19** was achieved by removal of the methyl ester using thiophenol in MeCN/Et_3_N, and cleavage of all the *tert*-butyl, PMB and Boc groups in a single step using TFA/TES, providing methylene UDP-carbagalactose **5** in 41% yield over two steps after size-exclusion chromatography.

**Scheme 2:**
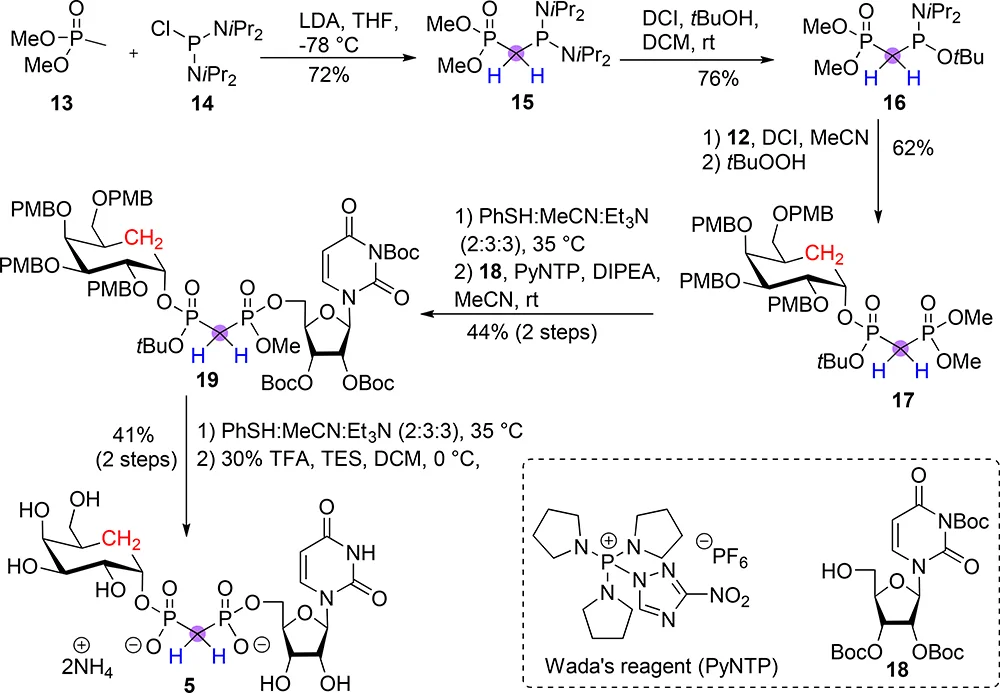
Synthesis of P-CH_2_–P UDP-Carbagalactose **5**

After successfully obtaining P–CH_2_–P analogue **5**, our attention was turned to the synthesis of the P–CF_2_–P-linked analogue **6** (Scheme 3). Like the P–CH_2_–P analogues, P–CF_2_–P pyrophosphate analogues are hydrolytically stable and have a polarity similar to the natural pyrophosphate.^14b,18^ Following our previously reported stepwise demethylation-condensation strategy^13^ using diethyl­(dimethyl)­difluoromethylene bisphosphonate reagent **20**, mono tetrabutylammonium salt **21** was generated and used in combination with Wada’s reagent in the condensation with partially protected uridine **18** to provide compound **22** in 65% yield over two steps. Next, the remaining methyl group in compound **22** was removed using tetrabutylammonium benzoate (1.0 equiv) to give compound **23**. Unfortunately, the subsequent coupling of compounds **23** and **12** using Wada’s reagent was unsuccessful, with no product formation observed even under reflux conditions or with 6 equiv of Wada’s reagent (Table 1, entries 1–3). This outcome may be attributed to the reduced reactivity of the secondary alcohol in **12** in comparison to the primary alcohols we previously successfully used.

**Scheme 3:**
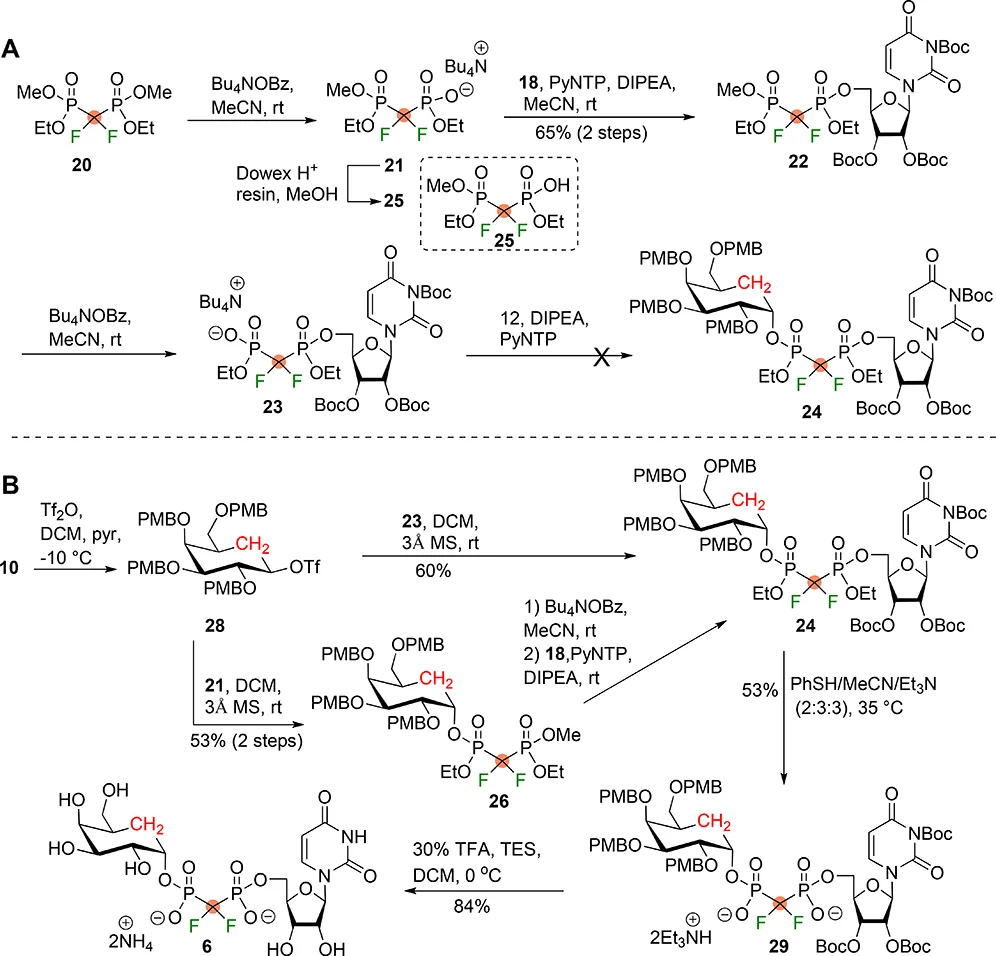
Synthesis of P-CF_2_–P UDP-Carbagalactose **6**

**Table 1:**
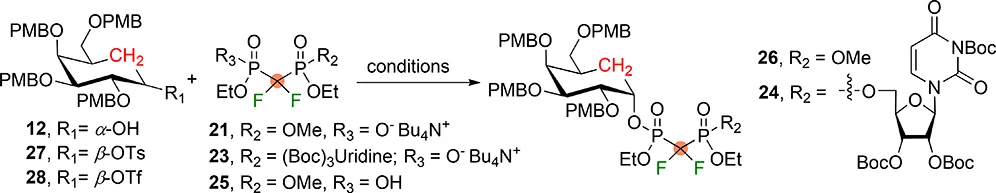
Optimization of the Coupling Reaction with Difluoromethylene Phosphonates

Entry	Carba-sugar (equiv)	Bisphosphonate (equiv)	Product	Conditions (equiv)	Solvent	Temp. (°C)	Yield (%)
1	**12** (1.0)	**23** (1.5)	**24**	PyNTP (3.0)	MeCN	rt	NR
2	**12** (1.0)	**23** (1.5)	**24**	PyNTP (6.0)	MeCN	rt	NR
3	**12** (1.0)	**23** (1.5)	**24**	PyNTP (3.0)	MeCN	reflux	NR
4	**10** (1.0)	**25** (2.0)	**26**	(*p*-ClPh)_3_P (2.0), DIAD (2.0), Et_3_N (5.0)	THF	rt	Trace
5	**27** (1.0)	**21** (2.0)	**26**	-	MeCN	rt	NR
6	**27** (1.0)	**21** (2.0)	**26**	-	MeCN	reflux	NR
7	**27** (1.0)	**21** (2.0)	**26**	-	DMF	rt	NR
8	**27** (1.0)	**21** (2.0)	**26**	-	DMF	100	NR
9	**28** (1.0)	**21** (1.0)	**26**		DCM	rt	51
10	**28** (1.0)	**21** (1.0)	**26**	3Å MS	DCM	rt	53
11	**28** (1.0)	**23** (1.0)	**24**	3Å MS	DCM	rt	60

A few other methods have been reported for attaching difluoromethylene bisphosphonates to hydroxyl groups, including the use of dehydrating agents such as dicyclohexylcarbodiimide (DCC) as well as the nucleophilic substitution of good leaving groups, such as tosylates or mesylates.^19^ However, all these methods have been applied exclusively to primary alcohols, and no direct coupling of a secondary alcohol with a difluoromethylene bisphosphonate reagent has been reported. Mitsunobu reactions have previously enabled the formation of phosphonates from phosphonic acid monoesters with both primary and secondary alcohols.^20^ Therefore, we explored Mitsunobu reaction conditions, following procedures reported for the coupling of secondary alcohols. Since compound **23** was obtained through a multistep synthesis, we used the more readily accessible acid **25**, prepared from **21** using a Dowex H^+^ ion-exchange resin, as a model nucleophile to screen reaction conditions. Unfortunately, after exploring a multitude of different conditions, in which we varied the solvent, temperature, reagent ratio, order of addition, azodicarboxylate reagent and P^III^ reagents (see SI), only trace amounts of product **26** were detected (entry 4). We therefore turned to a nucleophilic substitution strategy using tosylate **27**. This electrophile was subjected to a substitution with compound **21** under various conditions as summarized in entries 5–8, but no product was obtained.

We then probed the more reactive triflate **28** as an alternative. The synthesis of this triflate required significant optimization because of its hydrolytic lability. We eventually found that it could be effectively generated by treating alcohol **12** with 2 equiv of Tf_2_O and 20 equiv of pyridine at −10 °C (Scheme 3B). A rapid aqueous workup followed by washing with saturated copper­(II) sulfate solution to remove pyridine, solvent removal under reduced pressure below 20 °C, and coevaporation of the triflate with toluene (three times) minimized degradation and suppressed hydrolysis of the triflate during the coupling reaction. Upon exposure of triflate **28** to the dry difluoromethylene bisphosphonate nucleophile **21** in DCM, product **26** was formed in 51% yield (entry 9). The use of molecular sieves slightly increased the yield (entry 10). Ultimately, the substitution of **28** with 1 equiv of compound **23** provided the fully protected CF_2_ UDP-carbagalactose **24** in 60% yield (entry 11). Alternatively, this compound could also be obtained from intermediate **26** by demethylation and subsequent condensation of the resulting salt with primary alcohol **18** in the presence of Wada’s reagent. This procedure delivered **24** in 58% yield over two steps from **26**. Deprotection of **24** was achieved by removal of the ethyl groups with thiophenol in a mixture of MeCN/Et_3_N. Purification of the resulting product via preparative HPLC was required to remove side product resulting from cleavage of the carbagalactose phosphonate or uridine moieties to afford intermediate **29** in 53% yield. Deprotection of all PMB- and Boc-groups was accomplished in a single step by treating compound **29** with TFA and TES to give the CF_2_ UDP-carbagalactose **6** in 84% yield.

Analogues **5** and **6** were evaluated in an *in vitro* biochemical assay for the inhibition of recombinant human A4GALT (also known Gb3 synthase), an potential therapeutic target for Fabry disease, using C12-NBD-LacCer as the acceptor and UDP-Gal as the donor (see SI for details).^21^ Both P–CH_2_–P and P–CF_2_–P analogues **5** and **6** inhibited A4GALT, with half-maximal inhibitory concentration (IC_50_) values of 249 μM and 217 μM, respectively. Considering the relatively high concentration of UDP-Gal substrate (500 μM) used in the assay, these results indicate that simultaneous replacement of the glycosidic linkage and pyrophosphate moiety of UDP-Gal is well tolerated by A4GALT, with both chemically stabilized analogues retaining substantial recognition by the enzyme under these competitive assay conditions.

In conclusion, we have successfully designed and synthesized (difluoro) methylene bisphosphonate UDP-carbagalactose analogues as nonhydrolyzable UDP-Gal mimetics. The analogues incorporate carbagalactose and P–CH_2_–P and P–CF_2_–P motifs, thereby enhancing chemical and enzymatic stability while retaining key structural features essential for biological recognition. The successful construction of the P–CF_2_–P linkage was achieved through a nucleophilic substitution between a triflate and the tetrabutylammonium salt of a difluoromethylene bisphosphonate. This approach represents the first example of coupling between a secondary alcohol and a difluoromethylene bisphosphonate reagent, paving the way for the construction of a range of P–CF_2_–P linked sugar nucleotide analogues that will be of biological or medicinal interest. Notably, the inhibitory activity of compounds **5** and **6** is within the same order of magnitude as the concentration of the natural UDP-Gal substrate present in the assay, supporting their further evaluation as stabilized UDP-Gal mimetics and as molecular probes for structural studies of A4GALT, Such studies may help map the active site architecture and ultimately facilitate the development of strategies to block Gb3 production. Future development will require assessment of their activity across UDP-Gal-utilizing galactosyltransferases and, for cellular applications, strategies to overcome the limited membrane permeability expected from their charged bisphosphonate functionalities.

## Supplementary Material



## Data Availability

The data underlying this study are available in the published article and its Supporting Information.

## Notes

Experimental details, ^1^H and ^13^C NMR spectra for all new compounds (PDF)
