# Supplementary material for: Uridine Bisphosphonate-Carbagalactose: A Stabilized α‑1,4-Galactosyltransferase Inhibitor
Source: Org Lett. 2026 Oct 2;28(41):12965–70. doi: 10.1021/acs.orglett.6c03808 (PMC13682203; doi:10.1021/acs.orglett.6c03808)
Supplement: Supplementary file 1 [file ol6c03808_si_001.pdf]

Supporting Information (SI)

Uridine Bisphosphonate-Carbagalactose: a Stabilized α-1,4-Galactosyltransferase Inhibitor

Jianyun Guo, Nicky de Koster, Bob van Puffelen, Roy Steneker, Vincent Verhoeks, Tim P. Ofman, Herman S. Overkleeft, Marta Artola, Dmitri V. Filippov,* and Jeroen D. C. Codée*

†Leiden Institute of Chemistry, Leiden University, Einsteinweg 55, 2333 CC Leiden, The Netherlands

*Email: jcodee@chem.leidenuniv.nl; filippov@lic.leidenuniv.nl

Contents

1. General Information 3
2. Experimental Procedures 4
  2.1 Synthesis 4
  2.2 Biochemical Characterization 15
3. References 17
4. NMR Spectra 18

1. General Information

All chemicals were commercial grade and used as received and all moisture sensitive reactions were performed under an argon or nitrogen atmosphere, at ambient temperature (21°C), unless stated otherwise. For TLC analysis were used aluminium sheets (Merck, TLC silica gel 60 F_{254}), sprayed with a solution of H_2SO_4 (20%) in EtOH or with a solution of $(NH_4)_6Mo_7O_{24}\cdot 4H_2O$ (25 g/L) and $(NH_4)_4Ce(SO_4)_4\cdot 2H_2O$ (10g/L) in 10% aqueous H_2SO_4 or with a solution of $KMnO_4$ (2%) and K_2CO_3 (1%) in H_2O and then heated at ~150°C. For column chromatography was used 60Å silica gel (40-63 µm, SD Screening Devices). NMR spectra (^{1}H, ^{13}C, ^{19}F and ^{31}P) were recorded with a Bruker AV-400liq, Bruker AV-500 or a Bruker AV-600. Chemical shifts (δ) are given in ppm relative to tetramethylsilane as internal standard (^{1}H NMR in $CDCl_3$) or the residual signal of the deuterated solvent. Chemical shifts for ^{31}P spectra are indirectly referenced to H_3PO_4 (0.00 ppm) according to the IUPAC method. ^{31}P NMR spectra measured to monitor reactions were made by charging an NMR tube with an aliquot of the reaction mixture and fitting the tube with an acetone-d_6 capillary. Coupling constants (J) are given in Hz. All ^{13}C NMR and ^{31}P NMR spectra are proton decoupled. NMR peak assignments were made using COSY and HSQC experiments. LC/MS analysis was performed on a Surveyor HPLC system (Thermo Finnigan) equipped with a C18 column (Gemini, 4.6 mm x 50 mm, 5 µm particle size, Phenomenex), coupled to a LCQ Adventage Max (Thermo Finnigan) ion-trap spectrometer (ESI^+). Solvent system: A: Milli-Q H_2O, B: MeCN, C: 1% TFA (aq). High resolution mass spectra were recorded by direct injection on a mass spectrometer (Thermo Finnigan LTQ Orbitrap) equipped with an electrospray ion source in positive ion mode (source voltage 3.5 kV, sheath gas flow 10, capillary temperature 275°C) with resolution R = 60000 at m/z 400 (mass range m/z = 150-4000) and dioctyl phthalate (m/z = 391.28428) as a lock mass. Size-exclusion chromatography was performed on Sephadex™ LH-20 gel using CH_2Cl_2/MeOH (1:1, v/v) as the eluent, or on a TOYOPEARL HW-40-S resin column (16 × 600 mm) using an aqueous NH_4OAc buffer (0.15 M) containing 10% MeCN at a flow rate of 1 mL/min. Semi-preparative high-performance liquid chromatography (semiprep-HPLC) was performed on a Gilson system (322-H2 Pump, GX-281 Liquid Handler, UV/VIS-156 detector) equipped with a semi-preparative C18 column (NUCLEODUR C18 Gravity, 5 µm, 250 × 10 mm, Macherey Nagel) using Solvent A: 25 mM NH_4OAc in Milli-Q® H_2O; solvent B: acetonitrile; linear gradient of B in A (50 → 100% A/B), 5 mL/min.

2. Experimental Procedures

2.1 Synthesis

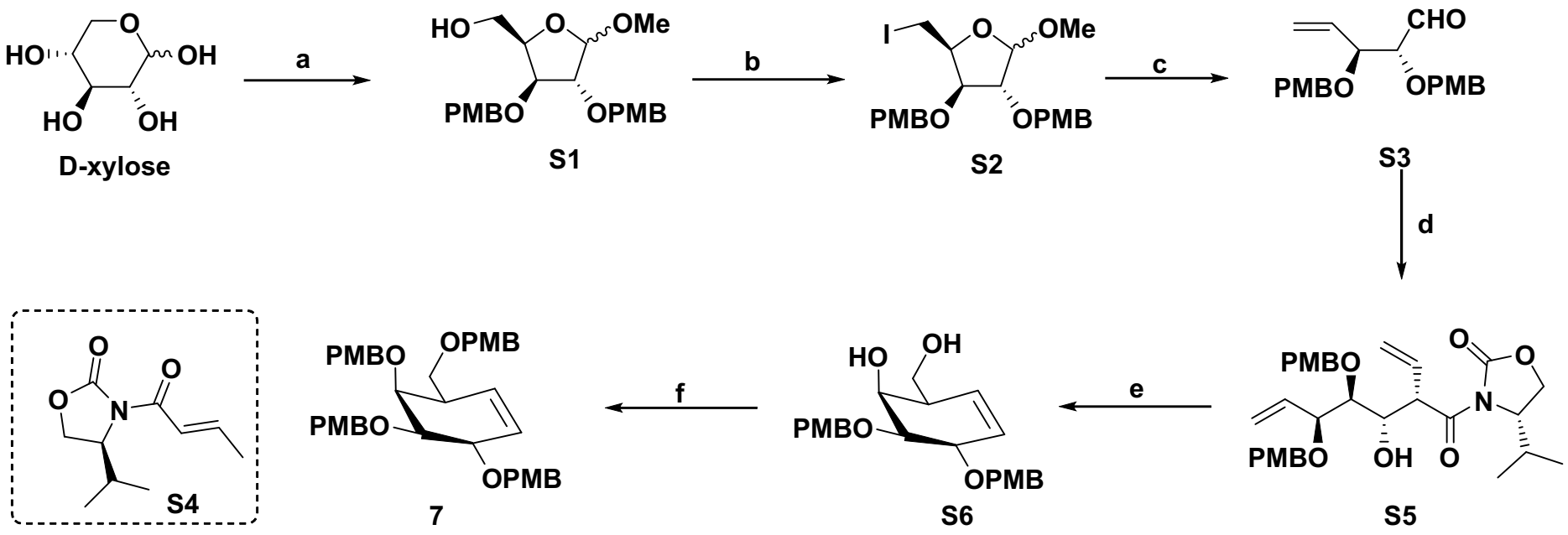

Scheme SI-1: Synthesis of cyclohexene derivative **7**. Reagents and conditions: a) (i) MeOH, AcCl (cat.), rt; (ii) TrtCl, DMAP, Et_3N, DMF, rt; (iii) PMBCl, NaH, DMF, 0 °C to rt; (iv) *p*-TsOH, DCM:MeOH (1:1, v/v), rt, 72 over 4 steps. b) I_2, PPh_3, imidazole, THF, 65 °C, 77%. c) zinc dust, THF/H_2O (9:1, v/v), ultrasound, 40°C, 94%. d) **S4**, Bu_2BOTf, Et_3N, -78 °C to rt, overnight, 94%. e) (i) $LiBH_4$, THF, 0 °C. (ii) Grubbs's second-generation catalyst, DCM, reflux, 95% over 2 steps. f) PMBCl, NaH, DMF, 0 °C to rt, 86%.

Methyl 2,3-di-O-(4-methoxybenzyl)-*α*/*β*-D-xylofuranoside (S1)

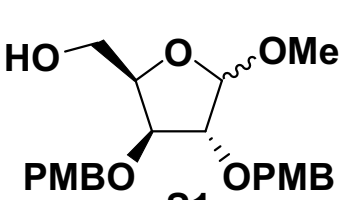

D-Xylose (30.0 g, 200 mmol) was dissolved in MeOH (600 mL) and cooled on ice. Acetyl chloride (6 mL, 84 mmol) was added and the reaction mixture was stirred for 5 h at room temperature until full conversion was reached (TLC: R_f = 0.35 (H_2O:MeCN, 1:9, v/v). The reaction was quenched by the addition of Et_3N until pH 8 was observed and subsequently concentrated to dryness under reduced pressure. The crude product was co-evaporated with toluene thrice and then dissolved in DMF (500 mL), and trityl chloride (67.0 g, 240 mmol), Et_3N (44.6 mL, 320 mmol), and DMAP (1.2 g, 10 mmol) were added. The reaction mixture was stirred for 24 h at room temperature. After full conversion was observed (TLC: R_f = 0.5 (EtOAc/pentane, 6:4, v/v)), the reaction was quenched by the addition of MeOH and concentrated under reduced pressure. The resulting oil was subsequently redissolved in Et_2O, washed with water, dried with MgSO4, filtered, and concentrated. The resulting oil was co-evaporated with toluene thrice, dissolved in DMF (500 mL) and cooled to 0 °C, NaH (60% dispersion in mineral oil, 32 g, 800 mmol) was added portionwise and stirred for 20 min, after which TBAI (3.7 g, 5 mmol) and PMBCl (96 mL, 700 mmol) were added, the reaction mixture was stirred at 0 °C for 1 h and then allowed to warm to ambient temperature. After 6 h, when TLC showed full conversion (TLC: R_f = 0.75 (EtOAc/pentane, 1:1, v/v), the reaction mixture was cooled to 0 °C and quenched by the addition of MeOH. The solution was further diluted with water and subsequently extracted with Et_2O, dried with $MgSO_4$, filtered, and concentrated *in vacuo*. The resulting product was redissolved in DCM (500 mL) and MeOH (500 mL), and *p*-TsOH was added until pH reached 2. The reaction mixture was stirred at room temperature for 18 h. Upon full conversion (TLC: R_f = 0.2 and 0.25 (EtOAc/Pentane 4:6 v/v)), the reaction was quenched with saturated $NaHCO_3$. The layers were separated, and the water layer was extracted with EtOAc. The combined organic layers were dried with $MgSO_4$, filtered, and concentrated under reduced pressure. Purification by flash column chromatography (EtOAc/pentane 3:7 to 4:6, v/v) gave the compound **S1** (58 g, 143 mmol, 72% over 4 steps) as an anomeric mixture (α/β ratio 2:1). Spectral data was in accordance with literature precedent.[1] **S1-α**: ^{1}H NMR (400 MHz, $CDCl_3$) δ 7.28 – 7.18 (m, 4H, CH_{arom}), 6.92 – 6.83 (m, 4H, CH_{arom}), 4.87 (d, *J* = 1.9 Hz, 1H, H-1), 4.56 (d, *J* = 11.7 Hz, 1H, C*H*H PMB), 4.51 (d, *J* = 11.5 Hz, 1H, CH*H* PMB), 4.46 (d, *J* = 11.3 Hz, 1H, C*H*H PMB), 4.40 (d, *J* = 11.7 Hz, 1H, CH*H* PMB), 4.27 (dt, *J* = 6.9, 4.8 Hz, 1H, H-4), 4.18 – 4.12 (m, 1H, H-3), 4.06 (dd, *J* = 3.9, 1.9 Hz, 1H, H-2), 3.80 (s, 3H, OMe), 3.79 (d, *J* = 3.5 Hz, 3H, OMe), 3.78 – 3.71 (m, 2H, H-5), 3.40 (s, 3H, 1-OMe), 2.57 (t, *J* = 6.6 Hz, 1H, 5-OH). ^{13}C NMR (101 MHz, $CDCl_3$) δ 159.5, 159.4, 130.4 ($C_{q\text{-}arom}$), 129.6, 129.5, 113.9, 113.9, 113.8, 113.8, 113.7 (CH_{arom}), 108.0 (C-1), 86.9 (C-2), 82.5 (C-3), 80.5 (C-4), 72.1, 71.9 (CH_2 PMB), 62.3 (C-5), 55.7 (1-OMe), 55.3 (OMe). **S1-β**: ^{1}H NMR (400 MHz, $CDCl_3$) δ 7.32 – 7.28 (m, 2H, CH_{arom}), 7.23 – 7.18 (m, 2H, CH_{arom}), 6.91 – 6.84 (m, 4H, CH_{arom}), 4.77 (d, *J* = 4.2 Hz, 1H, H-1), 4.64 (d, *J* = 11.4 Hz, 1H, CH*H* PMB), 4.59 (d, *J* = 11.6 Hz, 1H, C*H*H PMB), 4.52 (d, *J* = 11.6 Hz, 1H, CH*H* PMB), 4.48 (d, *J* = 11.4 Hz, 1H, C*H*H PMB), 4.39 (dd, *J* = 7.8, 6.4 Hz, 1H, H-3), 4.17 (dt, *J* = 7.7, 3.8 Hz, 1H, H-4), 4.01 (dd, *J* = 6.5, 4.2 Hz, 1H, H-2), 3.80 (s, 6H, OMe), 3.78 – 3.69 (m, 2H, H-5), 3.37 (s, 3H, 1-OMe), 2.45 (dd, *J* = 8.6, 5.0 Hz, 1H, 5-OH). ^{13}C NMR (101 MHz, $CDCl_3$) δ 159.5, 159.4 ($C_{q\text{-}arom}$), 129.9 (CH_{arom}), 129.7, 129.5 ($C_{q\text{-}arom}$), 129.5, 129.4, 113.9, 113.9, 113.9, 113.7 (CH_{arom}), 100.2 (C-1), 84.2 (C-2), 81.9 (C-3), 76.2(C-4), 72.4, 72.3 (CH_2 PMB), 62.3 (C-5), 55.3, 55.3 (OMe), 55.1 (1-OMe). **HRMS** (ESI) m/z: $[M+H]^+$ Calcd for $C_{22}H_{28}O_7Na$ 427.1727; Found 427.1727.

Methyl 2,3-di-*O*-(4-methoxybenzyl)-5-deoxy-5-iodo-*α*/*β*-D-xylofuranoside (S2)

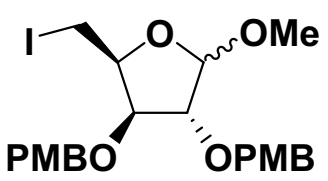

S2

Compound **S1** (45.0 g, 111 mmol) was co-evaporated with toluene, after which it was dissolved in anhydrous THF (500 mL). Triphenylphosphine (43.3 g, 165 mmol), imidazole (15.0 g, 220 mmol) and iodine (20.8 g, 78.8 mmol) were added, and the reaction mixture was heated to reflux until TLC analysis revealed full conversion of the starting material (4 h). The reaction mixture was cooled to room temperature and quenched with sat. aq. $Na_2S_2O_3$ solution. The mixture was extracted with Et_2O. The combined organic layers were washed with brine, dried over MgSO4 and concentrated under reduced pressure. Purification by flash column chromatography (R_f= 0.3, EtOAc/pentane 1:4, v/v) afforded compound **S2** (α/β ratio 4:1, 44.1 g, 85.8 mmol, 77%) as a yellow oil. Spectral data was in accordance with literature precedent.[2] **S2-α**: **^{1}H NMR** (400 MHz, $CDCl_3$) δ 7.28 – 7.20 (m, 4H, CH_{arom}), 6.91 – 6.86 (m, 4H, CH_{arom}), 4.91 (d, *J* = 1.3 Hz, 1H, H-1), 4.53 (d, *J* = 11.5 Hz, 1H, C*H*H PMB), 4.49 – 4.39 (m, 4H, H-4, C*H*H PMB, CH*H* PMB, C*H*H PMB), 4.01 (dd, *J* = 5.7, 2.7 Hz, 1H, H-3), 3.98 (dd, *J* = 2.9, 1.3 Hz, 1H, H-2), 3.81 (s, 6H, OMe), 3.42 (s, 3H, 1-OMe), 3.41 – 3.31 (m, 2H, H-5). **^{13}C NMR** (101 MHz, $CDCl_3$) δ 159.6 ($C_{q\text{-}arom}$), 129.8 (CH_{arom}), 129.7 ($C_{q\text{-}arom}$), 129.6 (CH_{arom}), 129.5 ($C_{q\text{-}arom}$), 114.0, 114.0 (CH_{arom}), 108.5 (C-1), 86.5(C-2), 82.1 (C-4), 81.7 (C-3), 72.5 (CH_2 PMB), 71.9 (CH_2 PMB), 56.2 (1-Ome), 55.4 (OMe), 4.8 (C-5). **S2-β**: **^{1}H NMR** (400 MHz, $CDCl_3$) δ 7.31 – 7.22 (m, 4H, CH_{arom}), 6.91 – 6.85 (m, 4H, CH_{arom}), 4.82 (d, *J* = 4.2 Hz, 1H, H-1), 4.62 – 4.42 (m, 4H, C*H*H PMB, CH*H* PMB, CH*H* PMB, C*H*H PMB), 4.38 (dt, *J* = 7.5, 6.1 Hz, 1H, H-4), 4.15 (dd, *J* = 6.5, 4.9 Hz, 1H, H-3), 4.01 – 3.94 (m, 1H, H-2), 3.81 (s, 6H, OMe), 3.41 (s, 3H, 1-OMe), 3.32 (dd, *J* = 10.1, 5.9 Hz, 1H, H-5), 3.18 (dd, *J* = 10.2, 7.5 Hz, 1H, H-5). **^{13}C NMR** (101 MHz, $CDCl_3$) δ 159.6, 159.4 ($C_{q\text{-}arom}$), 130.0 (CH_{arom}), 129.7 ($CH_{q\text{-}arom}$), 129.6, 114.0, 113.9 (CH_{arom}), 101.0 (C-1), 83. 6 (C-2), 81.5 (C-3), 77.7 (C-4), 72.5 (CH_2 PMB), 72.4 (CH_2 PMB), 55.7 (OMe), 55.4 (OMe), 55.4 (OMe), 3.3 (C-5). **HRMS** (ESI) m/z: $[M+Na]^+$ Calcd for $C_{22}H_{27}IO_6Na$ 537.0745; Found 537.0744.

(2*R*, 3*S*)-2,3-di-O-(4-methoxybenzyl)-pent-4-enal (S3)

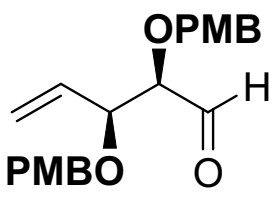

S3

Zinc dust was activated and dried immediately before use: Zn dust was added slowly to a vigorously stirred HCl solution (3 M). The mixture was stirred for 20 min at ambient temperature, filtered, and the solids were subsequently rinsed with water, acetone, and Et_2O, after which it was dried under high vacuum at 45 °C. A solution of compound **S2** (20.0 g, 34.8 mmol) in THF/H_2O (9:1, v/v, 348 mL) was sonicated at 40 °C under an argon atmosphere, after which the activated zinc dust (34.0 g, 522 mmol) was added. The reaction mixture was flushed again with argon and sonicated for 2 h at 40 °C. After TLC showed full conversion, the reaction was filtered on a celite pad and rinsed with Et_2O. The organic and aqueous layers were separated, and the aqueous layer was extracted with Et_2O. The combined organic layers were washed with H_2O, dried over $MgSO_4$ and concentrated under reduced pressure. Flash column chromatography (R_f= 0.3, pentane/Et_2O 2:1, v/v) of the crude product yielded compound **S3** as a colorless oil (11.6 g, 32.6 mmol, 94%). Spectral data were in accordance with literature precedent.[2] **^{1}H NMR** (400 MHz, $CDCl_3$) δ 9.61 (d, *J* = 1.6 Hz, 1H, H-1), 7.28 – 7.22 (m, 2H, CH_{arom}), 7.20 – 7.10 (m, 2H, CH_{arom}), 6.90 – 6.82 (m, 4H, CH_{arom}), 5.98 – 5.84 (m, 1H, H-4), 5.39 – 5.27 (m, 2H, H-5), 4.70 – 4.52 (m, 3H, C*H*H PMB, CH*H* PMB, C*H*H PMB), 4.27 (d, *J* = 11.8 Hz, 1H, C*H*H PMB), 4.14 – 4.10 (m, 1H, H-3), 3.80 (s, 6H, OMe), 3.77 (dd, *J* = 4.0, 1.6 Hz, 1H, H-2). **^{13}C NMR** (101 MHz, $CDCl_3$) δ 203.0 (C-1), 159.7, 159.4 ($C_{q\text{-}arom}$), 134.1 (C-4), 130.0, 129.7, 129.2 (CH_{arom}), 119.8 (C-5), 114.0, 113.9 (CH_{arom}), 85.0 (C-2), 79.7 (C-3), 73.3, 70.4 (CH_2 PMB), 55.4 (OMe). **HRMS** (ESI) m/z: $[M+Na]^+$ Calcd for $C_{21}H_{24}O_5Na$ 379.1516; Found 379.1516.

(*S*,*E*)-3-(But-2-enoyl)-4-isopropyloxazolidin-2-one (S4)

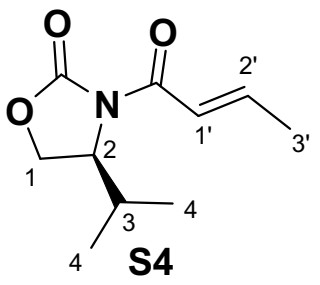

S4

(*S*)-4-isopropyloxazolidin-2-one (2.0 g, 15.5 mmol) was dissolved in dry THF (50 ml) and cooled to -78°C, *n*-butyl lithium (6.8 ml, 17 mmol, 2.5 M in hexanes) was added. After 15 min of stirring at −78 °C, (*E*)-2-butenoyl chloride (1.6 ml, 15.5 mmol) was added dropwise. The mixture was stirred at −78 °C for a further 30 min and then at room temperature for 1h. Upon full conversion on TLC, the reaction was quenched with saturated NH_4Cl aqueous solution. The mixture was diluted with sat. aq. $NaHCO_3$, and extracted with EtOAc, the combined organic layers were washed with brine, dried over $MgSO_4$, filtered and concentrated under reduced pressure. The crude product was purified by flash column chromatography (R_f= 0.4, pentane/EtOAc 4:1, v/v) to give **S4** as a white solid (2.67 g, 13.5 mmol, 87%). Spectral data was in accordance with literature precedent.[3] **^{1}H NMR** (400 MHz, $CDCl_3$) δ 7.32 – 7.26 (m, 1H, H-1’), 7.16 (m, 1H, H-2’), 4.52 – 4.46 (m, 1H, H-2), 4.33 – 4.24 (m, 1H, H-1), 4.22 (dd, *J* = 9.1, 3.2 Hz, 1H, H-1), 2.46 – 2.36 (m, 1H, H-3), 1.96 (dd, *J* = 6.8, 1.5 Hz, 3H, H-3’), 0.93 (d, *J* = 7.0 Hz, 3H, H-4), 0.89 (d, *J* = 7.0 Hz, 3H, H-4). **^{13}C NMR** (101 MHz, $CDCl_3$) δ 146.9 (C-2), 122.0 (C-1’), 63.5 (C-1), 58.6 (C-2), 28.6 (C-3), 18.7 (C-3’), 18.1, 14.8 (C-4). **HRMS** (ESI) m/z: $[M+Na]^+$ Calcd for $C_{10}H_{15}NO_3Na$ 220.0944; Found 220.0948.

Diene S5

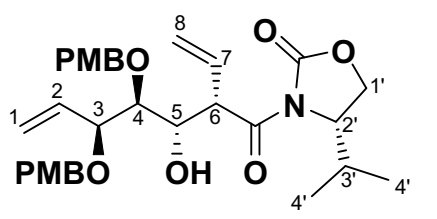

S5

(*S*,*E*)-3-(But-2-enoyl)-4-isopropyloxazolidin-2-one **(S4)** (18.3 g, 92.7 mmol) was co-evaporated with dry toluene and dissolved in dry DCM (150 mL). A solution of 1.0 M dibutylboryltrifluoromethanesulfonate in DCM (92.7 mL, 92.7 mmol) was added at –78 °C and stirred at this temperature for 10 min. Distilled Et_3N (16.4 mL, 123.6 mmol) was added and the reaction mixture was stirred for 1 h at -78°C, followed by 15 minutes at 0°C. After the reaction mixture was cooled back to –78°C and maintained at this temperature, a solution of aldehyde **S3** (22 g, 61.8 mmol) in dry DCM (70 mL) was added to the reaction mixture, after which the reaction mixture was slowly warmed to -30 °C and then was kept at this temperature overnight. Upon full conversion (as judged by TLC), the reaction was quenched with pH 7.0 phosphate buffer solution (75 mL). An aqueous 30% H_2O_2 solution was then added dropwise while maintaining the internal temperature below 5 °C. The mixture was stirred for an additional 1h while slowly warming to room temperature. The reaction was then poured over sat. aq. $NaHCO_3$ (200 mL) and the aqueous layer was extracted with DCM. The combined organic layers were dried over $MgSO_4$, filtered, and concentrated under reduced pressure. Flash column chromatography (R_f = 0.3, pentane/Et_2O 6:4) afforded the compound **S5** as a colorless oil (32.3 g, 58.4 mmol, 94%). **^{1}H NMR** (400 MHz, $CDCl_3$) δ 7.26 – 7.20 (m, 4H, CH_{arom}), 6.88 – 6.82 (m, 4H, CH_{arom}), 6.07 – 5.97 (m, 1H, H-2), 5.95 – 5.85 (m, 1H, H-7), 5.43 – 5.34 (m, 3H, H-1, H-8), 5.28 (dd, *J* = 10.2, 1.5 Hz, 1H, H-8), 5.01 – 4.92 (m, 1H, H-6), 4.65 – 4.54 (m, 2H, C*H*H PMB, CH*H* PMB), 4.46 – 4.37 (m, 2H, C*H*H PMB, H-5), 4.34 (d, *J* = 11.4 Hz, 1H, C*H*H PMB), 4.27 – 4.20 (m, 1H, H-3), 4.11 – 4.03 (m, 1H, H-2'), 3.91 (dd, *J* = 9.0, 3.1 Hz, 1H, H-1'), 3.79 (d, *J* = 2.8 Hz, 6H, OMe), 3.51 (dd, *J* = 8.3, 3.8 Hz, 1H, H-4), 3.45 (t, *J* = 8.8 Hz, 1H, H-1'), 3.38 (d, *J* = 2.2 Hz, 1H, 5-OH), 2.29 – 2.16 (m, 1H, H-3'), 0.79 (d, *J* = 7.0 Hz, 3H, H-4'), 0.74 (d, *J* = 6.9 Hz, 3H, H-4'). **^{13}C NMR** (101 MHz, $CDCl_3$) δ 172.7 (C=O, oxazolidinone), 159.3, 159.2 ($C_{q\text{-}arom}$), 153.5 (C=O, amide), 134.6 (C-2), 133.4 (C-7), 130.1 ($C_{q\text{-}arom}$), 129.8, 129.6, 129.0 (CH_{arom}), 120.5 (C-8), 119.0 (C-1), 114.0, 113.8, 113.6 (CH_{arom}), 81.3 (C-4), 79.4 (C-3), 72.7, 70.8 (CH_2 PMB), 70.7 (C5), 62.5 (C-1'), 58.0 (C-3'), 55.3, 55.3 (OMe), 49.9 (C-6), 28.0 (C-2'), 17.8, 14.5 (C-4'). **HRMS** (ESI) m/z: $[M+Na]^+$ Calcd for $C_{31}H_{39}NO_8Na$ 576.2568; Found 576.2567.

Carba-galactose alkene S6

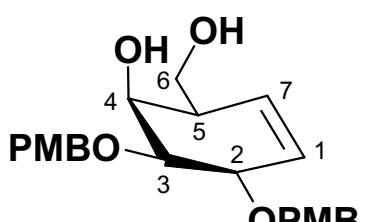

S6

Compound **S5** (32.0 g, 57.8 mmol) was dissolved in THF/H_2O (9:1, v/v; 600 mL), $LiBH_4$ (4.88 g, 224 mmol) was added at 0 °C. The reaction mixture was stirred at 0°C for 30 min and at room temperature for 2 h. After full conversion was observed, the reaction was quenched by the addition of 1 M NaOH on ice. The mixture was extracted with Et_2O, and the organic layer was separated. The combined organic layers were washed with sat. aq. $NaHCO_3$ solution and brine, dried over $MgSO_4$ and concentrated under reduced pressure. The residue was co-evaporated with dry toluene and then dissolved in dry DCM (1600 mL). 2nd generation Grubbs catalyst (2.4 g, 2.9 mmol, 5 mol%) was added, and the reaction mixture was stirred for 3 days at 40°C in the dark. Upon full conversion, the reaction mixture was concentrated in vacuo. Flash column chromatography of the crude product (R_f = 0.3, pentane/EtOAc 1:2, v/v) provided compound **S6** as a brown oil (21.7 g, 54.3 mmol, 94% over two steps). **^{1}H NMR** (400 MHz, $CDCl_3$) δ 7.31 – 7.25 (m, 4H, CH_{arom}), 6.90 – 6.84 (m, 4H, CH_{arom}), 5.83 (dt, *J* = 10.2, 2.6 Hz, 1H, H-7), 5.59 – 5.54 (m, 1H, H-1), 4.70 – 4.60 (m, 4H, C*H*H PMB, CH*H* PMB, C*H*H PMB, CH*H* PMB), 4.36 – 4.32 (m, 1H, H-3), 4.30 – 4.23 (m, 1H, H-2), 3.90 – 3.86 (dt, *J* = 11.6, 3.3 Hz, 1H, H-6), 3.83 – 3.76 (m, 7H, H-6, OMe), 3.64 (dd, *J* = 7.5, 2.4 Hz, 1H, H-4), 2.72 – 2.58 (m, 2H, 4-OH, 6-OH), 2.52 – 2.45 (m, 1H, H-5). **^{13}C NMR** (101 MHz, $CDCl_3$) δ 159.4, 159.3, 130.7, 130.2 ($C_{q\text{-}arom}$), 129.6, 129.5 (CH_{arom}), 127.7 (C-7), 126.8 (C-1), 114.0, 113.9 (CH_{arom}), 81.4 (C-3), 76.3 (C2), 72.0, 72.0 (CH_2 PMB), 70.4 (C-4), 63.7 (C-6), 55.4 (OMe), 41.9 (C-5). **HRMS** (ESI) m/z: $[M+Na]^+$ Calcd for $C_{23}H_{28}O_6Na$ 423.1778; Found 423.1781.

Carba-galactose alkene 7

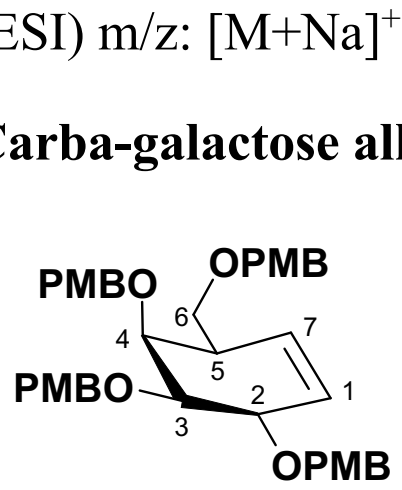

7

Compound **S6** (24.0 g, 60 mmol) was co-evaporated with toluene thrice and dissolved in DMF (500 mL), cooled to 0 °C, and sodium hydride (60% dispersion in mineral oil, 9.6 g, 240 mmol) was added and the reaction mixture was stirred for 20 min, after which TBAI (1.1 g, 3 mmol, 5, mol%) and PMBCl (23.5 mL, 180 mmol) were added, the reaction mixture was stirred at 0 °C for 1 h and then allowed to warm to ambient temperature. After 6 h, when TLC showed full consumption, the reaction mixture was cooled to 0 °C and quenched by the addition of MeOH. The solution was further diluted with water and subsequently extracted with Et_2O, dried with MgSO4, filtered, and concentrated. Flash column chromatography of the crude product (R_f = 0.3, pentane/EtOAc 4:1, v/v) provided compound **7** as a colorless oil (33.0 g, 51.5 mmol, 86%). **^{1}H NMR** (400 MHz, $CDCl_3$) δ 7.33 – 7.25 (m, 3H, CH_{arom}), 7.28 – 7.16 (m, 5H, CH_{arom}), 6.91 – 6.78 (m, 8H, CH_{arom}), 5.72 (m, 1H, H-7), 5.44 (d, *J* = 10.2 Hz, 1H, H-1), 4.83 (d, *J* = 11.2 Hz, 1H, C*H*H PMB), 4.71 – 4.52 (m, 5H, C*H*H PMB, CH*H* PMB, CH*H* PMB, C*H*H PMB, CH*H* PMB), 4.45 – 4.34 (m, 3H, H-2, C*H*H PMB, CH*H* PMB), 4.13 (dd, *J* = 3.2,

1.5 Hz, 1H, H-4), 3.84 – 3.76 (m, 12H, OMe), 3.68 (dd, *J* = 8.0, 1.8 Hz, 1H, H-3), 3.53 (t, *J* = 8.7 Hz, 1H, H-6), 3.42 (dd, *J* = 8.7, 6.4 Hz, 1H, H-6), 2.63 (m, 1H, H-5). **^{13}C NMR** (101 MHz, $CDCl_3$) δ 159.4, 159.2, 159.2 ($C_{q\text{-}arom}$), 131.4, 131.2, 131.1, 130.5 ($C_{q\text{-}arom}$), 129.9, 129.7, 129.6, 129.6 (CH_{arom}), 129.3 (C-7), 127.8 (C-1), 113.9, 113.9, 113.8, 113.7 (CH_{arom}), 83.0 (C-3), 77.5 (C-2), 74.9 (C-4), 73.8, 73.1, 72.2, 72.0 (CH_2 PMB), 70.1 (C-6), 55.4, 55.4 (OMe), 42.1 (C-5). **HRMS** (ESI) m/z: $[M+Na]^+$ Calcd for $C_{39}H_{44}O_8Na$ 663.2928; Found 663.2928.

Epoxide 8 and 9

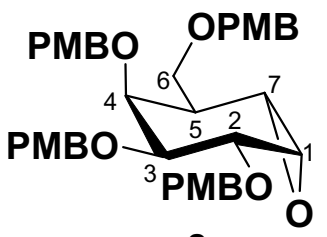

8

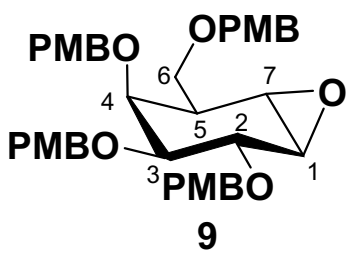

9

Alkene **7** (2.2 g, 3.4 mmol) was dissolved in DCM (17 mL), *m*-CPBA (77%, 1.5 g, 8.6 mmol) was added at 0 °C and the mixture was stirred at 0 °C overnight. Upon full conversion, the reaction mixture was quenched with sat. aq. $Na_2S_2O_3$ and sat. aq. $NaHCO_3$ and diluted with EtOAc, the layers were separated, and the water layer was extracted with EtOAc. The combined organic layers were dried with MgSO4 and concentrated under reduced pressure. Purification by silica gel column chromatography (R_f = 0.33 and 0.52 for **8** and **9** respectively, pentane/EtOAc 1:2, v/v) provided compound **9** as a colorless oil (1.4 g, 2.1 mmol, 61%) and compound **8** as a white amorphous solid (233 mg, 0.36 mmol, 10%). **8: ^{1}H NMR** (400 MHz, $CDCl_3$) δ 7.34 – 7.22 (m, 6H, CH_{arom}), 7.17 – 7.12 (m, 2H, CH_{arom}), 6.91 – 6.80 (m, 8H, CH_{arom}), 4.81 – 4.70 (m, 3H, CH*H* PMB, C*H*H PMB, CH*H* PMB), 4.65 – 4.57 (m, 2H, CH*H* PMB, C*H*H PMB), 4.47 – 4.38 (m, 3H, CH*H* PMB, C*H*H PMB, CH*H* PMB), 4.22 – 4.17 (m, 1H, H-2), 3.85 (dd, *J* = 3.2, 1.5 Hz, 1H, H-4), 3.84 – 3.77 (m, 12H, OMe), 3.55 (dd, *J* = 8.5, 1.4 Hz, 1H, H-3), 3.54 – 3.50 (m, 2H, H-6), 3.31 (dd, *J* = 3.9, 2.4 Hz, 1H, H-1), 2.91 (dd, *J* = 4.0, 1.5 Hz, 1H, H-7), 2.24 (m, H, H-5). **^{13}C NMR** (126 MHz, $CDCl_3$) δ 159.4, 159.4, 159.2, 159.1, 131.2, 130.9, 130.4, 130.4($C_{q\text{-}arom}$), 129.7, 129.6, 129.3, 114.0, 113.9, 113.9, 113.6 (CH_{arom}), 82.4 (C-3), 76.1(C-2), 74.0, 73.3, 73.1, 72.5 (CH_2 PMB), 72.5 (C-4), 68.8 (C-6), 55.4, 55.4, 55.4 (OMe), 54.7 (C-1), 52.7 (C-7), 40.4 (C-5). **HRMS (ESI) M/Z**: Calcd for $C_{39}H_{44}O_9Na$ 679.2878 $[M+Na]^+$; Found 679.2876. **9: ^{1}H NMR** (400 MHz, $CDCl_3$) δ 7.31 – 7.24 (m, 4H, CH_{arom}), 7.23 – 7.16 (m, 4H, CH_{arom}), 6.90 – 6.79 (m, 8H, CH_{arom}), 4.78 – 4.66 (m, 3H, CH*H* PMB, C*H*H PMB, CH*H* PMB), 4.61 (d, *J* = 2.2 Hz, 2H, CH*H* PMB, C*H*H PMB), 4.50 (d, *J* = 11.7 Hz, 1H, CH*H* PMB), 4.43 – 4.35 (m, 2H, C*H*H PMB, CH*H* PMB), 4.08 (d, *J* = 8.7 Hz, 1H, H-2), 3.89 – 3.84 (m, 1H, H-4), 3.83 – 3.76 (m, 12H, OMe), 3.65 (dd, *J* = 8.9, 7.0 Hz, 1H, H-6), 3.55 (dd, *J* = 8.9, 7.3 Hz, 1H, H-6), 3.39 (dd, *J* = 8.8, 2.1 Hz, 1H, H-3), 3.20 (d, *J* = 3.8 Hz, 1H, H-1), 3.16 – 3.12 (m, 1H, H-7), 2.29 – 2.21 (m, 1H, H-5). **^{13}C NMR** (101 MHz, $CDCl_3$) δ 159.3, 159.3, 159.1, 159.0, 131.1, 130.7, 130.3, 130.2 ($C_{q\text{-}arom}$), 129.6, 129.5, 129.2, 113.9, 113.8, 113.7, 113.5 (CH_{arom}), 82.3 (C-3), 75.9 (C-2), 73.8, 73.1, 73.0, 72.4 (CH_2 PMB), 72.3 (C-4), 68.7 (C-6), 55.3, 55.3, 55.3 (OMe), 54.6 (C-1), 52.6 (C-7), 40.3 (C-5). **HRMS** (ESI) m/z: $[M+Na]^+$ Calcd for $C_{39}H_{44}O_9Na$ 679.2878; Found 679.2877.

Table SI-1 Explored conditions for the ring-opening reaction of epoxide **8**

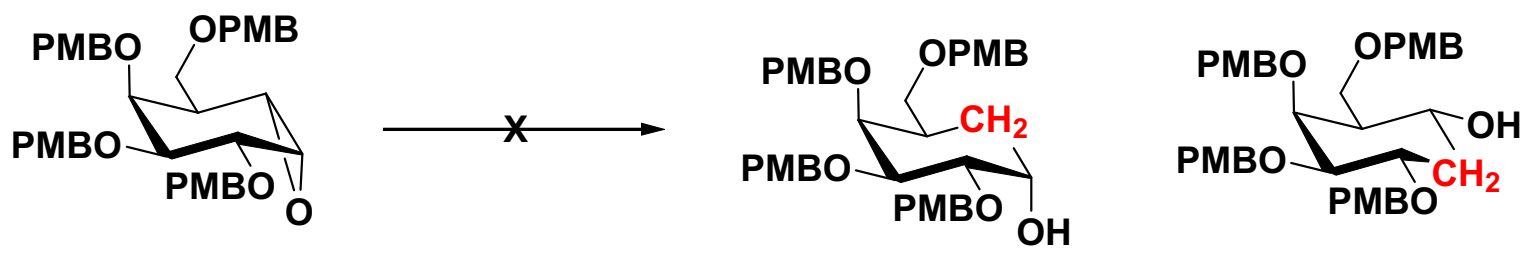

8 **12** **S7**

entry	reducing agent	**12** : **S7**	temperature	time	yield (%)
1	$LiBH_4$	-	rt-reflux	48 h	NR
2	$LiHBEt_3$	-	rt-reflux	48 h	trace conversion with degradation
3	$LiAlH_4$	-	reflux	48 h	trace conversion with degradation
4	L-selectride	-	0 °C-rt	48 h	NR
5	L-selectride	-	reflux	48 h	trace conversion with degradation

Table SI-2 Optimization of the ring-opening reaction of epoxide **9**

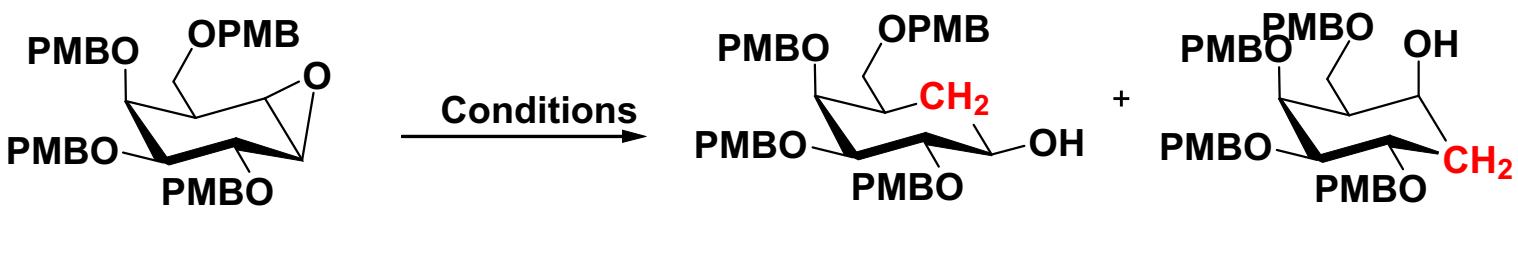

9 **10** **11**

entry	reducing agent	**10 :1 1**	temperature	time
1	$LiHBEt_3$	2:3 (38%:55%)	-15	5h
2	$LiAlH_4$	2:1 (45%:22%)	rt	4h
3	$LiAlH_4$	3:1 (54%:16%)	reflux	4h
4	L-selectride	6:1 (60%:10%)	rt	2 h
5	L-selectride	7:1 (71%:10%)	0 °C	4 h

β-Carbagalactose 10 and cyclitol 11

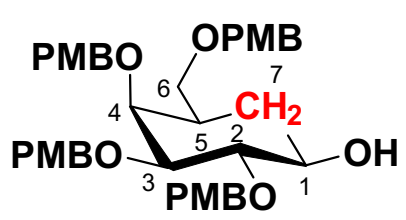

10

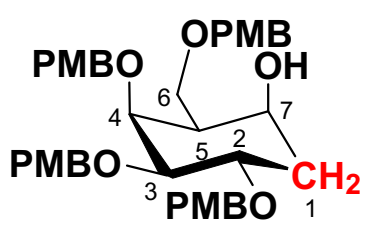

11

Epoxide **9** (3.0 g, 4.6 mmol) was co-evaporated with toluene three times and then dissolved in THF (46 mL). The solution was cooled to 0 °C, L-selectride (1.0 M in THF, 9.2 mL, 9.2 mmol) was added dropwise and the reaction was stirred for 4 h. After full conversion was observed, the reaction was quenched with water on ice. The mixture was extracted with DCM. The combined organic layers were dried over anhydrous Na_2SO_4, filtered and concentrated under reduced pressure. The residue was purified by silica gel column chromatography (R_f = 0.24 and 0.47 for **10** and **11** respectively, pentane/EtOAc 3:2, v/v) to provide compound **10** as a white amorphous solid (2.1 g, 3.2 mmol, 71%) and **11** as a colorless oil (305 mg, 0.46 mmol, 10%). **10:** ^{1}**H NMR** (400 MHz, $CDCl_3$) δ 7.33 – 7.29 (m, 2H, CH_{arom}), 7.26 – 7.19 (m, 6H, CH_{arom}), 6.90 – 6.83 (m, 8H, CH_{arom}), 4.95 (d, *J* = 10.9 Hz, 1H, CH*H* PMB), 4.91 (d, *J* = 11.0 Hz, 1H, C*H*H PMB), 4.71 (d, *J* = 11.2 Hz, 1H, CH*H* PMB), 4.59 (d, *J* = 11.0 Hz, 2H, CH*H* PMB, C*H*H PMB), 4.44 (d, *J* = 11.0 Hz, 1H, CH*H* PMB), 4.41 – 4.31 (m, 2H, CH*H* PMB, C*H*H PMB), 4.09 – 4.06 (m, 1H, H-4), 3.83 – 3.77 (m, 12H, OMe), 3.77 – 3.68 (m, 1H, H-2), 3.55 – 3.42 (m, 2H, H-6, H-1), 3.38 (dd, *J* = 9.6, 2.4 Hz, 1H, H3), 3.28 (dd, *J* = 8.8, 5.1 Hz, 1H, H-6), 2.38 – 2.34 (m, 1H, 1-OH), 1.78 – 1.69 (m, 1H, H-5), 1.67 – 1.61 (m, 1H, H-7), 1.51 (q, *J* = 12.4 Hz, 1H, H-7). ^{13}**C NMR** (101 MHz, $CDCl_3$) δ 159.4, 159.3, 159.2, 159.0, 131.7, 131.1, 130.8, 130.4 ($C_{q\text{-}arom}$), 129.8, 129.6, 129.2, 129.1, 114.1, 113.9, 113.9, 113.8, 113.6 (CH_{arom}), 84.6 (C-3), 83.4 (C-2), 75.1(CH_2 PMB), 74.3 (C-4), 74.2, 73.0, 72.1 (CH_2 PMB), 71.9 (C-1), 70.5 (C-6), 55.4 (OMe), 37.7 (C-5), 29.1 (C-7). **HRMS** (ESI) m/z: Calcd for $C_{39}H_{46}O_9Na$ 681.3034 $[M+Na]^+$; Found 681.3032. **11:** ^{1}**H NMR** (400 MHz, CDCl3) δ 7.38 – 7.25 (m, 6H, CH_{arom}), 7.23 – 7.14 (m, 2H, CH_{arom}), 6.94 – 6.84 (m, 8H, CH_{arom}), 4.92 (d, *J* = 10.7 Hz, 1H, CH*H* PMB), 4.78 (d, *J* = 11.3 Hz, 1H, C*H*H PMB), 4.72 (d, *J* = 10.8 Hz, 2H, C*H*H PMB, CH*H* PMB), 4.65 (d, *J* = 10.9 Hz, 1H, C*H*H PMB), 4.50 – 4.37 (m, 3H, CH*H* PMB, C*H*H PMB, CH*H* PMB), 4.27 (m, 1H, H-4), 4.20 – 4.11 (m, 1H, H-2), 4.00 – 3.92 (m, 1H, H-7), 3.86 – 3.82 (m, 12H, OMe), 3.73 – 3.67 (m, 2H, H-6, H-7), 3.67 – 3.61 (m, 1H, H-6), 3.46 (dd, *J* = 9.4, 2.4 Hz, 1H, H-3), 2.43 (m, 1H, H-1), 1.83 (s, 1H, H-5), 1.74 (s, 1H, 7-OH), 1.56 – 1.43 (m, 1H, H-1). ^{13}**C NMR** (101 MHz, $CDCl_3$) δ 159.4, 159.4, 159.2, 131.2, 131.1, 130.5, 130.3 ($C_{q\text{-}arom}$), 129.7, 129.7, 129.4, 129.2, 114.1, 114.0, 113.9, 113.9, 113.8, 113.8, 113.8 (CH_{arom}), 85.1 (C-3), 78.1 (C-4), 75.1 (CH_2 PMB), 74.8 (C-2), 73.2, 72.8, 72.6 (CH_2 PMB), 69.2 (C-7), 68.4 (C-6), 55.4, 55.4 (OMe), 43.8 (C-5), 38.9 (C-1). **HRMS** (ESI) m/z: $[M+Na]^+$ Calcd for $C_{39}H_{46}O_9Na$ 681.3034; Found 681.3032.

Figure-SI-1. Ring-opening pathways of β-Gal-Cyclophellitol **10** with L-selectride

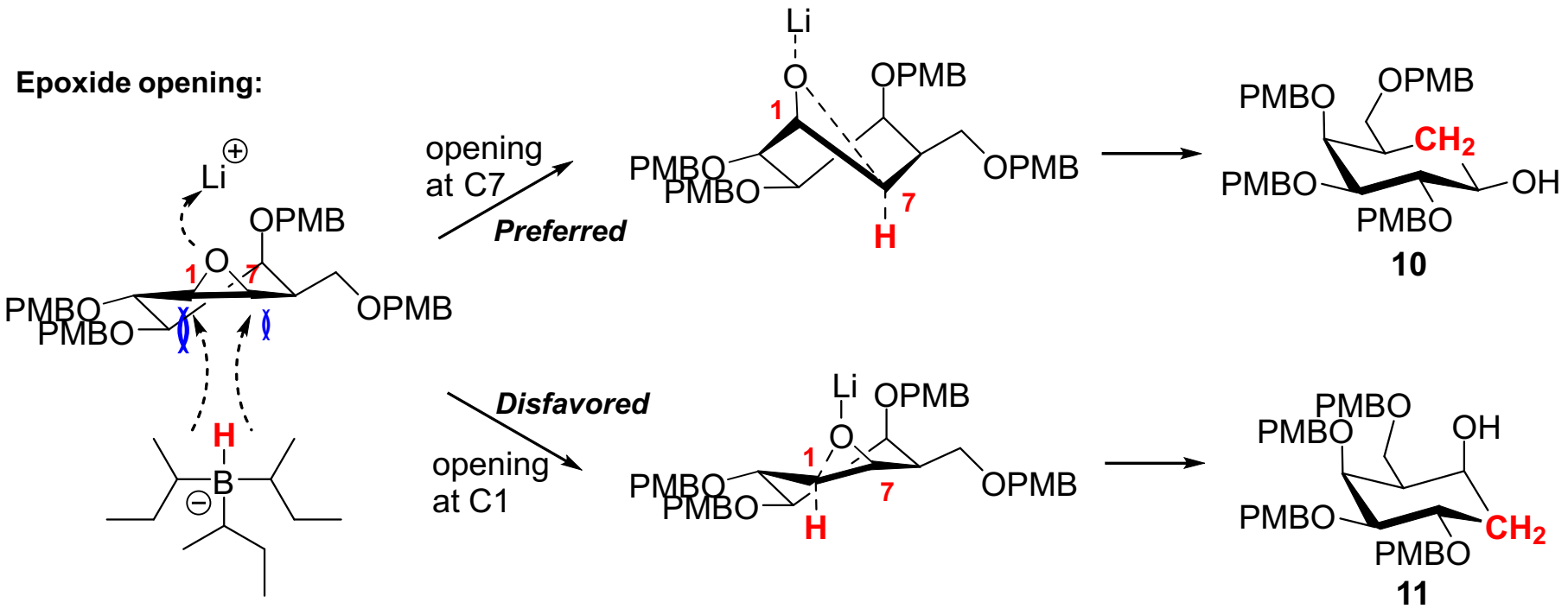

α-Carbagalactose 12

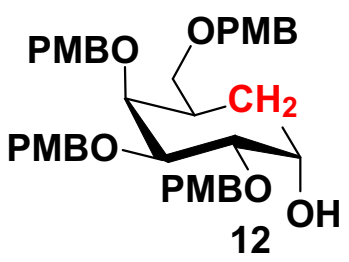

12

β-Carbagalactose **10** (100 mg, 0.15 mmol) was dissolved in THF (1 mL). PPh_3 (161 mg, 0.62 mmol) and *p*-nitrobenzoic acid (100 mg, 0.6 mmol) were added under argon atmosphere. Diisopropyl azodicarboxylate (DIAD) (124 µL, 0.63 mmol) was added at 0 °C, and the mixture was stirred for 2 h at room temperature. After the reaction was complete, the solvent was removed under reduced pressure to afford the crude ester. To a solution of this ester in MeOH (1 mL) was added K_2CO_3 (41 mg, 0.3 mmol) at room temperature. After the resulting mixture had been stirred for 3 h at rt, the reaction was quenched with H_2O. The mixture was extracted with EtOAc, and the combined organic layers were washed with water, brine, dried over Na_2SO_4, filtered, and concentrated. Purification by silica gel column chromatography (R_f = 0.2, pentane/EtOAc 2:1, v/v) afforded α-carbagalactose **12** as a colorless oil (76 mg, 76% over 2 steps). **^{1}H NMR** (400 MHz, $CDCl_3$) δ 7.36 – 7.18 (m, 8H, CH_{arom}), 6.92 – 6.83 (m, 8H, CH_{arom}), 4.89 (d, *J* = 10.8 Hz, 1H, C*H*H PMB), 4.74 – 4.69 (m, 2H, CH*H* PMB, C*H*H PMB), 4.66 (d, *J* = 11.3 Hz, 1H, CH*H* PMB), 4.74 – 4.59 (m, 4H, C*H*H PMB, C*H*H PMB, C*H*H PMB, CH*H* PMB), 4.15 – 4.09 (m, 2H, H-4, H-1), 3.86 (dd, *J* = 9.7, 3.3 Hz, 1H, H-2), 3.84 – 3.80 (m, 12H, OMe, OMe, OMe, OMe), 3.72 (dd, *J* = 9.7, 2.5 Hz, 1H, H-3), 3.46 (t, *J* = 9.3 Hz, 1H, H-6), 3.27 (dd, *J* = 8.9, 5.0 Hz, 1H, H-6), 2.56 (s, 1H, 1-OH), 2.31 – 2.19 (m, 1H, H-5), 1.61 – 1.50 (m, 2H, H-7). **^{13}C NMR** (101 MHz, $CDCl_3$) δ 159.3, 159.3, 159.1, 159.0, 131.9, 131.4, 130.9, 130.6 ($C_{q\text{-}arom}$), 129.6, 129.5, 129.4, 129.1, 113.9, 113.9, 113.8, 113.6 (CH_{arom}), 80.9 (C-3), 79.8 (C-2), 75.3 (C-4), 74.3, 72.8, 72.6, 72.5 (CH_2 PMB), 70.4 (C-6), 67.4 (C-1), 55.4, 55.4 (OMe), 35.2 (C-5), 27.7 (C-7). **HRMS** (ESI) m/z: $[M+Na]^+$ Calcd for $C_{39}H_{46}O_9Na$ 681.3034; Found 681.3032.

Reagent 15

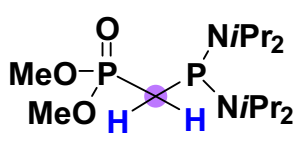

15

n-BuLi (2.5M in n-hexane, 3.4 mL, 8.4 mmol) was carefully added to a cooled solution (-78 ˚C) of diisopropylamine (1.2 mL, 8.4 mmol) in dry THF (3 mL), under argon atmosphere. The reaction mixture was stirred for 30 minutes. A solution of di-tert-butyl methylphosphonate (437 µL, 4.0 mmol) in THF (2 mL) was added dropwise over 5 minutes, and the reaction mixture was stirred for an additional 30 minutes. Next, a solution of bis(diisopropylamino)chlorophosphoamidite (1.3 g, 4.8 mmol) in n-hexane:THF (2:1 v:v, 3 mL) was prepared and added dropwise through a syringe fitted with a silica filter to remove insoluble diisopropylamine hydrochloride originating from the commercial $P^{(III)}$ reagent. After addition, the reaction mixture was allowed to warm to room temperature and stirred overnight. The reaction mixture was poured into saturated aqueous $NaHCO_3$ and extracted with EtOAc. The organic layer was dried over Na_2SO_4, filtered and concentrated in vacuo. Purification by silica gel column chromatography (pentane/ EtOAc 8:2 + 2% Et_3N) yielded the compound **15** as a colorless oil (1.3 g, 2.9 mmol, 72%). **^{1}H NMR** (400 MHz, CD_3CN) δ 3.66 (s, 3H, P(O)OMe), 3.63 (s, 3H, P(O)OMe), 3.44 (m, 4H, C*H*(CH_3)$_2$), 2.25 (d, *J* = 3.3 Hz, 1H, P-C*H*H-P), 2.21 – 2.19 (m, 1H, P-C*H*H-P), 1.19 (s, 6H, C*H*(CH_3)$_2$), 1.18 (s, 6H, CH(CH_3)$_2$), 1.13 (s, 6H, CH(CH_3)$_2$), 1.11 (s, 6H, CH(CH_3)$_2$) (The isopropyl methyls appear as four singlets (6 H each) due to high concentration of the sample). ^{13}C NMR (101 MHz, CD_3CN) δ 52.6 (d, $J_{C\text{-}P}$ = 2.3 Hz, P(O)OMe), 52.5 (d, $J_{C\text{-}P}$ = 2.2 Hz, P(O)OMe), 47.8, 47.7 (*C*H(CH_3)$_2$), 26.6 (dd, $J_{C\text{-}P}$ = 131.2, 29.5 Hz, P-*C*H_2-P), 24.5, 24.5, 24.1, 24.1 (CH(*C*H_3)$_2$. **^{31}P NMR** (162 MHz, CD_3CN) δ 38.25 (d, $J_{P\text{-}P}$ = 79.6 Hz, P^{III}), 32.85 (d, $J_{P\text{-}P}$ = 78.8 Hz, P^V). **HRMS** (ESI) m/z: $[M+Na]^+$ Calcd for $C_{15}H_{36}N_2O_3P_2Na$ 377.2093; Found 377.2091.

Reagent 16

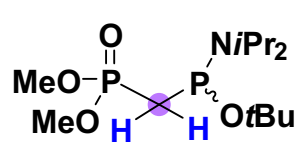

16

Compound **15** (1.6 g, 4.5 mmol) was co-evaporated with toluene, dissolved in MeCN and put under argon atmosphere. Dry *t*BuOH (337 mg, 4.5 mmol) and DCI (319 mg, 2.7 mmol, 0.6 eq.) were added. The reaction progression was followed by ^{31}P NMR, which indicated completion after 5 h. The reaction mixture was concentrated in vacuo, and the residue was purified by silica

gel column chromatography (DCM/acetone 4:1 + 2% Et_3N) to yield compound **16** as a colorless oil (1.0 g, 2.7 mmol, 76%). **^{1}H NMR** (400 MHz, $CDCl_3$) δ 3.75 (d, *J* = 2.1 Hz, 3H, P(O)OMe), 3.72 (d, *J* = 2.1 Hz, 3H, P(O)OMe), 3.59 – 3.44 (m, 2H, C*H*(CH_3)$_2$), 2.39 – 2.24 (m, 1H, P-C*H*H-P), 1.94 – 1.79 (m, 1H, P-C*H*H-P), 1.31 (s, 9H, C(CH_3)$_3$), 1.18 (d, *J* = 6.8 Hz, 6H, CH(CH_3)$_2$), 1.10 (d, *J* = 6.8 Hz, 6H, CH(CH_3)$_2$). **^{13}C NMR** (101 MHz, $CDCl_3$) δ 52.2, 52.1 (P(O)OMe), 44.8, 44.7 (*C*H(CH_3)$_2$, 31.6 (dd, $J_{c\text{-}p}$ = 130.5, 36.0 Hz, P-*C*H_2-P), 30.6, 30.5 (C(*C*H_3)$_3$), 23.7, 23.4, 22.7 (CH(*C*H_3)$_2$). **^{31}P NMR** (162 MHz, $CDCl_3$) δ 99.55 (d, $J_{p\text{-}p}$ = 45.6 Hz, P^{III}), 31.41 (d, $J_{p\text{-}p}$ = 45.4 Hz, P^V). **HRMS** (ESI) m/z: $[M+Na]^+$ Calcd for $C_{13}H_{31}NO_4P_2Na$ 350.1621; Found 350.1623.

Methylene bisphosphonate 17

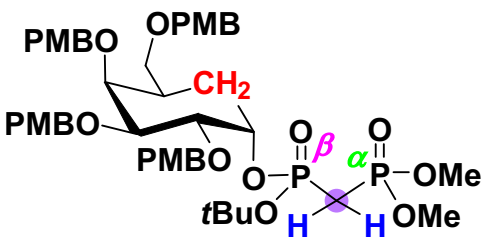

17

α-Carbagalactose **12** (155 mg, 0.24 mmol) and phosphanyl methylphosphonate **16** (116 mg, 0.35 mmol) were co-evaporated with toluene, dissolved in MeCN (2 ml), and put under argon atmosphere. DCI (0.25M in MeCN, 1.9 ml, 0.48 mmol) was added and the reaction mixture was stirred for 30 min at room temperature. The mixture was cooled to 0 °C, and tert-butyl hydroperoxide (5.0-6.0 M in decane, 0.44 ml, 2.4 mmol, 2 eq.) was added. The reaction mixture was stirred for an additional 30 minutes. The solution was poured into DCM, and the organic layer was washed with sat. aq. $NaHCO_3$ and brine. The organic layer was dried over Na_2SO_4 and concentrated *in vacuo*. Purification by silica gel column chromatography (R_f = 0.2, acetone/DCM 3:7) yielded compound **17** as a colorless oil (132 mg, 0.15 mmol, 62%). **^{1}H NMR** (400 MHz, $CDCl_3$) δ 7.36 – 7.29 (m, 4H, CH_{arom}), 7.25 – 7.12 (m, 4H, CH_{arom}), 6.91 – 6.79 (m, 8H, CH_{arom}), 5.09 (m, 1H, H-1), 4.87 (d, *J* = 10.7 Hz, 1H, C*H*H PMB), 4.76 – 4.65 (m, 3H, C*H*H PMB, C*H*H PMB, CH*H* PMB), 4.61 (d, *J* = 10.8 Hz, 1H, C*H*H PMB), 4.43 – 4.33 (m, 3H, C*H*H PMB, C*H*H PMB, CH*H* PMB), 4.12 – 4.07 (m, 1H, H-4), 3.88 – 3.83 (m, 2H, H-2, H3), 3.82 – 3.77 (m, 12H, OMe, OMe, OMe, OMe), 3.68 (d, *J* = 11.3 Hz, 3H, P(O)OMe), 3.60 (d, *J* = 11.3 Hz, 3H, P(O)OMe), 3.44 (t, *J* = 8.8 Hz, 1H, H-6), 3.24 (dd, *J* = 8.9, 5.6 Hz, 1H, H-6), 2.58 – 2.44 (m, 2H, P-C*H*H-P, P-CH*H*-P), 2.26 – 2.16 (m, 1H, H-5), 1.72 – 1.59 (m, 2H, H-7), 1.51 (s, 9H, C(CH_3)$_3$). **^{13}C NMR** (101 MHz, $CDCl_3$) δ 159.3, 159.2, 159.1, 159.1, 131.6, 131.4, 130.5 ($C_{q\text{-}arom}$), 130.1, 129.5, 129.4, 129.0, 129.0, 113.9, 113.8, 113.7, 113.6 (CH_{arom}), 80.4 (C-2), 78.6 (C-3), 75.6 (C-4), 74.9 (C-1), 74.8, 74.4, 72.8, 72.6 (CH_2 PMB), 70.4 (C-6), 55.4, 55.3 (OMe), 52.9 (d, $J_{c\text{-}p}$ = 6.4 Hz, P(O)OMe), 52.6 (d, $J_{c\text{-}p}$ = 6.3 Hz, P(O)OMe), 35.8 (C-5), 30.5, 30.4 (C(CH_3)$_3$), 28.7 (C-6), 26.1 (dd, $J_{C\text{-}P}$ =144.4, 135.6 Hz, P-CH_2-P). **^{31}P NMR** (162 MHz, $CDCl_3$) δ 23.59 (d, $J_{p\text{-}p}$ = 10.9 Hz, α-P), 16.04 (d, $J_{p\text{-}p}$ = 10.9 Hz, β-P). **HRMS** (ESI) m/z: $[M+Na]^+$ Calcd for $C_{46}H_{62}O_{14}P_2Na$ 923.3507; Found 923.3508.

3-*N*-(*tert*-Butoxycarbonyl)-2',3'-di-*O*-(*tert*-butoxycarbonyl)uridine 18

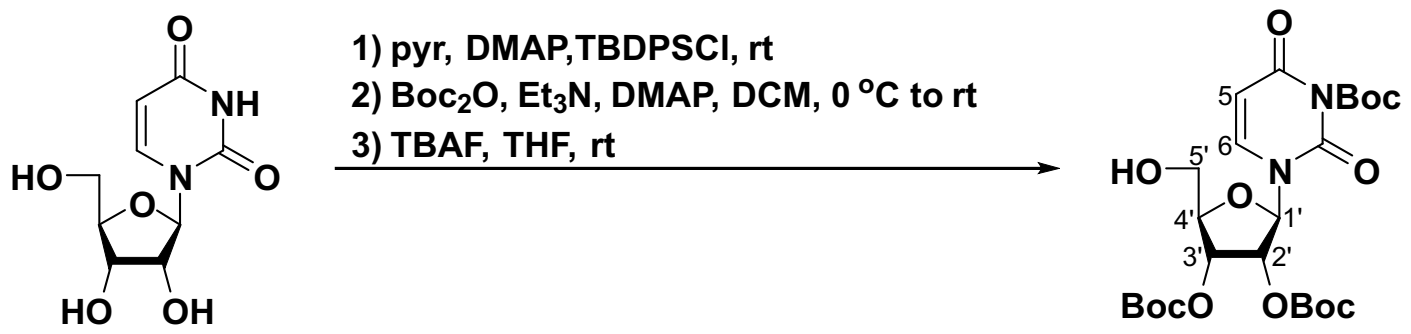

uridine 18

Uridine (10 g, 41 mmol) was dissolved in pyridine (50 ml), DMAP (501 mg, 4.1 mmol) and TBDPSCl (12.9 ml, 49 mmol) were added. The reaction was stirred overnight under N_2 atmosphere. Upon full conversion, as indicated by TLC, the reaction was quenched with MeOH, diluted with saturated aqueous $NaHCO_3$ and brine, and extracted with EtOAc. The combined organic layers were washed with brine, dried over $MgSO_4$, filtered and concentrated under reduced pressure. The crude product was co-evaporated with toluene thrice and dissolved in DCM (200 ml). Et_3N (32.7 ml, 246 mmol) and DMAP (1.5 g, 12.3 mmol) were added, and the mixture was cooled on ice. Di-tert-butyl dicarbonate (54 g, 246 mmol) was added and the reaction mixture was allowed to stir at room temperature for 4 h. After full conversion, the reaction mixture was concentrated to dryness. The crude product was dissolved in THF (150 ml), TBAF (1.0 M in THF; 164 ml, 164 mmol) was added and the mixture was stirred overnight. Upon full conversion, the reaction mixture was diluted with saturated aqueous $NaHCO_3$ and brine, extracted with EtOAc, dried over $MgSO_4$, filtered, and concentrated under reduced pressure. The residue was purified by silica gel column chromatography (R_f = 0.3, pentane/EtOAc 2:1 v/v) provided compound **18** as a white solid (19.2 g, 35.2 mmol, 86% over three steps). **^{1}H NMR** (400 MHz, $CDCl_3$) δ 7.64 (s, 1H, H-6), 5.93 (d, *J* = 5.7 Hz, 1H, H-1'), 5.78 (d, *J* = 8.2 Hz, 1H, H-5), 5.40 (t, *J* = 5.5 Hz, 1H, H-2'), 5.33 (dd, *J* = 5.4, 3.7 Hz, 1H, H-3'), 4.28 (m, 1H, H-4'), 3.97 (dd, *J* = 12.2, 2.2 Hz, 1H, H-5'), 3.83 (dd, *J* = 12.1, 2.2 Hz, 1H, H-5'), 1.59 (s, 9H, C(CH_3)$_3$), 1.49 (s, 9H, C(CH_3)$_3$), 1.47 (s, 9H, C(CH_3)$_3$). **^{13}C NMR** (101 MHz, $CDCl_3$) δ 160.4 (C=O uracil), 152.5 (C=O carbamate), 152.1 (C=O uracil), 148.6, 147.6 (C=O carbonate), 140.6 (C-6), 102.9 (C-5), 89.2 (C-1'), 87.2, 83.8, 83.6 (*C*(CH_3)$_3$), 82.9 (C-4'), 75.0 (C-2'), 73.0 (C-3'), 61.8 (C-5'), 27.8, 27.7, 27.6 (C(*C*H_3)$_3$). **HRMS** (ESI) m/z: $[M+Na]^+$ Calcd for $C_{24}H_{36}N_2O_{12}Na$ 567.2160; Found 567.2160.

CH$_2$ UDP-carbagalactose 19

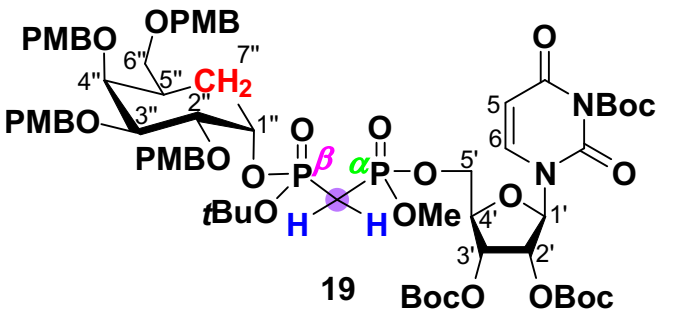

Compound **17** (130 mg, 0.14 mmol) was dissolved in MeCN: Et_3N: PhSH (3:3:2, 1 ml) and stirred at 35 °C for 4 h. Reaction progression was monitored by LC-MS, which indicated complete consumption of the starting material. The solution was concentrated *in vacuo*. The residue was purified by flash silica gel column chromatography (100% DCM to 100% Methanol), the product and compound **18** were co-evaporated with toluene, then dissolved in dry MeCN (1 mL), DIPEA (238 µL, 1.4 mmol) and PyNTP (210 mg, 0.42 mmol) were added, and the mixture was stirred at room temperature. After 2 h, the reaction was complete as indicated by TLC. The reaction mixture was poured into DCM and washed with water and brine. The organic layer was dried over Na_2SO_4, filtered and concentrated *in vacuo*. The crude product was purified by flash silica gel chromatography (R_f = 0.3, acetone/DCM 1:9) and then by size exclusion chromatography (LH-20) (DCM/MeOH 1:1) to afford **19** as a white amorphous solid (89 mg, 63 µmol, 44% over 2 steps). *A mixture of diastereoisomers.* **^{1}H NMR** (400 MHz, $CDCl_3$) δ 7.79 – 7.72 (m, 1H, H-6), 7.35 – 7.27 (m, 4H, CH_{arom}), 7.25 – 7.11 (m, 4H, CH_{arom}), 6.91 – 6.78 (m, 8H, CH_{arom}), 6.15 – 6.07 (m, 1H, H-1'), 5.86 – 5.70 (m, 1H, H-5), 5.35 – 5.16 (m, 2H, H-2', H-3'), 5.12 – 4.82 (m, 2H, CH*H* PMB, H-1''), 4.76 – 4.58 (m, 4H, C*H*H PMB, CH*H* PMB, C*H*H PMB, H-5), 4.43 – 4.18 (m, 5H, C*H*H PMB, C*H*H PMB, CH*H* PMB, H-4', H-5'), 4.15 – 3.92 (m, 2H, H-3'', H-4''), 3.89 – 3.84 (m, 1H, H-2''), 3.83 – 3.66 (m, 15H, OMe, OMe, OMe, OMe, P(O)OMe), 3.58 (d, J = 11.3 Hz, 1H, H-6''), 3.48 – 3.39 (m, 1H, H-6''), 2.75 – 2.38 (m, 2H, P-C*H*H-P, P-CH*H*-P), 2.24 – 2.13 (m, 1H, H-5''), 1.68 – 1.61 (m, 2H, H-7''), 1.63 – 1.56 (m, 9H, $C(CH_3)_3$), 1.52 – 1.40 (m, 27H, $C(CH_3)_3$). **^{13}C NMR** (101 MHz, $CDCl_3$) δ 160.4, 160.4, 160.3 (C=O uracil), 159.3, 159.3, 159.1, 159.1, 159.0 ($C_{q\text{-}arom}$), 152.2, 152.1, 152.0 (C=O carbamate), 152.0 (C=O uracil), 148.7, 148.6 (C=O carbonate), 147.7, 147.7, 147.6 (C=O carbonate), 139.9, 139.6 (C-6), 131.7, 131.6, 131.6, 131.3, 131.3, 131.2, 130.9, 130.5, 130.5 ($C_{q\text{-}arom}$), 130.0, 130.0, 129.8, 129.6, 129.4, 129.4, 129.1, 129.0, 129.0, 113.9, 113.8, 113.8, 113.8, 113.7, 113.6, 113.6 (CH_{arom}), 103.1, 103.0, 102.9 (C-5), 87.0, 86.9 ($C(CH_3)_3$), 86.8, 86.8, 86.7, 86.6, 86.4 (C-1'), 85.1, 84.6, 84.5, 84.4, 84.3, 83.6, 83.6, 83.6, 83.5, 83.5, 83.4 ($C(CH_3)_3$), 80.6, 80.4, 80.3, 80.2 (C-3'', C-4'), 78.5 (C-2''), 75.5, 75.4, 75.2, 75.1 (C-4''), 74.8, 74.7, 74.6 (C-1''), 74.5 (C-2'), 74.4, 73.1, 73.0, 72.9, 72.8, 72.7, 72.6, 72.5, 72.4 (CH_2 PMB), 72.3, 72.1, 72.0 (C-3'), 70.4, 70.2 (C-6''), 64.8, 64.7, 64.4 (C-5'), 55.42, 55.38, 53.35 (OMe PMB), 53.3, 53.22, 53.20, 52.87, 52.81 (P-CH_2-P), 35.8, 35.6 (C-5''), 30.54, 30.50, 30.5, 30.4 ($C(CH_3)_3$), 28.8, 28.5 (C-7''), 27.74, 27.70, 27.5 ($C(CH_3)_3$). **^{31}P NMR** (162 MHz, $CDCl_3$) *mixture of four diastereomers* δ 23.61 (d, J = 11.0 Hz, α-P), 23.32 (d, $J_{p\text{-}p}$ = 9.0 Hz, α-P), 23.24 (d, $J_{p\text{-}p}$ = 8.1 Hz, α-P), 22.76 (d, $J_{p\text{-}p}$ = 8.9 Hz, α-P), 15.56 (d, $J_{p\text{-}p}$ = 8.9 Hz, β-P), 15.43 (d, $J_{p\text{-}p}$ = 11.2 Hz, β-P), 12.41 – 12.23 (m, β-P). **HRMS** (ESI) m/z: $[M+Na]^+$ Calcd for $C_{70}H_{96}N_2O_{25}P_2Na$ 1435.5513; Found 1435.5515.

CH$_2$ UDP-carbagalactose analogue 5

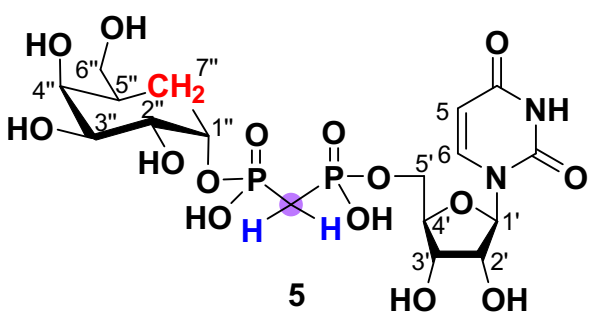

Compound **19** (60 mg, 0.04 mmol) was dissolved in 30% TFA in DCM (0.5 ml), followed by the addition of TES (0.24 mL, 1.5 mmol, 10.0 eq.). The reaction was stirred overnight at 0 °C, after which LC-MS analysis indicated the reaction to be complete. The reaction was neutralized by the addition of 30% aqueous ammonia. The mixture was concentrated and the residue was purified by size-exclusion chromatography (HW-40, 0.15 M aqueous NH_4OAc) followed by lyophilization to yield UDP-carbagalactose analogue **5** as a white powder (9.8 mg, 17.5 µmol, 41% over 2 steps). **^{1}H NMR** (400 MHz, D_2O) δ 7.96 (d, J = 8.1 Hz, 1H, H-6), 5.95 – 5.89 (m, 2H, H-7, H-1'), 4.60 – 4.51 (m, 1H, H-1''), 4.37 – 4.29 (m, 2H, H-2', H-3'), 4.22 (m, 1H, H-4'), 4.18 – 4.02 (m, 3H, H-4'', H-5'), 3.77 (dd, J = 10.2, 3.1 Hz, 1H, H-3''), 3.64 – 3.54 (m, 2H, H-2'', H-6''), 3.48 (dd, J = 11.0, 6.4 Hz, 1H, H-6''), 2.24 – 2.07 (m, 3H, H-5'', P-CH_2-P), 1.84 – 1.76 (m, 1H, H-7''), 1.57 – 1.48 (m, 1H, H-7''). **^{13}C NMR** (101 MHz, D_2O) δ 166.3 (C=O uracil), 151.8 (C=O uracil), 141.8 (C-6), 102.5 (C-5), 88.6 (C-1'), 83.3 (d, $J_{C\text{-}P}$ = 8.0 Hz, C-4'), 74.0 (d, $J_{C\text{-}P}$ = 6.5 Hz, C-1''), 73.8 (C-2'), 71.7 (C-3''), 70.9, 70.9 (d, J = 6.1 Hz, C-2''), 69.9 (C-4''), 69.5 (C-3'), 63.3 (d, $J_{C\text{-}P}$ = 5.3 Hz, C-5'), 62.4 (C-6''), 36.3 (C-5''), 28.0 (C-7''), 26.5 (dd, $J_{C\text{-}P}$ =129.4, 129.3 Hz, P-CH_2-P) . **^{31}P NMR** (162 MHz, D_2O): δ 18.09 (d, $J_{P\text{-}P}$= 11.2 Hz, α-P), 16.38 (d, $J_{P\text{-}P}$ = 11.2 Hz, β-P). **HRMS** (ESI) m/z: $[M+Na]^+$ Calcd for $C_{17}H_{29}N_2O_{15}P_2$ 563.1038; Found 563.1033.

Methylene bisphosphonate 22

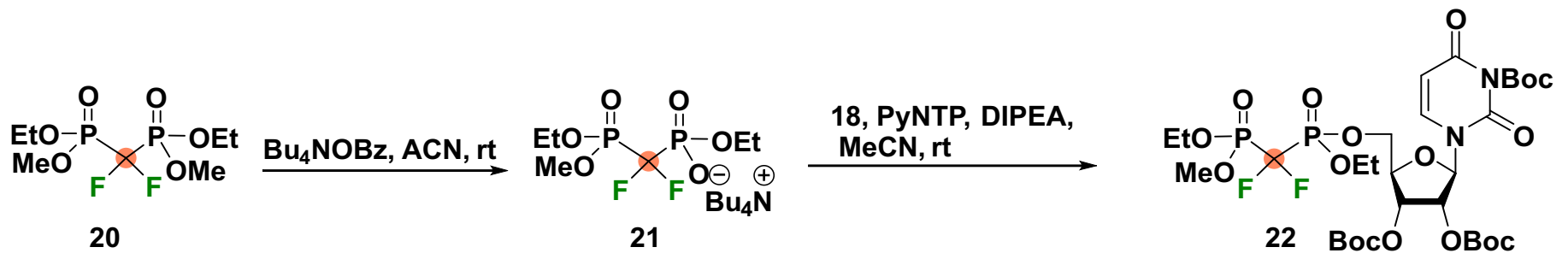

Diethyl(dimethyl) difluoromethylene bisphosphonate **20** (546 mg, 1.8 mmol) was co-evaporated with toluene and dissolved in dry ACN (9.0 mL), and tetrabutylammonium benzoate (670 mg, 1.8 mmol) was added under nitrogen.

The resulting mixture was stirred overnight. Compound **18** (2.9 g, 4.16 mmol), dried prior to use by evaporation with toluene, DIPEA (3.0 mL, 18 mmol), and PyNTP (2.7 g, 5.4 mmol) were added consecutively. After 2 h, the reaction was complete as indicated by TLC analysis. The reaction mixture was poured into DCM and washed with water and brine. The organic layer was dried over Na_2SO_4, filtered and concentrated under reduced pressure. Purification by silica gel column chromatography (R_f = 0.2, pentane/EtOAc 1:2) and subsequent size exclusion chromatography (LH-20) (DCM/MeOH 1:1) yielded **22** as a white solid (970 mg, 1.2 mmol, 65%). *A mixture of diastereoisomers.* **^{1}H NMR** (400 MHz, $CDCl_3$) δ 7.72 – 7.55 (m, 1H, H-6), 6.17 – 6.07 (m, 1H, H-5), 5.85 – 5.73 (m, 1H, H-1'), 5.39 – 5.14 (m, 2H, H-3', H-2'), 4.60 – 4.45 (m, 2H, H-5'), 4.46 – 4.29 (m, 5H, H-4', CH_2 Et), 4.02 – 3.92 (m, 3H, P(O)OMe), 1.60 (s, 3H, CH_3 Et), 1.51 – 1.42 (m, 18H, $C(CH_3)_3$), 1.45 – 1.36 (m, 6H, CH_3 Et). **^{13}C NMR** (101 MHz, $CDCl_3$) δ 160.4 (C=O uracil), 152.2, 152.1 (C=O carbamate), 151.9 (C=O uracil), 148.6, 148.5 (C=O carbonate), 147.6, 147.6 (C=O carbonate), 139.4, 139.3 (C-6), 103.0, 102.9 (C-5), 87.3, 87.3 (C-1'), 87.1 ($C(CH_3)_3$), 86.7 (C-1'), 83.8, 83.7, 83.7 ($C(CH_3)_3$, $C(CH_3)_3$, $C(CH_3)_3$), 80.1, 80.0, 79.9 (C-4'), 74.8, 74.8 (C-2'), 71.8 (C-3'), 66.8, 66.6, 66.6, 66.4, 66.3, 66.1 (C-5', CH_2 Et, CH_2 Et), 55.7, 55.6 (P(O)OMe, P(O)OMe), 27.7, 27.7, 27.6 ($C(CH_3)_3$, $C(CH_3)_3$, $C(CH_3)_3$), 16.6, 16.5 (CH_3 Et, CH_3 Et). **^{31}P NMR** (162 MHz, $CDCl_3$): δ 5.61 – 3.11 (m). **^{19}F NMR** (376 MHz, $CDCl_3$): δ -120.34 – -123.42 (m). **HRMS** (ESI) m/z: $[M+Na]^+$ Calcd for $C_{30}H_{48}F_2N_2O_{17}P_2Na$ 831.2288; Found 831.2288.

Compound 25

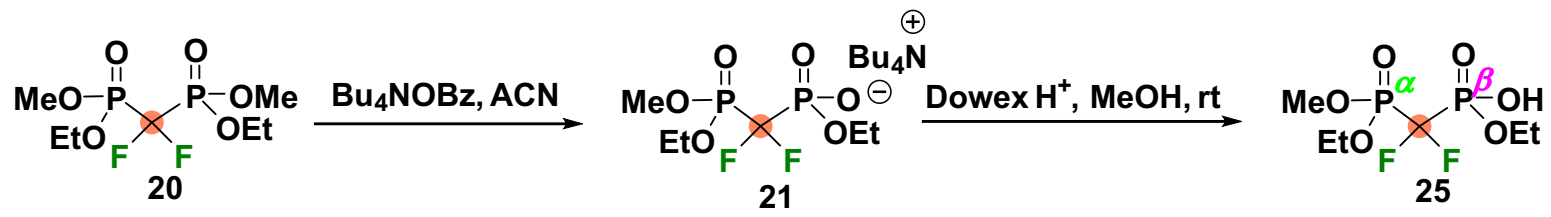

20 21 25

Diethyl(dimethyl) difluoromethylene bisphosphonate **20** (5.0 g, 16.9 mmol) was co-evaporated with toluene and dissolved in dry ACN (56 mL), and tetrabutylammonium benzoate (6.1 g, 16.9 mmol) was added under nitrogen and the resulting mixture was stirred overnight. Upon full conversion to compound **21**, the reaction was concentrated. The residue was dissolved in methanol (56 ml), Dowex 50W-X8 (H^+) resin (6.0 g) was added, the resulting mixture was stirred at rt for 4 h. The solution was filtered and removal of the solvent under reduced pressure gave the compound **25** as a light-yellow oil (3.4 g, 12 mmol, 71%). **^{1}H NMR** (300 MHz, $CDCl_3$) δ 8.85 (s, 1H), 4.44 – 4.22 (m, 4H), 3.95 (d, J = 12.0 Hz, 3H), 1.37 (q, J = 6.0 Hz, 6H). **^{13}C NMR** (75 MHz, $CDCl_3$) δ 66.1, 66.0, 65.4, 65.3, 55.6, 55.5, 16.5. **^{31}P NMR** (121 MHz, $CDCl_3$): δ 3.47 (ABX, $J_{P\text{-}F}$ = 88.3 Hz, $J_{P\text{-}P}$= 71.4 Hz, α-P), 2.77 (ABX, $J_{P\text{-}F}$ = 83.5 Hz, $J_{P\text{-}P}$= 70.2 Hz, β-P). **^{19}F NMR** (376 MHz, $CDCl_3$): δ -122.37 (t, $J_{P\text{-}F}$ = 86.5 Hz). **HRMS** (ESI) m/z: $[M+Na]^+$ Calcd for $C_{17}H_{29}N_2O_{15}P_2$ 563.1038; Found 563.1033.

Table SI-3 Explored Mitsunobu reaction conditions

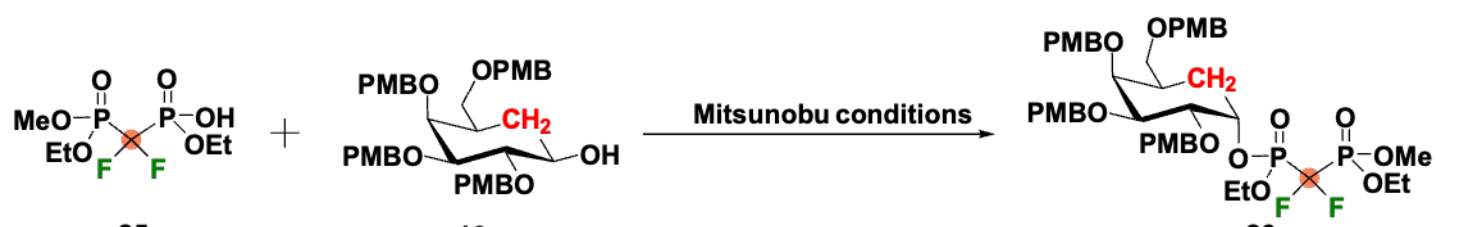

25 10 26

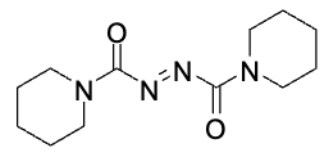

1,1'-(Azodicarbonyl)dipiperidine(ADDP)

Entry	Alcohol (eq.)	Acid (eq.)	Phosphine	Azodicarboxylate	Additive	Solvent	Temperature	Yield (%)
				Alcohol-limiting conditions[a]				
1-3	1.0	2.0	(p-ClPh)$_3$P (2.0), Ph_3P (2.0), Bu_3P (2.0)	DIAD (2.0)	TEA (5.0)	THF	rt	trace
4	1.0	2.0	(p-ClPh)$_3$P (3.0)	DIAD (3.0)	TEA (5.0)	THF	rt	trace
5	1.0	2.0	(p-ClPh)$_3$P (2.0)	DIAD (2.0)	TEA (10.0)	THF	rt	trace
6	1.0	4.0	(p-ClPh)$_3$P (2.0)	DIAD (2.0)	TEA (5.0)	THF	rt	trace
7	1.0	2.0	(p-ClPh)$_3$P (2.0)	DIAD (2.0)	TEA (5.0)	THF	reflux	
8	1.0	2.0	(p-ClPh)$_3$P (2.0)	ADDP (2.0)	TEA (5.0)	THF	rt	trace
				Acid-limiting conditions[b]				
9	2.0	1.0	P(p-ClPh)$_3$ (2.0)	DIAD (2.0)	-	THF	rt	NR
10[c]	2.0	1.0	P(p-ClPh)$_3$ (2.0)	DIAD (2.0)	-	THF	rt	NR
11	2.0	1.0	P(p-ClPh)$_3$ (2.0)	DIAD (2.0)	-	toluene	rt	NR
12	3.0	1.0	P(p-ClPh)$_3$ (2.0)	DIAD (2.0)	-	THF	rt	NR
13	2.0	1.0	PPh_3 (2.0)	DIAD (2.0)	-	THF	rt	trace
14	3.0	1.0	PPh_3 (2.0)	DIAD (2.0)	-	THF	rt	<10
15	2.0	1.0	PPh_3 (4.0)	DIAD (4.0)	-	THF	rt	<10
16	2.0	1.0	PPh_3 (2.0)	DIAD (2.0)	TEA (5.0)	THF	rt	NR
17	2.0	1.0	PPh_3 (2.0)	ADDP (2.0)	-	THF	rt	NR

a. General Procedure for alcohol-limiting conditions: the alcohol was added into a pre-mixed solution of the acid, azodicarboxylate reagent, and P^{III} component.

b. General Procedure for acid-limiting conditions: azodicarboxylate reagent was added to a mixture of the acid, alcohol, and P^{III} component. c. Following the procedure for alcohol-limiting conditions.

Compound 26

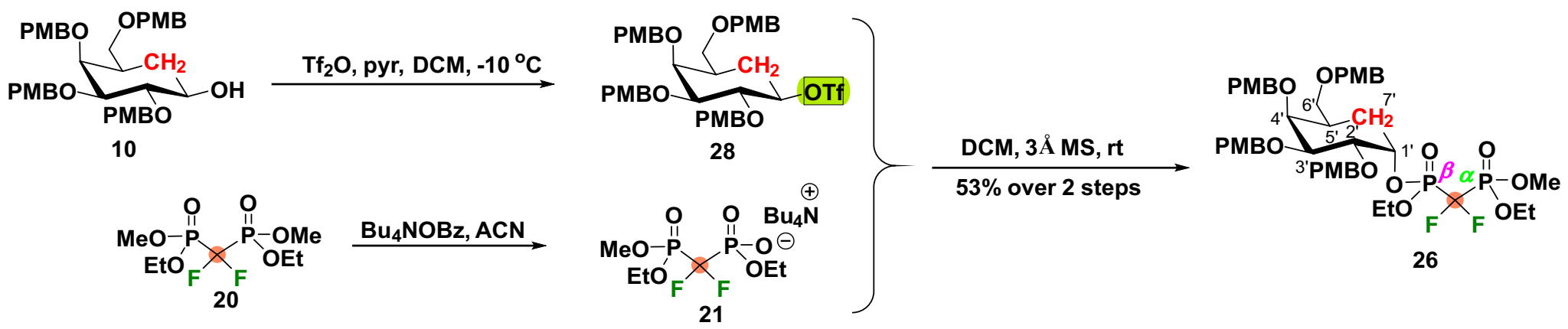

Compound **10** (45 mg, 0.15 mmol) was co-evaporated three times with toluene and dissolved in DCM (1 mL). Pyridine (237 µl, 3.0 mmol) was added and the reaction mixture was cooled to -10 °C. Tf_2O (51 µl, 0.3 mmol) was added dropwise and the reaction was stirred for 4 h. After the starting material was fully converted, the reaction was quenched with sat. aq. $NaHCO_3$ and extracted with DCM. The combined organic layers were washed with sat. aq. $CuSO_4$ three times and brine, dried over anhydrous Na_2SO_4, filtered and concentrated below 20 °C. The resulting crude residue **28** was evaporated 3 times with toluene below 20 °C and used directly for the next step.

Diethyl (dimethyl) difluoromethylene bisphosphonate **20** (100 mg, 0.15 mmol) was co-evaporated with toluene and dissolved in dry ACN (1.0 ml), tetrabutylammonium benzoate (55 mg, 0.15 mmol) was added under nitrogen and the resulting mixture was stirred overnight. After completion of the reaction, as indicated by TLC, the reaction mixture was concentrated and the residue dissolved in dry DCM (1 ml), after which 3 Å MS (150 mg) were added. The crude triflate **28** was added to this mixture and stirred at room temperature for 4 h. After completion of the reaction, the reaction mixture was concentrated. Purification by silica gel column chromatography (R_f = 0.23 and 0.32, pentane/EtOAc 1:1) and subsequent size exclusion chromatography (LH-20) (DCM/MeOH 1:1) yielded **27** as a colorless oil (74 mg, 80 µmol, 53% over 2 steps). *A mixture of diastereoisomers.* **^{1}H NMR** (500 MHz, $CDCl_3$) δ 7.35 – 7.12 (m, 8H, CH_{arom}), 6.88 – 6.79 (m, 8H, CH_{arom}), 5.09 – 5.04 (m, 1H, C-1'), 4.84 (d, *J* = 10.8 Hz, 1H, C*H*H PMB), 4.76 – 4.57 (m, 4H, C*H*H PMB, CH*H* PMB, C*H*H PMB, CH*H* PMB), 4.41 – 4.35 (m, 3H, CH*H* PMB, CH*H* PMB, C*H*H PMB), 4.35 – 4.20 (m, 4H, CH_2 Et, CH_2 Et), 4.07 (d, *J* = 2.7 Hz, 1H, H-4'), 3.92 – 3.84 (m, 4H, H-2', P(O)OMe), 3.82 (d, *J* = 2.4 Hz, 1H, H-3), 3.81 – 3.77 (m, 12H, OMe, OMe, OMe, OMe), 3.44 (t, *J* = 9.0 Hz, 1H, H-6'), 3.26 (dd, *J* = 9.0, 5.6 Hz, 1H, H-6'), 2.29 – 2.19 (m, 1H, H-5'), 1.85 – 1.79 (m, 1H, H-7'), 1.70 – 1.59 (m, 1H, H-7'), 1.40 – 1.28 (m, 6H, CH_3 Et, CH_3 Et). **^{13}C NMR** (126 MHz, $CDCl_3$) δ 159.3, 159.1, 159.0, 131.6, 131.4, 130.9, 130.5 ($C_{q\text{-}arom}$), 129.5, 129.5, 129.4, 129.1, 113.9, 113.7, 113.6, 113.6 (CH_{arom}), 80.4 (C-3'), 77.39 (C-1'), 77.36 (C-2'), 75.5 (C4'), 74.3 (CH_2 PMB), 73.01, 72.99, 72.8, 72.1 (CH_2 PMB), 70.1 (C-6'), 65.7, 65.6, 65.6 (CH_2 Et), 55.4, 55.3, 55.3, 55.3, 55.2 (OMe), 35.4 (C-5'), 28.5 (C-7'), 16.5 (d, $J_{C\text{-}P}$= 5.4 Hz, CH_3 Et), 16.4 (d, $J_{C\text{-}P}$ = 5.8 Hz, CH_3 Et). **^{31}P NMR** (202 MHz, $CDCl_3$) δ 5.02 (2 x ABX_2, $J_{P\text{-}F}$ = 87.2, $J_{P\text{-}P}$ = 70.9 Hz, *β*-P), 2.24 (2 x ABX_2, $J_{P\text{-}F}$ = 86.9, 70.9 Hz, *α*-P). **^{19}F NMR** (471 MHz, $CDCl_3$) δ -119.42 – -122.05 (m). **HRMS** (ESI) m/z: $[M+Na]^+$ Calcd for $C_{30}H_{48}F_2N_2O_{17}P_2Na$ 831.2288; Found 831.2288.

Compound 27

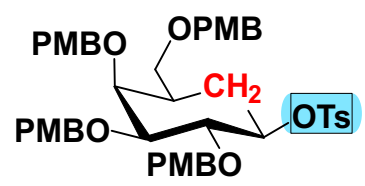

27

Compound **10** (146 mg, 0.22 mmol) was co-evaporated with toluene and dissolved in dry pyridine (2.0 ml), whereupon TsCl (126 mg, 0.66 mmol) was added under nitrogen, and the resulting mixture was stirred overnight. Upon completion of the reaction, as indicated by TLC analysis. The reaction mixture was poured into sat. aq. $NaHCO_3$ and extracted with EtOAc. The combined organic layers were washed with brine, dried over $MgSO_4$, filtered and concentrated under reduced pressure. The crude product was purified by flash column chromatography over silica gel (R_f = 0.2, pentane/EtOAc 3:1) to obtain **27** as a white amorphous solid (121 mg, 0.15 mmol, 67%). **^{1}H NMR** (500 MHz, $CDCl_3$) δ 7.74 (d, *J* = 8.2 Hz, 2H, CH_{arom}), 7.24 – 7.13 (m, 8H, CH_{arom}), 7.09 – 7.03 (m, 2H, CH_{arom}), 6.90 – 6.80 (m, 6H, CH_{arom}), 6.79 – 6.74 (m, 2H, CH_{arom}), 4.86 (d, *J* = 10.9 Hz, 1H, C*H*H PMB), 4.65 – 4.54 (m, 3H, CH*H* PMB, C*H*H PMB, CH*H* PMB), 4.54 – 4.46 (m, 2H, H-1, C*H*H PMB), 4.39 (d, *J* = 10.9 Hz, 1H, C*H*H PMB), 4.36 – 4.29 (m, 2H, C*H*H PMB, CH*H* PMB), 3.99 (d, *J* = 2.1 Hz, 1H, H-4), 3.89 (t, *J* = 9.4 Hz, 1H, H-2), 3.83 – 3.76 (m, 12H, OMe, OMe, OMe, OMe), 3.39 (t, *J* = 8.8 Hz, 1H, H-6), 3.31 (dd, *J* = 9.6, 2.4 Hz, 1H, H-3), 3.21 (dd, *J* = 8.9, 4.8 Hz, 1H, H-6'), 2.37 (s, 3H, Me Ts), 1.87 – 1.79 (m, 1H, H-7), 1.79 – 1.72 (m, 1H, H-7'), 1.72 – 1.65 (m, 1H, H-5). **^{13}C NMR** (126 MHz, $CDCl_3$) δ 159.3, 159.1, 159.0, 159.0, 144.3, 134.5, 131.3, 130.8, 130.6, 130.1 ($C_{q\text{-}arom}$), 113.9, 113.7, 113.6, 113.5 (CH_{arom}), 84.1 (C-3), 82.9 (C-1), 80.1 (C-2), 75.1, 74.2 (CH_2 PMB), 73.7 (C-4), 72.9, 72.7 (CH_2 PMB), 69.7 (C-6), 55.3 (OMe), 37.2 (C-5), 28.6 (C-7), 21.6 (Me Ts). **HRMS** (ESI) m/z: $[M+Na]^+$ Calcd for $C_{46}H_{52}O_{11}SNa$ 835.3123; Found 835.3123.

Protected CF_2 UDP-carbagalactose 24

Method 1:

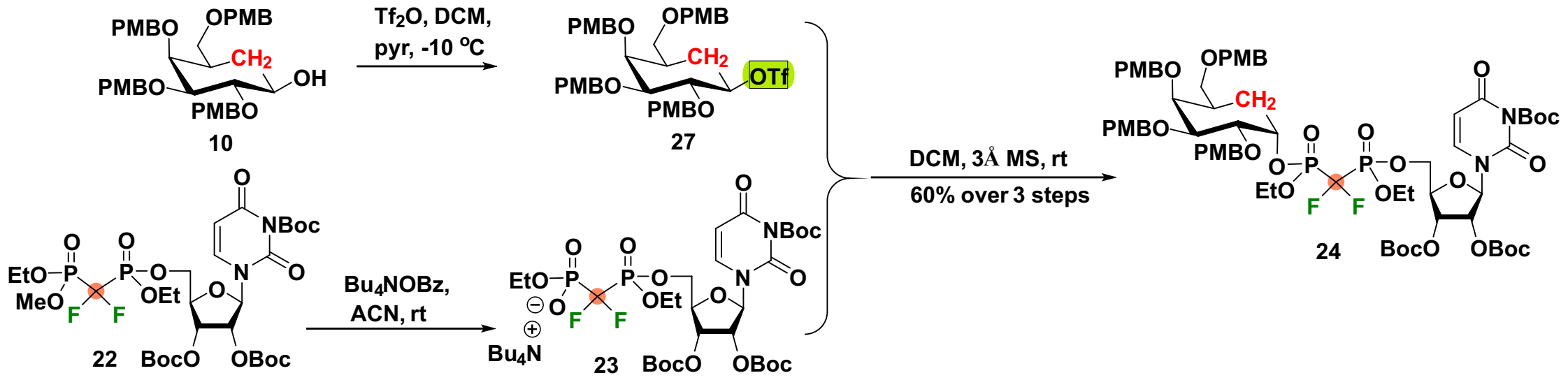

Compound **10** (79 mg, 0.12 mmol) was co-evaporated 3 times with toluene and dissolved in DCM (1 mL). Pyridine (191 µl, 2.4 mmol) was added and the reaction mixture was cooled to -10 °C. Tf_2O (41 µl, 0.24 mmol) was added dropwise and the reaction was stirred for 4 h. After the starting material was fully converted, the reaction was quenched with sat. aq. $NaHCO_3$ and extracted with DCM. The combined organic layers were washed with sat. aq. $CuSO_4$ three times and brine, dried over anhydrous sodium sulfate, filtered and concentrated below 20 °C. The resulting crude residue **28** was evaporated three times with toluene below 20 °C and used directly for the next step.

Compound **22** (96.5 mg, 0.12 mmol) was co-evaporated with toluene dissolved in dry ACN (1.0 mL) and treated with tetrabutylammonium benzoate (44 mg, 0.12 mmol) under a nitrogen atmosphere. After completion of the reaction as indicated by TLC, the reaction mixture was concentrated and redissolved in dry DCM (1 mL) and 3 Å MS (180 mg) were added. The crude triflate **28** (0.12 mmol) was added and the reaction mixture was stirred for 4 h. After completion of the reaction as indicated by TLC, the reaction mixture was concentrated. The crude product was purified by flash column chromatography over silica gel (R_f = 0.22 and 0.36, pentane/EtOAc 2:1) and then by size exclusion chromatography (LH-20) (DCM/MeOH 1:1) to afford **24** as a colorless oil (102 mg, 71 µmol, 60%).

Method 2:

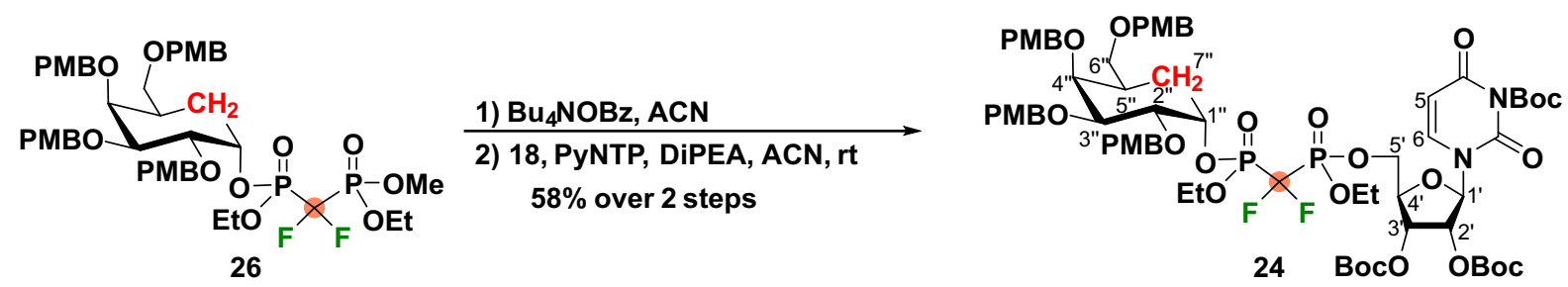

Compound **26** (200 mg, 0.22 mmol) was co-evaporated with toluene and dissolved in dry ACN (2.0 ml), tetrabutylammonium benzoate (80 mg, 0.22 mmol) was added under nitrogen and the resulting mixture was stirred overnight. Upon full conversion, compound **18** (359 mg, 0.66 mmol), dried prior to use by evaporation with toluene, DIPEA (384 µL, 2.2 mmol) and PyNTP (439 mg, 0.88 mmol) were added. The mixture was stirred at room temperature for 2 h. After the starting material was fully converted as indicated by TLC-analysis, the reaction mixture was poured into DCM and washed with water and brine. The organic layer was dried over Na_2SO_4, filtered and evaporated *in vacuo*. The crude product was purified by flash column chromatography over silica gel (R_f = 0.22 and 0.26, pentane/EtOAc 1:2) and followed by size exclusion chromatography (LH-20) (DCM/MeOH 1:1) to obtain **24** as a colorless oil (182 mg, 1.3 mmol, 58% over 2 steps). *A mixture of diastereoisomers.* **^{1}H NMR** (400 MHz, $CDCl_3$) δ 7.71 and 7.58 (2 x d, *J*= 5.8 Hz, 1H, H-6), 7.39 – 7.33 (m, 2H, CH_{arom}), 7.31 – 7.27 (m, 2H, CH_{arom}), 7.25 – 7.21 (m, 2H, CH_{arom}), 7.18 – 7.12 (m, 2H, CH_{arom}), 6.89 – 6.78 (m, 8H, CH_{arom}), 6.15 and 6.14 (2 x d, *J* = 7.8 Hz, 1H, H-1'), 5.81 and 5.76 (2 x d, *J* = 8.2 Hz, 1H, H-5), 5.44 – 5.25 (m, 1H, H-3'), 5.23 – 5.13 (m, 2H, H-2, H-1''), 4.91 – 4.83 (m, 1H, C*H*H PMB), 4.79 and 4.77 (2 x d, *J* = 10.6, 1H, CH*H* PMB), 4.71 – 4.61 (m, 2H, C*H*H PMB, CH*H* PMB), 4.58 – 4.45 (m, 3H, C*H*H PMB, CH*H* PMB, C*H*H PMB), 4.44 – 4.29 (m, 6H, CH_2 Et, CH_2 Et, C*H*H PMB, H-4'), 4.16 – 3.99 (m, 3H, H-5', H-4''), 3.93 – 3.85 (m, 1H, H-2''), 3.83 – 3.75 (m, 12H, OMe, OMe, OMe, OMe), 3.67 – 3.62 (m, 1H, H-3''), 3.45 (m, 1H, H-6''), 3.32 – 3.23 (m, 1H, H-6''), 2.25 – 2.14 (m, 1H, H-5''), 1.88 – 1.69 (m, 2H, H-7''), 1.68 – 1.56 (m, 9H, $C(CH_3)_3$), 1.50 – 1.46 (m, 9H, $C(CH_3)_3$), 1.46 – 1.41 (m, 9H, $C(CH_3)_3$), 1.40 – 1.35 (m, 3H, CH_3 Et), 1.16 – 1.08 (m, 3H, CH_3 Et). **^{13}C NMR** (101 MHz, $CDCl_3$) δ 160.3, 160.2 (C=O uracil), 159.3, 159.2, 159.2, 159.1, 159.0 ($C_{q\text{-}arom}$), 152.1, 152.0 (C=O carbamate), 151.9 (C=O uracil), 148.7, 148.6 (C=O carbonate), 147.7, 147.6 (C=O carbonate), 139.3, 139.2 (C-6), 131.64, 131.60, 131.2, 131.1, 130.63, 130.58, 130.5, 130.4, 130.29, 130.25 ($C_{q\text{-}arom}$), 129.7, 129.5, 129.4, 129.1, 113.9, 113.8, 113.7, 113.65, 113.63, 113.61, 113.5 6 ($C_{q\text{-}arom}$), 103.0, 102.9 (C-5), 86.99, 86.97 ($C(CH_3)_3$), 86.91, 86.5 (C-1'), 83.71, 83.69, 83.63, 83.5 ($C(CH_3)_3$, $C(CH_3)_3$, $C(CH_3)_3$, $C(CH_3)_3$), 80.3, 80.2, 80.14, 80.09, (C-3'', C-4'), 77.9, 77.8 (C-2''), 77.3 (C-1''), 77.1, 77.0 (C-2'), 75.2, 75.1 (C-4''), 74.8 (C-4'), 74.52, 74.48 (CH_2 PMB), 72.91, 72.88, 72.58, 72.55, 72.47, 72.4 (CH_2 PMB), 72.2, 72.0 (C-3'), 70.1, 70.0 (C-6''), 66.8, 66.7, 66.5, 66.44, 66.39, 66.33, 66.26, 66.20 (CH_2 Et), 65.7, 65.67, 65.56, 65.49 (C-5'), 55.37 (OMe), 35.54, 35.50 (C-5''), 27.74, 27.69, 27.5 (C-7'', $C(CH_3)_3$), 16.6, 16.6, 16.5, 16.4, 16.2, 16.1 (CH_3 Et, CH_3 Et). **^{31}P NMR** (162 MHz, $CDCl_3$) δ 5.04 (m), 1.93

(m). **^{19}F NMR** (376 MHz, $CDCl_3$) δ -120.35 – -123.91 (m). **HRMS** (ESI) m/z: $[M+Na]^+$ Calcd for $C_{68}H_{90}F_2N_2O_{25}P_2Na$ 1457.5168; Found 1457.5168.

Compound 29

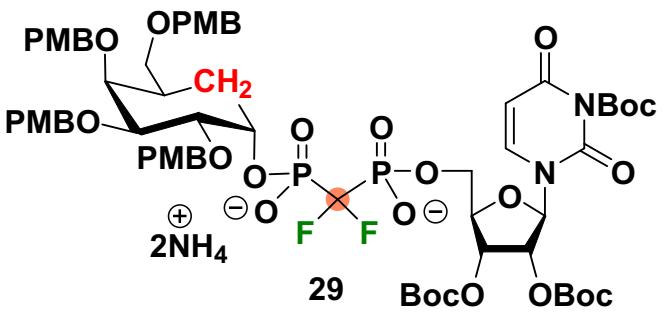

Compound **17** (52 mg, 36 μmol) was dissolved in MeCN: Et_3N: PhSH (3:3:2, 0.5 ml) and stirred at 35 °C. Reaction progression was monitored by LC-MS, which indicated complete consumption of the starting material after 6 h. The solution was concentrated *in vacuo* and the residue was purified by flash silica gel column chromatography (100% DCM to 100% methanol) to remove excess thiophenol. The crude product was then purified by HPLC to give compound **29** (26.4 mg, 19 μmol, 53%) as a white solid. Due to the amphiphilic nature of compound **29**, no interpretable ^{1}H or ^{13}C NMR spectra could be obtained. **^{31}P NMR** (162 MHz, $CDCl_3$) δ 3.34 – 0.25 (m). **^{19}F NMR** (376 MHz, $CDCl_3$) δ -119.14 (t, J_{P-F} = 76.9 Hz). **HRMS** (ESI) m/z: $[M+Na]^+$ Calcd for $C_{68}H_{90}F_2N_2O_{25}P_2Na$ 1401.4542; Found 1401.4545.

CF_2 UDP-carbagalactose analogue 6

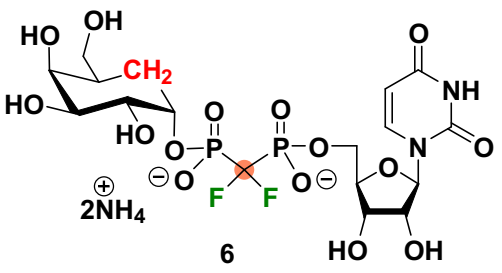

Compound **29** (26 mg, 19 μmol) was dissolved in 30% TFA in DCM (0.5 mL), followed by the addition of TES (0.24 mL, 1.5 mmol, 10.0 eq.). The reaction was stirred at 0 °C for 12 h. The reaction was complete as indicated by LC-MS analysis. The mixture was neutralized by the addition of 30% aqueous ammonia. The mixture was concentrated, and the residue was purified by size-exclusion chromatography (HW40-column, 0.15 M aqueous NH_4OAc in water) followed by lyophilization to yield UDP-carbagalactose analogue **6** (9.6 mg, 16 μmol, 84%) as a white powder. **^{1}H NMR** (400 MHz, D2O) δ 7.92 (d, *J* = 8.1 Hz, 1H, H-6), 5.96 – 5.86 (m, 2H, H-5, H-1'), 4.66 (m, 1H, H-1''), 4.36 – 4.17 (m, 5H, H-2', H-3', H-4', H-5'), 4.06 (m, 1H, H-4''), 3.79 – 3.71 (m, 1H, H-3''), 3.64 – 3.54 (m, 2H, H-2'', H-6''), 3.48 (dd, *J* = 11.0, 6.3 Hz, 1H, H-6''), 2.20 – 2.06 (m, 1H, H-5''), 1.85 – 1.78 (m, 1H, H-7''), 1.64 – 1.49 (m, 1H, H-7''). **^{13}C NMR** (101 MHz, D_2O) δ 166.4 (C=O uracil), 152.0 (C=O uracil), 141.6 (C-6), 102.6 (C-5), 88.3 (C-1'), 83.3 (d, J_{C-P} = 6.8 Hz, C-4'), 76.3 (d, J_{C-P} = 6.6 Hz, C-1''), 73.8 (C-2'), 71.6 (C-3''), 70.7 (d, J_{C-P} = 6.3 Hz, C-2''), 69.7 (C-4''), 69.6 (C-3'), 65.4 (d, J_{C-P} = 5.7 Hz, C-5'), 62.3 (C-6''), 36.2 (C-5''), 28.2 (C-7''). **^{31}P NMR** (162 MHz, D_2O) δ 4.76 – 2.30 (m). **^{19}F NMR** (471 MHz, D_2O) δ -118.25 – -120.87 (m). **HRMS** (ESI) m/z: $[M+Na]^+$ Calcd for $C_{17}H_{26}F_2N_2O_{15}P_2Na$ 621.0669; Found 621.0668.

2.2 Biochemical Characterization

***In vitro* rhA4GALT activity assay**

The *in vitro* half maximal inhibitory concentration (IC50) values in recombinant human (rhA4GALT) were determined using a previously developed HPLC-based activity assay.[4]

Briefly, 1.25 μL of 0.1 mM C12-NBD-LacCer in ethanol was dried under a stream of N2-gas at 45 °C. Subsequently, 2.5 μL of 10 mM UDP-Gal in MQ-H2O, 1 μL of 0.6 M MnCl2, 1 μL of 10% (w/v) Taurocholate, 2 μL of 2% (w/v) BSA, 4 μL of 500 mM MOPS buffer (pH 6.6), 0.5 μL of inhibitor solution in DMSO (at various concentrations, final 1% DMSO in the assay), 29 μL of MQ-H2O and 10 μL of rhA4GalT (100x diluted, 1.31 μM, in 50 mM MOPS pH 6.6 with 0.1% (w/v) BSA) were added. The final assay volume was 50 μL. The reaction mixtures were incubated for 1 h at 37 °C in a shaking water bath. Reactions were terminated either by immediate freezing in liquid N2 or by initiating lipid extraction though addition of 290 μL MeOH, 150 μL CHCl3 and 10 μL 10 μM C12-NBD-GlcCer in MeOH as internal standard, as previously described (REF as above).

A4GALT activity was calculated as the ratio of C12-NBD-Gb3 signal over the internal standard (Gb3:IS) and normalized against the ratio of the 100% activity control (no inhibitor). Assay and standard samples were separated on a reversed-phase BDS Hypersil 120A C18 column (3 μm, 150 x 4.6 mm) using a Thermo Fisher Ultimate 3000 Dionex UHPLC Standard Quaternary System with fluorescence detection (λex = 460 nm, λem = 540 nm, 280 FW). The injection volume was 25 μL and separation was achieved in 30 min by isocratic elution with 1 mL/min 70:30 ACN (HPLC-S gradient grade, Biosolve): MQ-water. Chromeleon version 7.2 SR4 software was used for peak analysis. If inhibition was observed, the normalized activity was plotted against the inhibitor concentration (Log[inhibitor] (M)) in GraphPad Prism version 9.0.0 (121). A curve was then fitted from the

average of the technical triplicate applying the non-linear regression function ‘log(inhibitor) *vs.* normalized response’ to determine the final IC_{50} value as shown in **Figure-SI-2**.

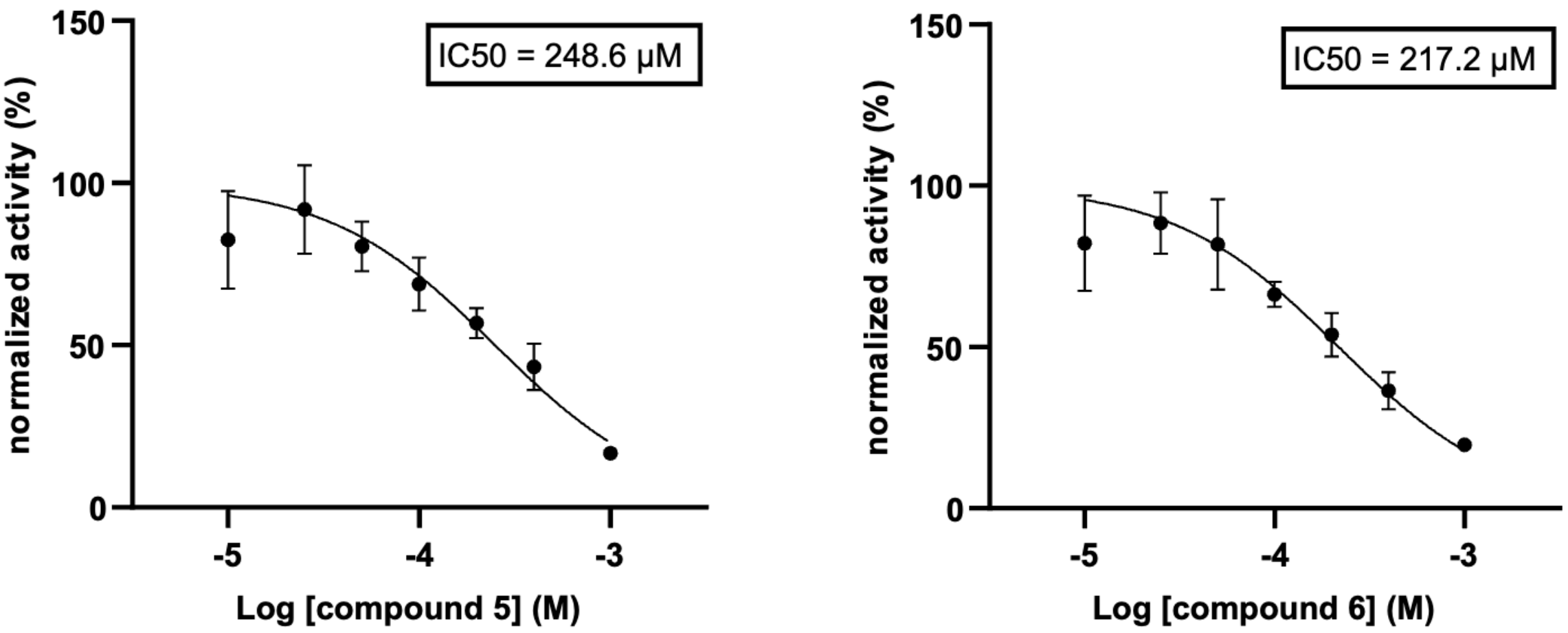

Figure-SI-2. Apparent IC_{50} curves of bisphosphonate compounds **5** and **6** and using C12-NBD-LacCer as substrate for HPLC-based A4GALT activity assays, n = 3.

3. References

(1) Ramana, C. V.; Khaladkar, T. P.; Chatterjee, S.; Gurjar, M. K. Total Synthesis and Determination of Relative and Absolute Configuration of Multiplolide A. *J. Org. Chem.* **2008**, *73*, 3817-3822.
(2) Ofman, T. P.; Heming, J. J. A.; Nin-Hill, A.; Küllmer, F.; Moran, E.; Bennett, M.; Steneker, R.; Klein, A.-M.; Ruijgrok, G.; Kok, K.; et al. Conformational and Electronic Variations in 1,2- and 1,5a-Cyclophellitols and their Impact on Retaining α-Glucosidase Inhibition. *Chem. Eur. J.* **2024**, *30*, e202400723.
(3) Evans, D. A.; Chapman, K. T.; Bisaha, J. Asymmetric Diels-Alder cycloaddition reactions with chiral .alpha.,.beta.-unsaturated N-acyloxazolidinones. *J. Am. Chem. Soc.* **1988**, *110*, 1238-1256.
(4) de Koster, N.; Vidal-Gironès, Ò.; de Graaf, R.; Kok, K.; Lelieveld, L. T.; Hoogervorst, J.; Ferraz, M. J.; Rivas-Fernández, J. P.; Lammers, R. F.; Overkleeft, H. S.; Aerts, J. M. F. G.; Boot, R. G.; Rovira, C.; Artola, M. Unraveling A4GALT mechanism and its modulation with adamantyl-galactosylceramide analogues: advancing Fabry disease therapeutic strategies. *Angew. Chem. Int. Ed.* **2026**, e9633257

4. NMR Spectra

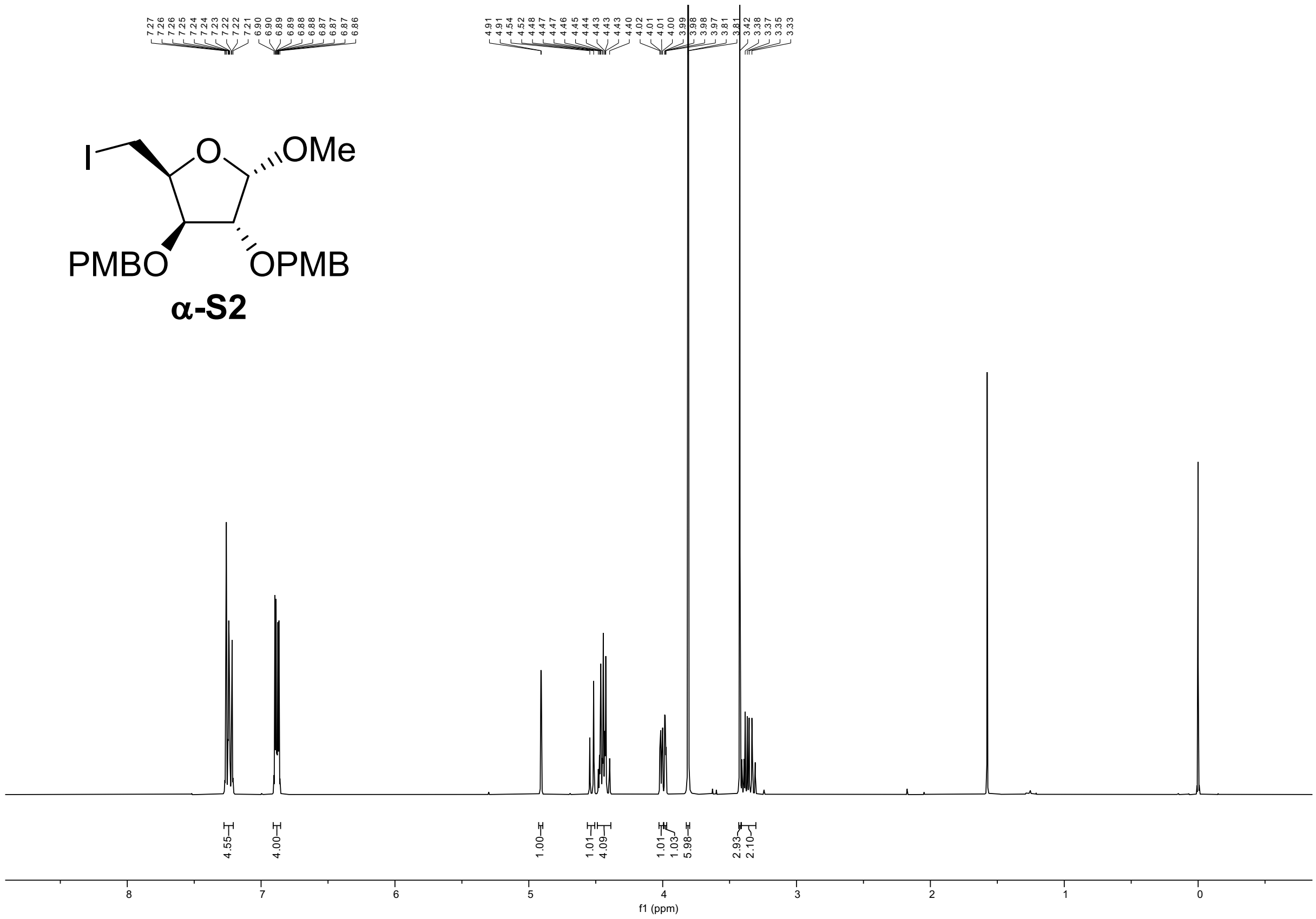

^{1}H NMR (400 MHz, $CDCl_3$) of compound **α-S2**

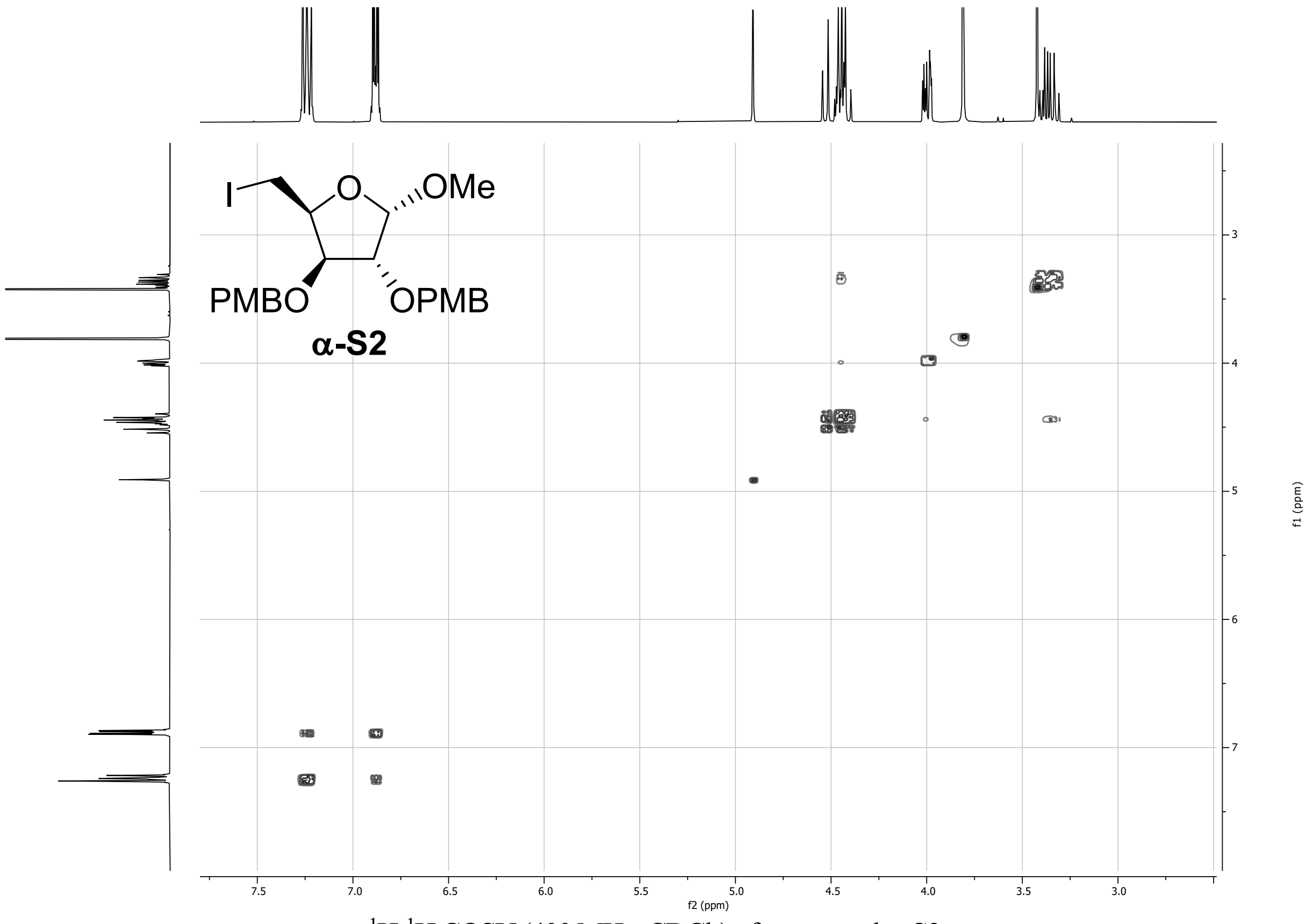

^{1}H-^{1}H COSY (400 MHz, $CDCl_3$) of compound **α-S2**

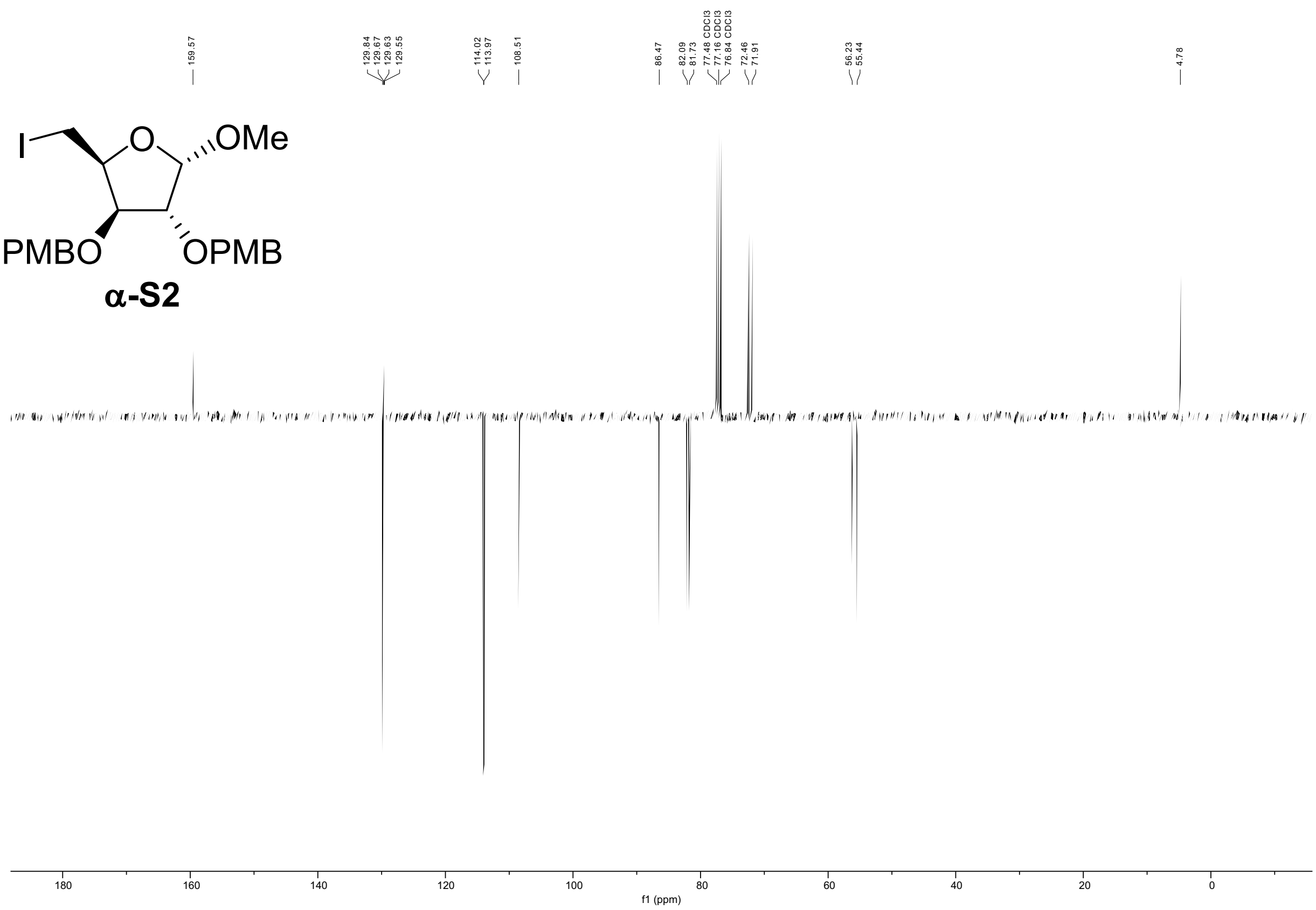

^{13}C NMR (101 MHz, $CDCl_3$) of compound **α-S2**

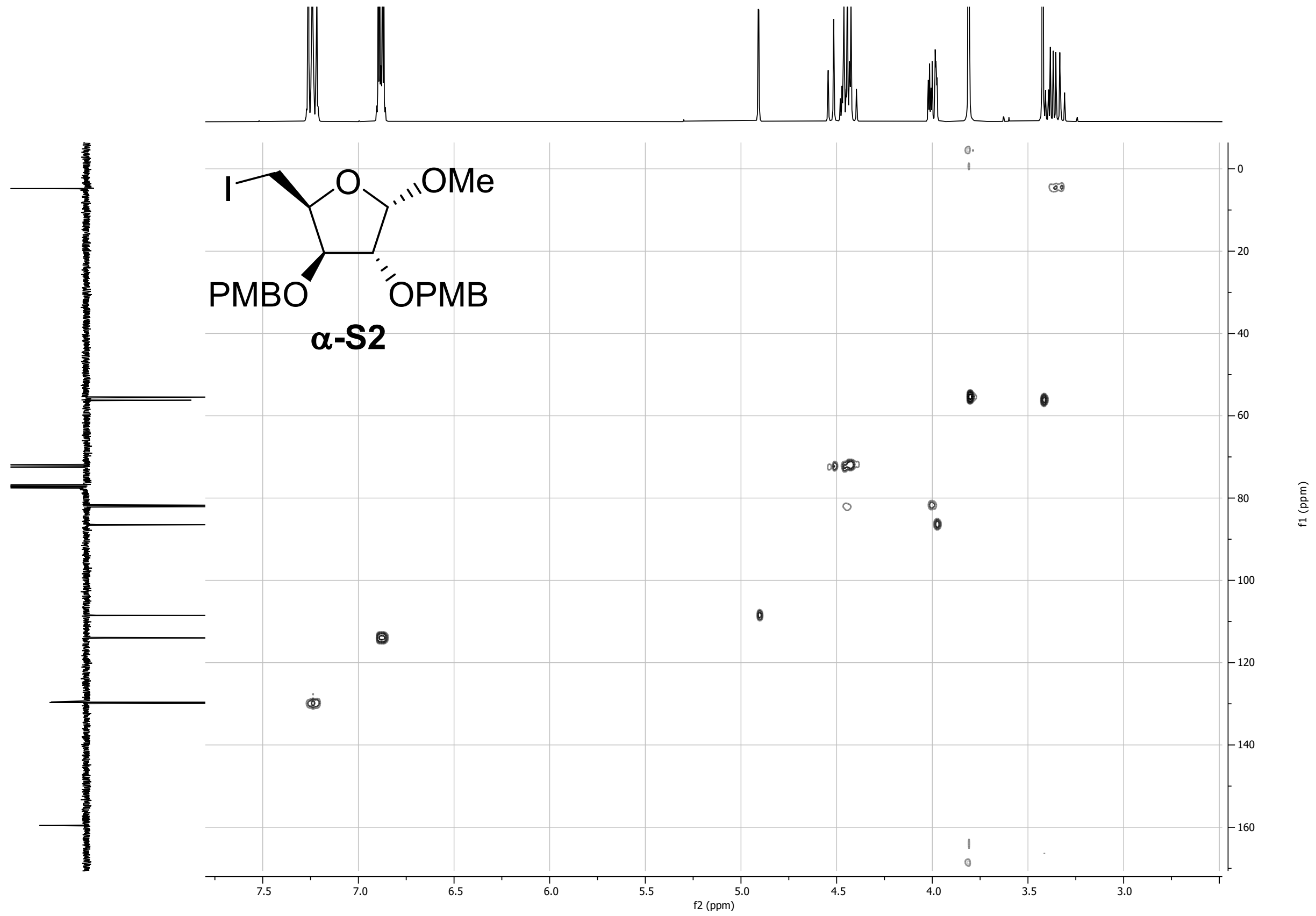

^{1}H-^{13}C HSQC (400 MHz, $CDCl_3$) of compound **α-S2**

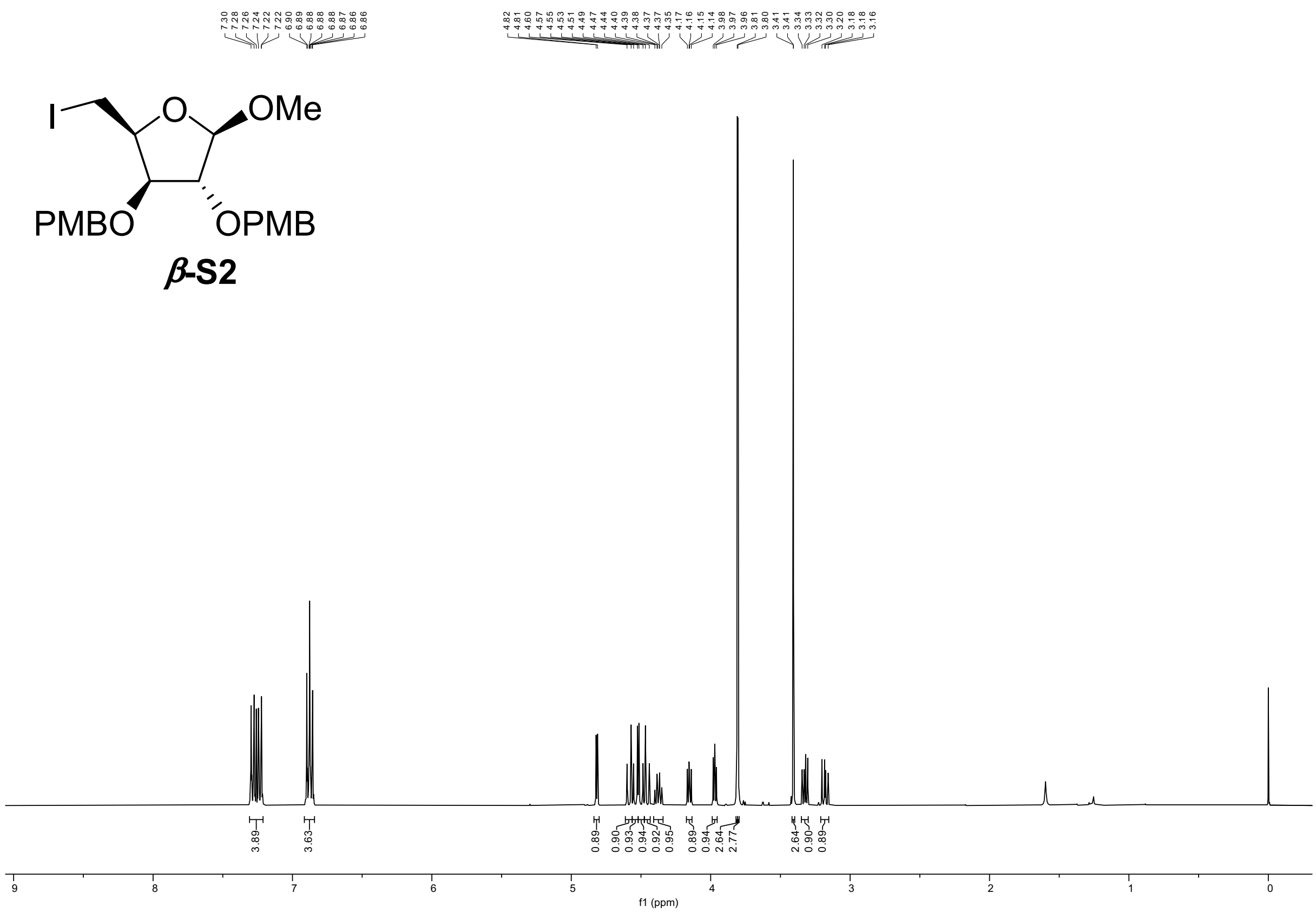

^{1}H NMR (400 MHz, $CDCl_3$) of compound **β-S2**

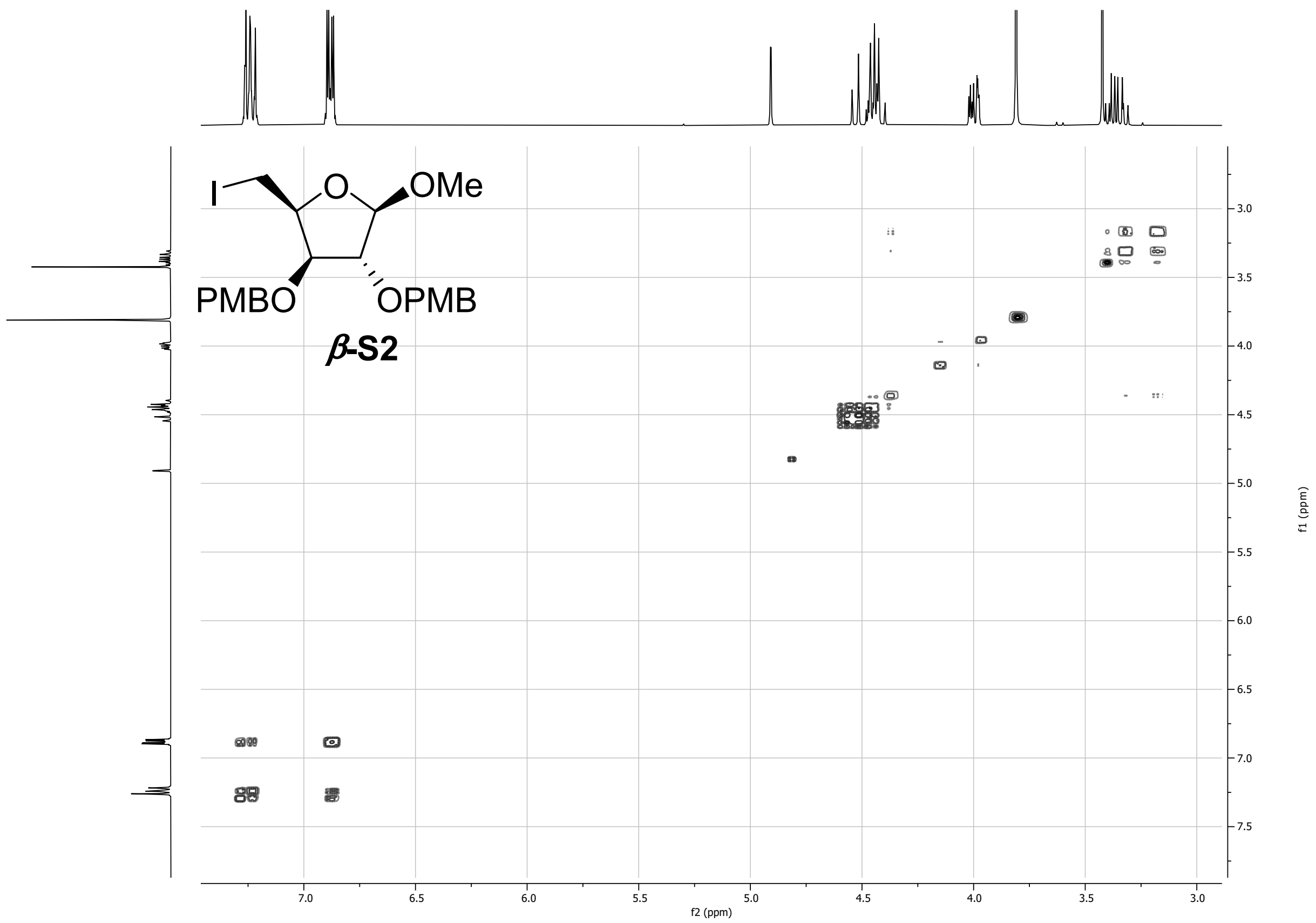

^{1}H-^{1}H COSY (400 MHz, $CDCl_3$) of compound **β-S2**

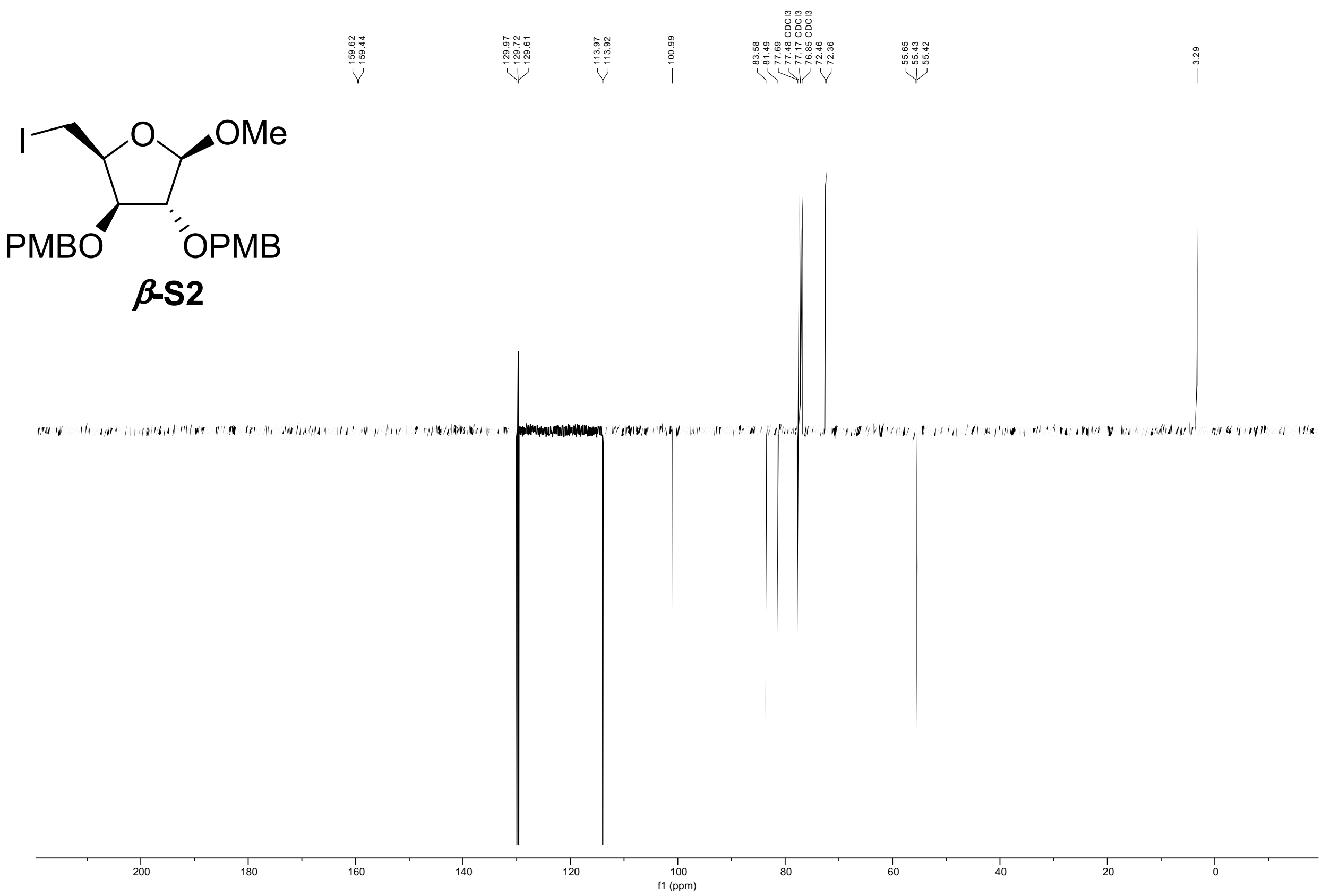

^{13}C NMR (101 MHz, CDCl$_3$) of compound **β-S2**

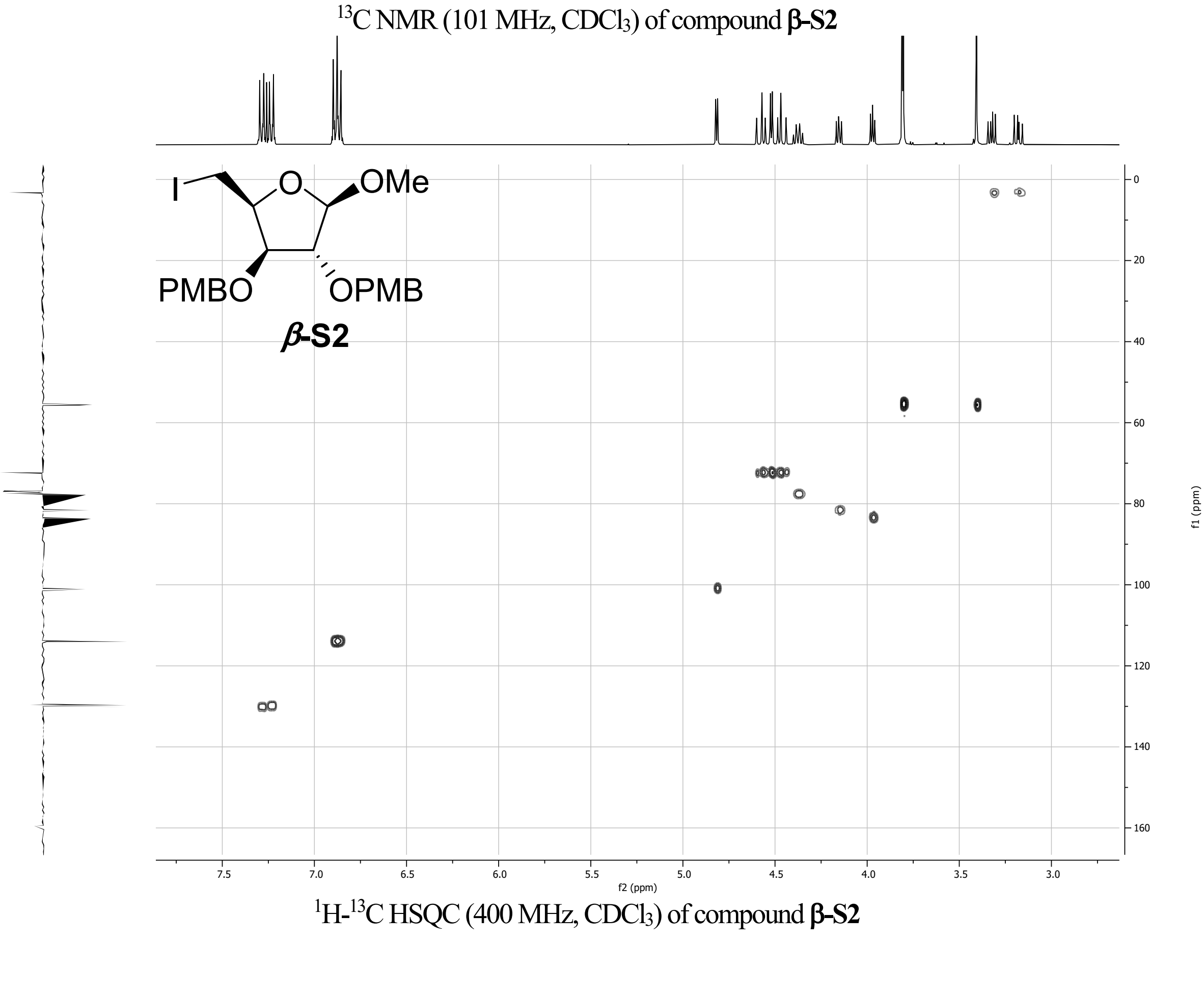

^{1}H-^{13}C HSQC (400 MHz, CDCl$_3$) of compound **β-S2**

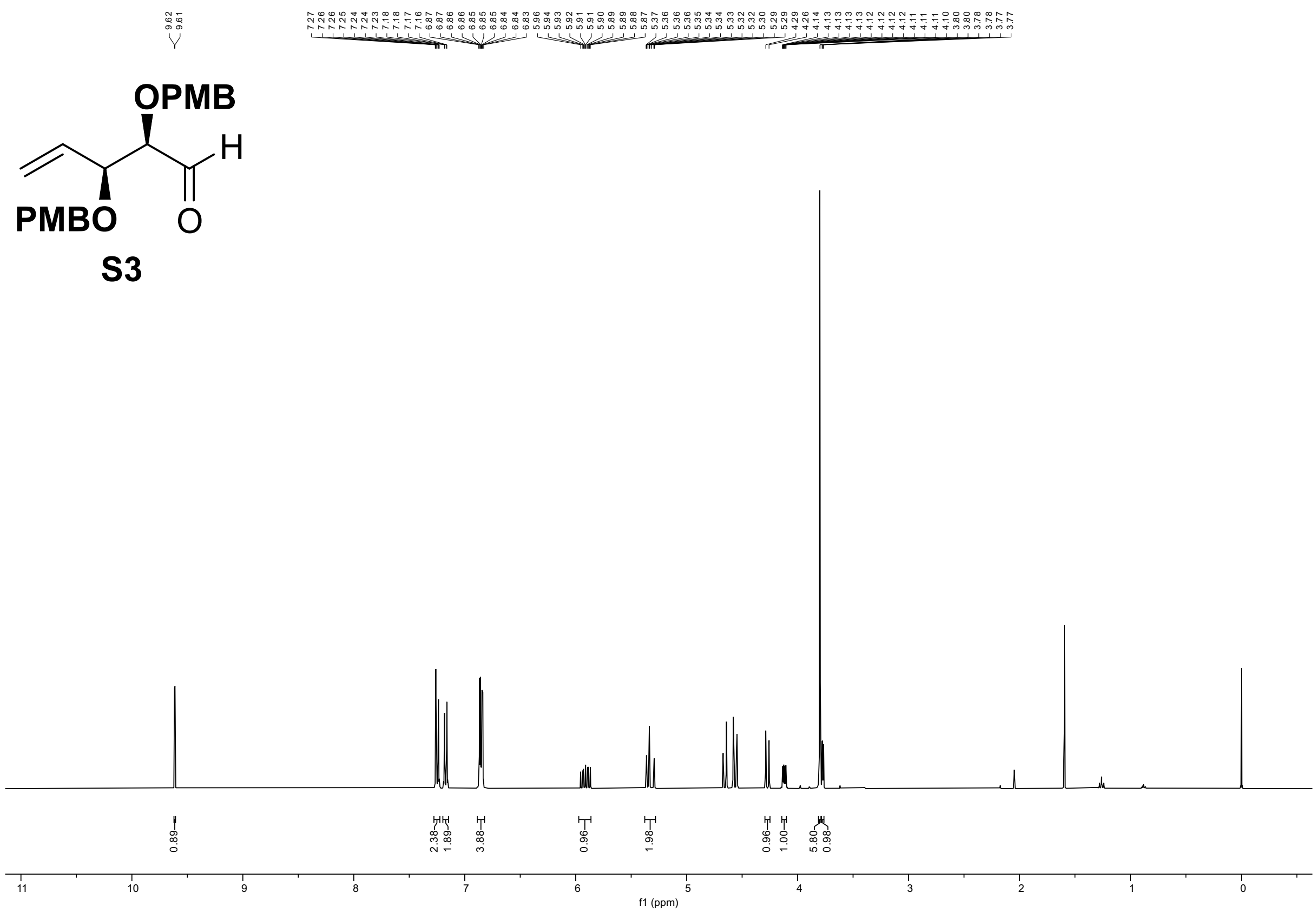

^{1}H NMR (400 MHz, $CDCl_3$) of compound **S3**

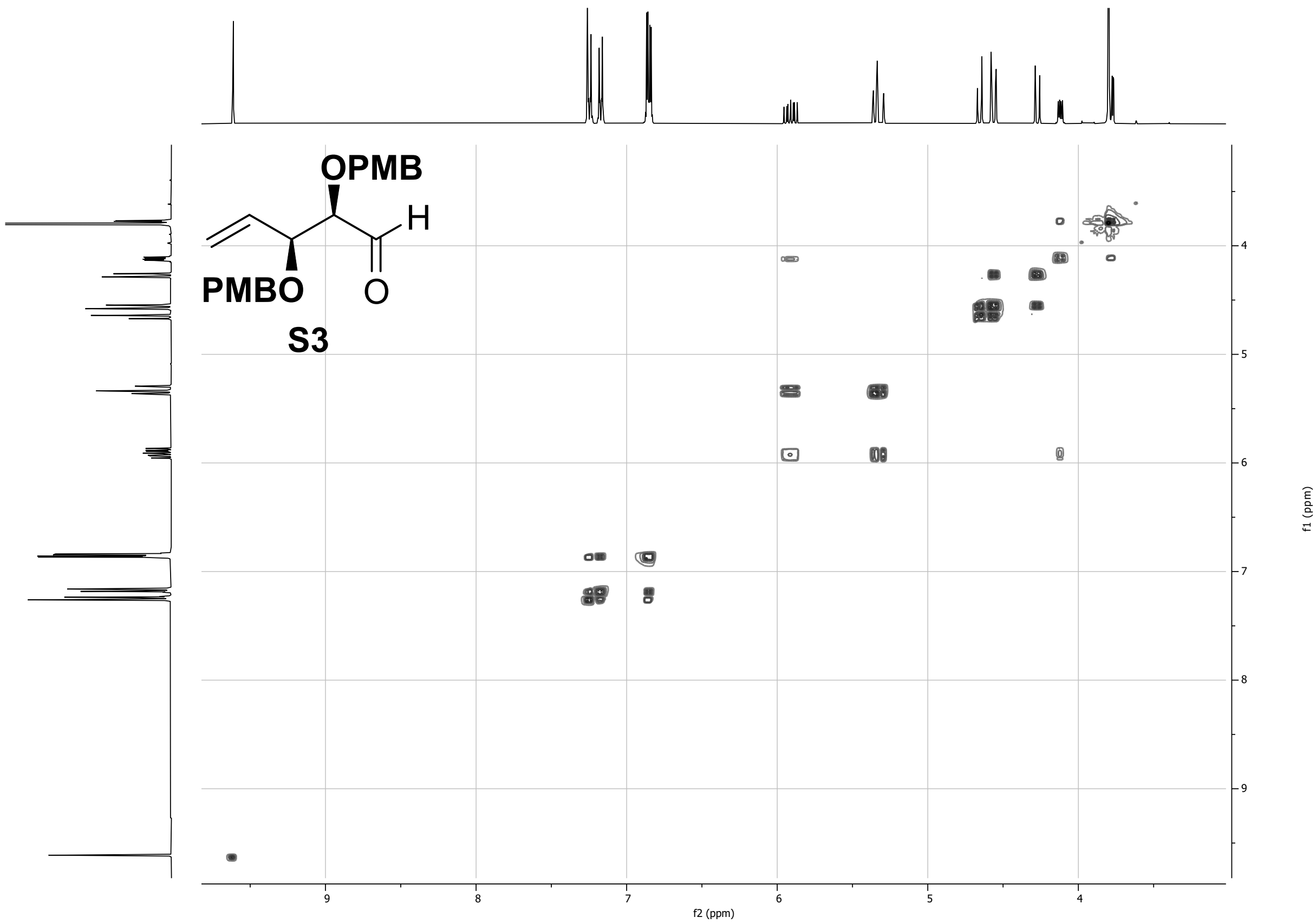

^{1}H-^{1}H COSY (400 MHz, $CDCl_3$) of compound **S3**

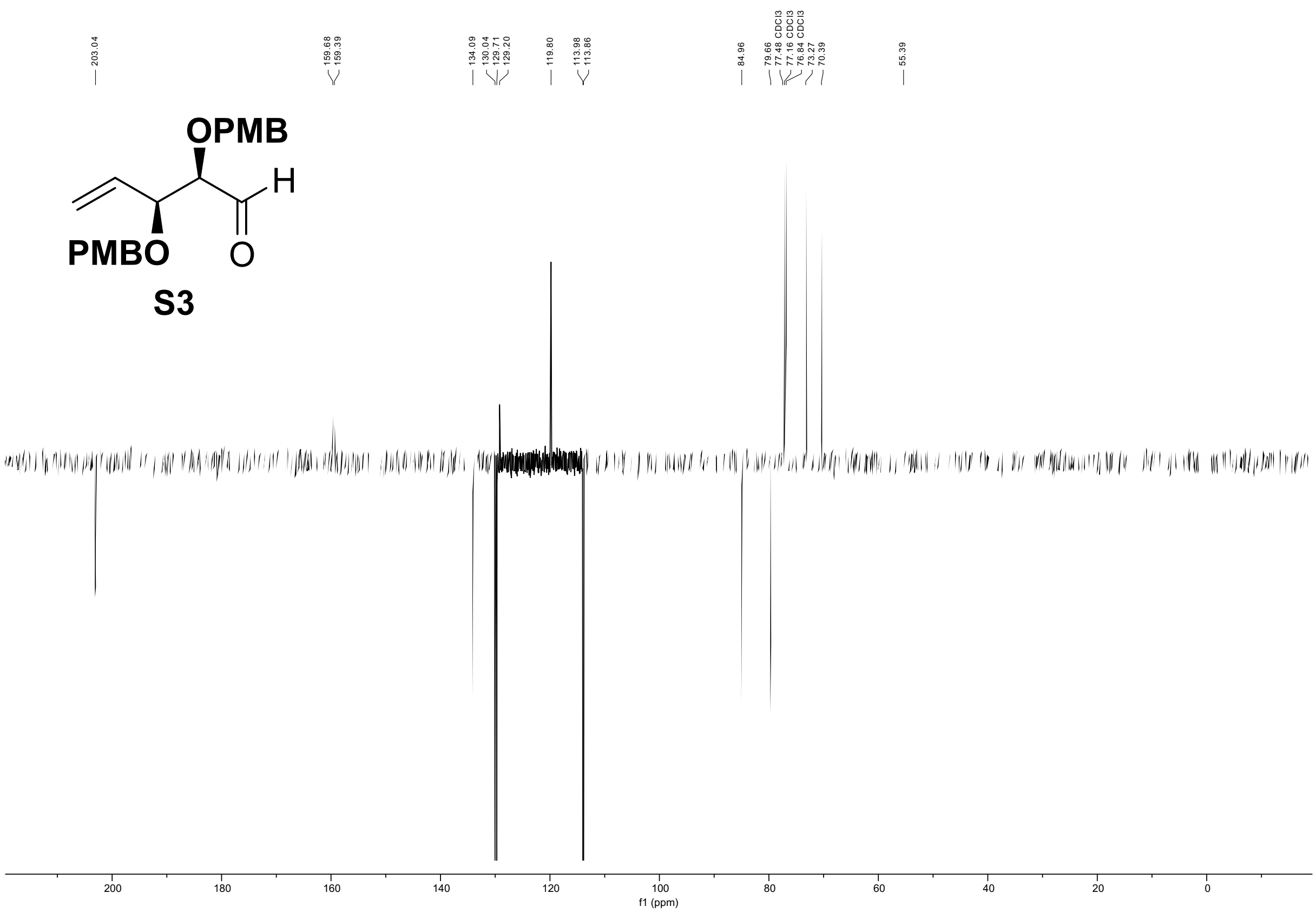

^{13}C NMR (101 MHz, CDCl3) of compound **S3**

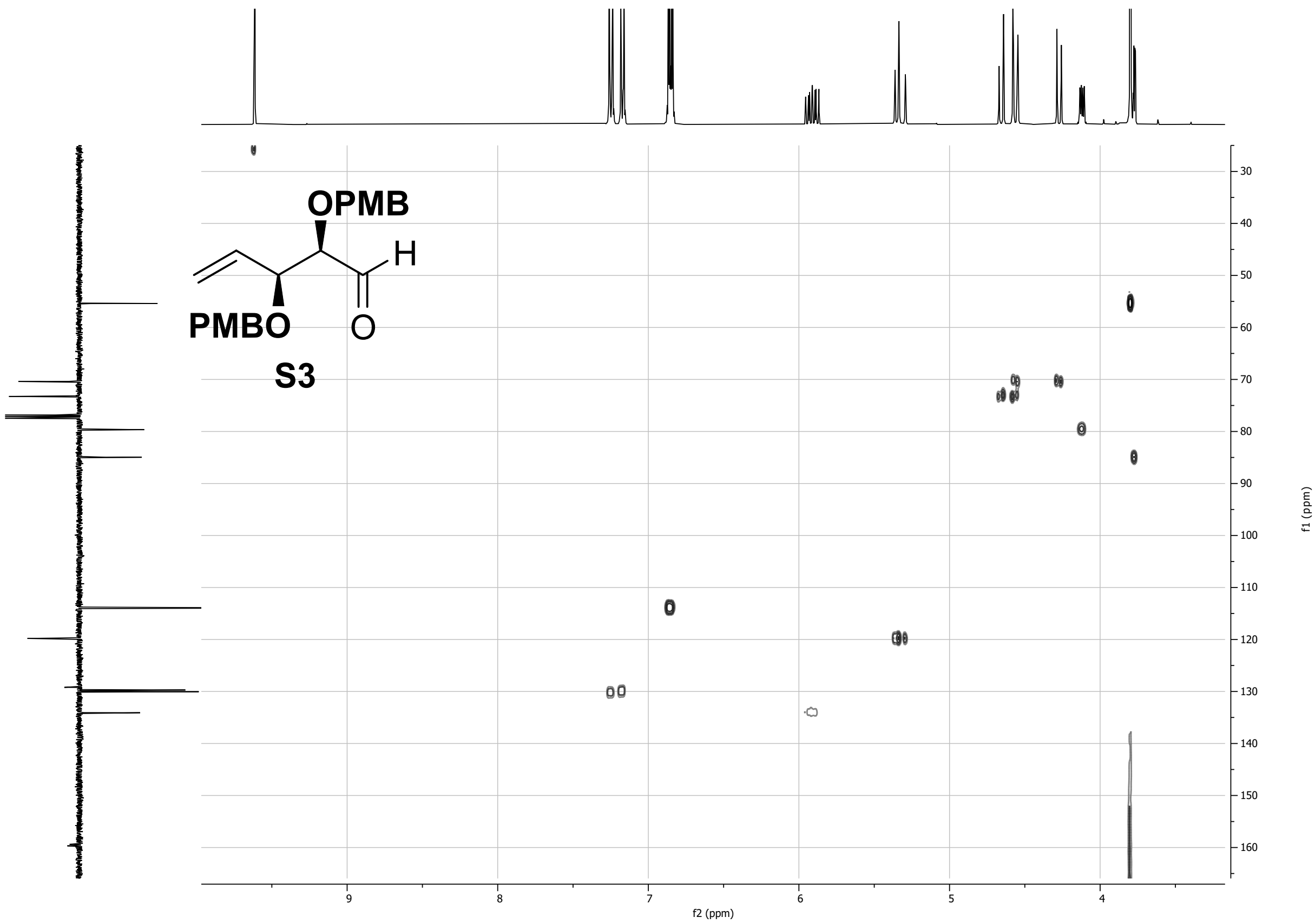

-^{13}C HSQC (400 MHz, $CDCl_3$) of compound **S3**

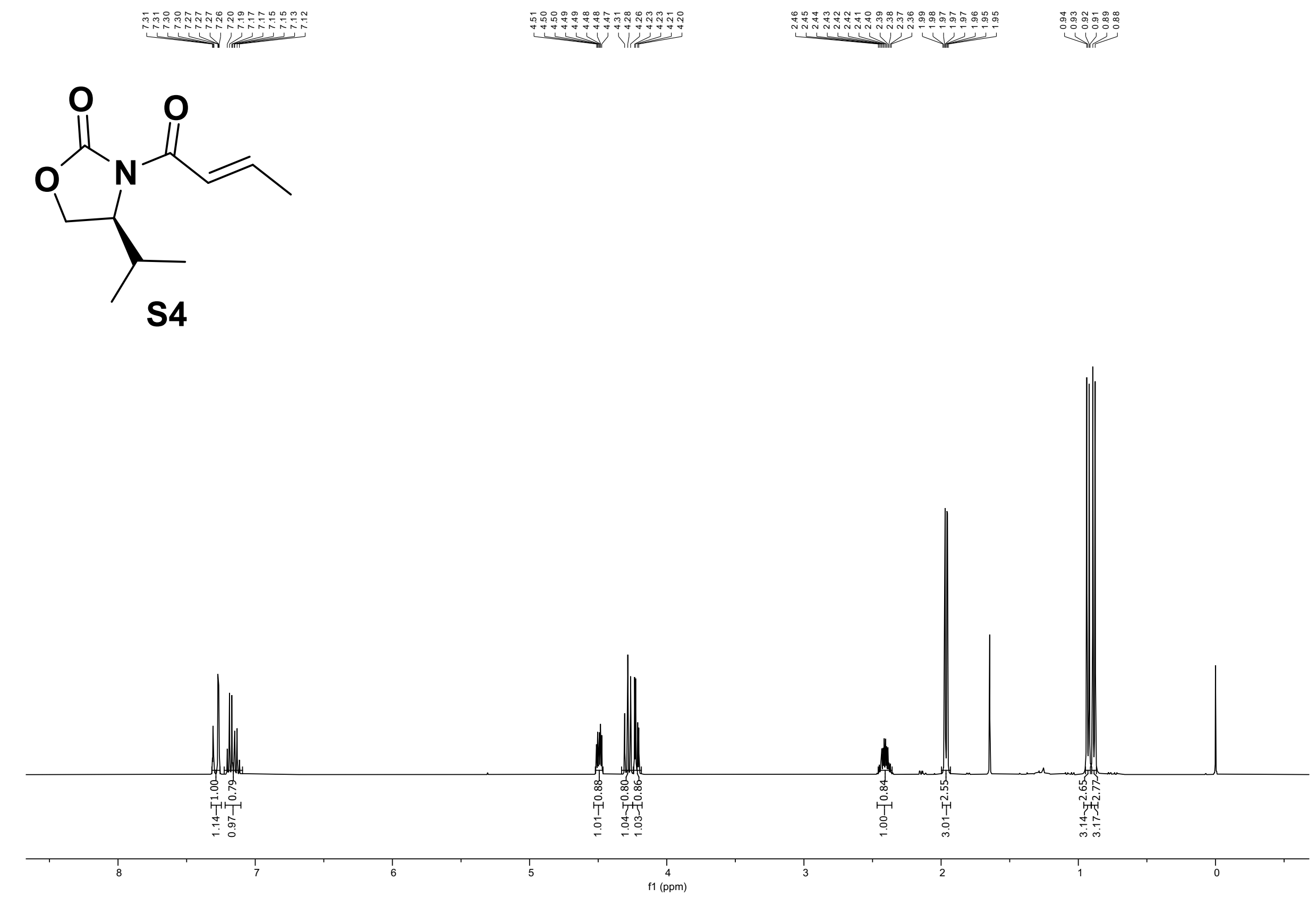

^{1}H NMR (400 MHz, $CDCl_3$) of compound **S4**

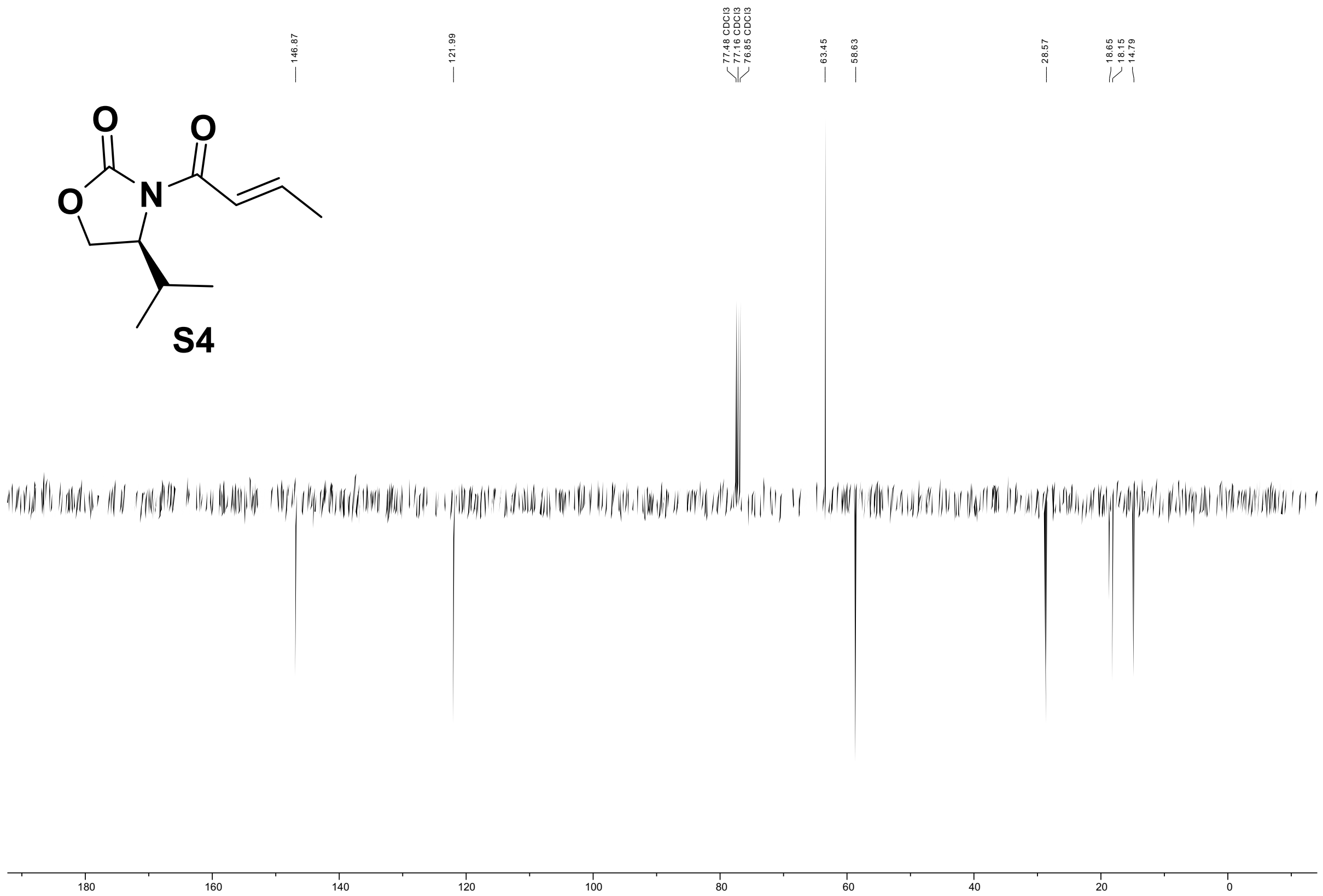

^{13}C NMR (101 MHz, $CDCl_3$) of compound **S4**

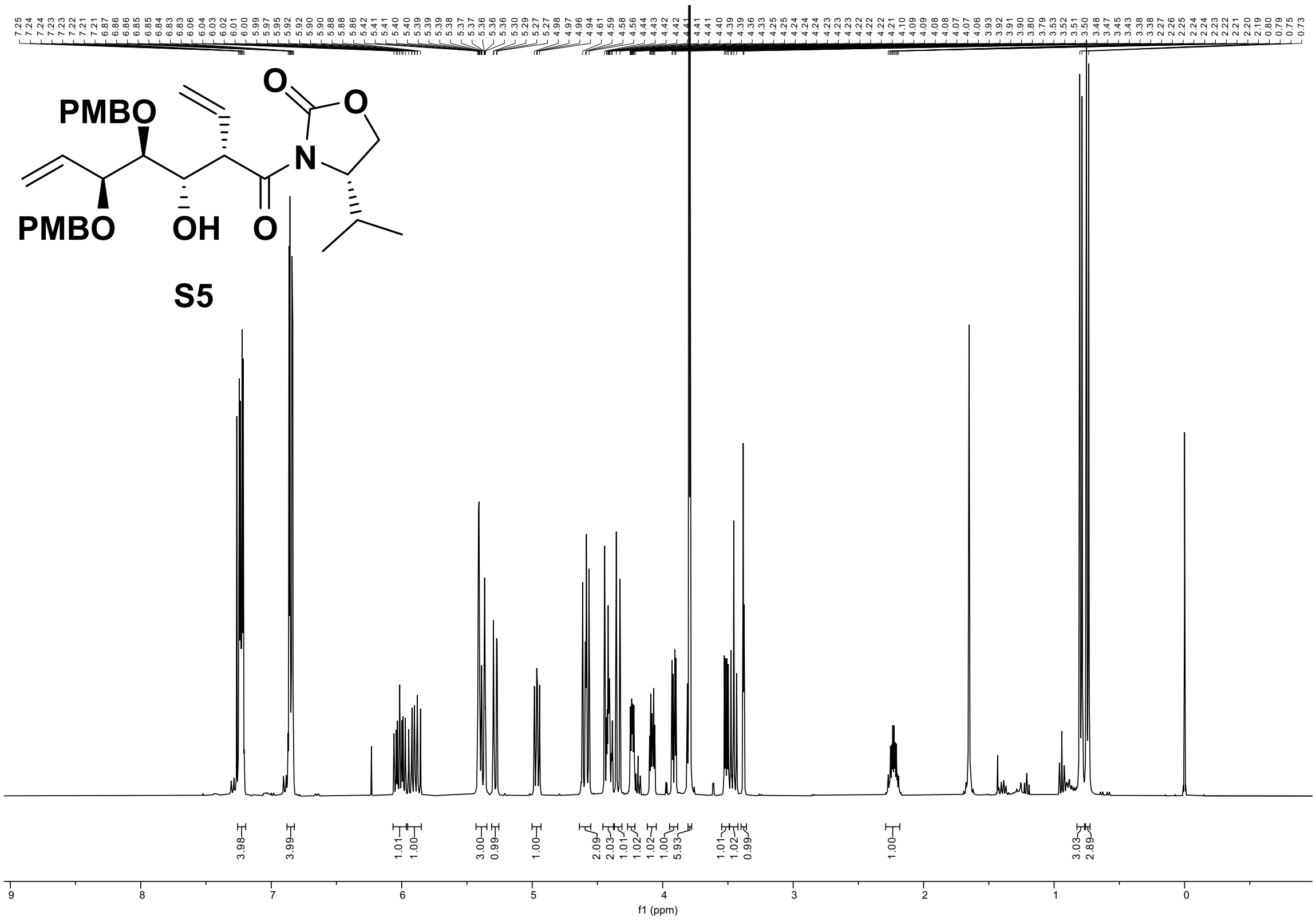

^{1}H NMR (400 MHz, $CDCl_3$) of compound **S5**

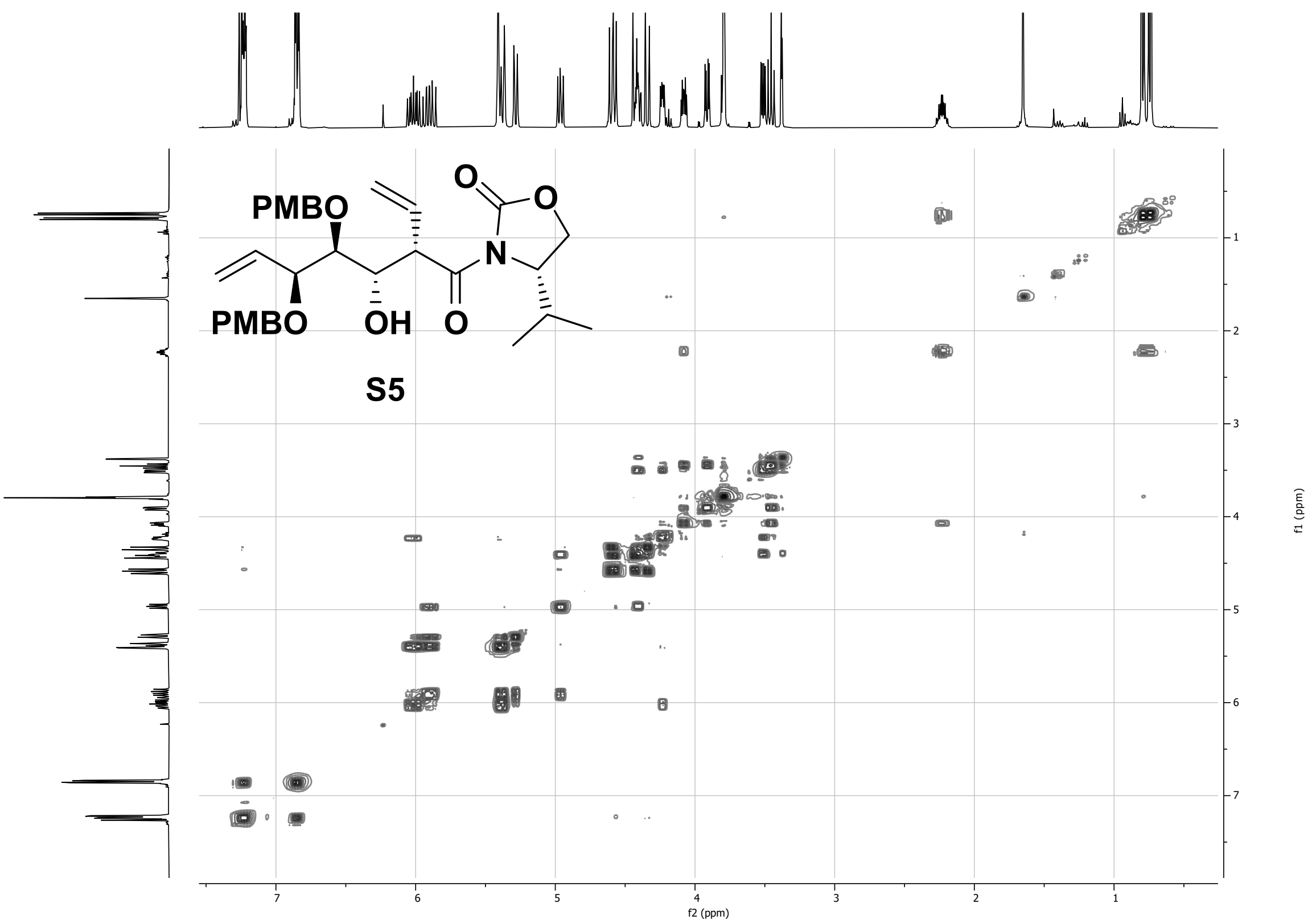

^{1}H-^{1}H COSY (400 MHz, $CDCl_3$) of compound **S5**

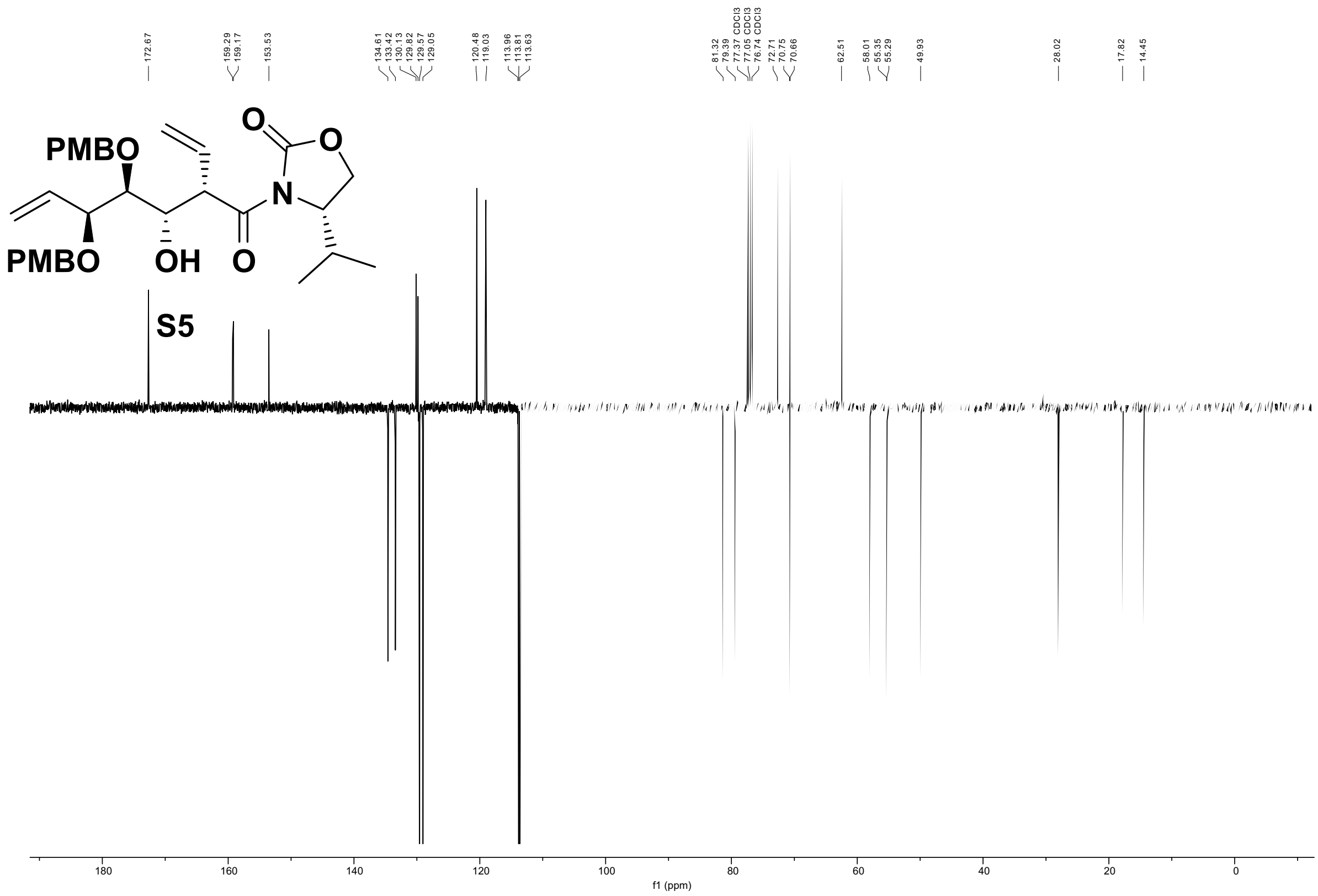

^{13}C NMR (101 MHz, $CDCl_3$) of compound **S5**

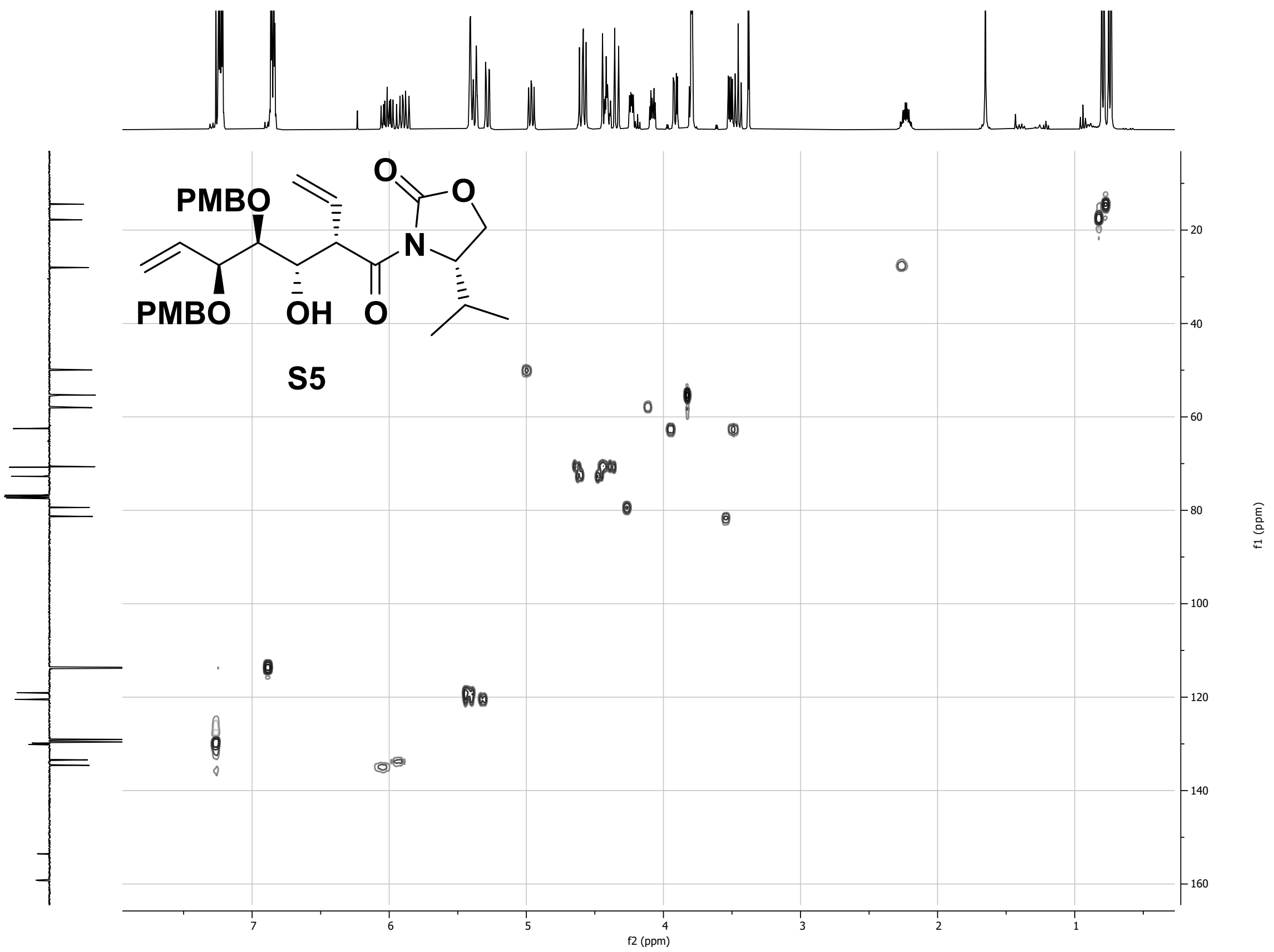

^{1}H-^{13}C HSQC (400 MHz, $CDCl_3$) of compound **S5**

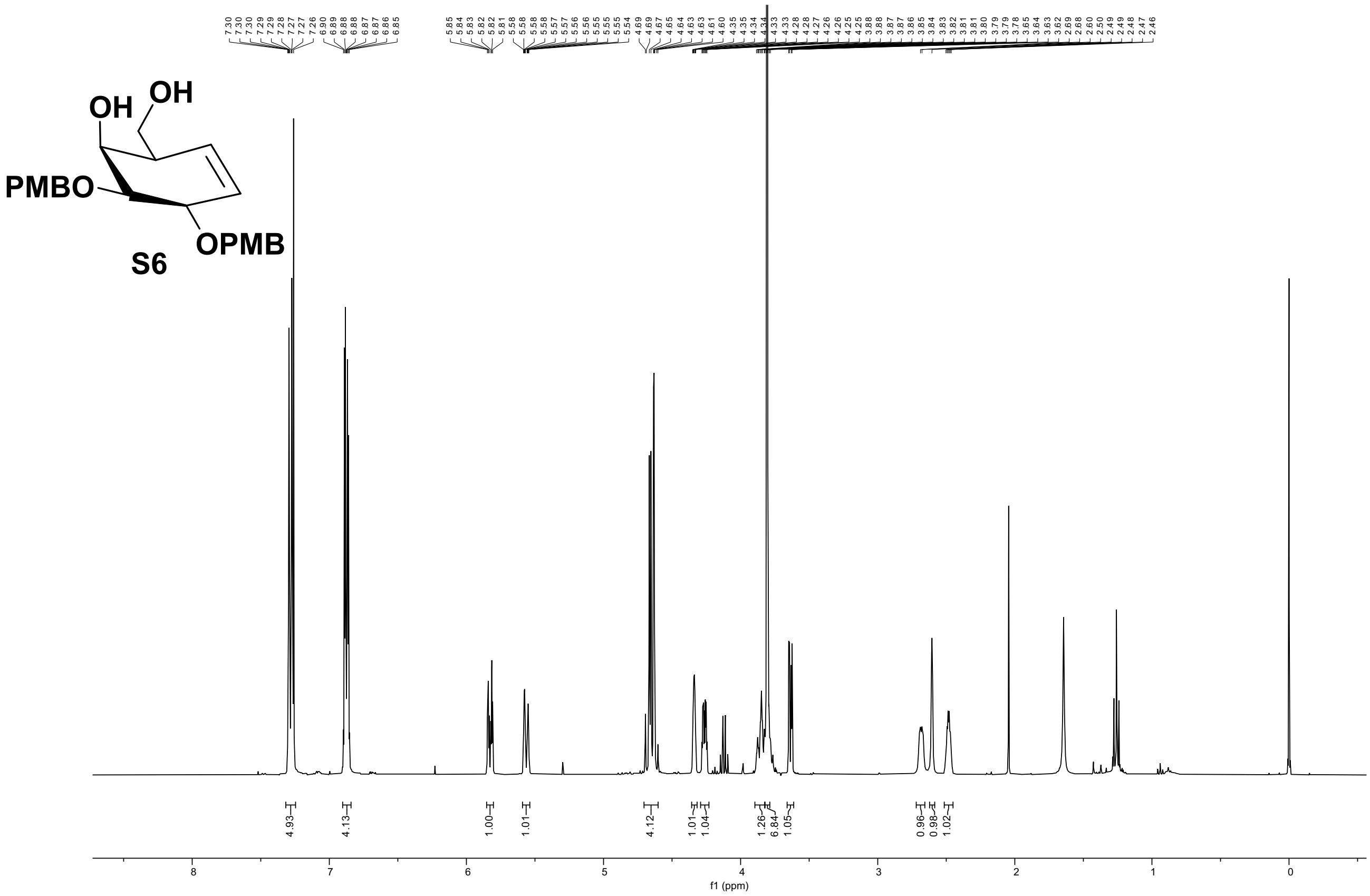

^{1}H NMR (400 MHz, $CDCl_3$) of compound **S6**

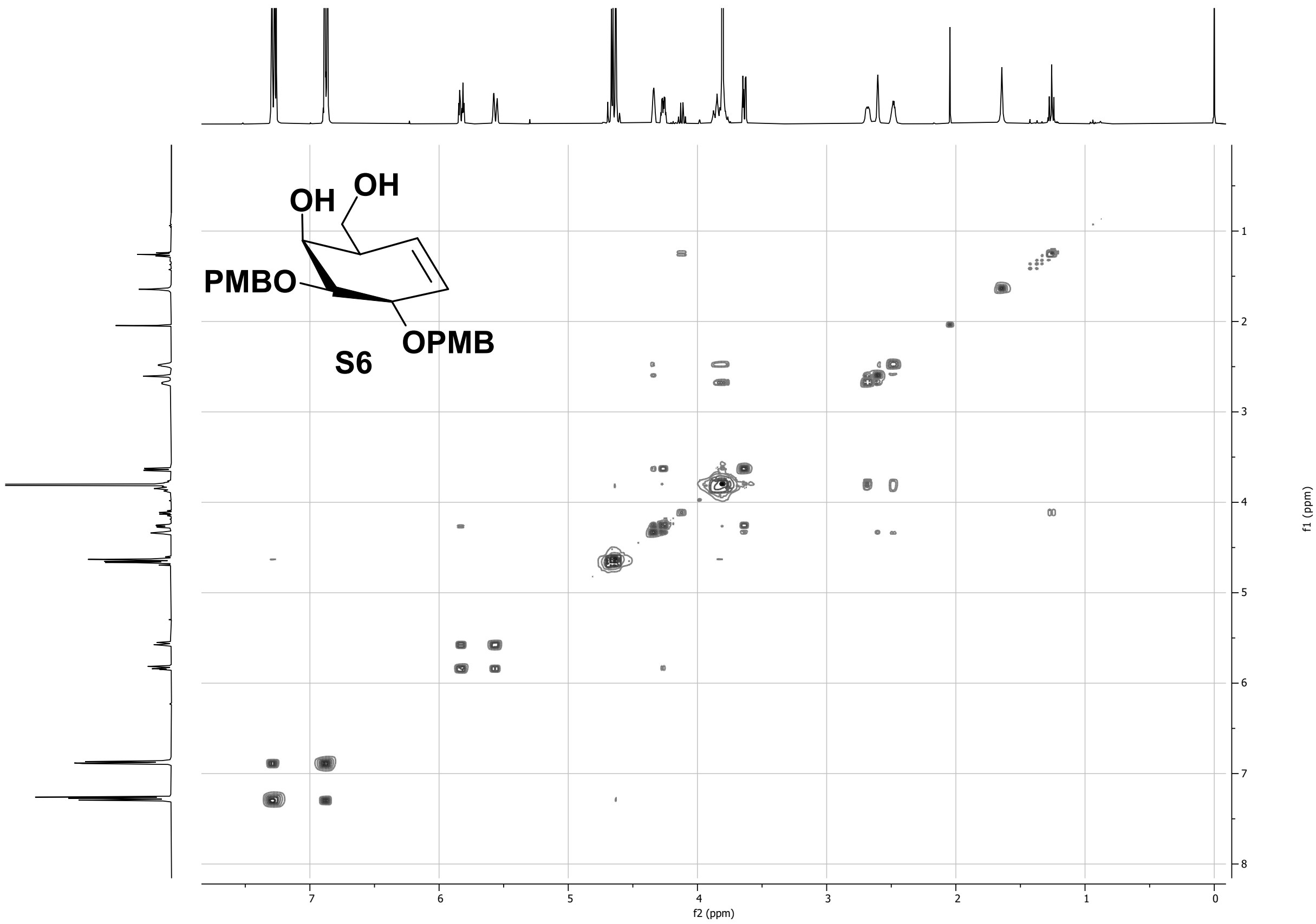

^{1}H-^{1}H COSY (400 MHz, $CDCl_3$) of compound **S6**

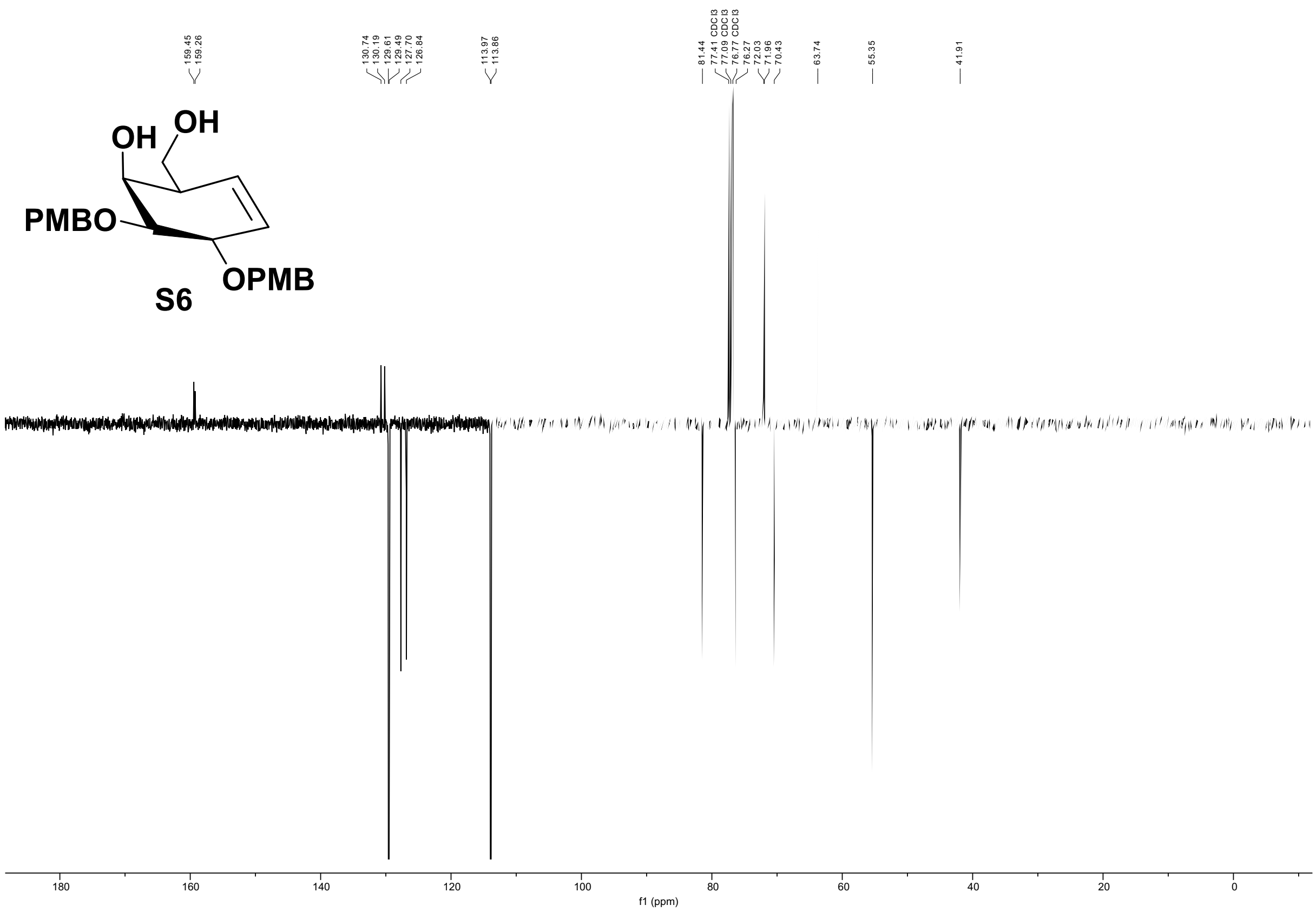

^{13}C NMR (101 MHz, $CDCl_3$) of compound **S6**

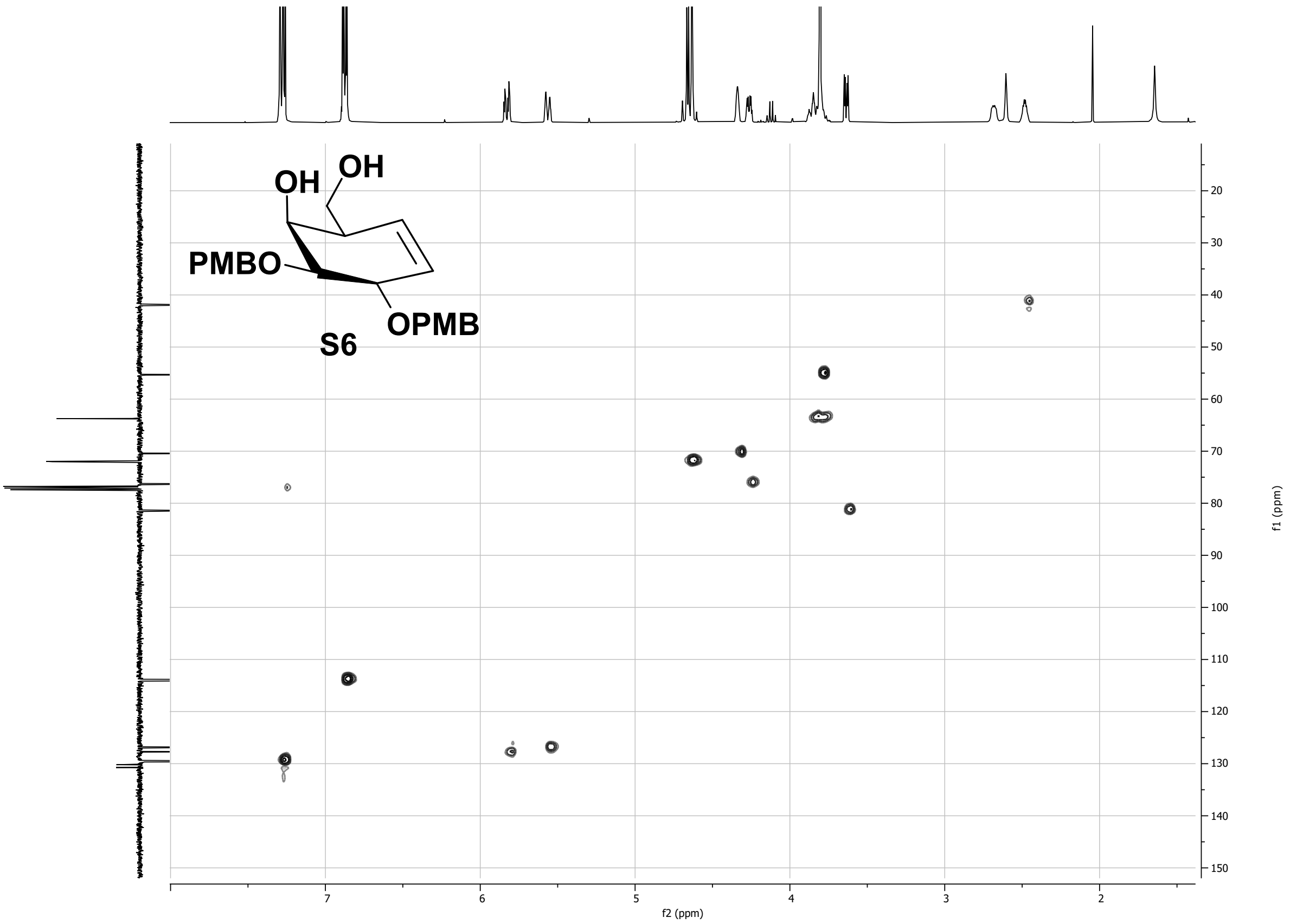

^{1}H-^{13}C HSQC (400 MHz, $CDCl_3$) of compound **S6**

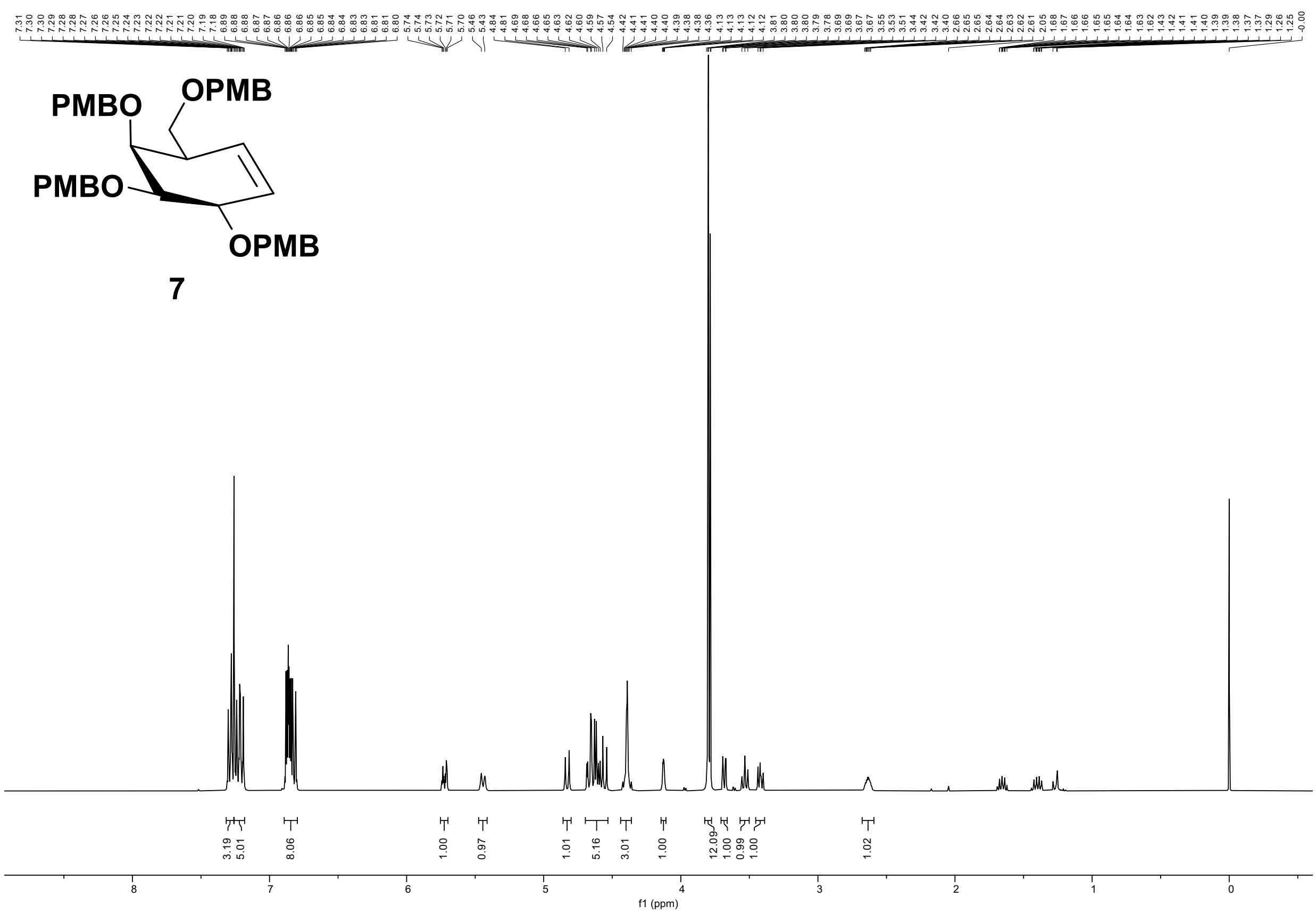

^{1}H NMR (400 MHz, $CDCl_3$) of compound **7**

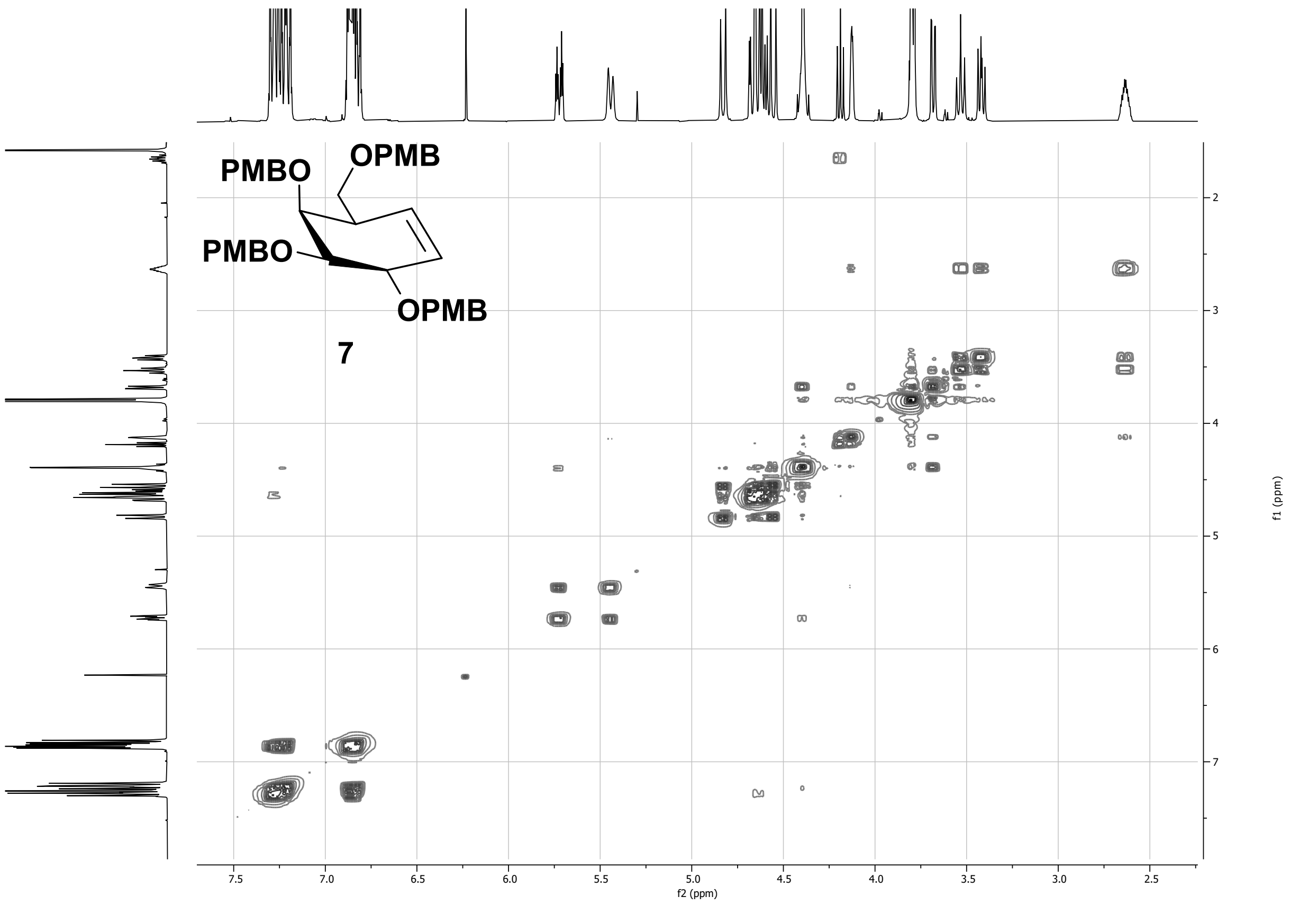

^{1}H-^{1}H COSY (400 MHz, $CDCl_3$) of compound **7**

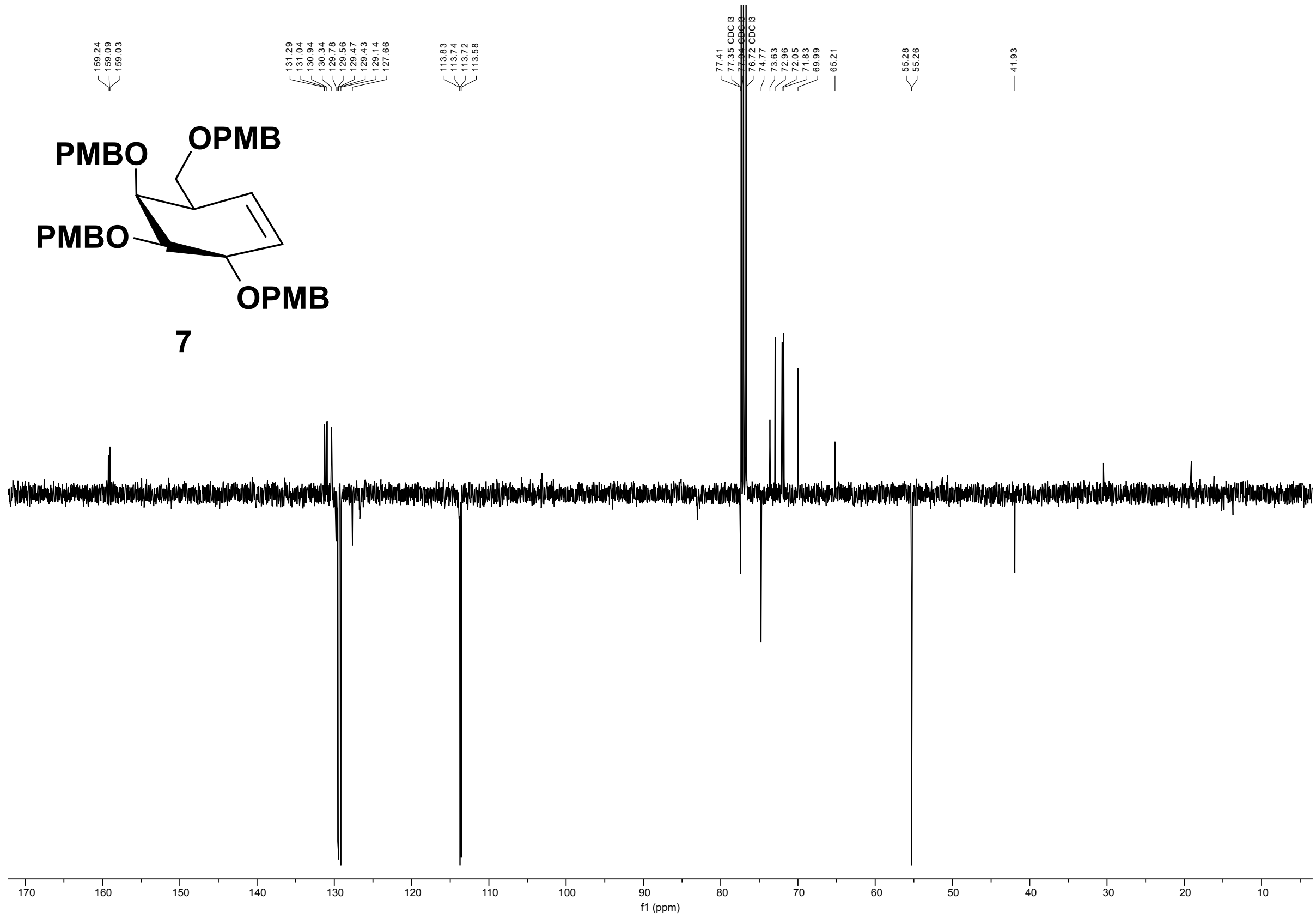

^{13}C NMR (101 MHz, $CDCl_3$) of compound **7**

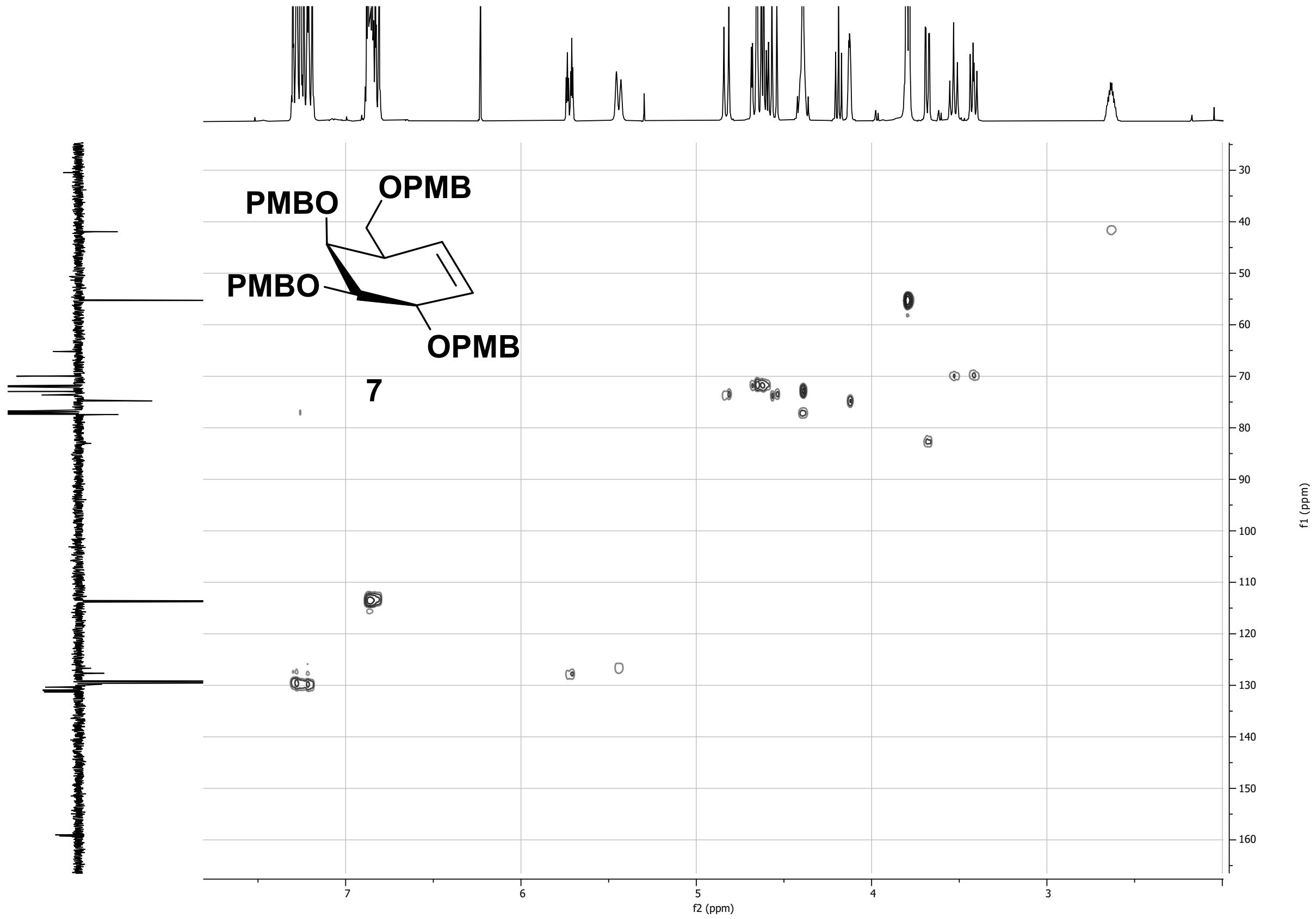

^{1}H-^{13}C HSQC (400 MHz, $CDCl_3$) of compound **7**

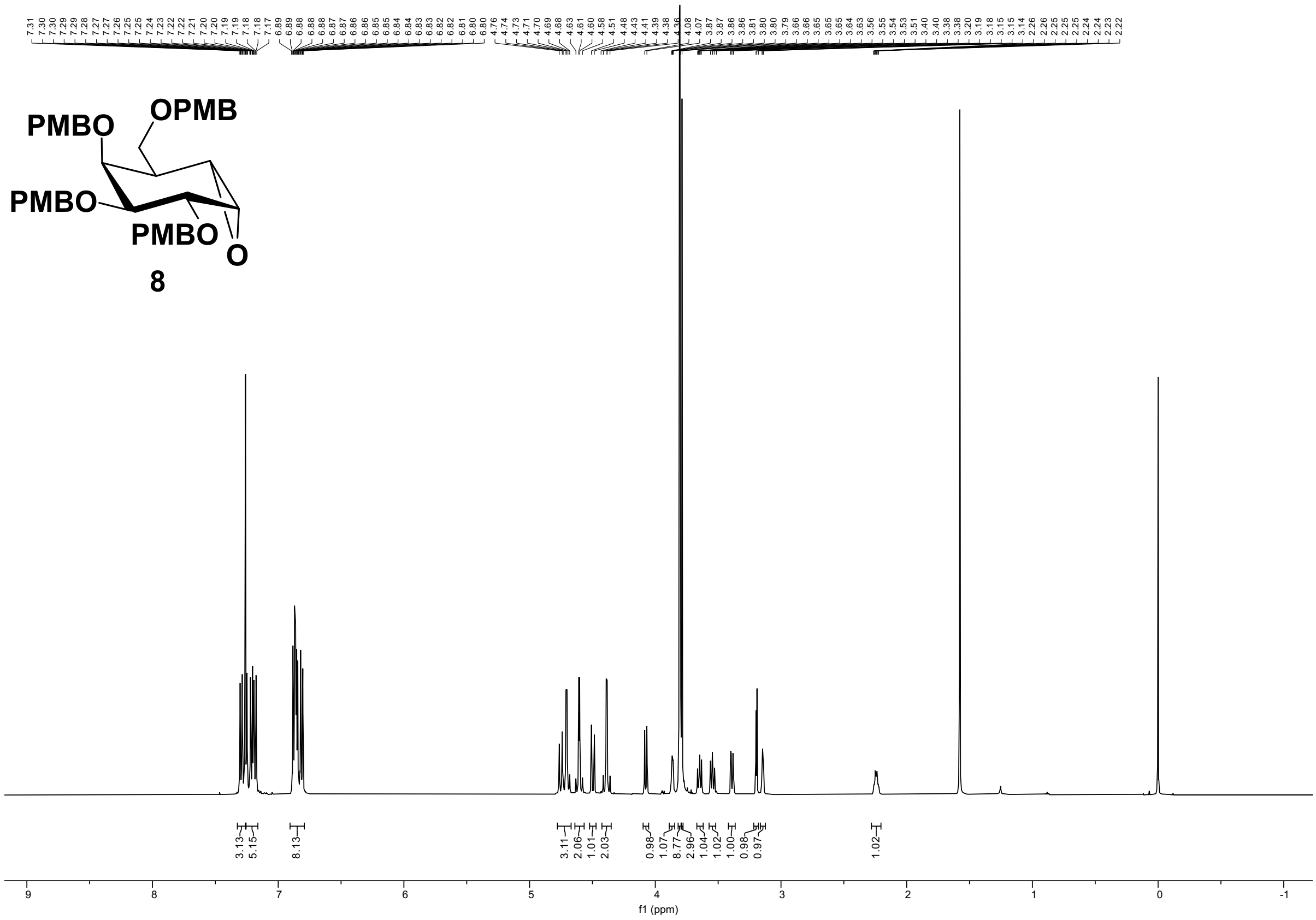

^{1}H NMR (500 MHz, $CDCl_3$) of compound **8**

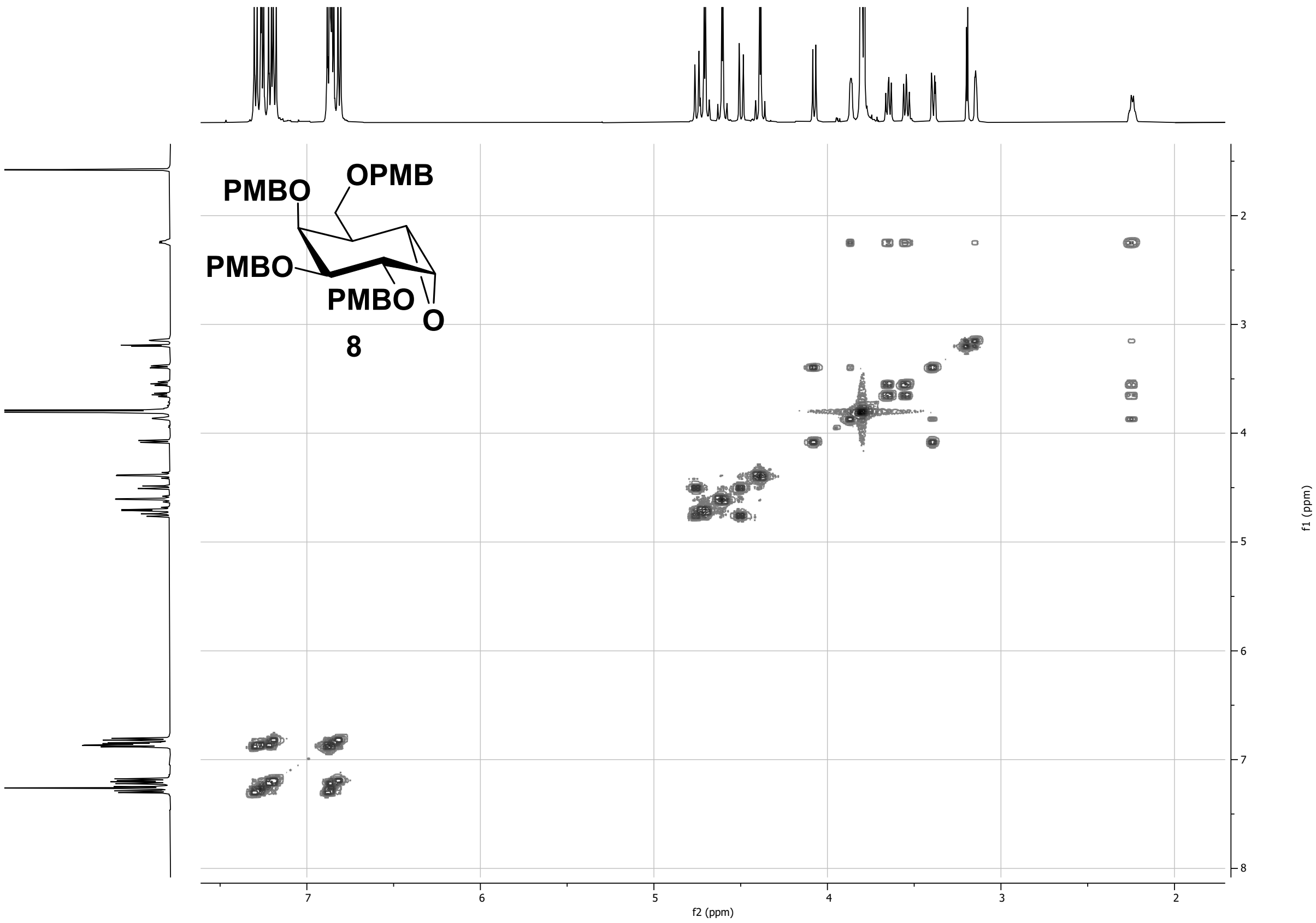

^{1}H-^{1}H COSY (500 MHz, $CDCl_3$) of compound **8**

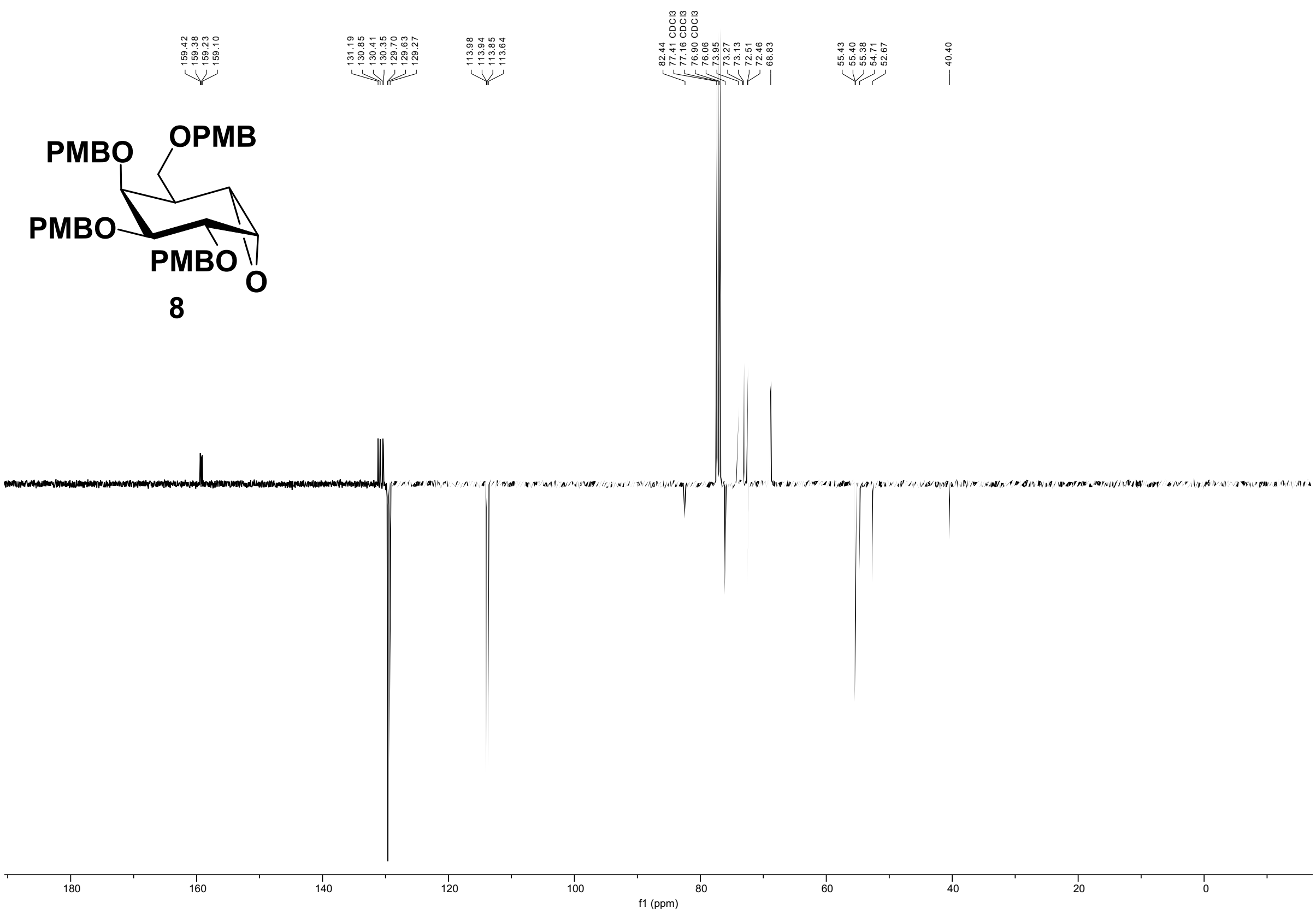

^{13}C NMR (126 MHz, CDCl$_3$) of compound **8**

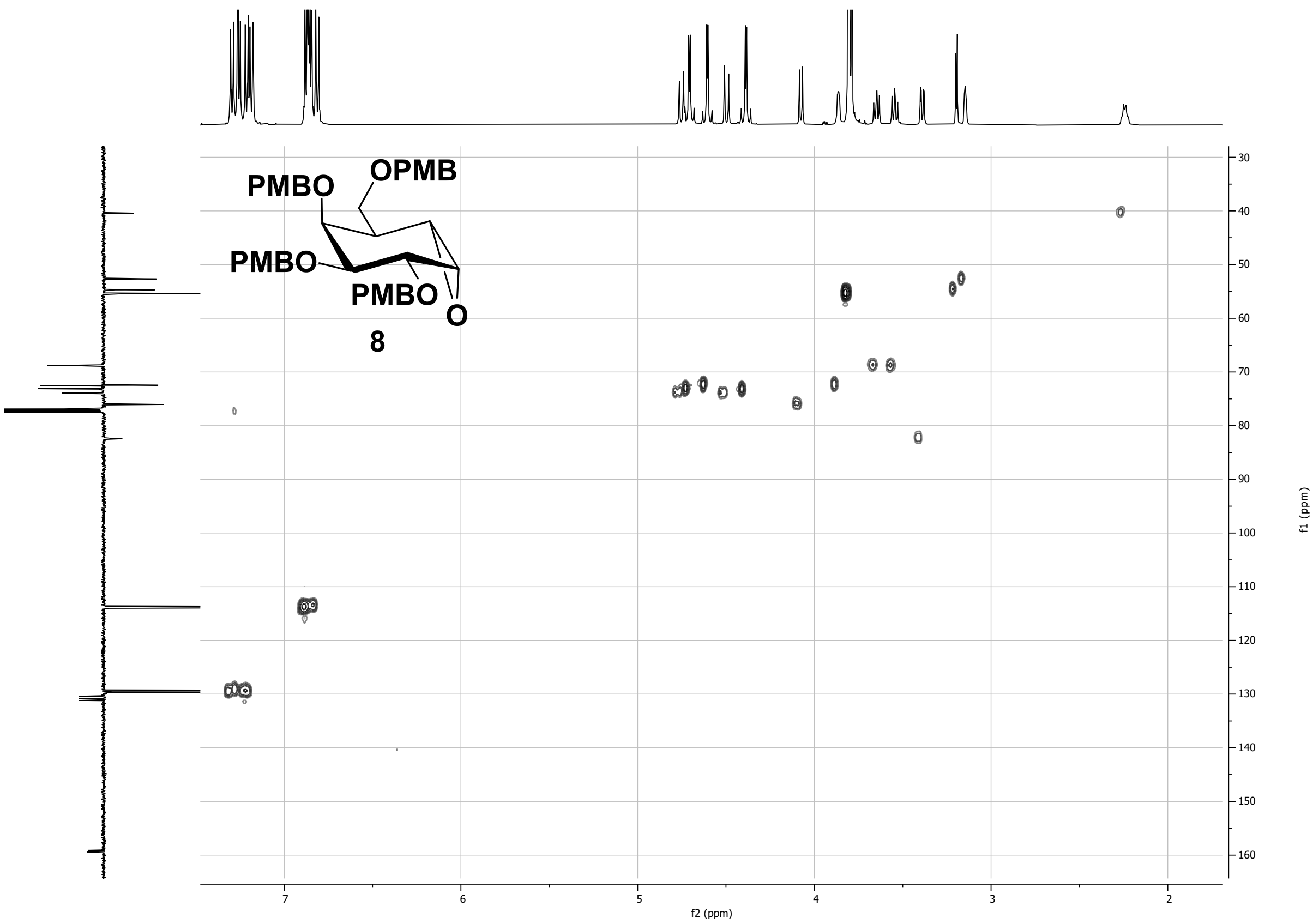

^{1}H-^{13}C HSQC (500 MHz, CDCl$_3$) of compound **8**

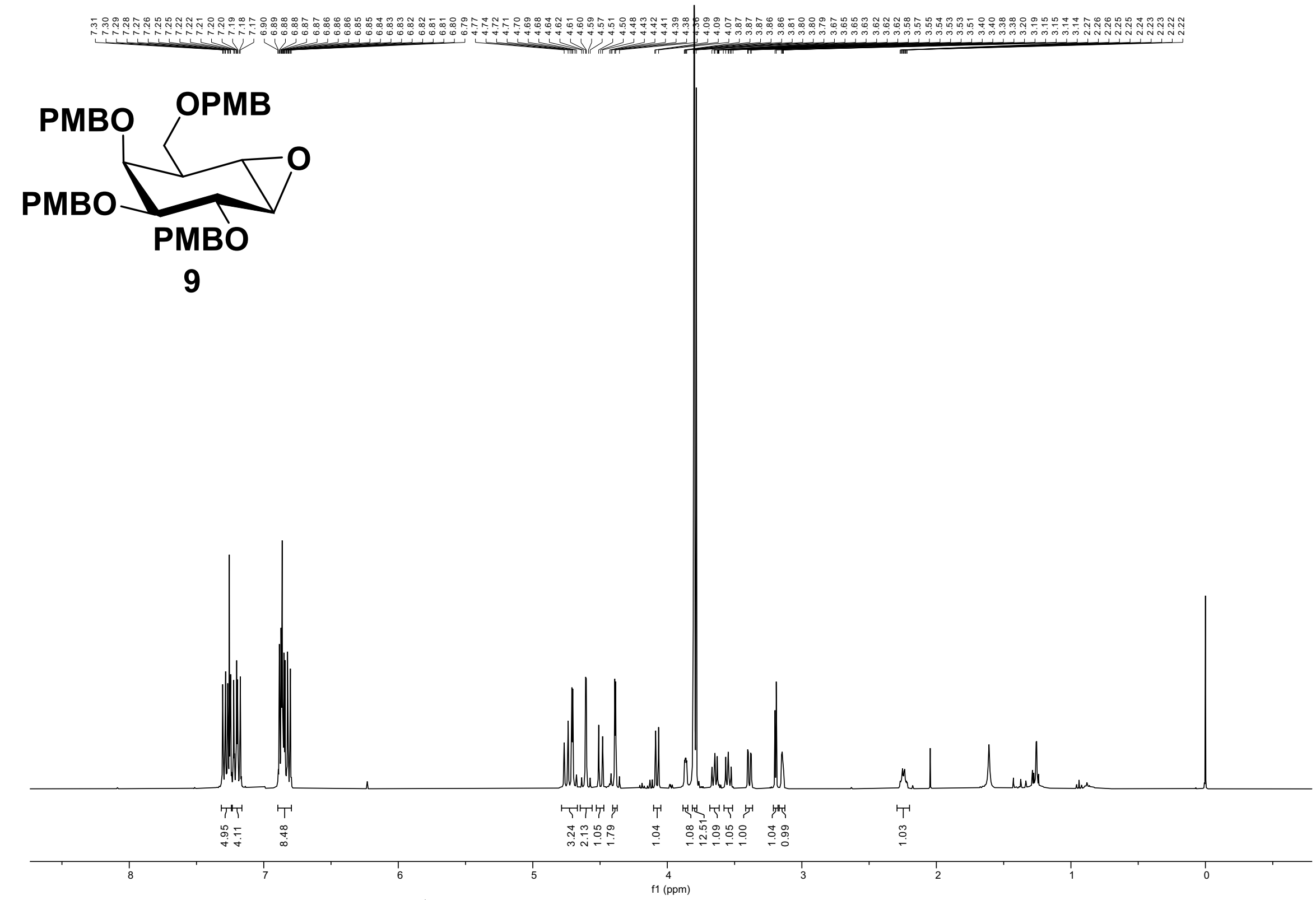

^{1}H NMR (400 MHz, $CDCl_3$) of compound **9**

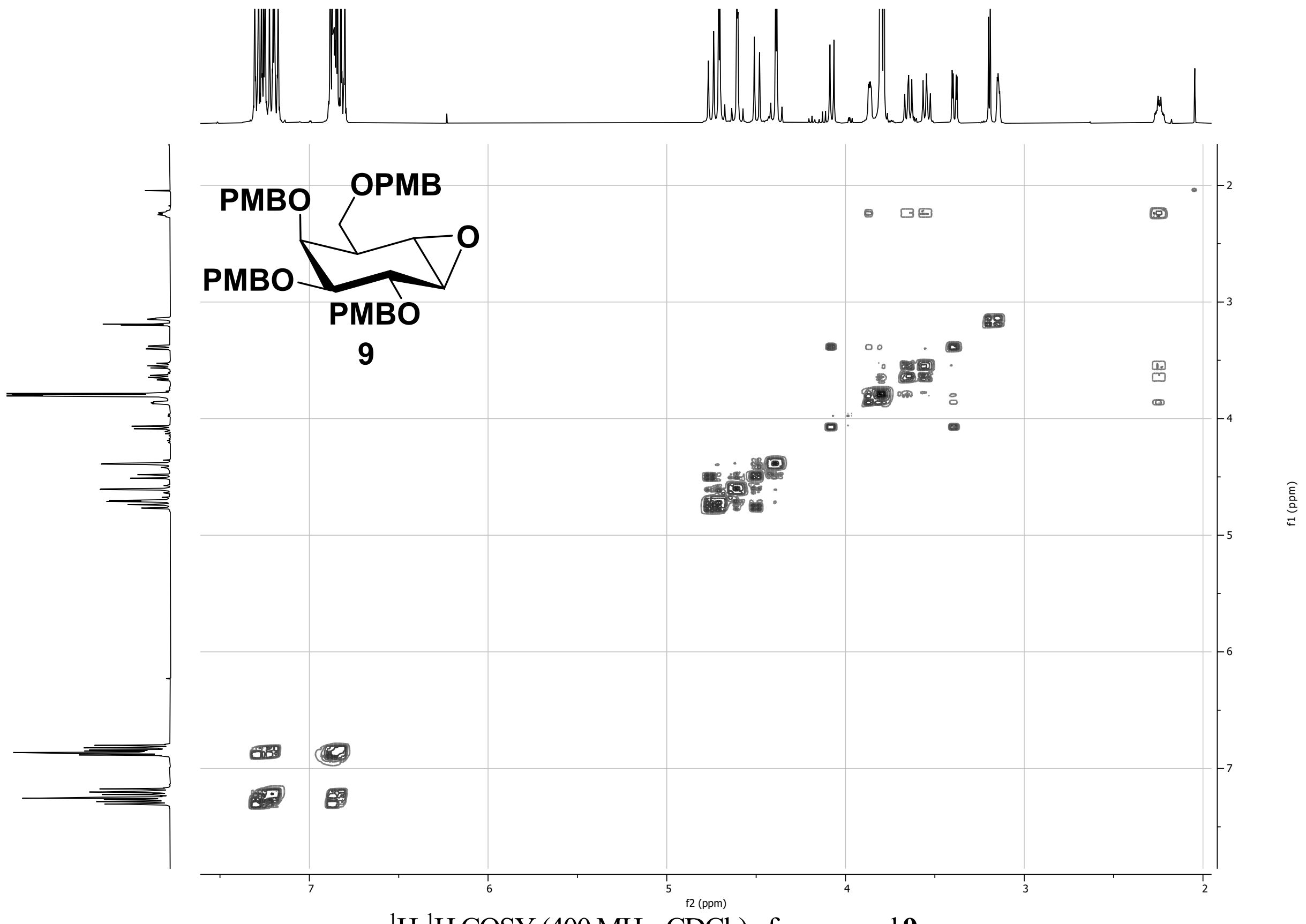

^{1}H-^{1}H COSY (400 MHz, $CDCl_3$) of compound **9**

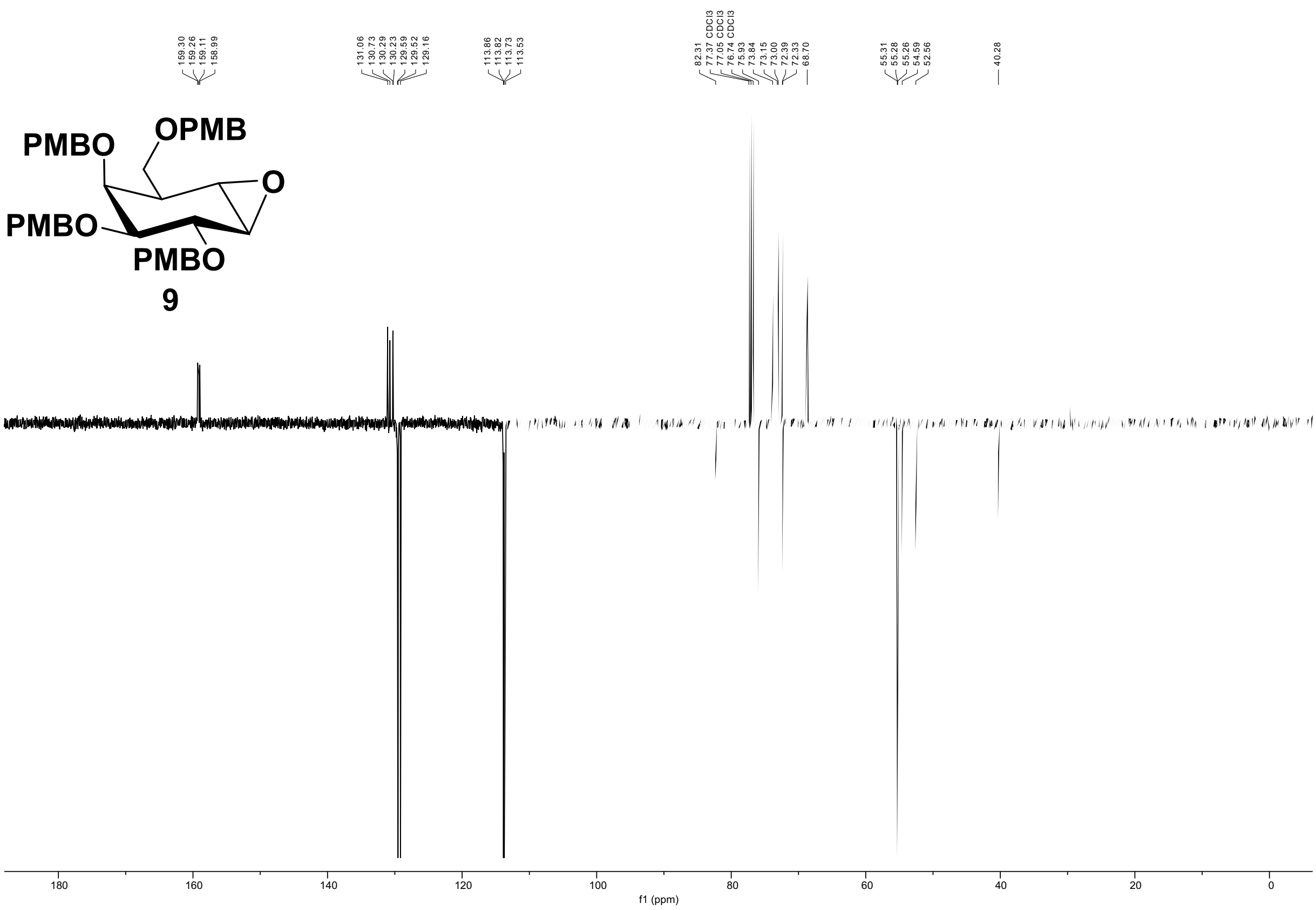

^{13}C NMR (101 MHz, $CDCl_3$) of compound **9**

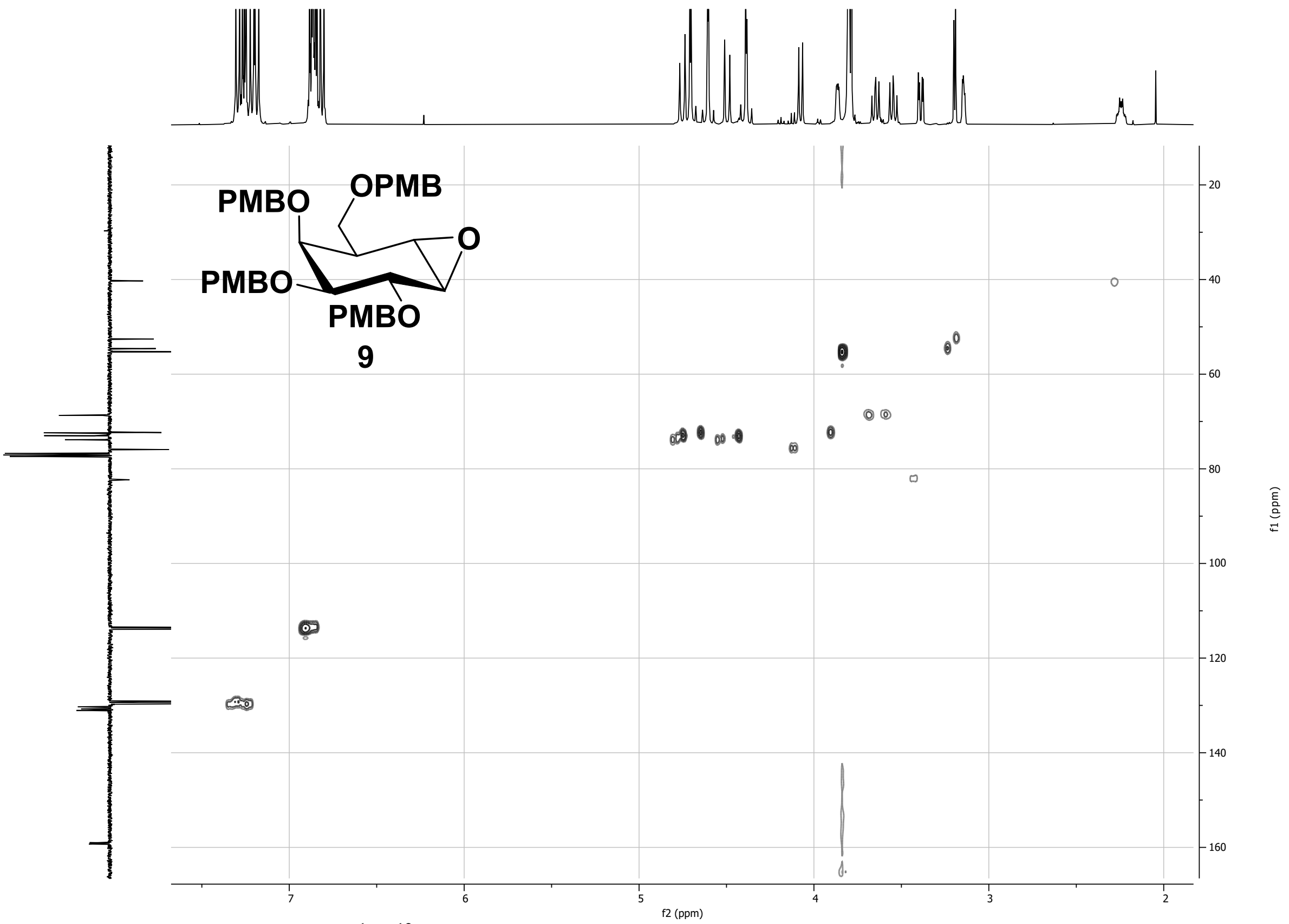

^{1}H-^{13}C HSQC (400 MHz, $CDCl_3$) of compound **9**

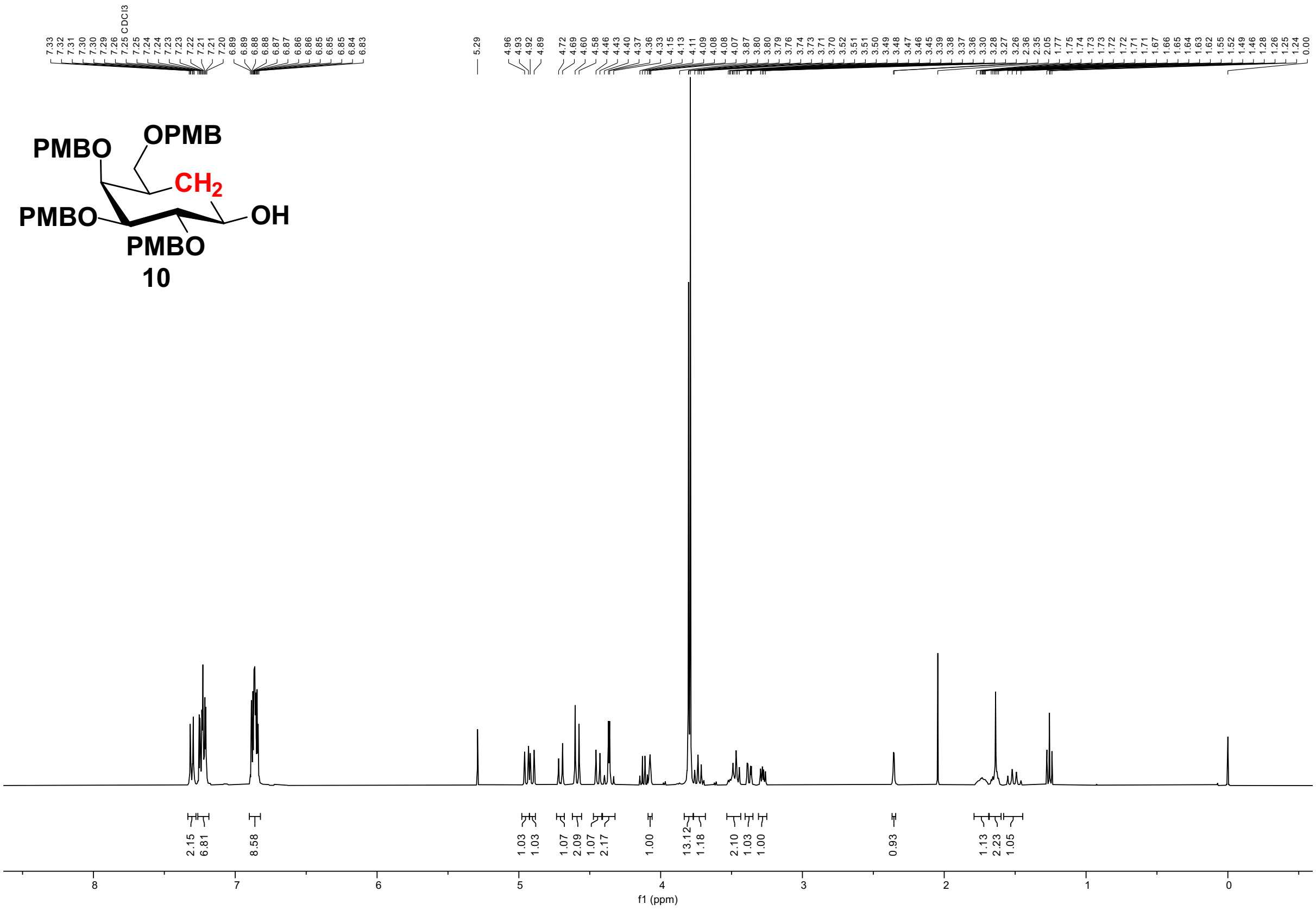

^{1}H NMR (400 MHz, $CDCl_3$) of compound **10**

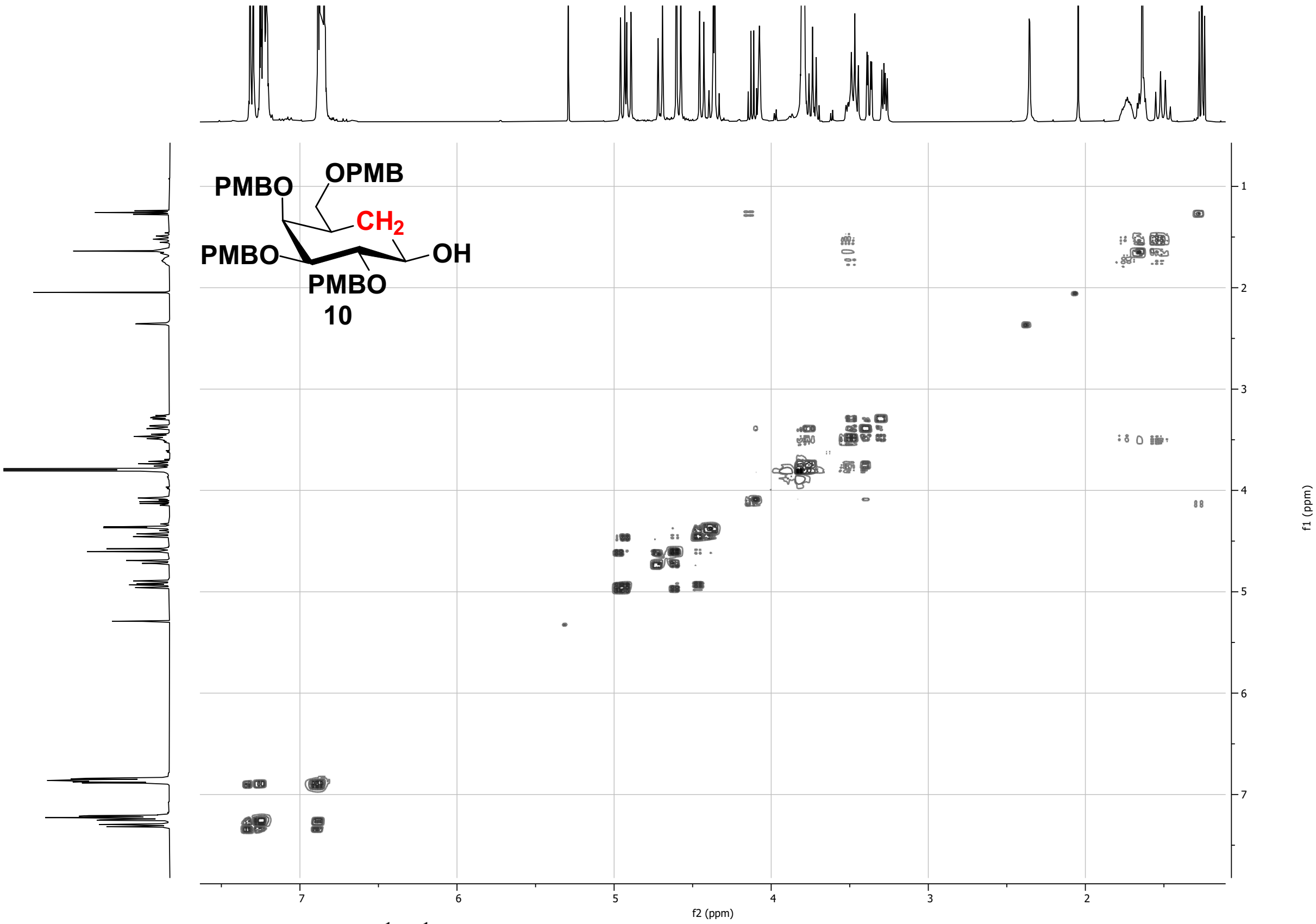

^{1}H-^{1}H COSY (400 MHz, $CDCl_3$) of compound **10**

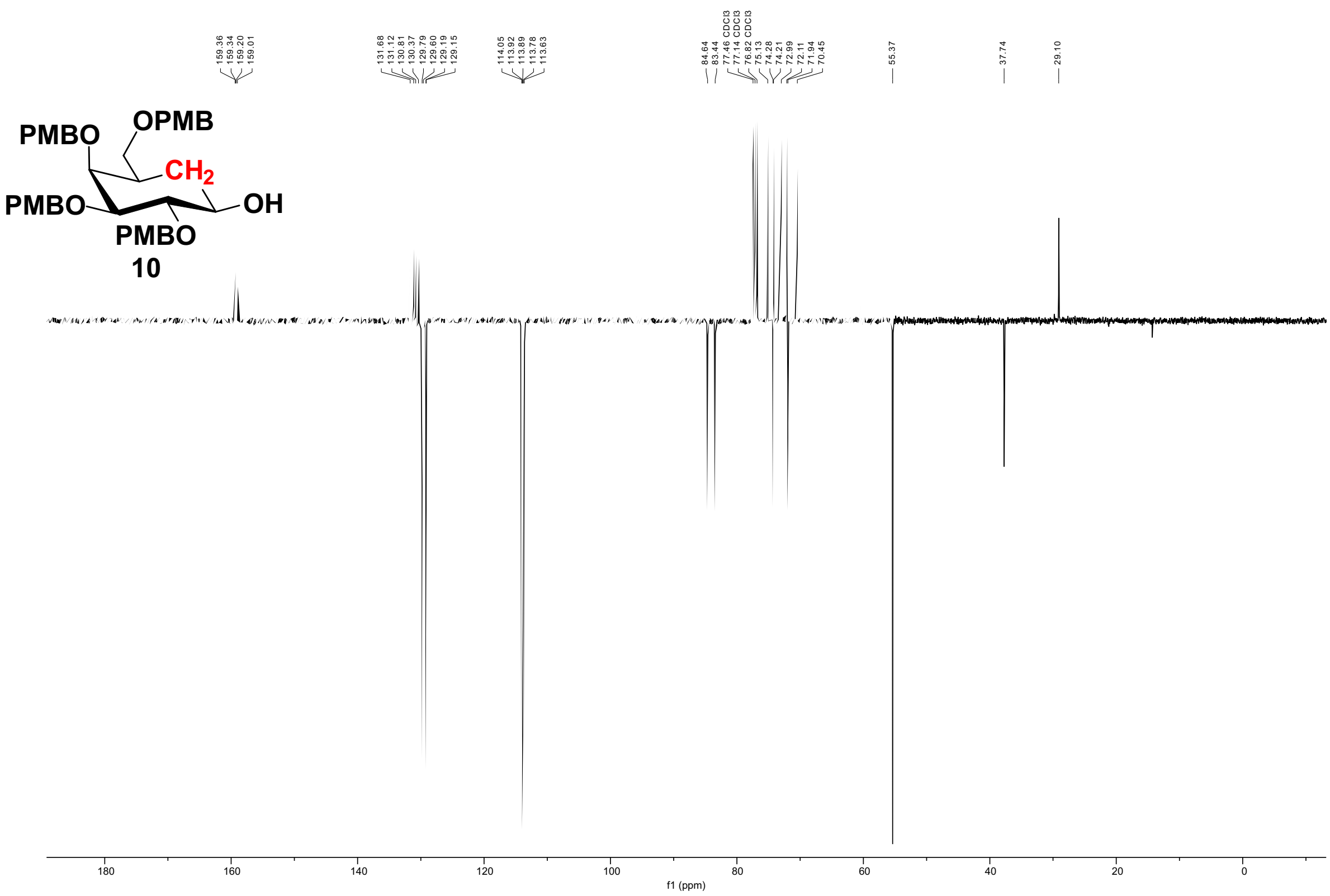

^{13}C NMR (101 MHz, $CDCl_3$) of compound **10**

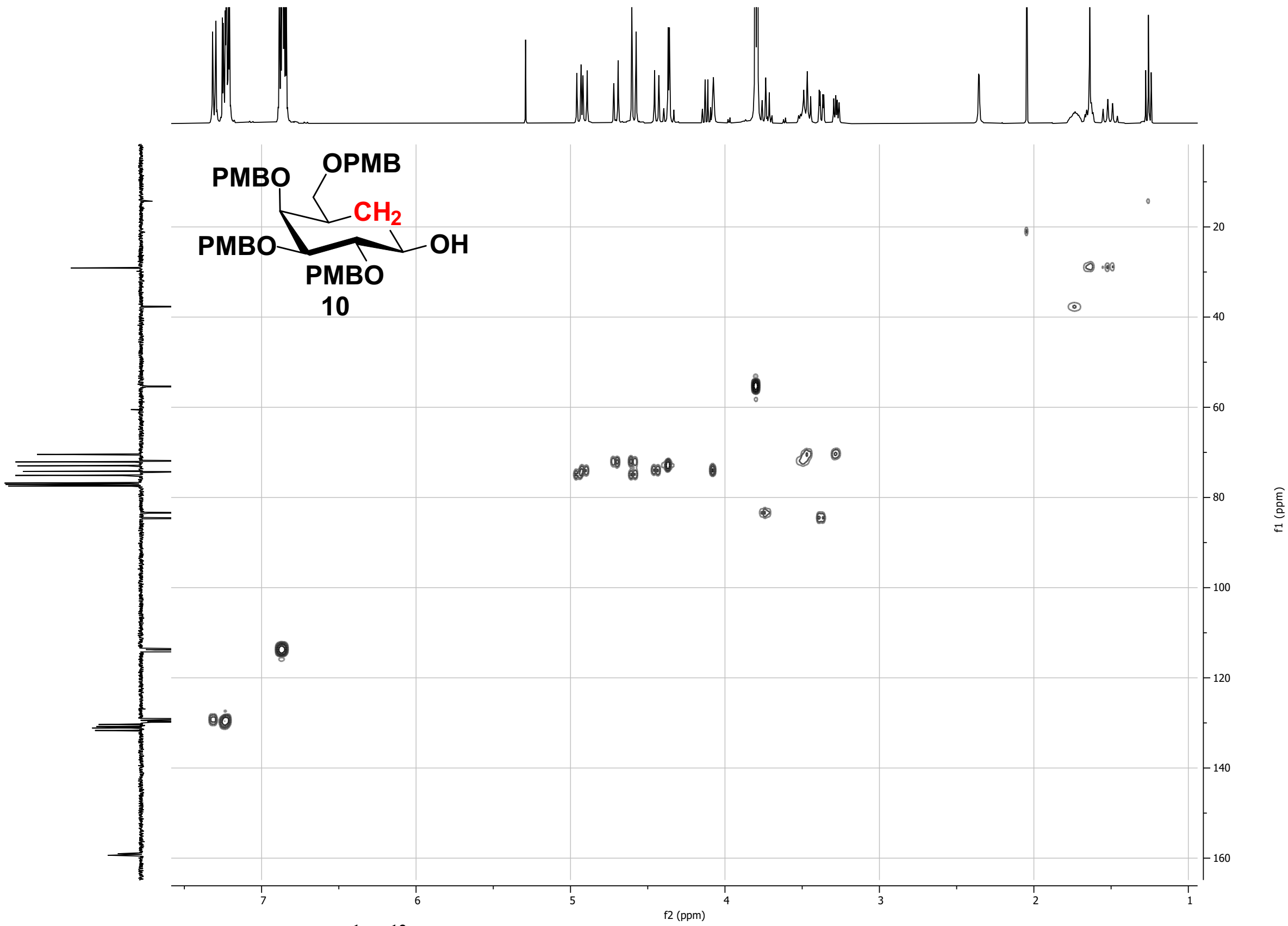

^{1}H-^{13}C HSQC (400 MHz, $CDCl_3$) of compound **10**

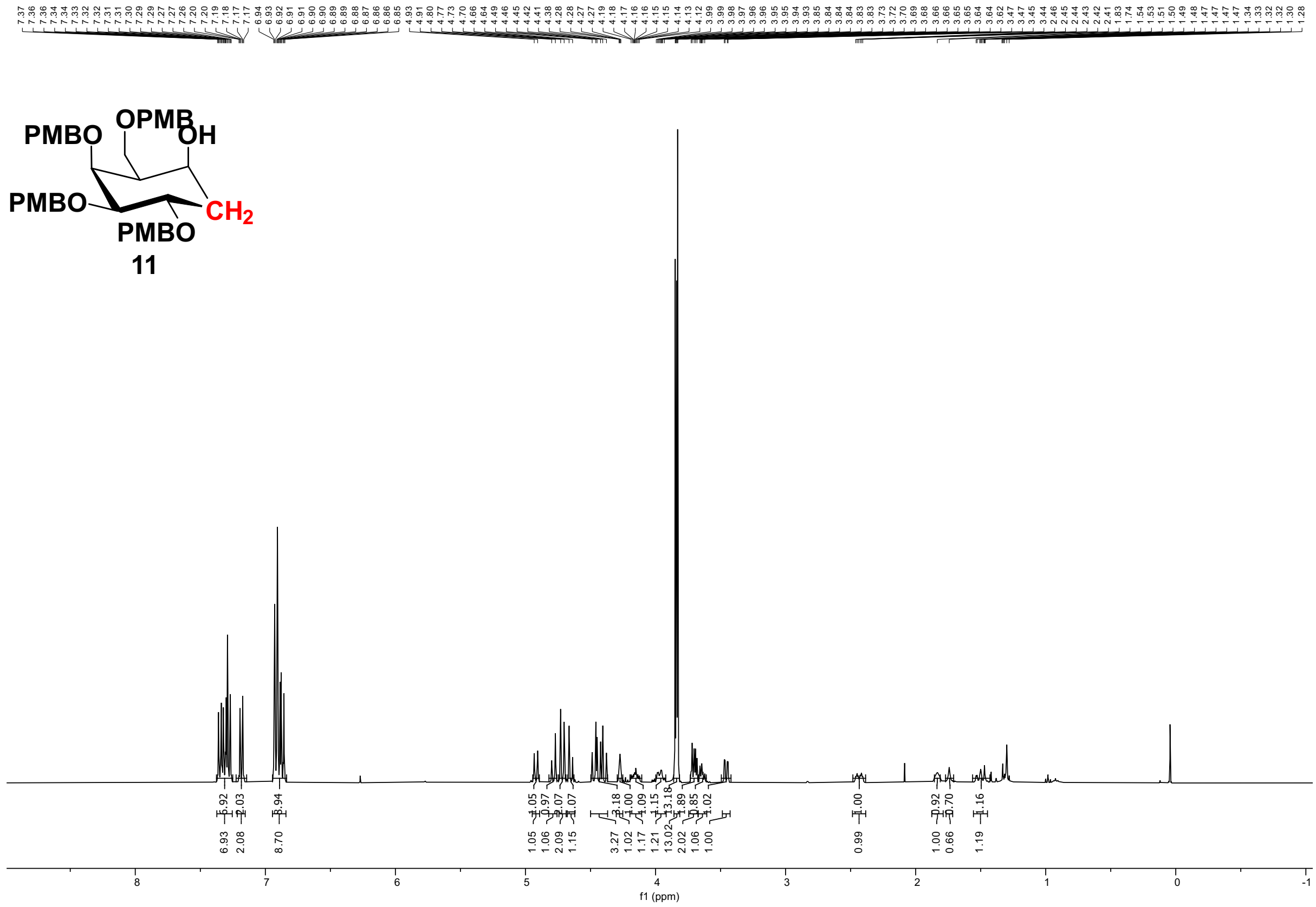

^1H NMR (400 MHz, $CDCl_3$) of compound **11**

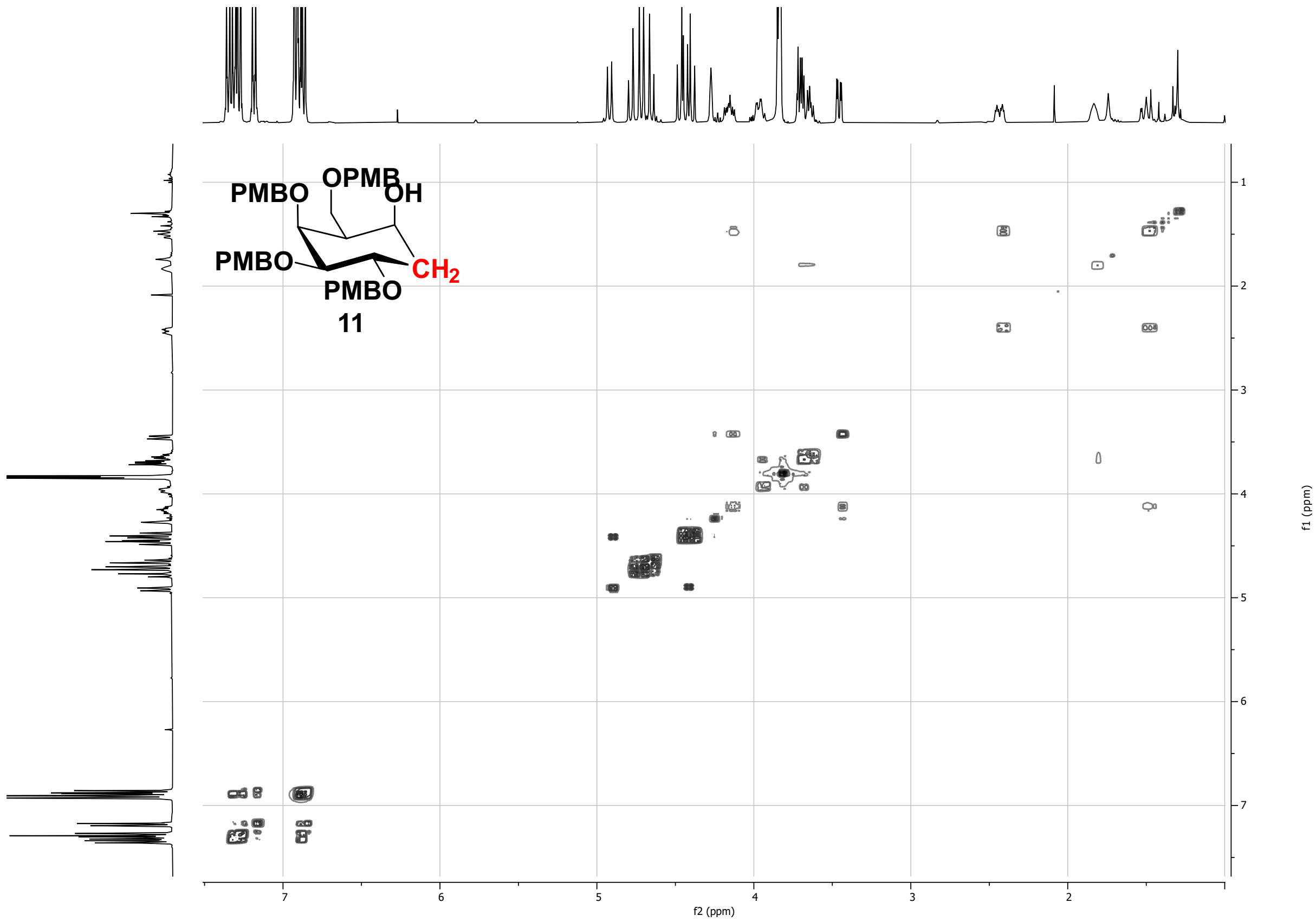

^1H-^1H COSY (400 MHz, $CDCl_3$) of compound **11**

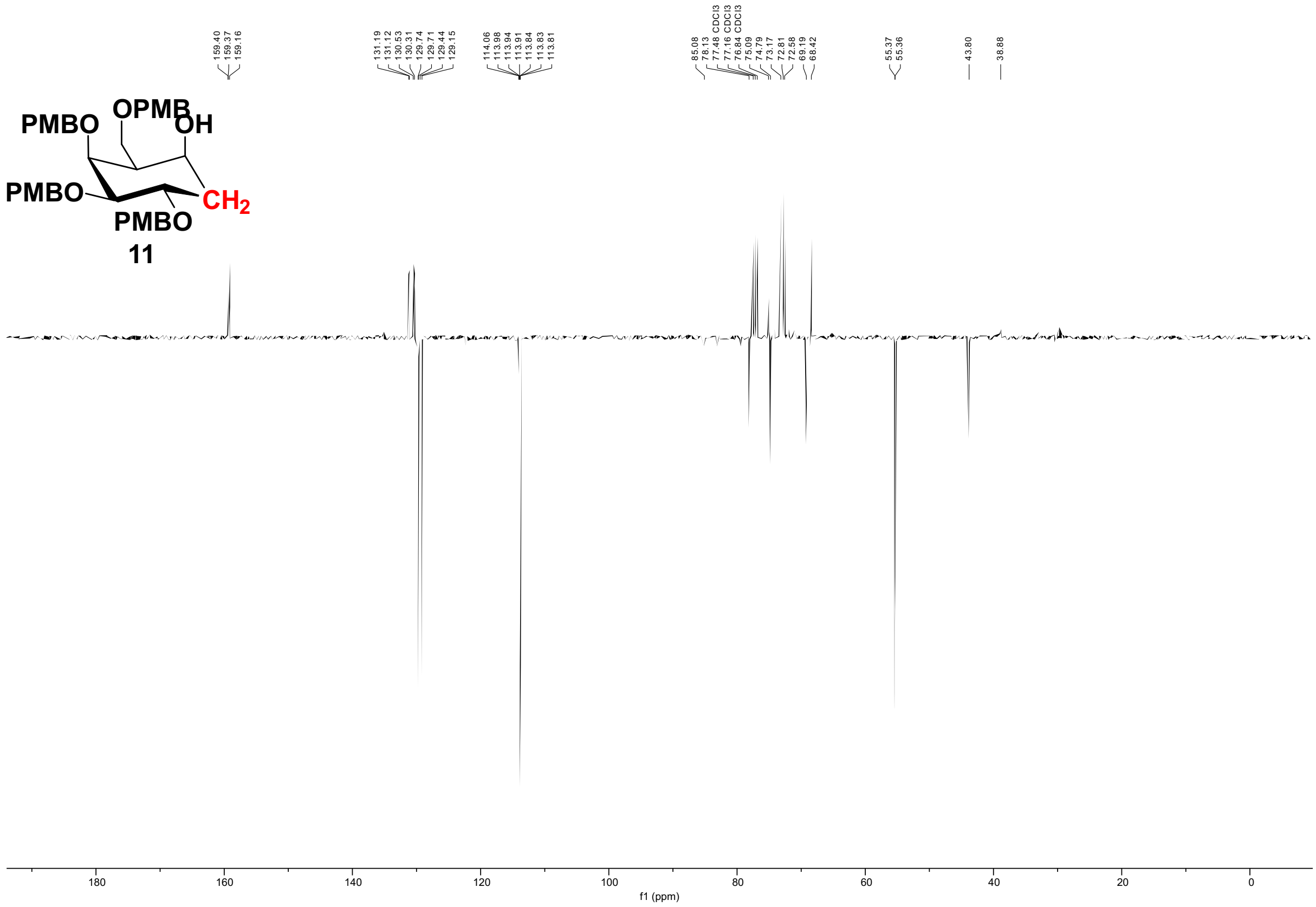

^{13}C NMR (101 MHz, CDCl$_3$) of compound **11**

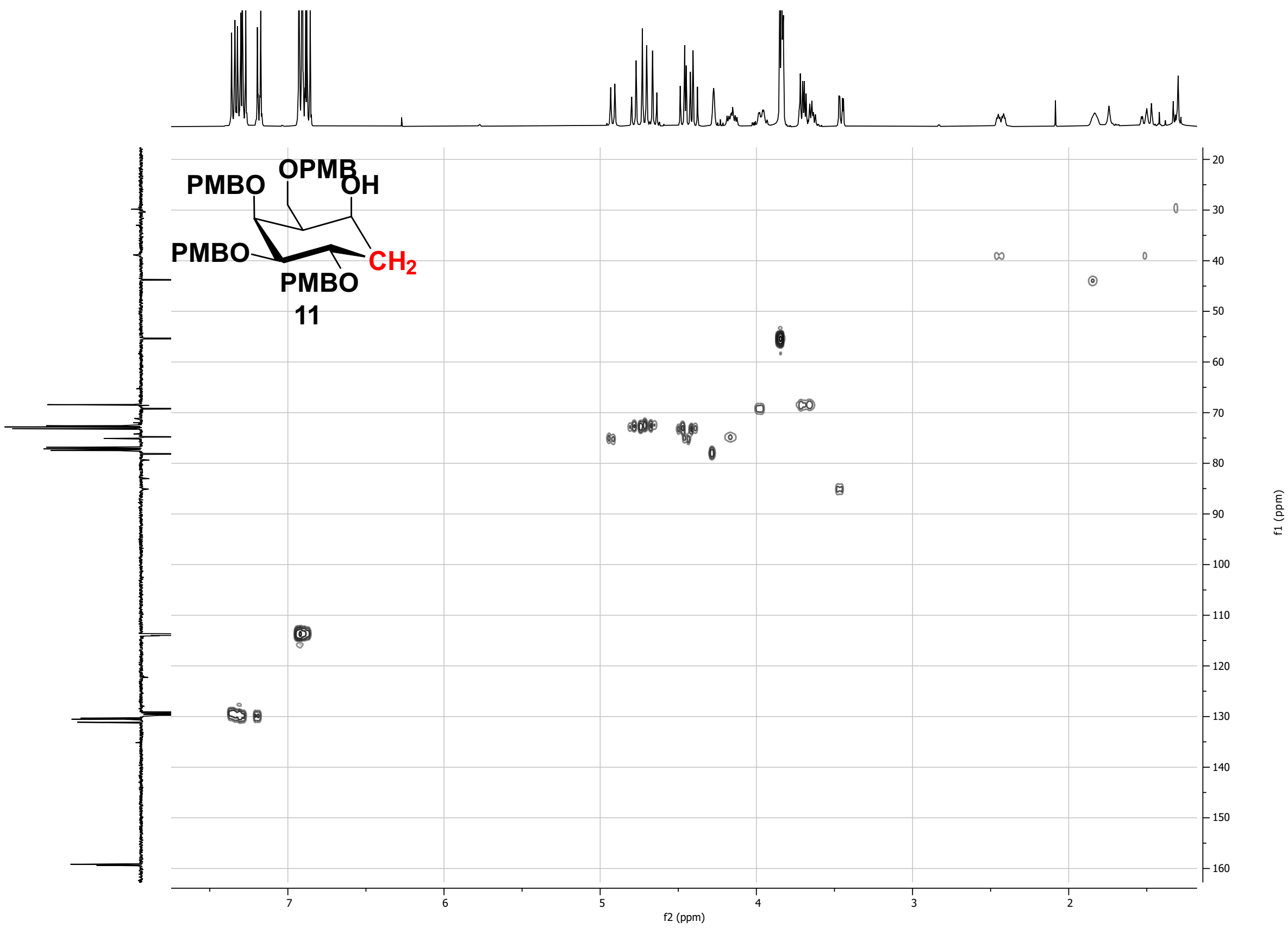

^{1}H-^{13}C HSQC (400 MHz, CDCl$_3$) of compound **11**

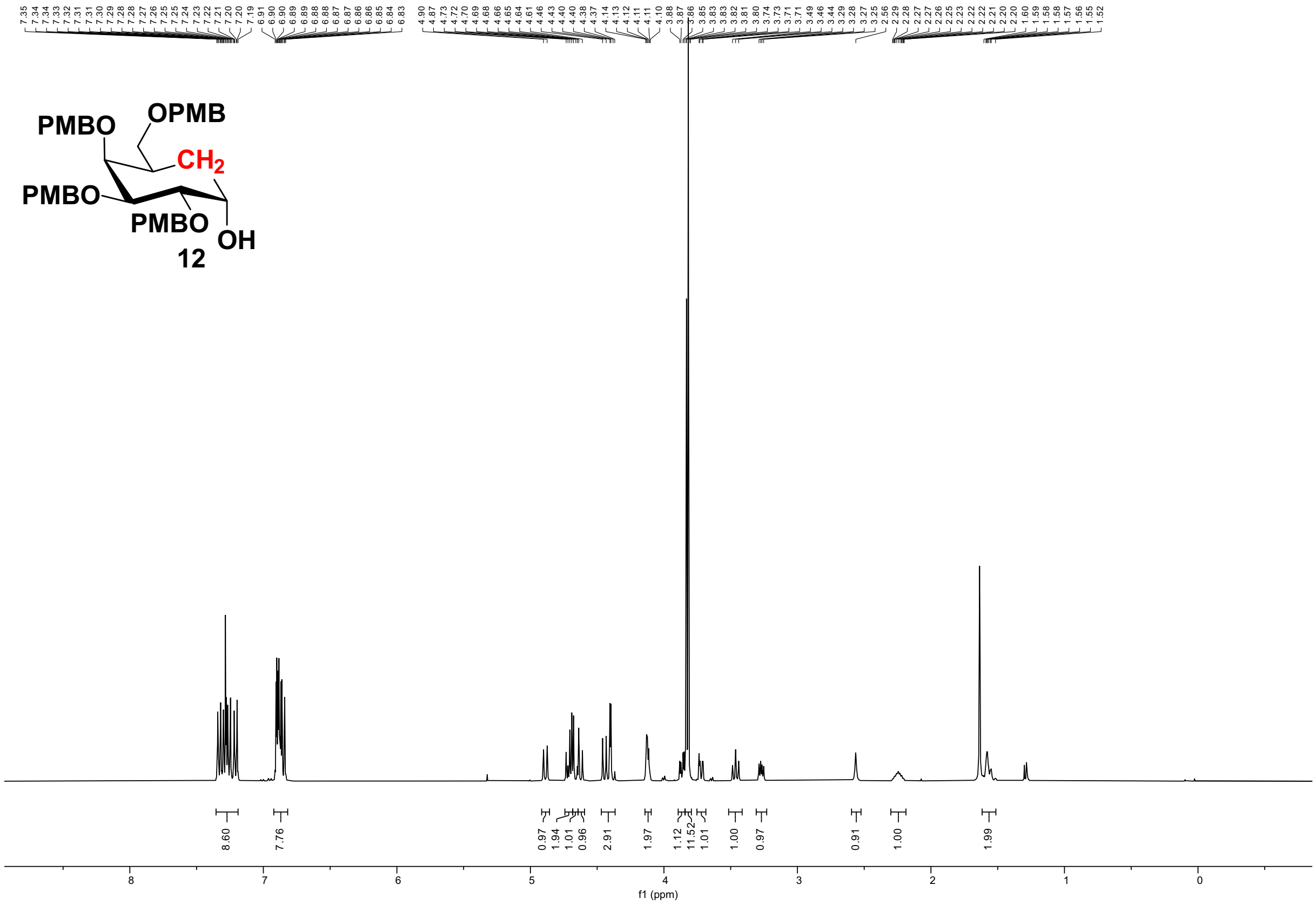

^{1}H NMR (400 MHz, $CDCl_3$) of compound **12**

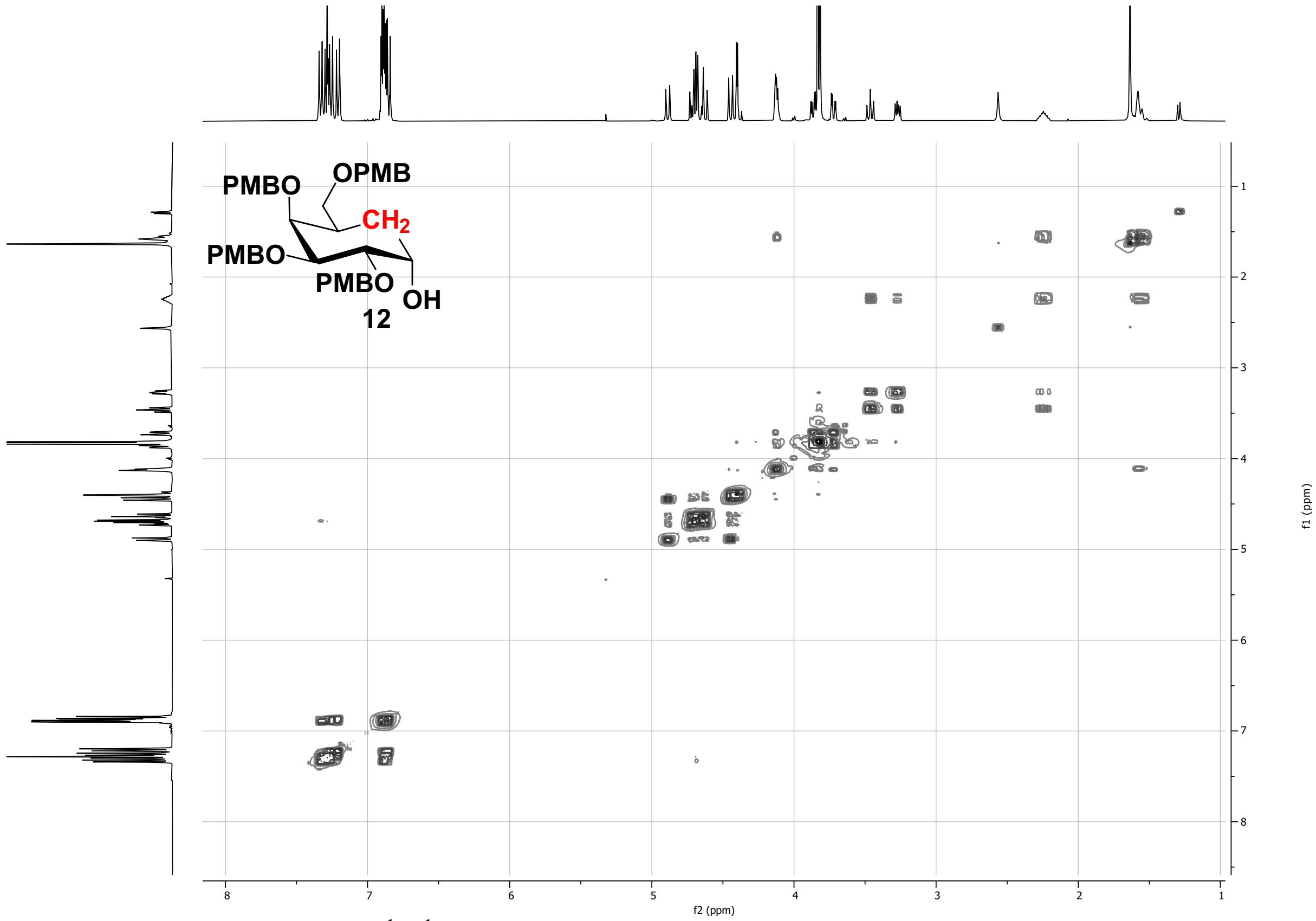

^{1}H-^{1}H COSY (400 MHz, $CDCl_3$) of compound **12**

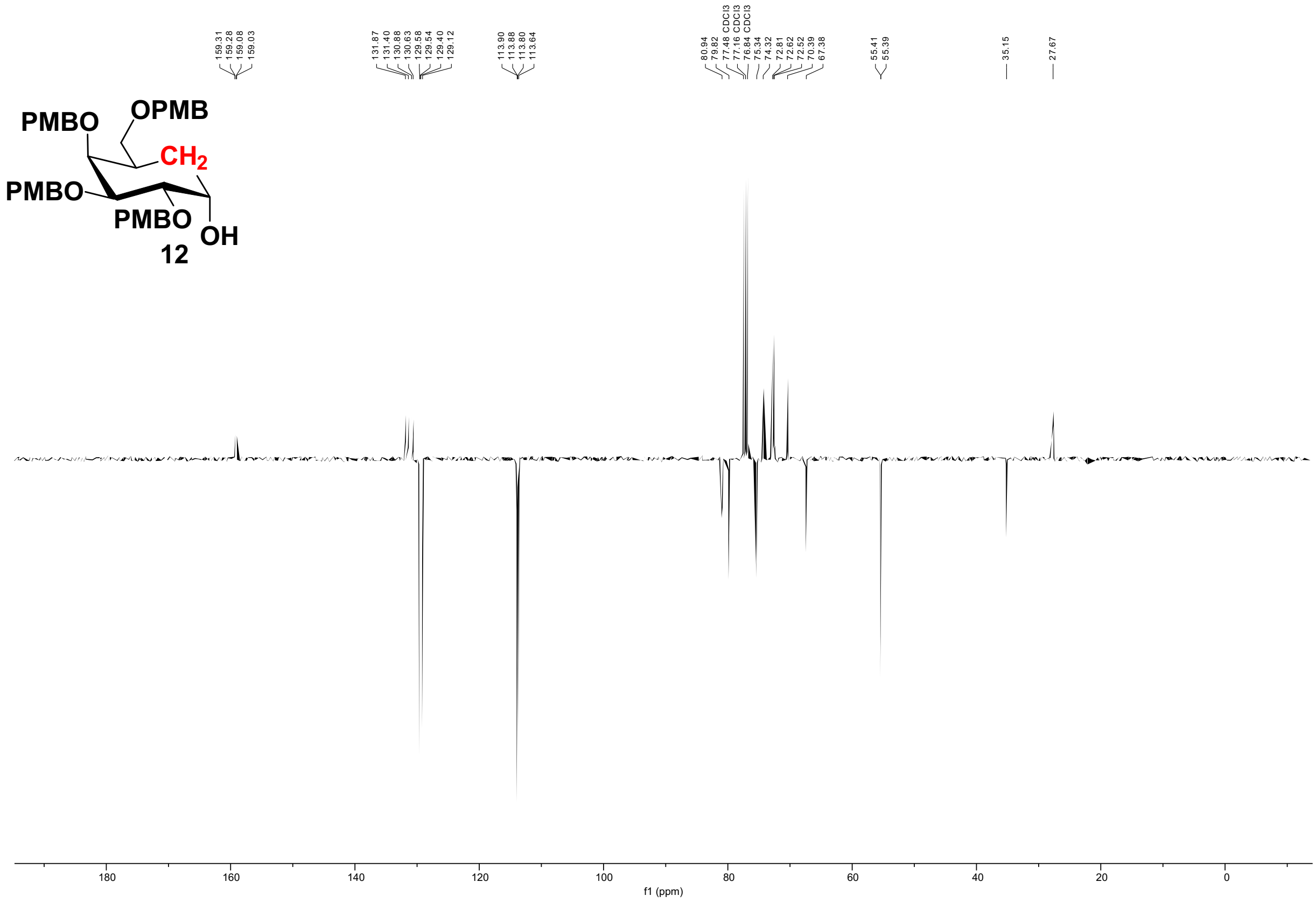

^{13}C NMR (101 MHz, $CDCl_3$) of compound **12**

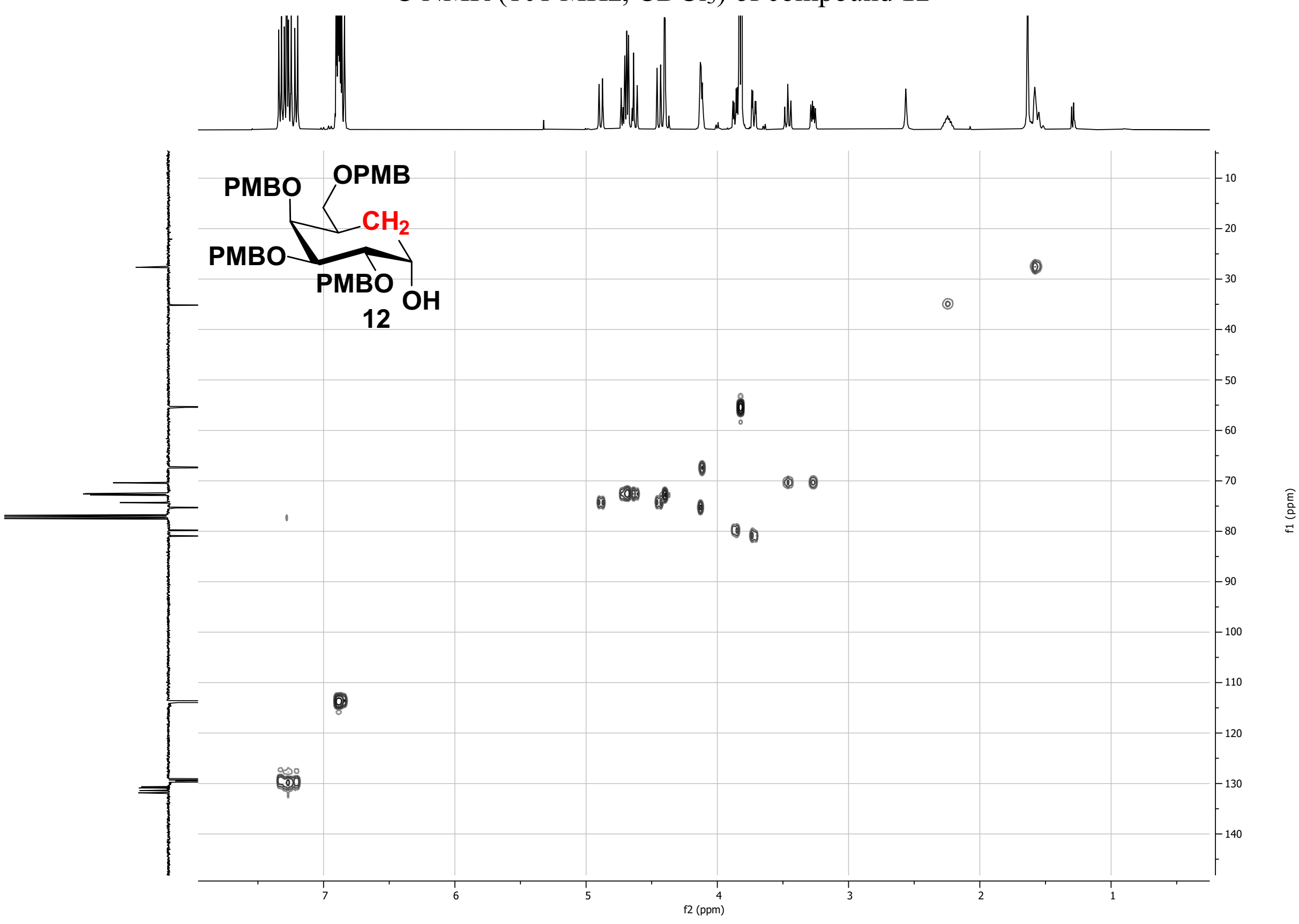

^{1}H-^{13}C HSQC (400 MHz, $CDCl_3$) of compound **12**

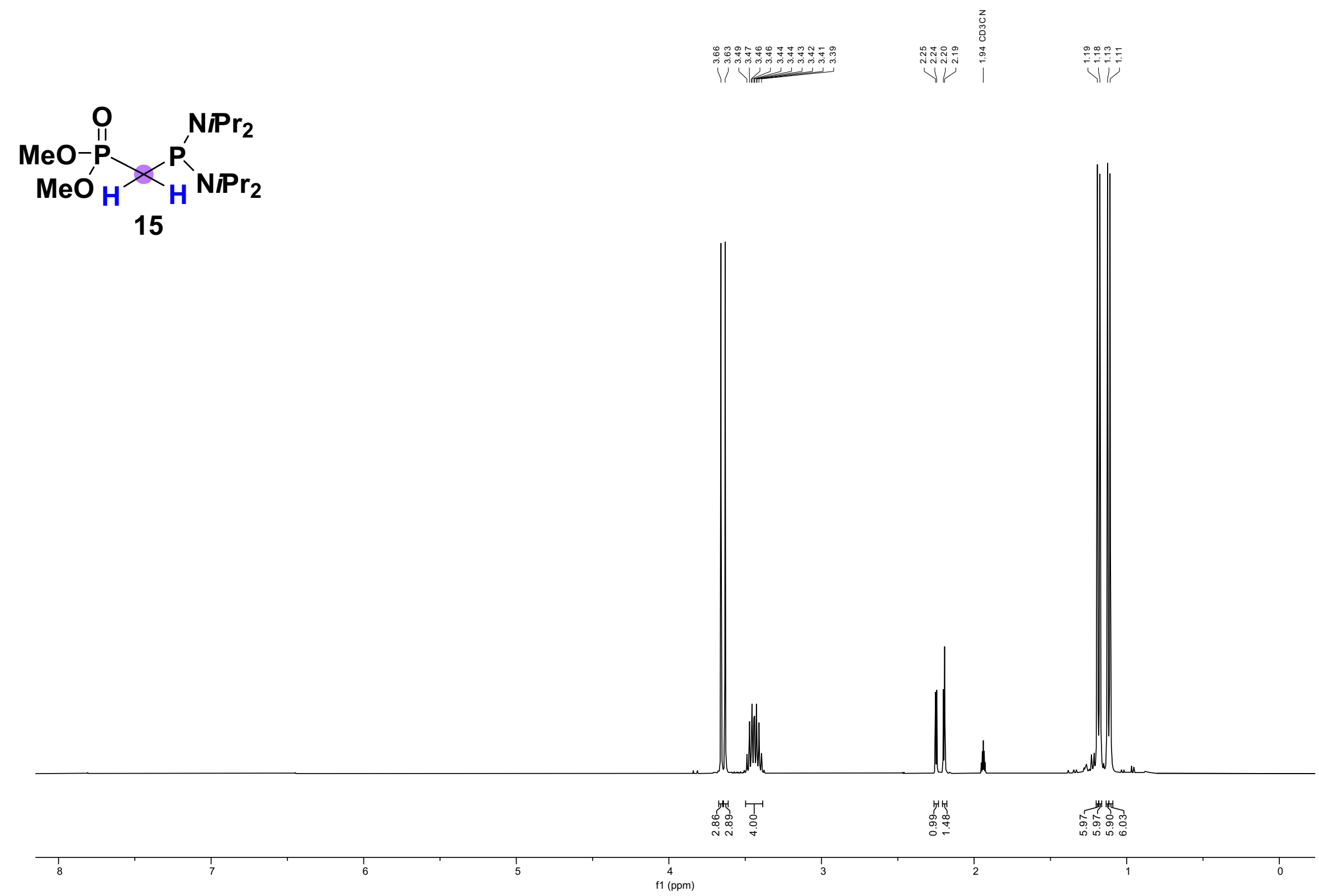

^{1}H NMR (400 MHz, $CDCl_3$) of compound **15**

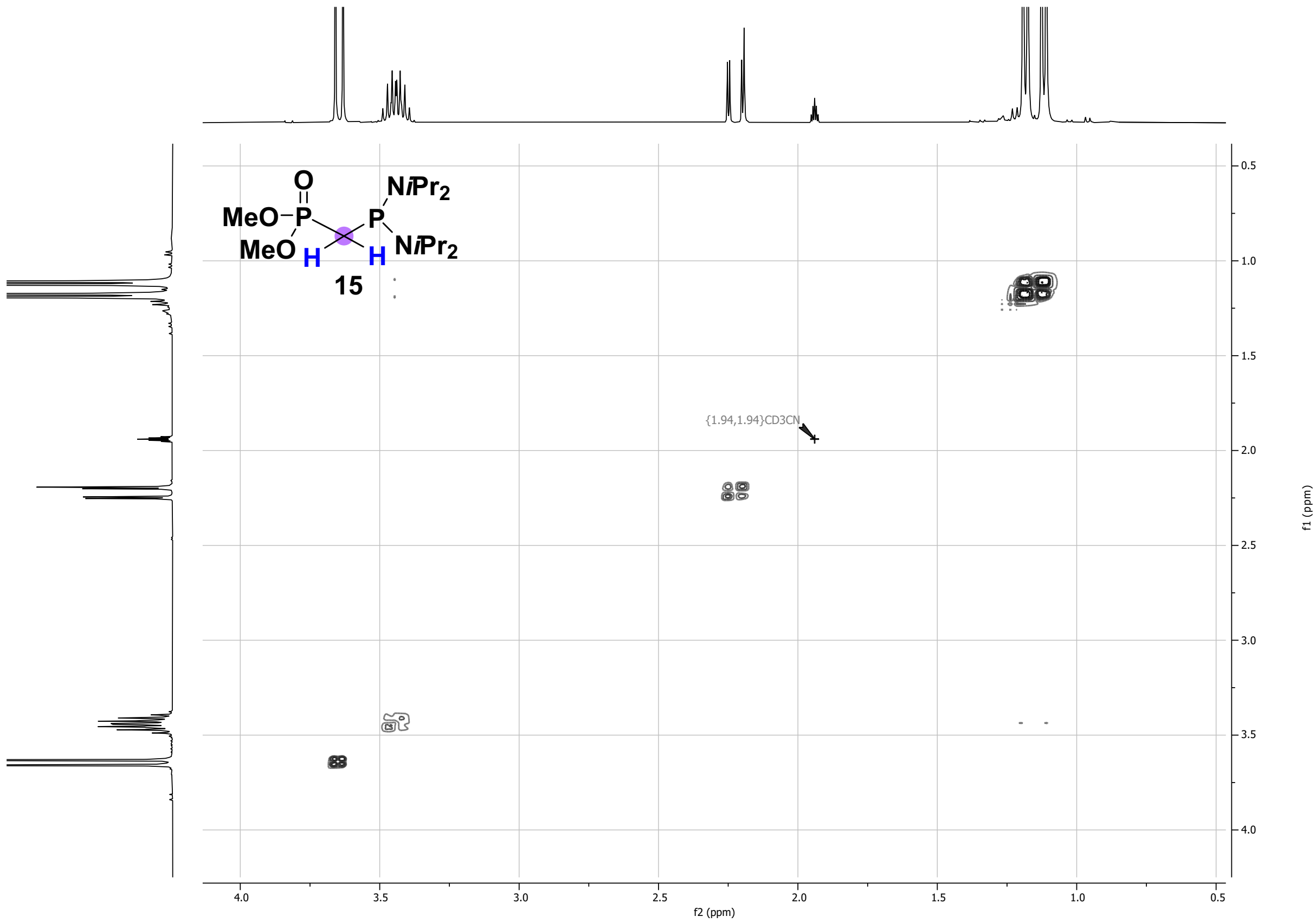

^{1}H-^{1}H COSY (400 MHz, $CDCl_3$) of compound **15**

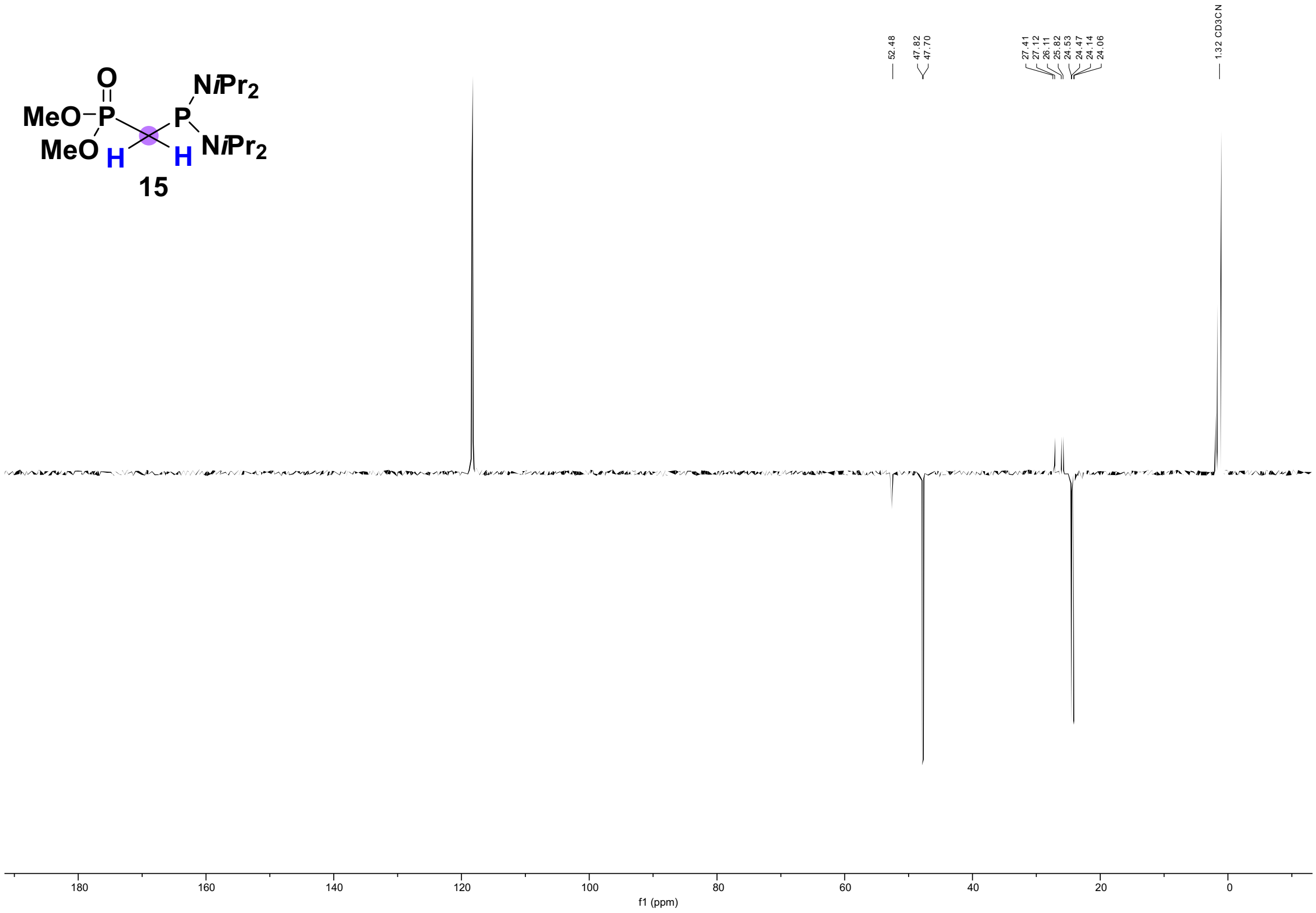

^{13}C NMR (101 MHz, $CDCl_3$) of compound **15**

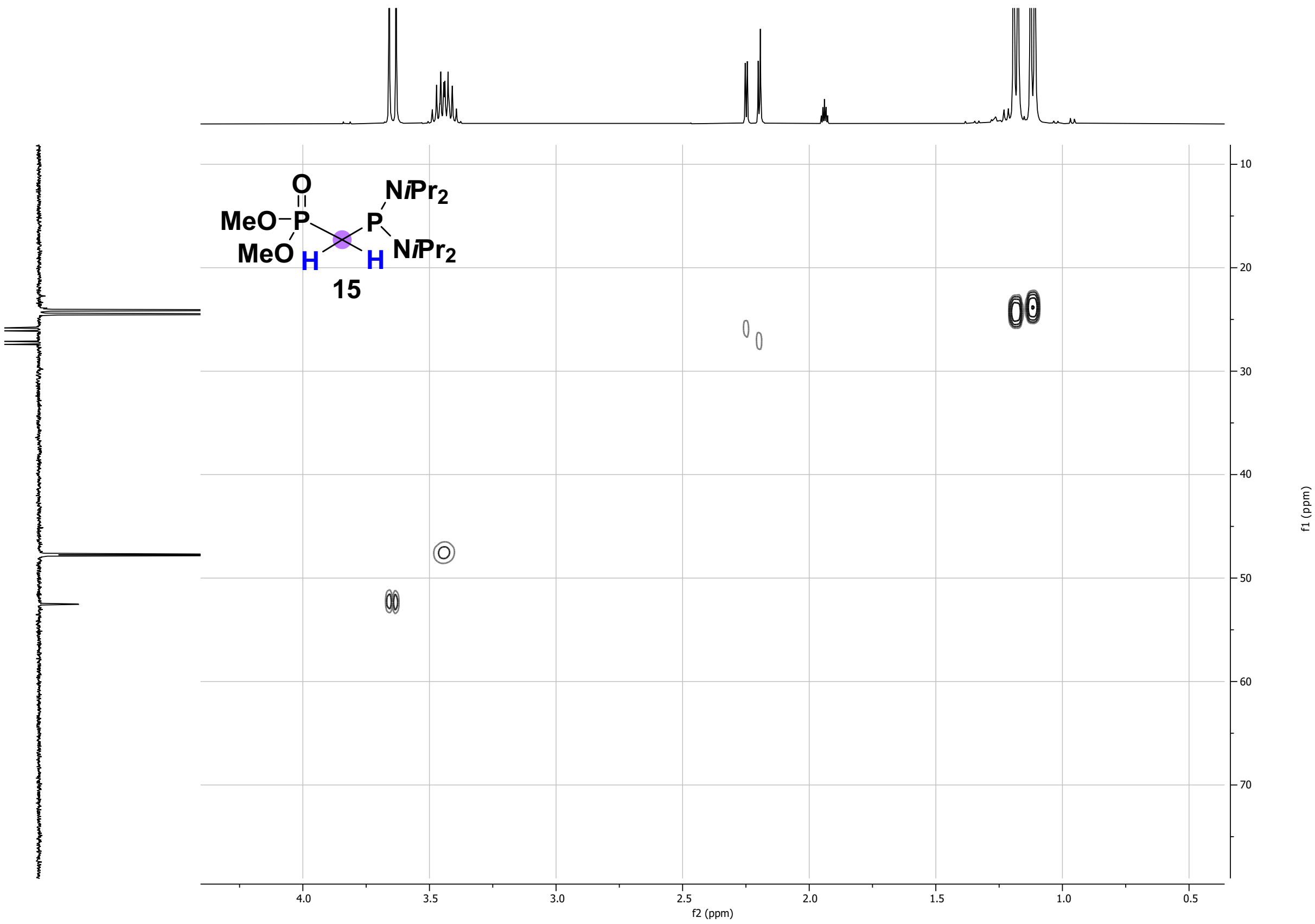

^{1}H-^{13}C HSQC (400 MHz, $CDCl_3$) of compound **15**

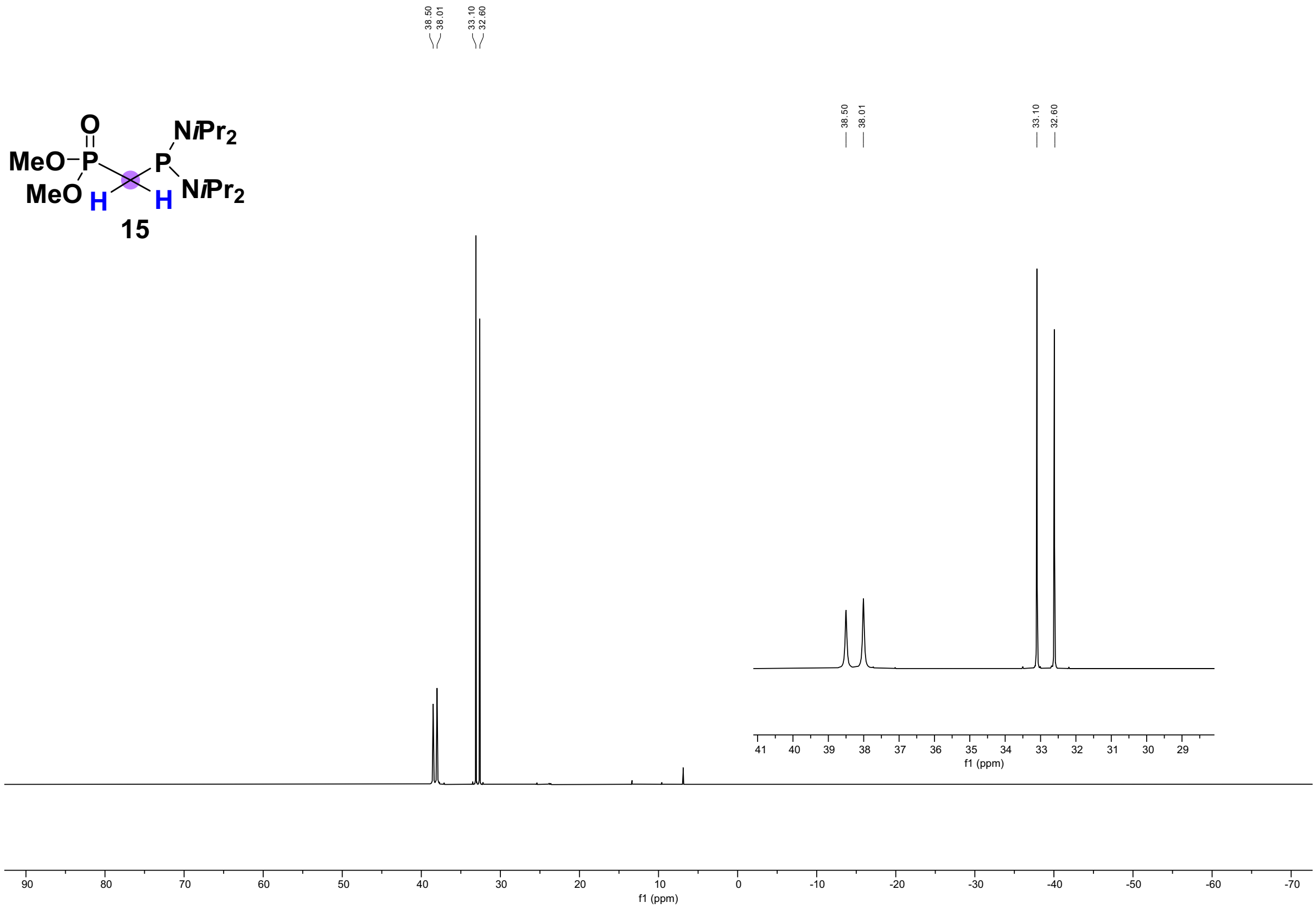

^{31}P NMR (162 MHz, CDCl3) of compound **15**

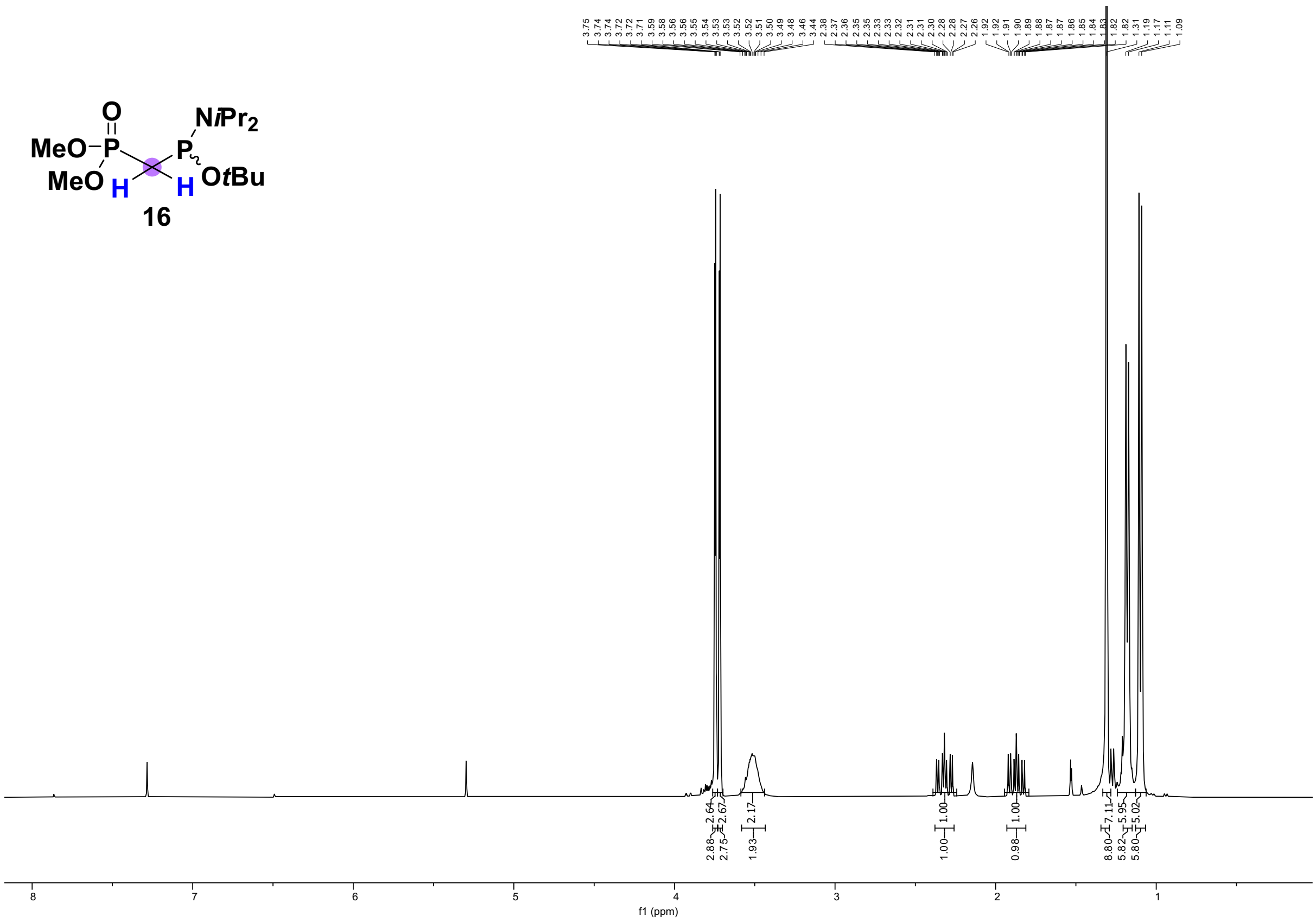

^{1}H NMR (400 MHz, $CDCl_3$) of compound **16**

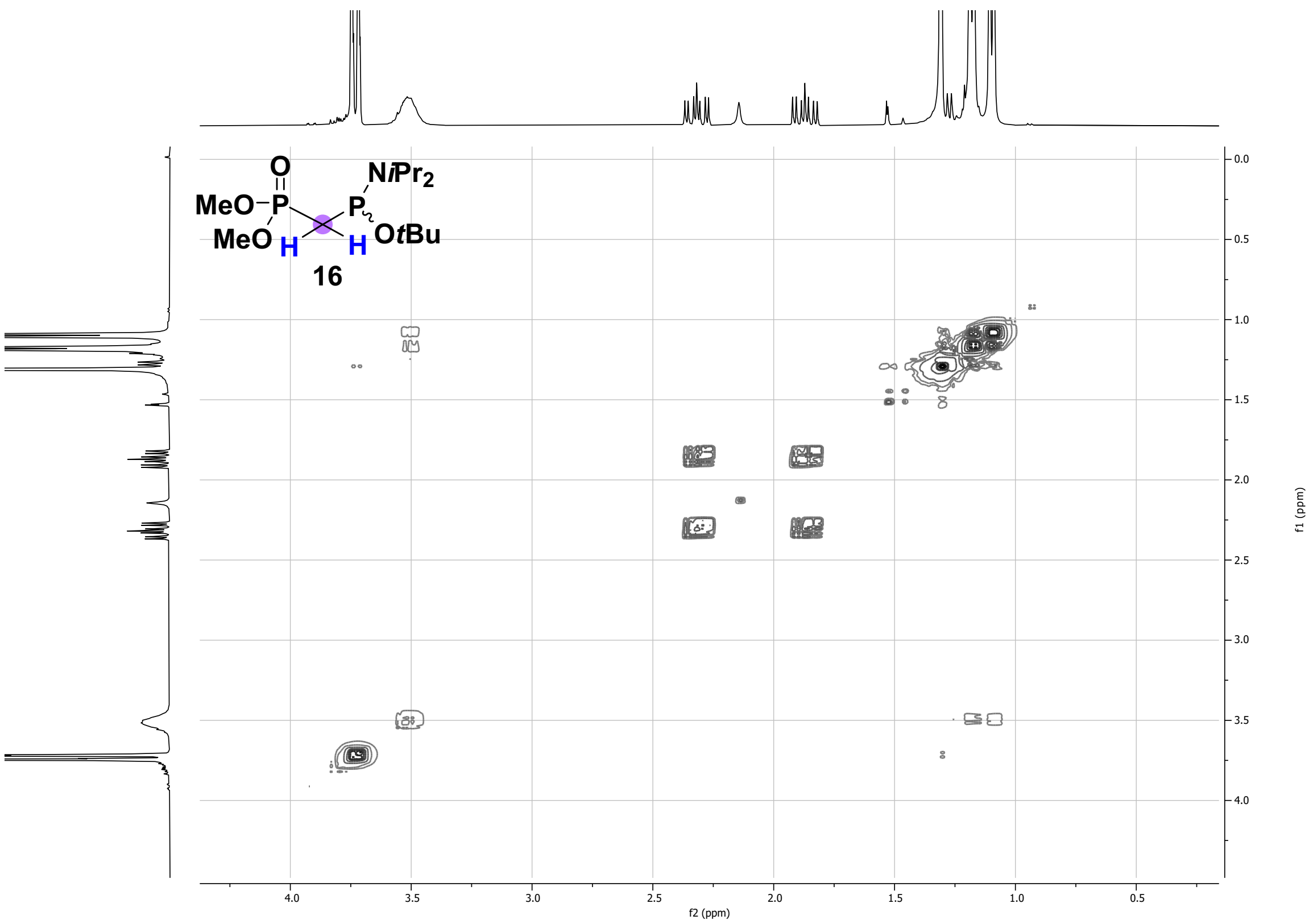

^{1}H-^{1}H COSY (400 MHz, $CDCl_3$) of compound **16**

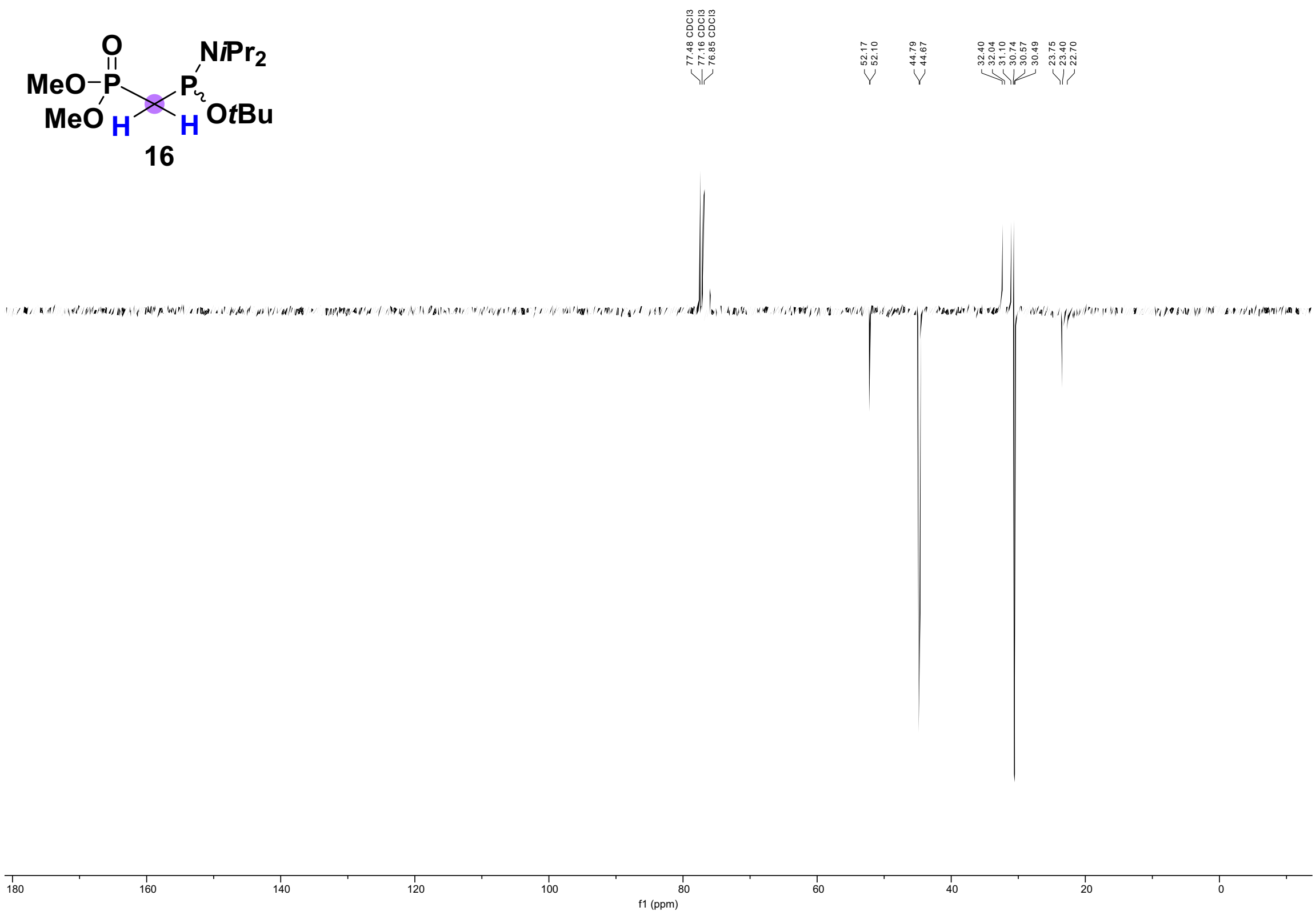

^{13}C NMR (101 MHz, $CDCl_3$) of compound **16**

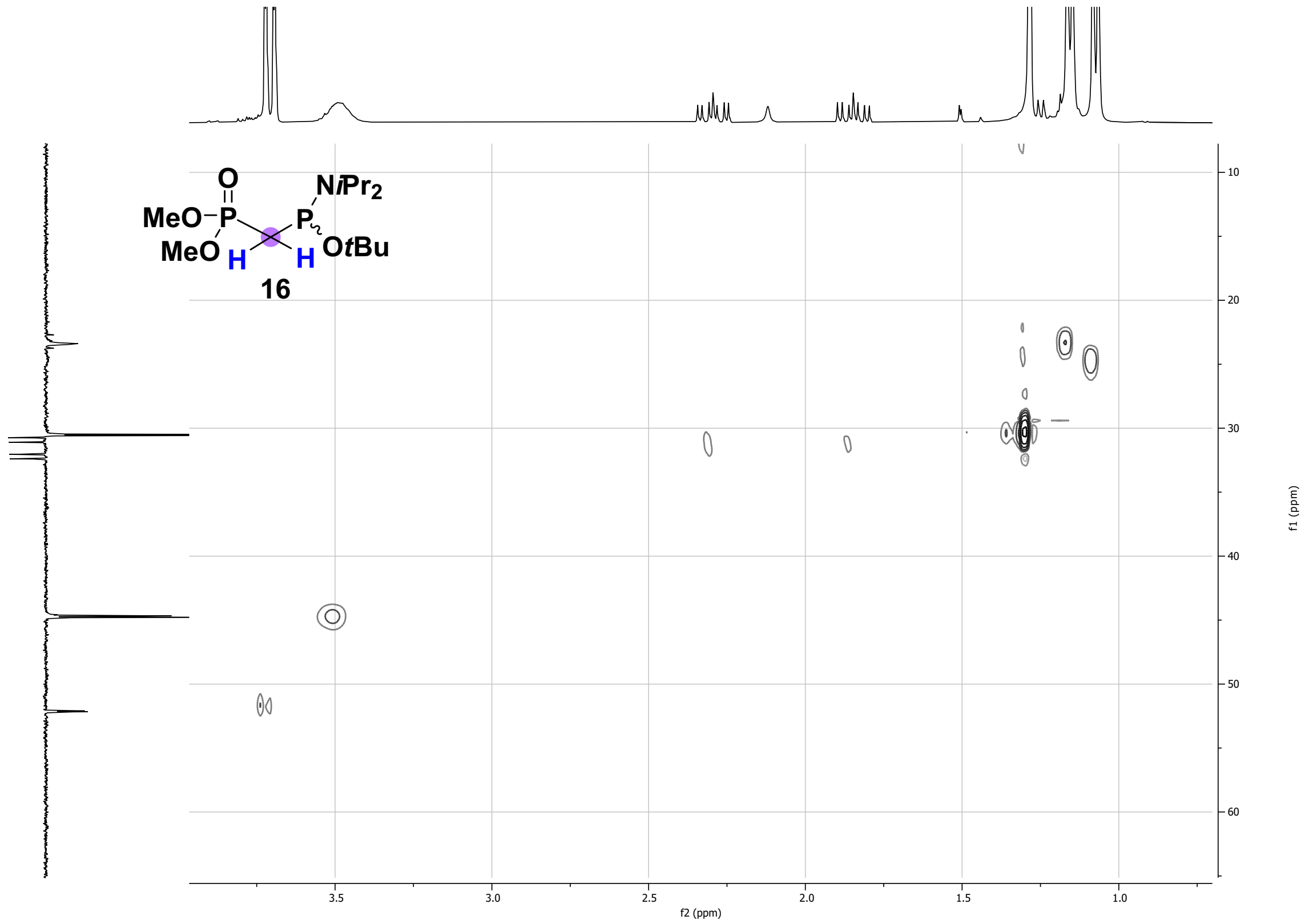

^{1}H-^{13}C HSQC (400 MHz, $CDCl_3$) of compound **16**

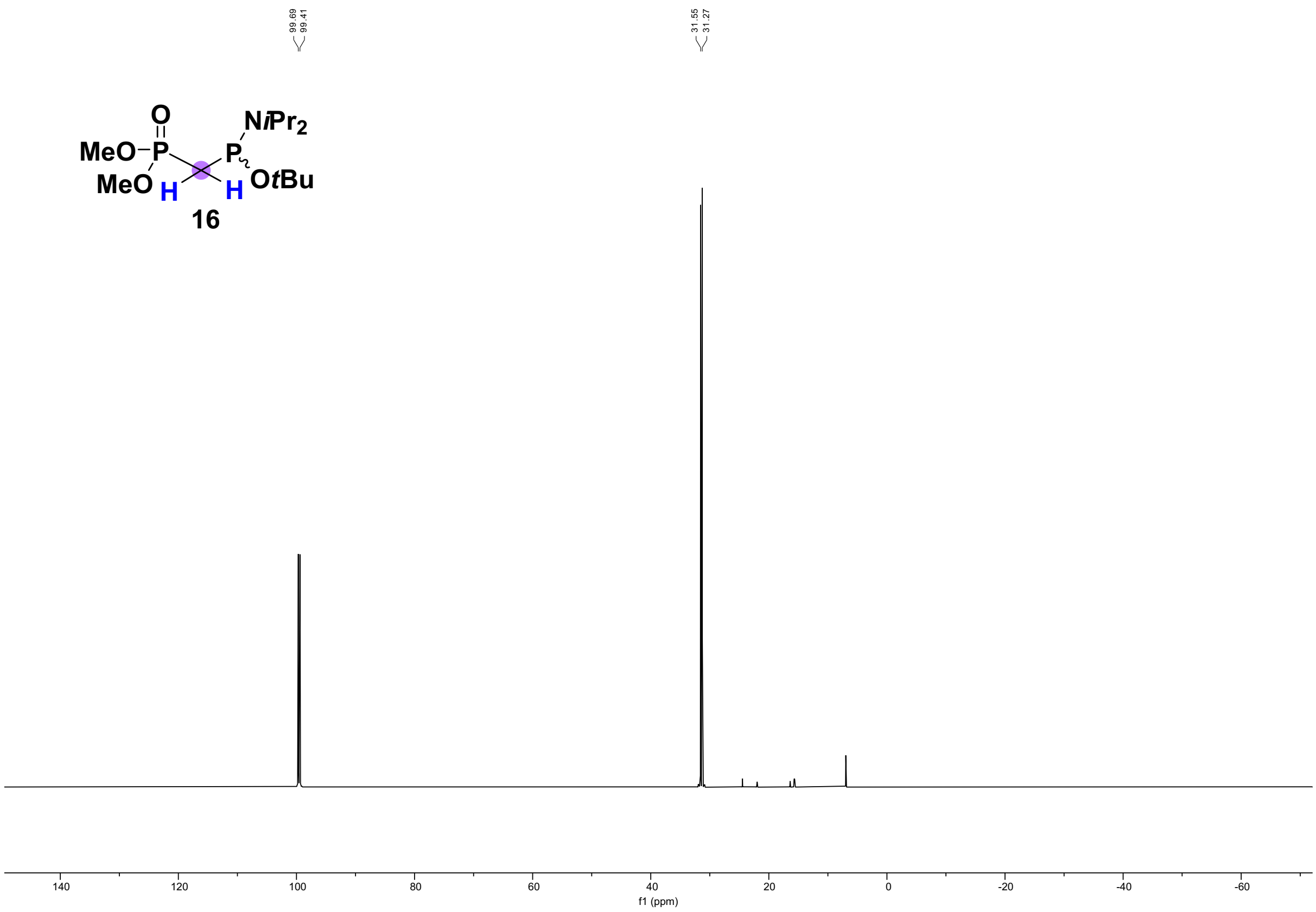

^{31}P NMR (162 MHz, CDCl3) of compound **16**

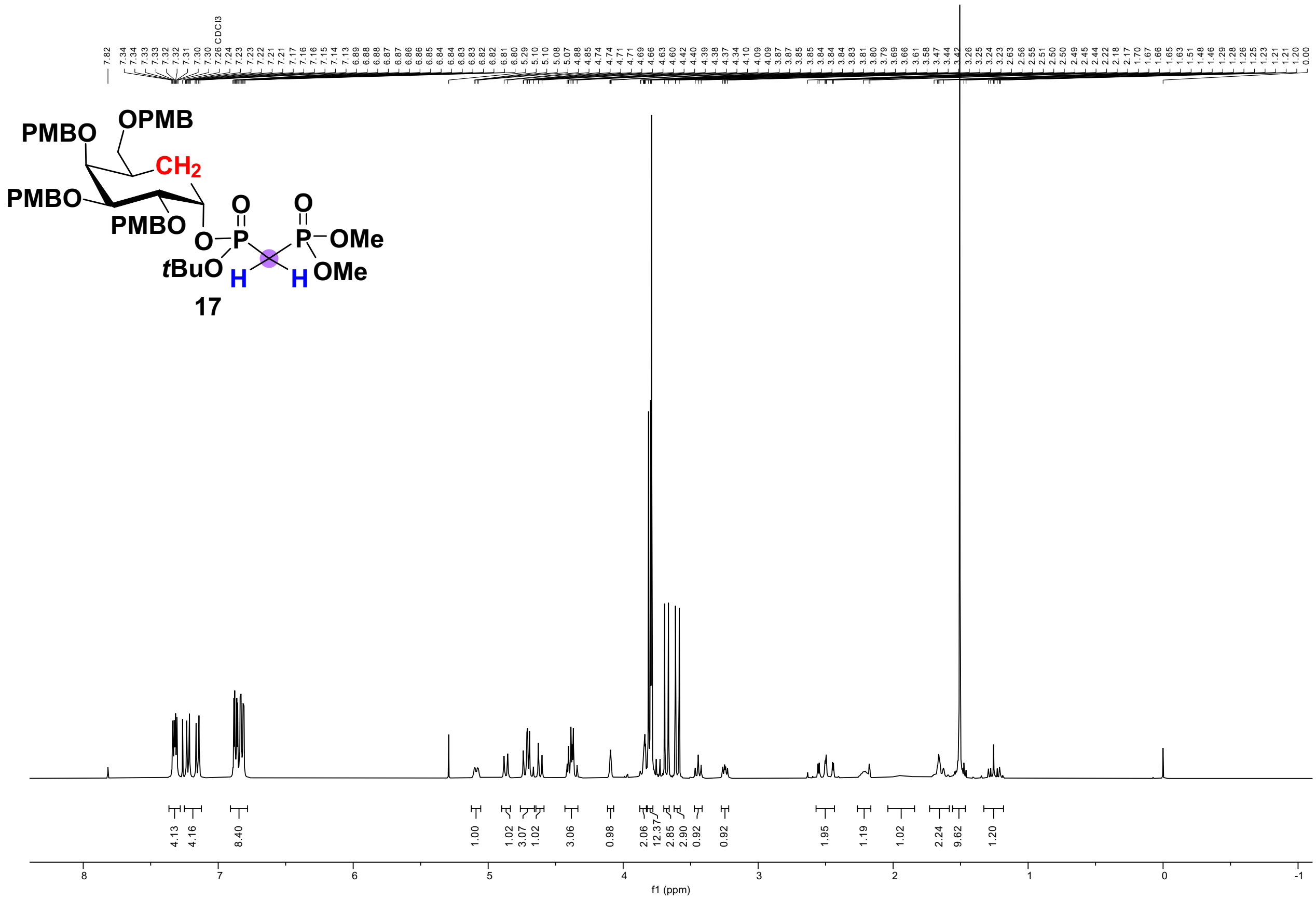

^{1}H NMR (400 MHz, CDCl$_3$) of compound **17**

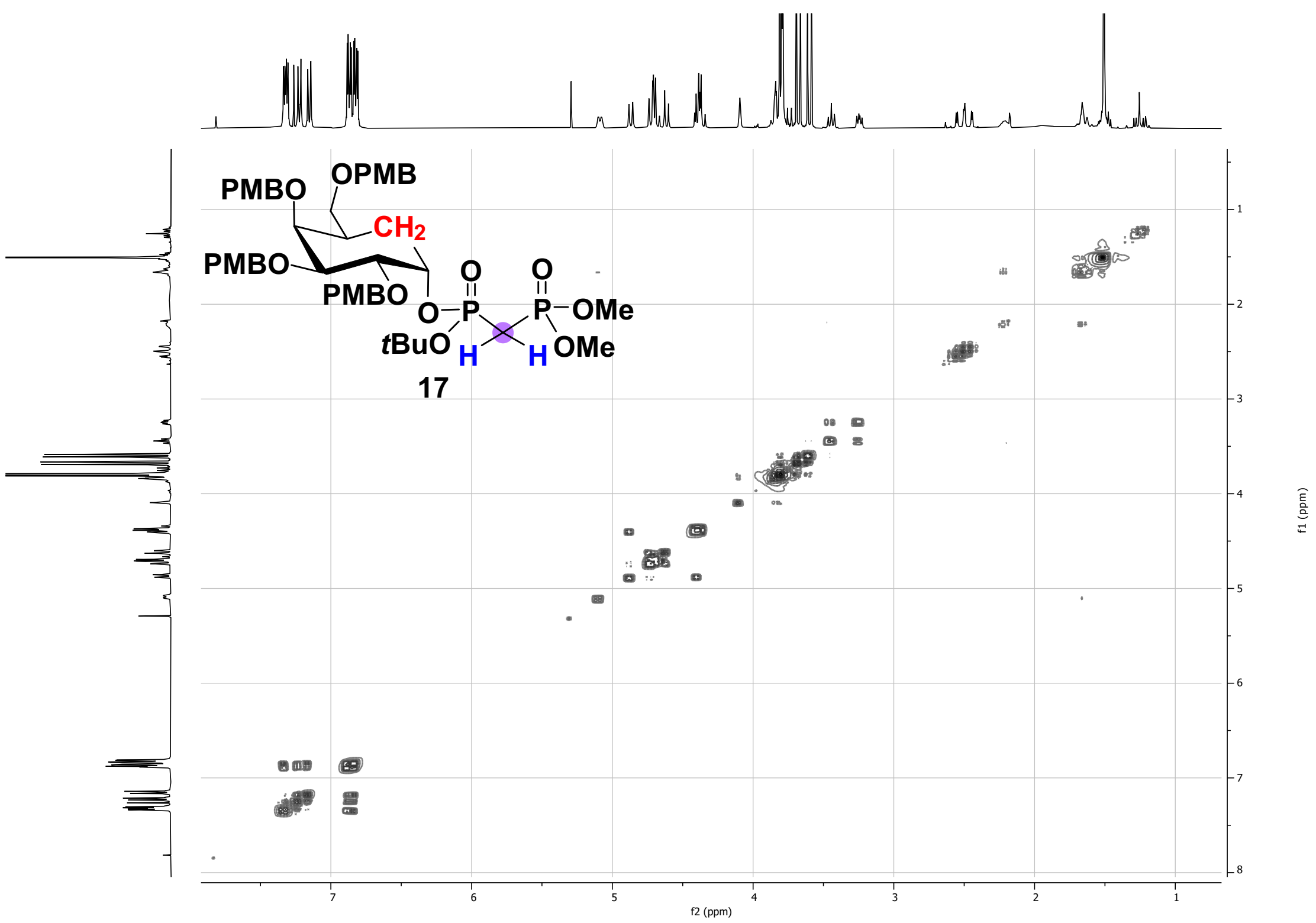

^{1}H-^{1}H COSY (400 MHz, CDCl$_3$) of compound **17**

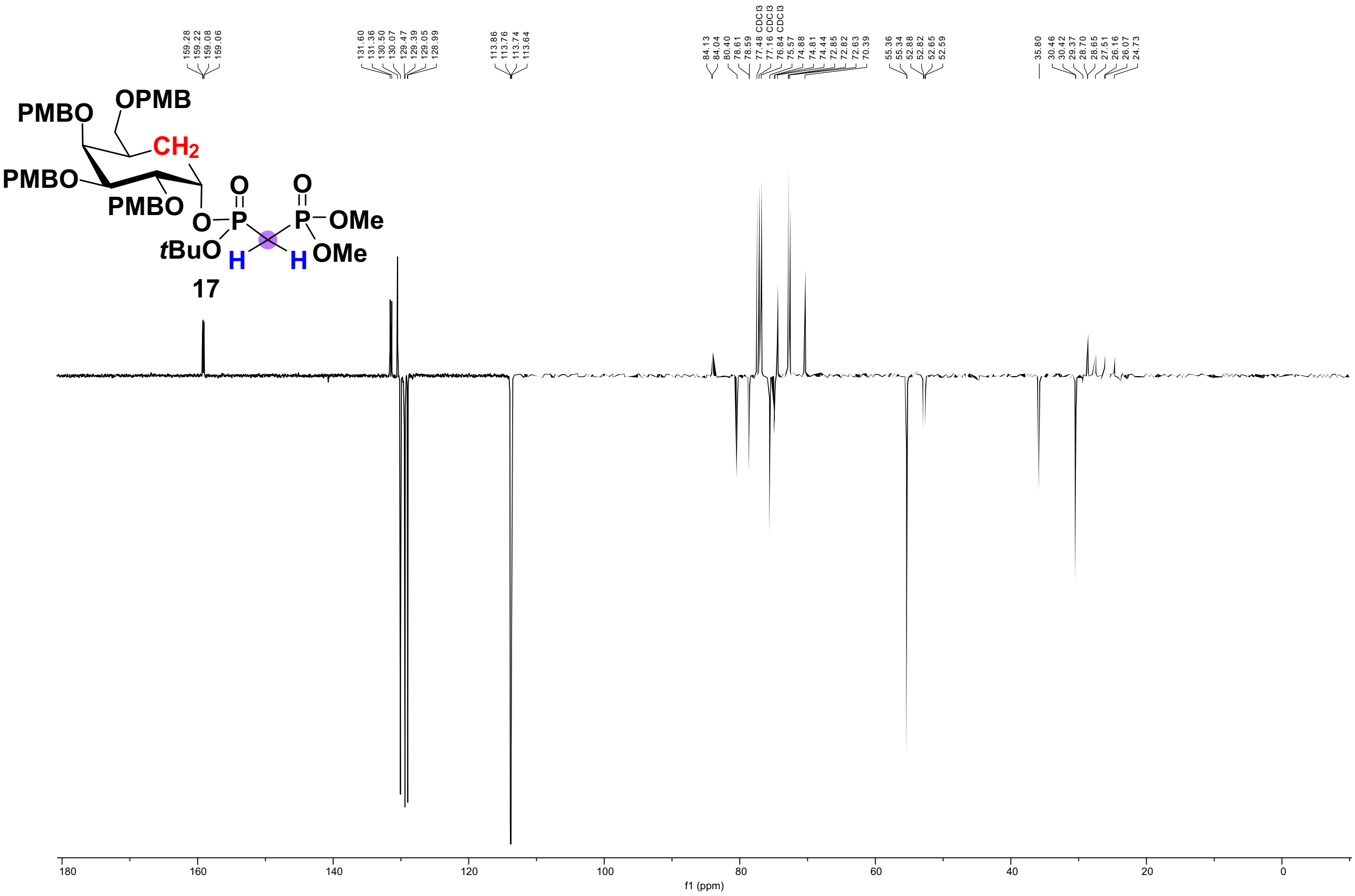

^{13}C NMR (101 MHz, CDCl$_3$) of compound **17**

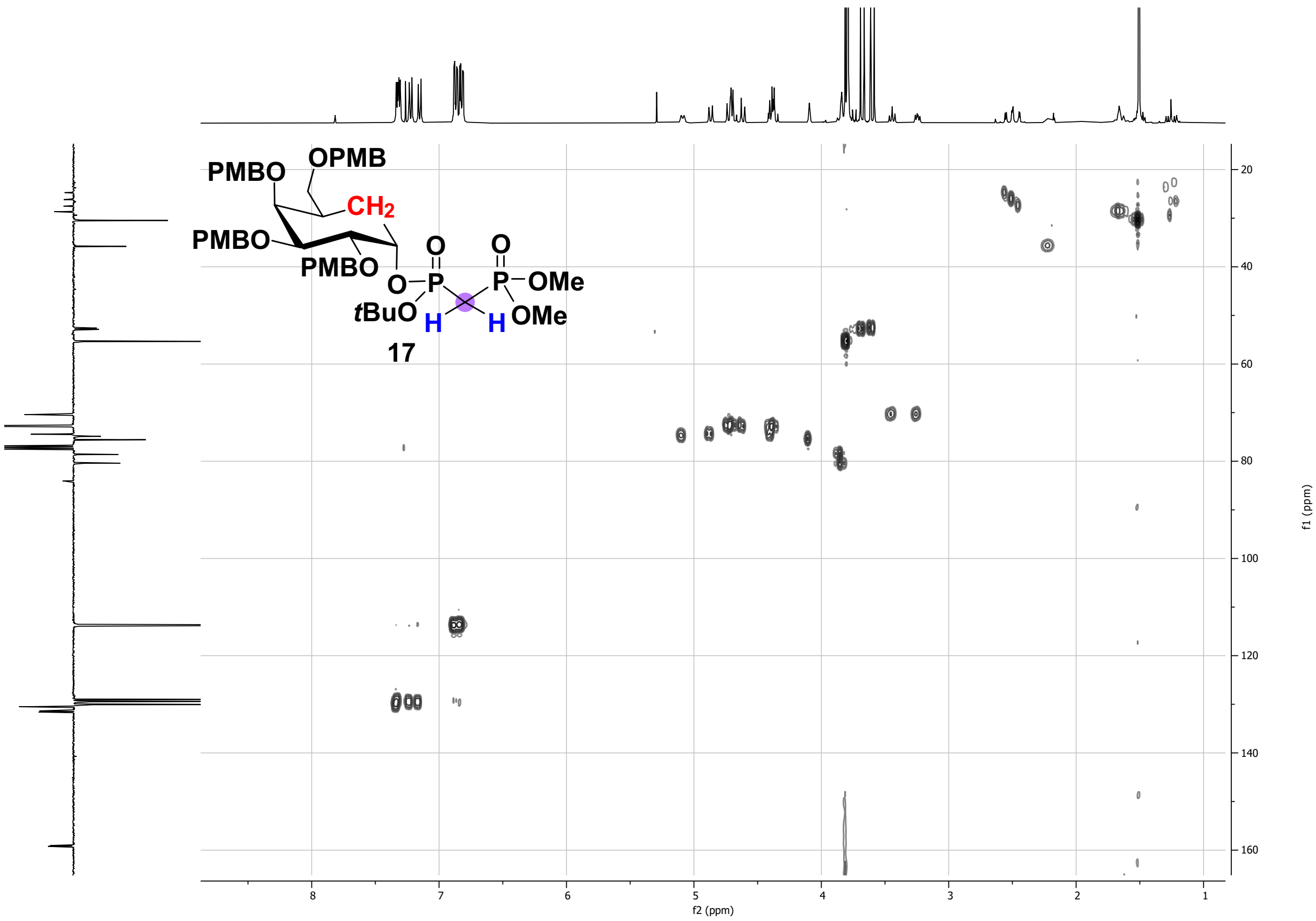

^{1}H-^{13}C HSQC (400 MHz, CDCl$_3$) of compound **17**

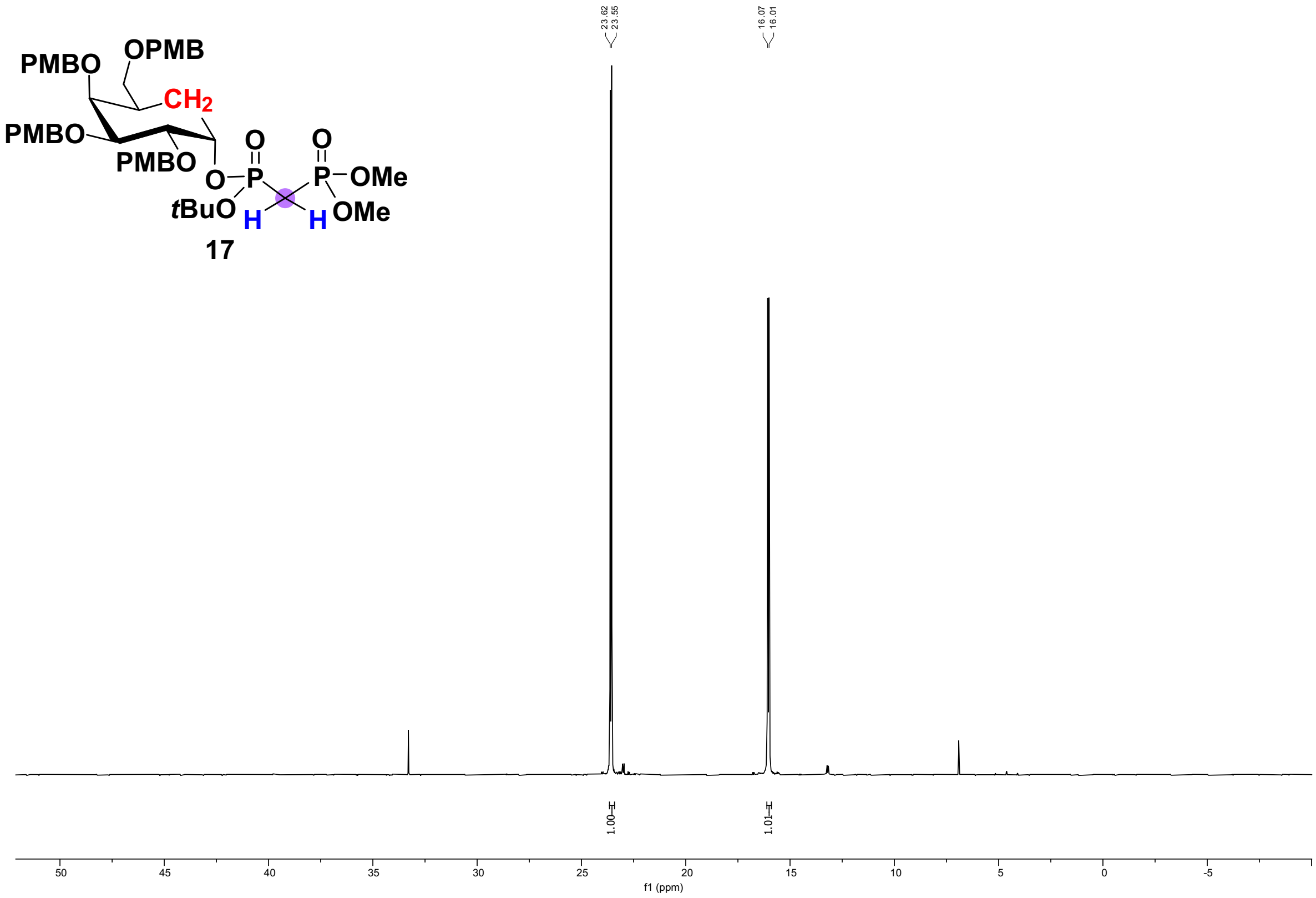

^{31}P NMR (162 MHz, $CDCl_3$) of compound **17**

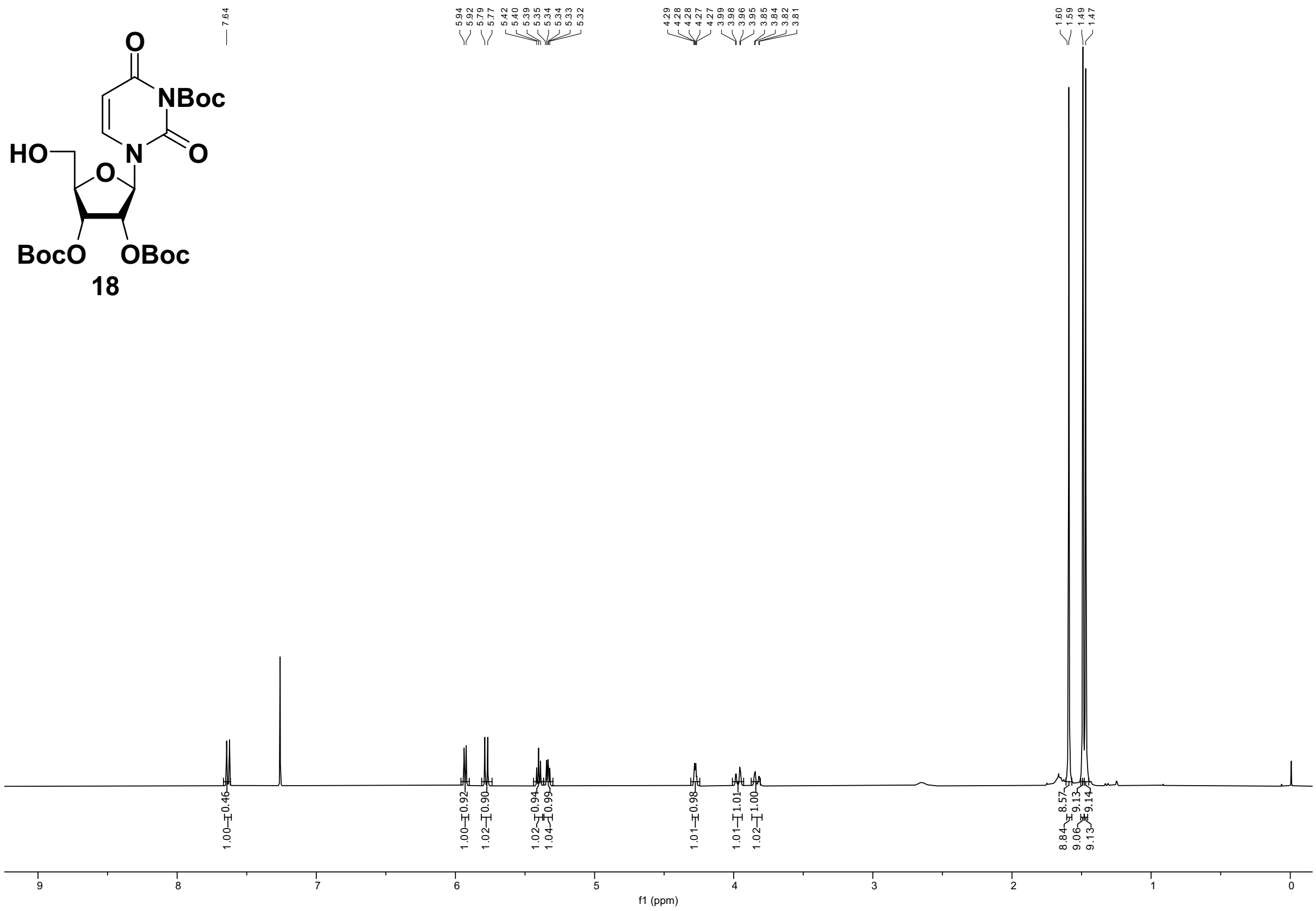

^{1}H NMR (400 MHz, $CDCl_3$) of compound **18**

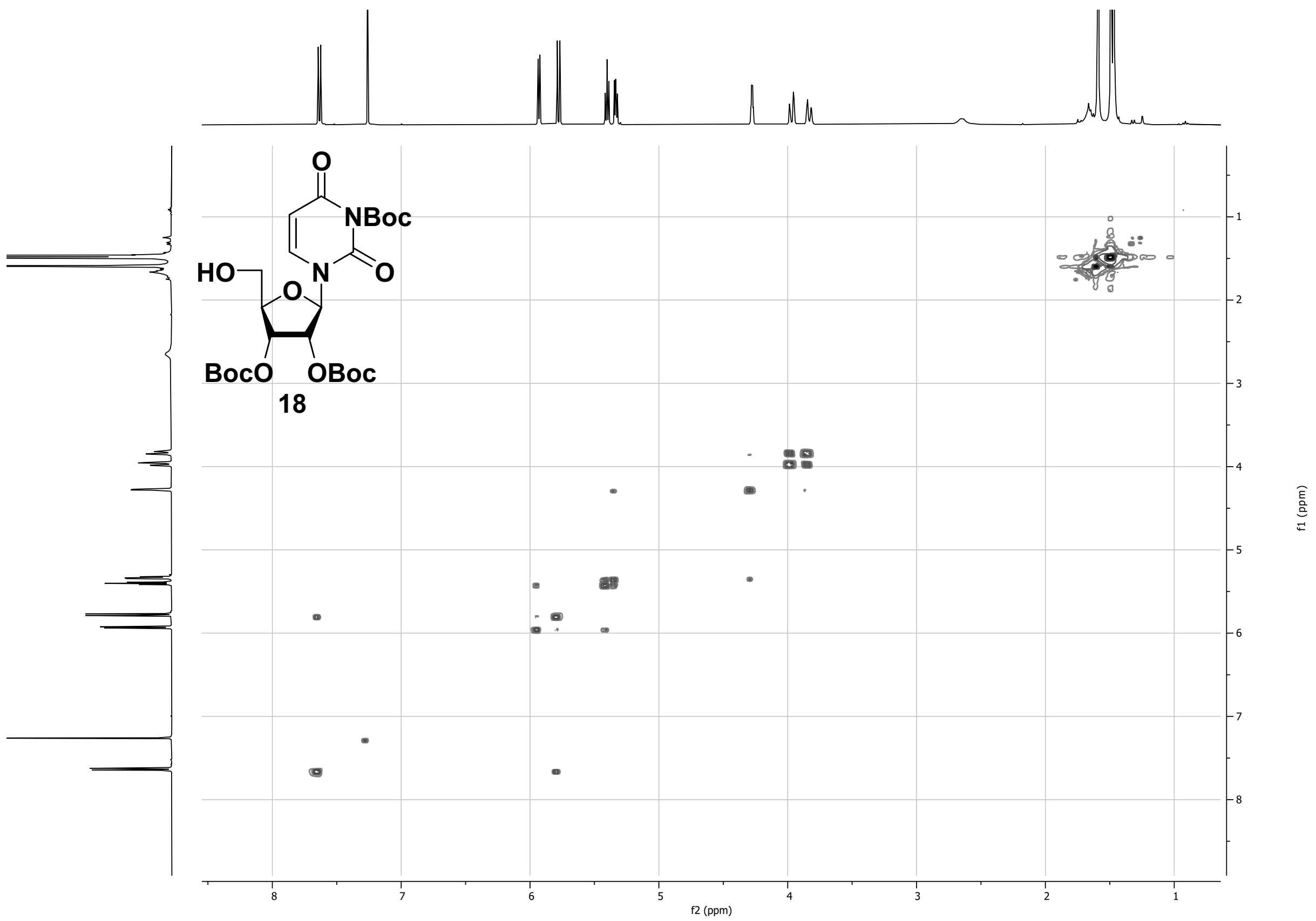

^{1}H-^{1}H COSY (400 MHz, $CDCl_3$) of compound **18**

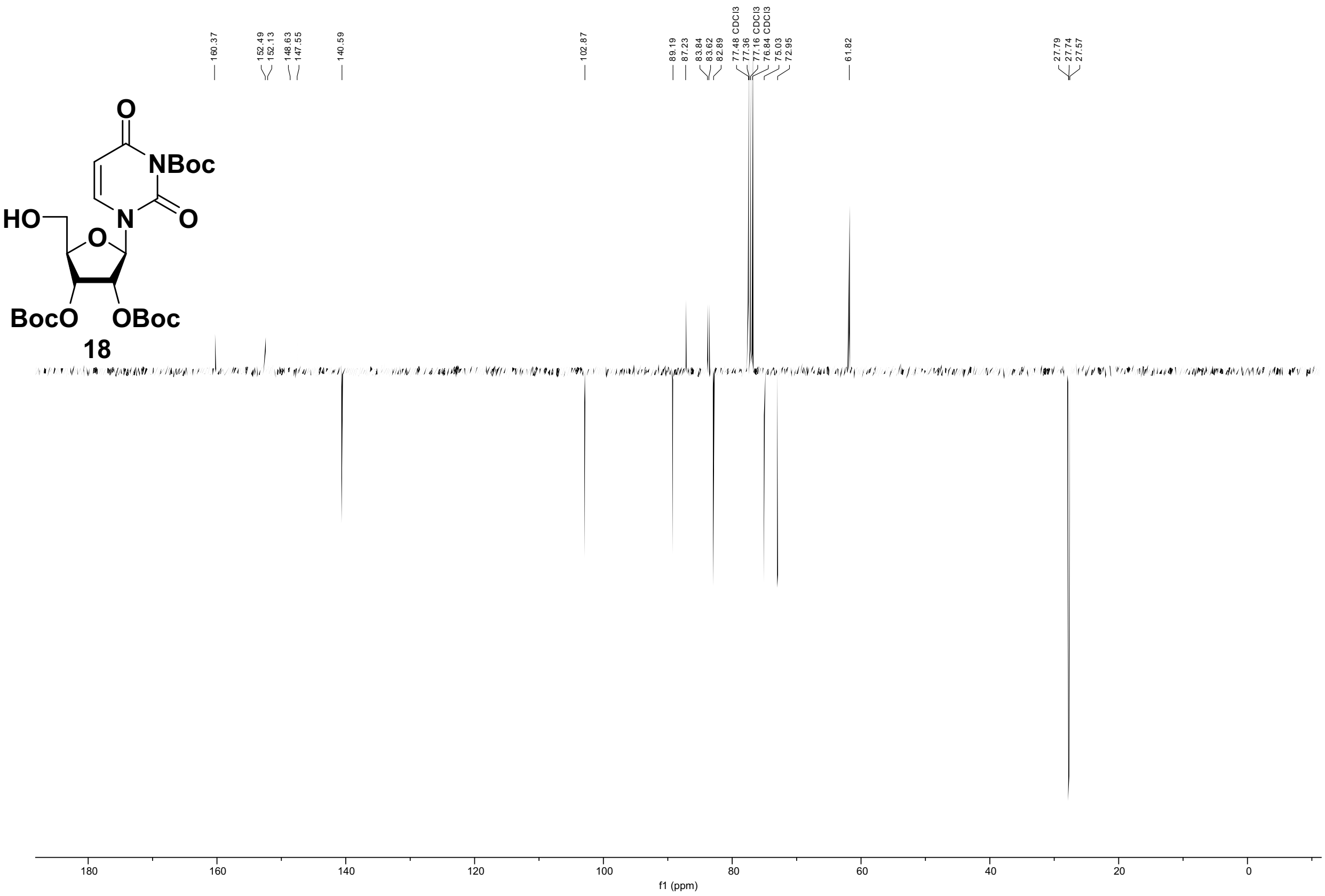

^{13}C NMR (101 MHz, $CDCl_3$) of compound **18**

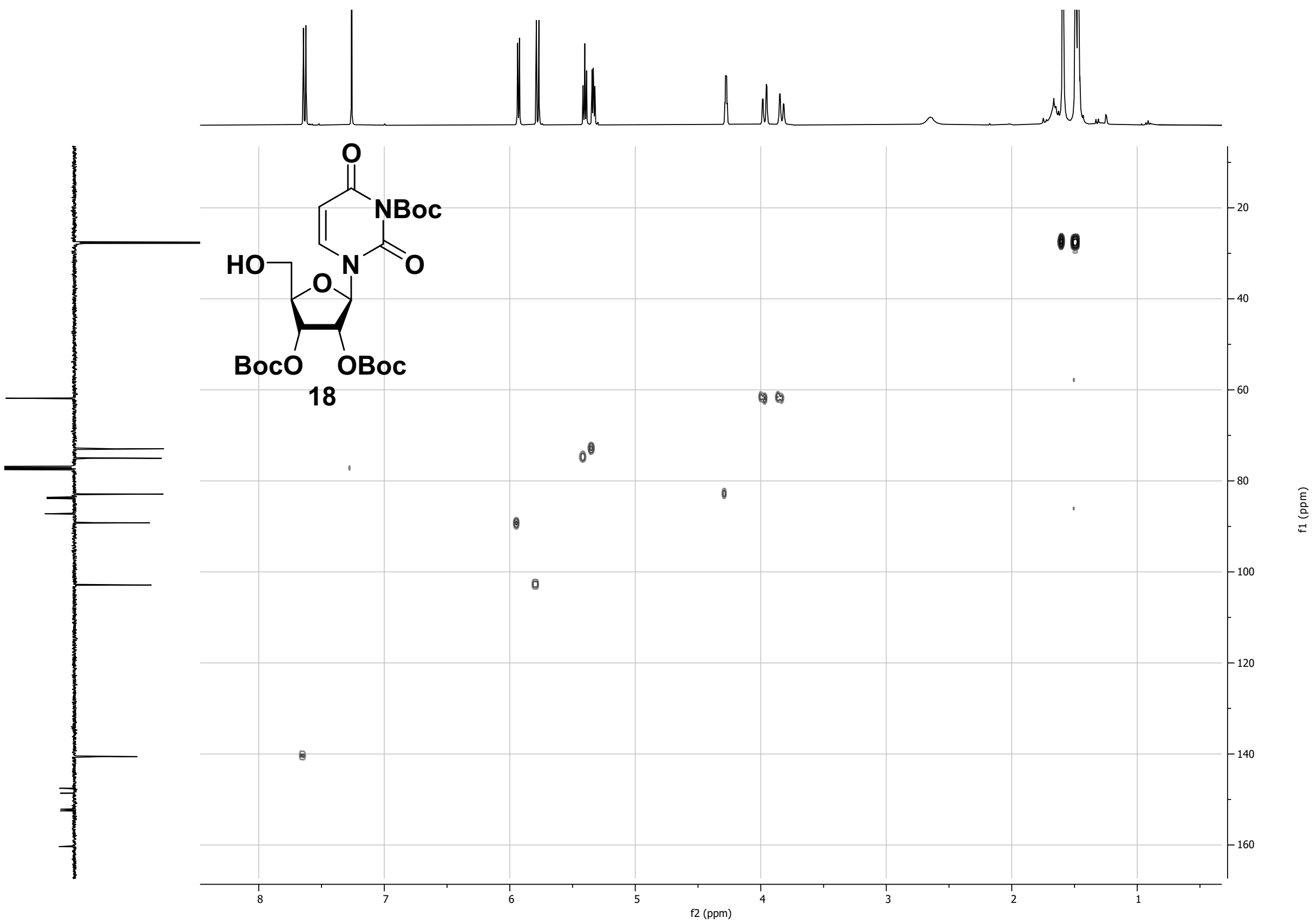

^{1}H-^{13}C HSQC (400 MHz, CDCl$_3$) of compound **18**

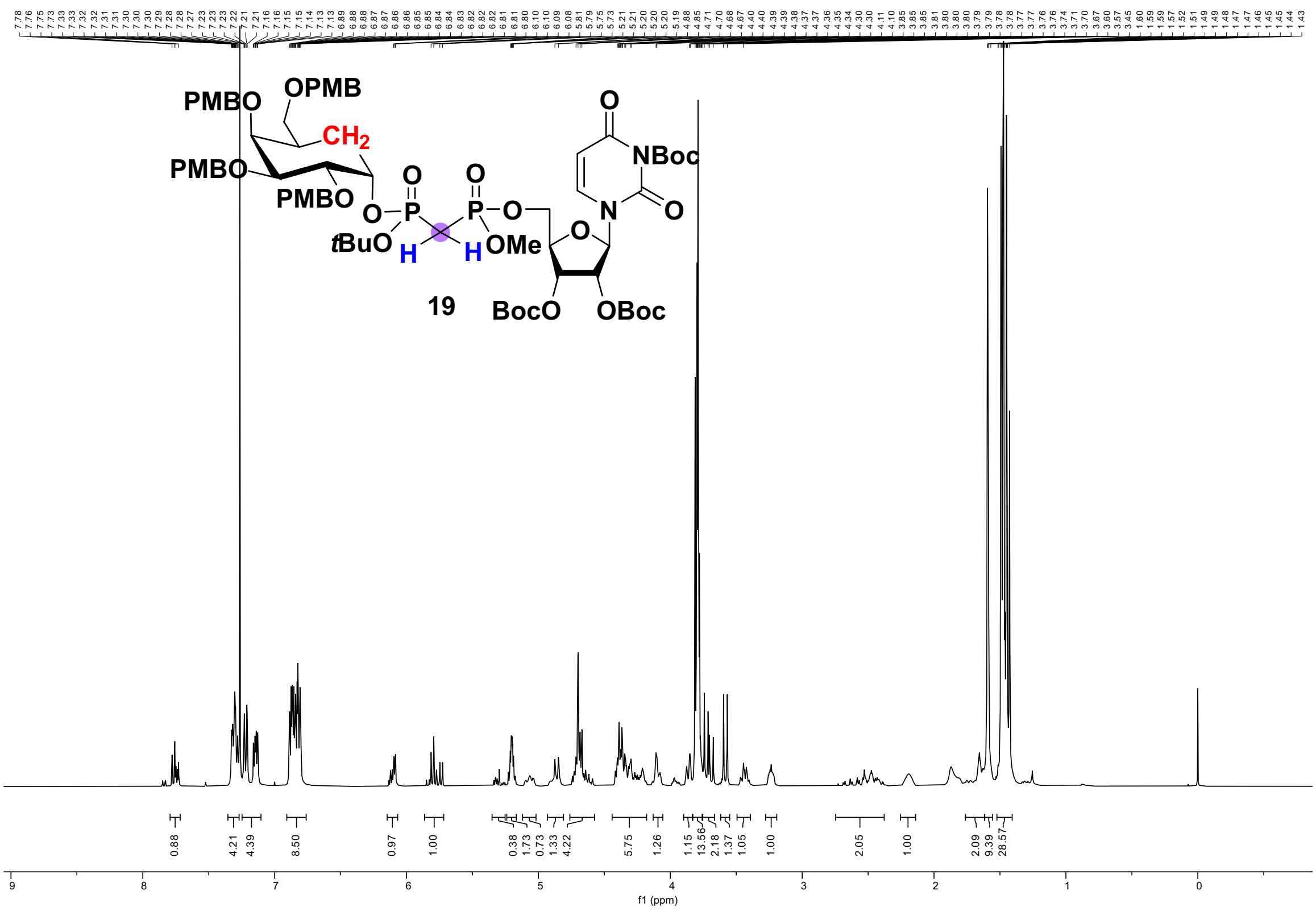

^{1}H NMR (400 MHz, CDCl$_3$) of compound **19**

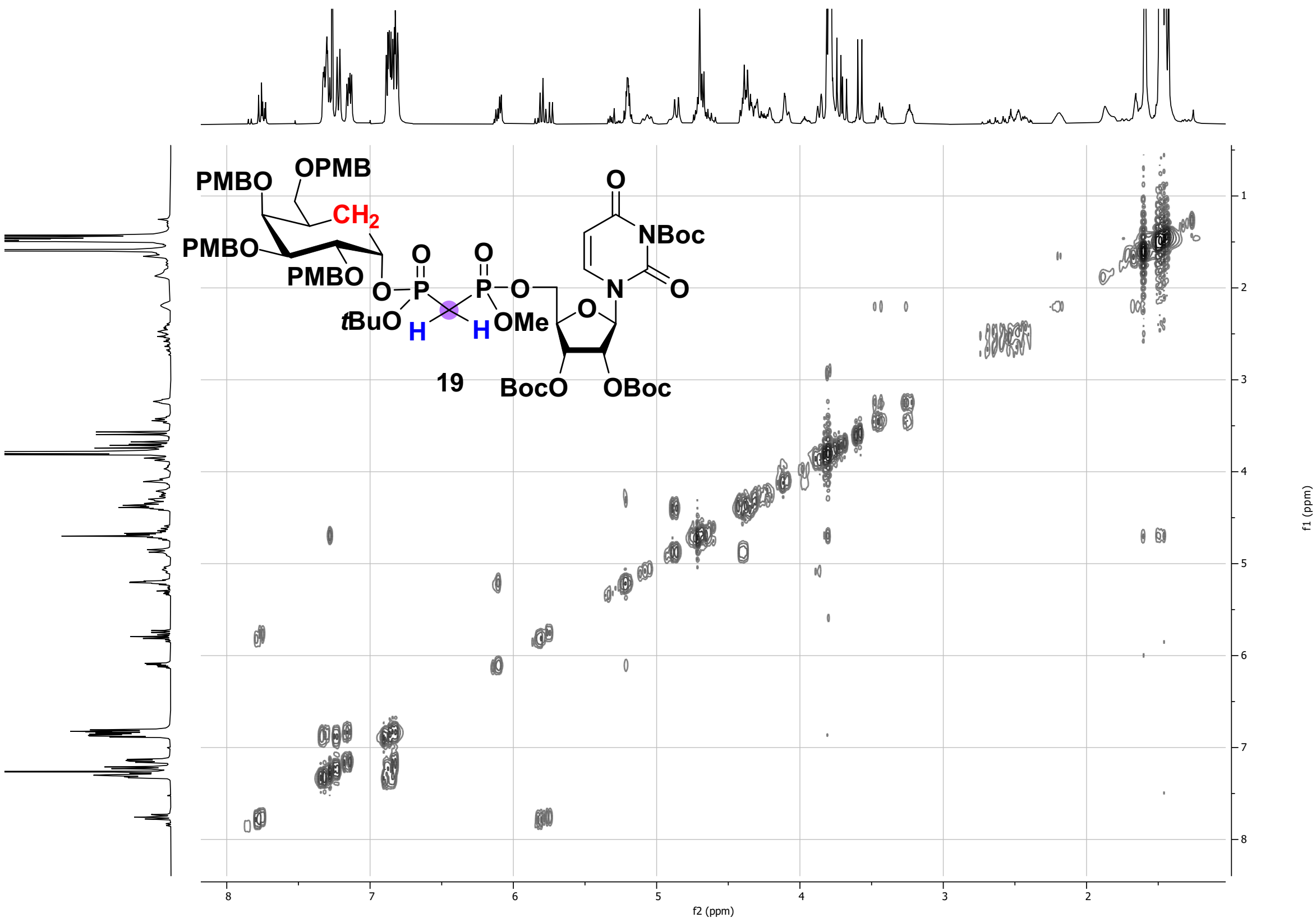

^{1}H-^{1}H COSY (400 MHz, CDCl$_3$) of compound **19**

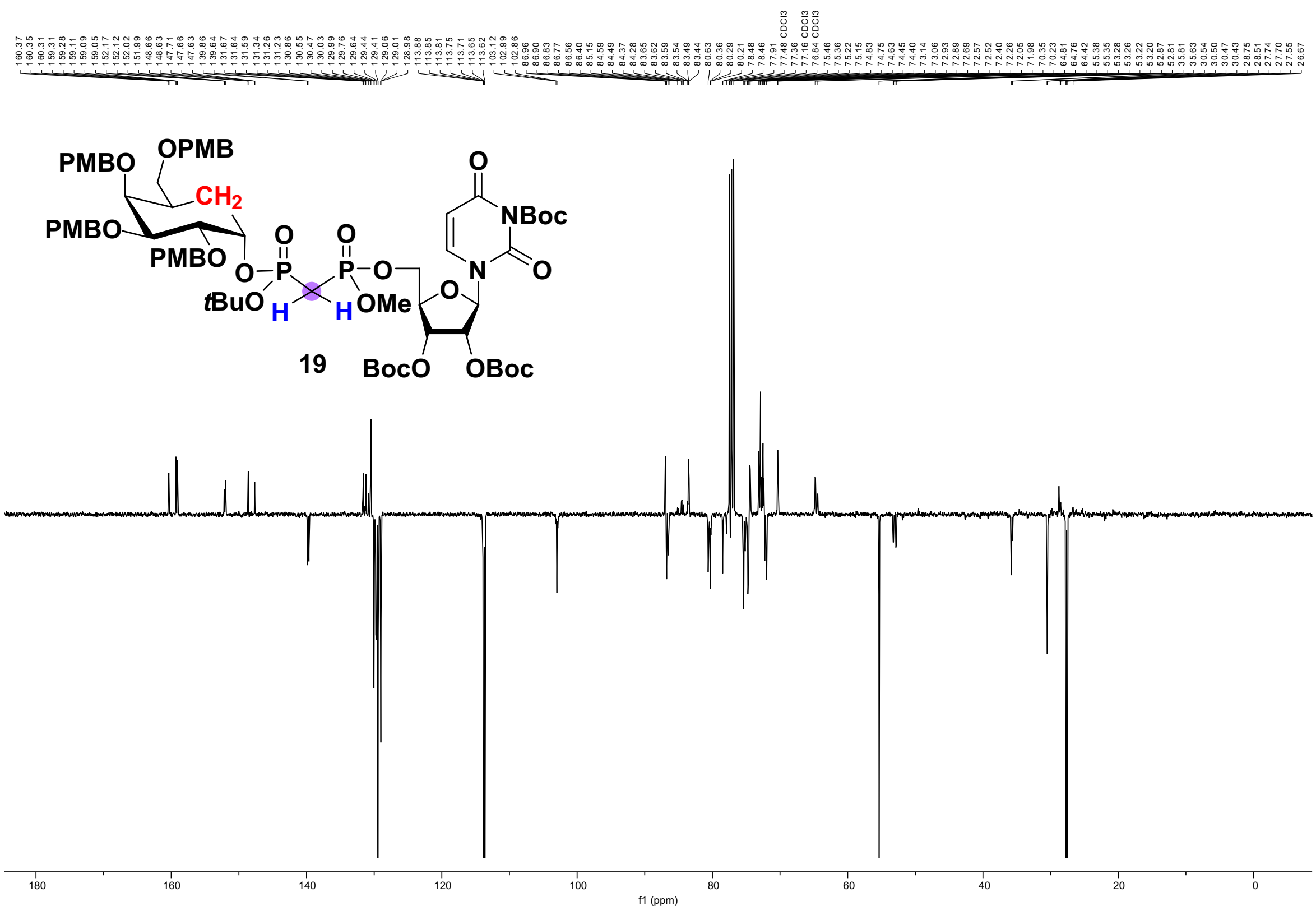

^{13}C NMR (101 MHz, CDCl$_3$) of compound **19**

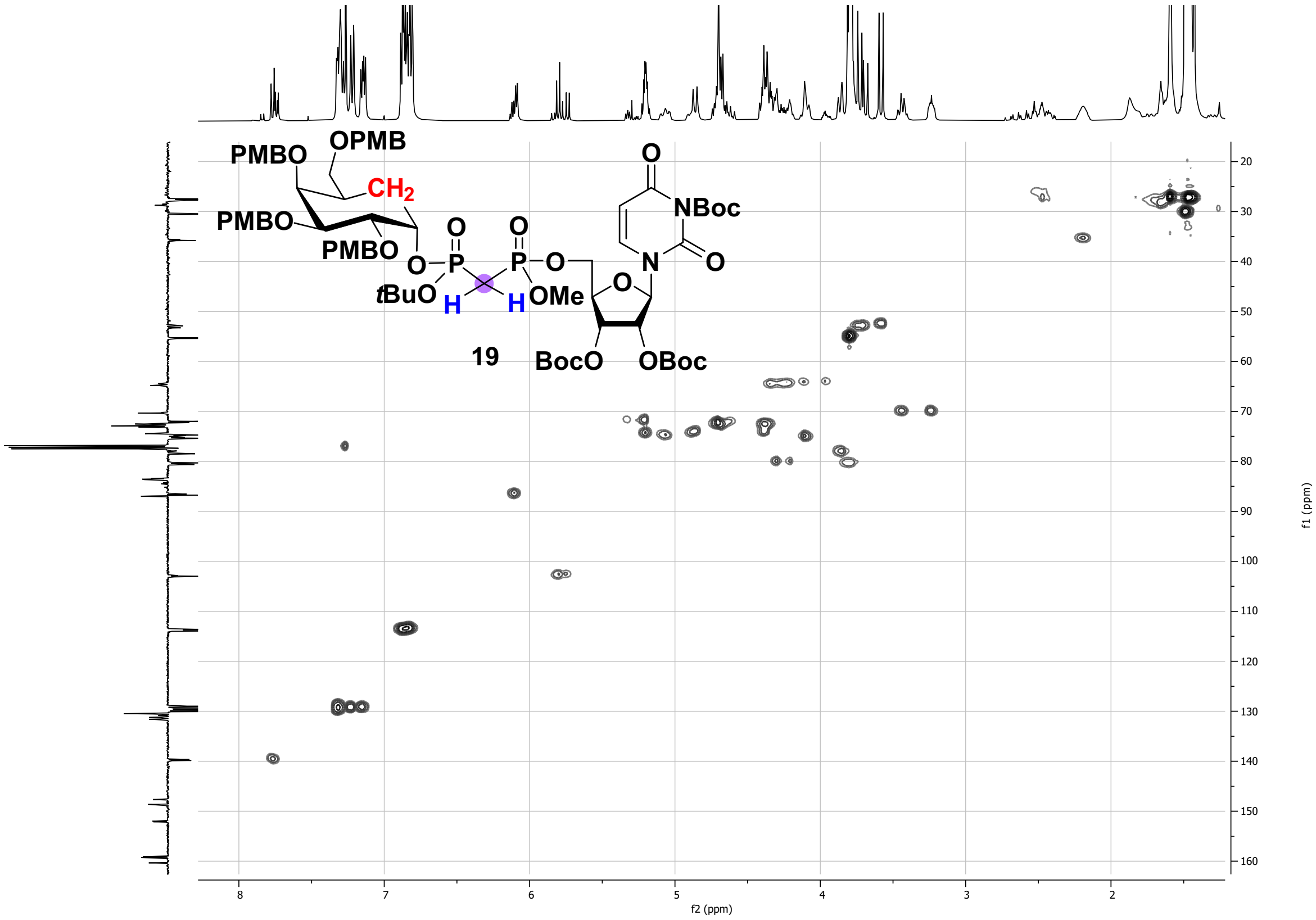

^{1}H-^{13}C HSQC (400 MHz, $CDCl_3$) of compound **19**

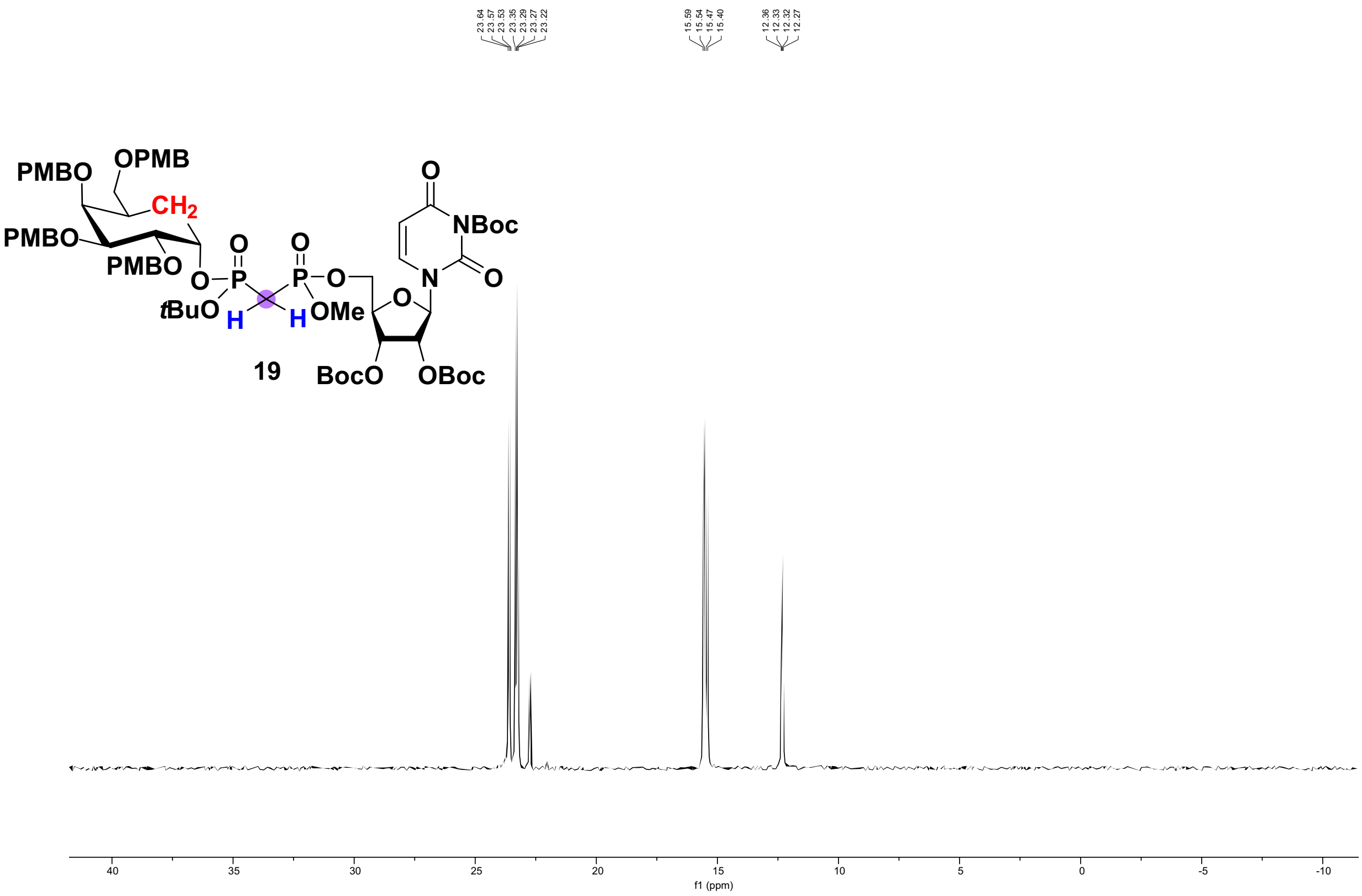

^{31}P NMR (162 MHz, $CDCl_3$) of compound **19**

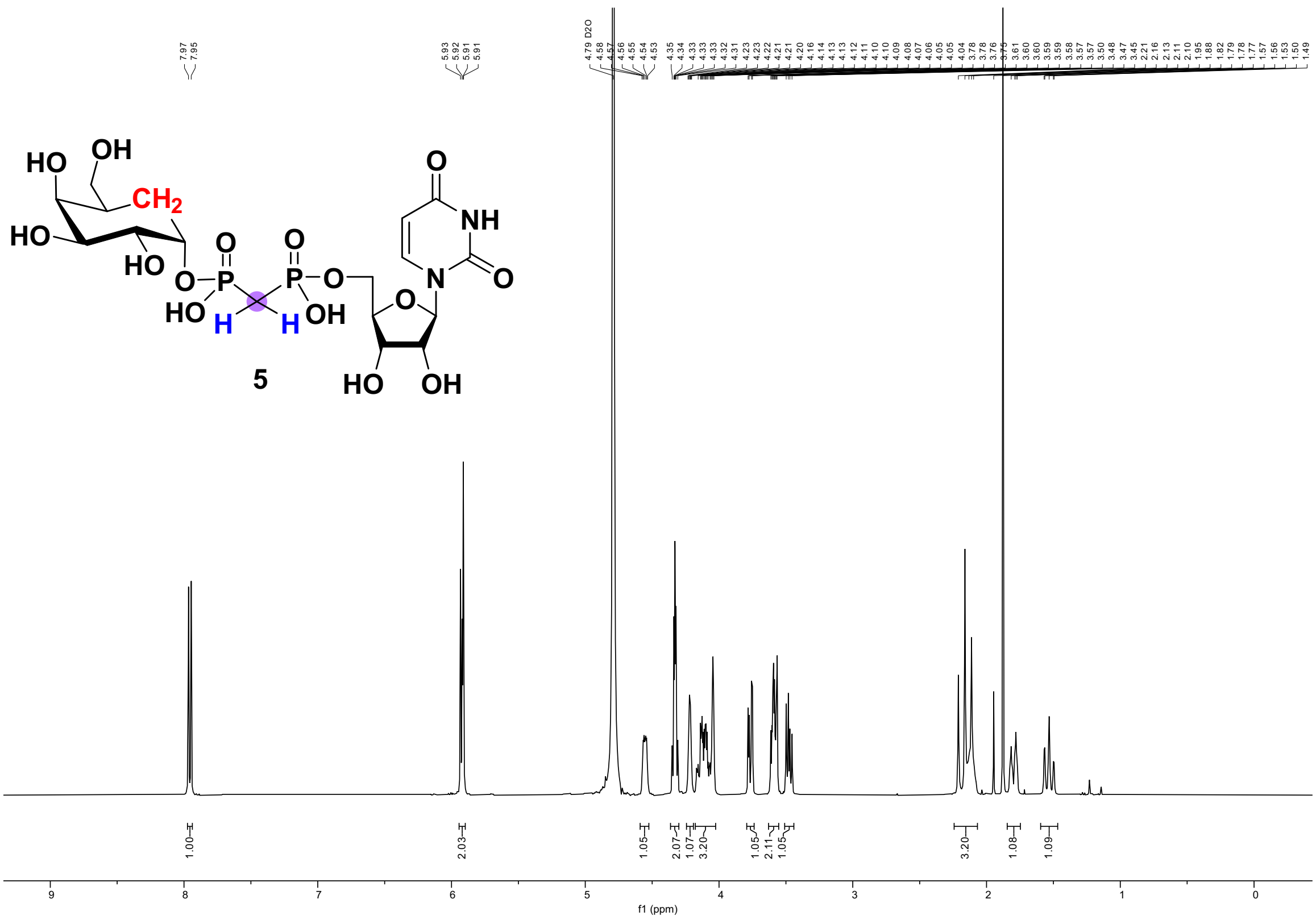

^{1}H NMR (400 MHz, $CDCl_3$) of compound **5**

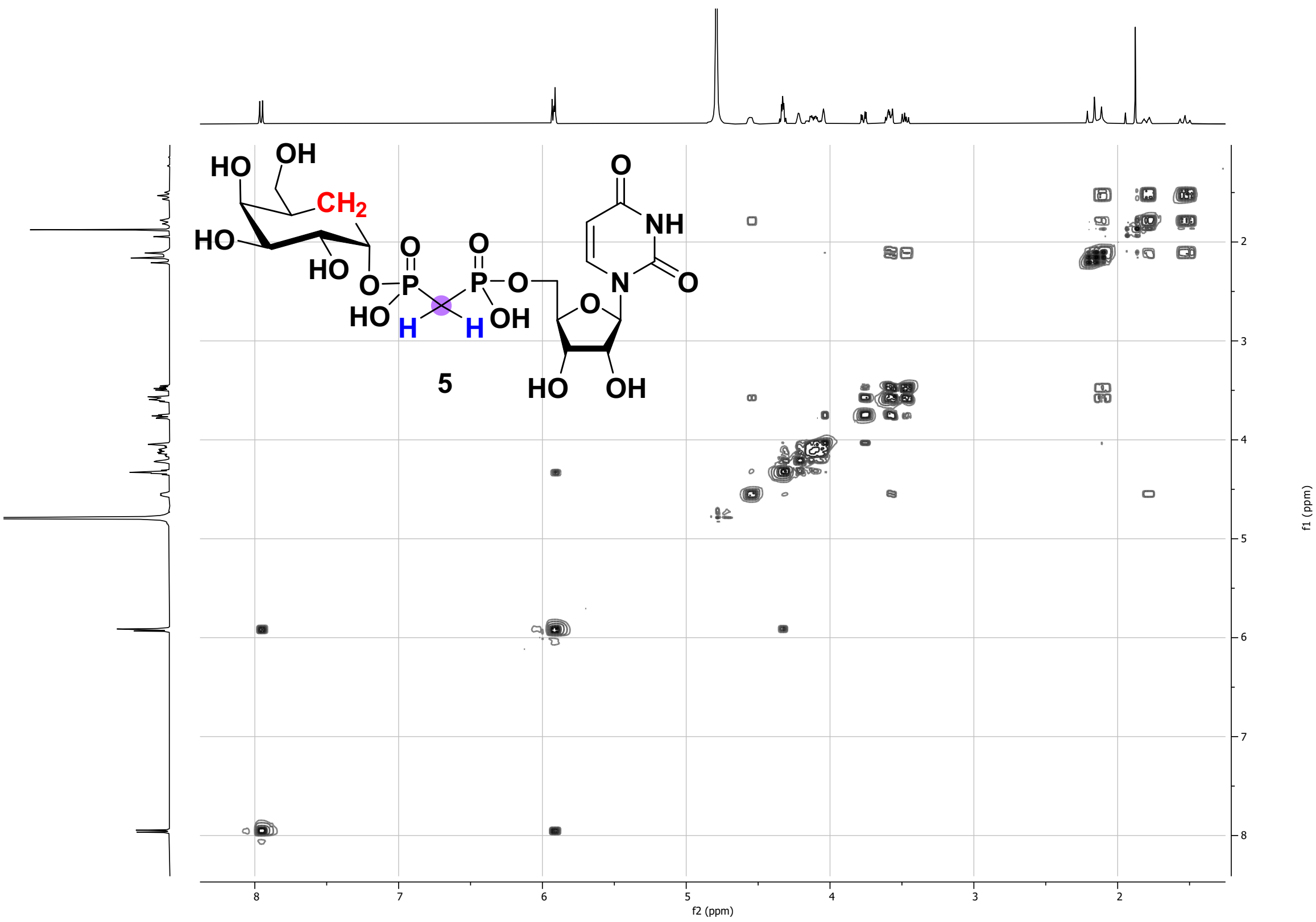

^{1}H-^{1}H COSY (400 MHz, $CDCl_3$) of compound **5**

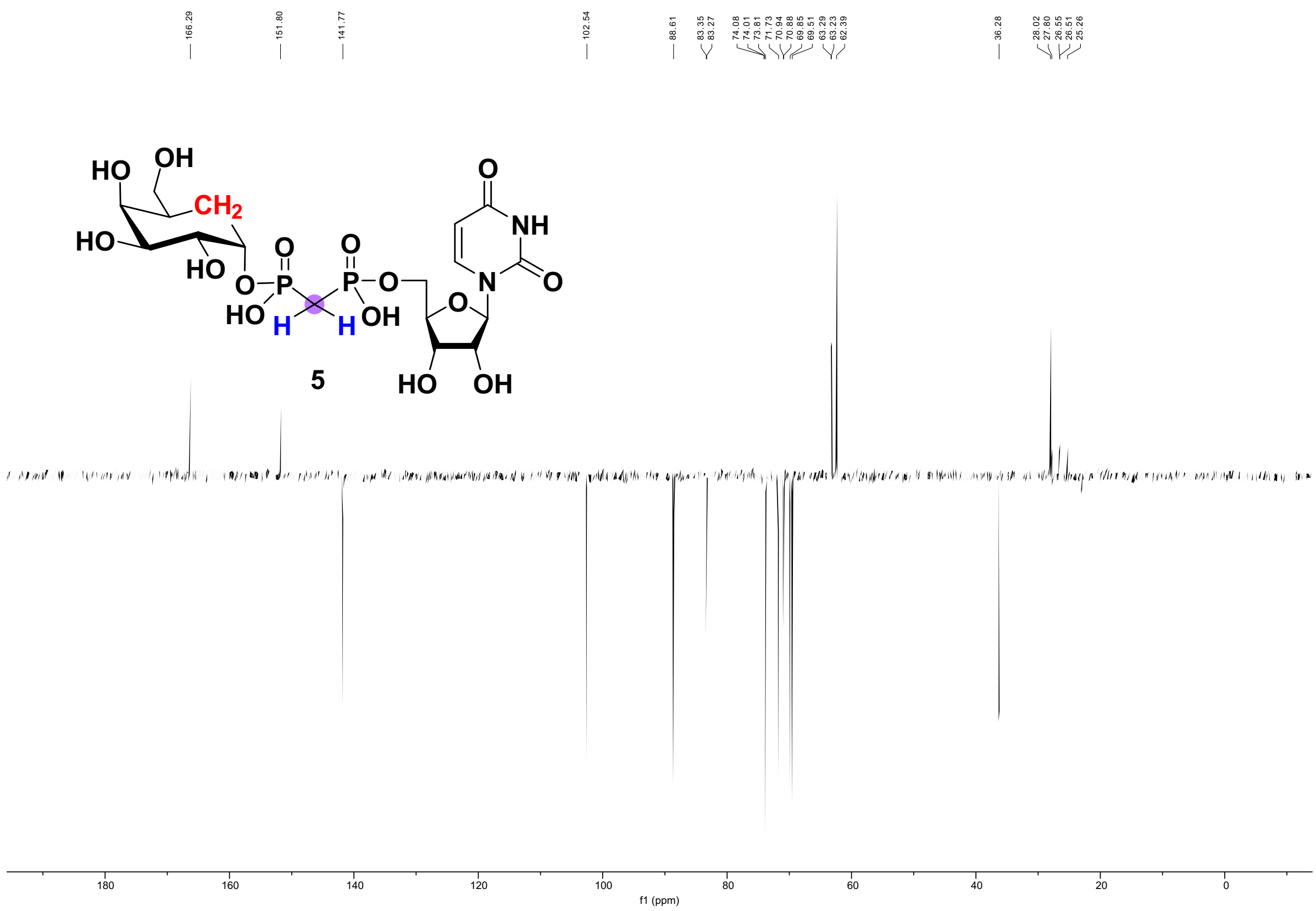

^{13}C NMR (101 MHz, CDCl$_3$) of compound **5**

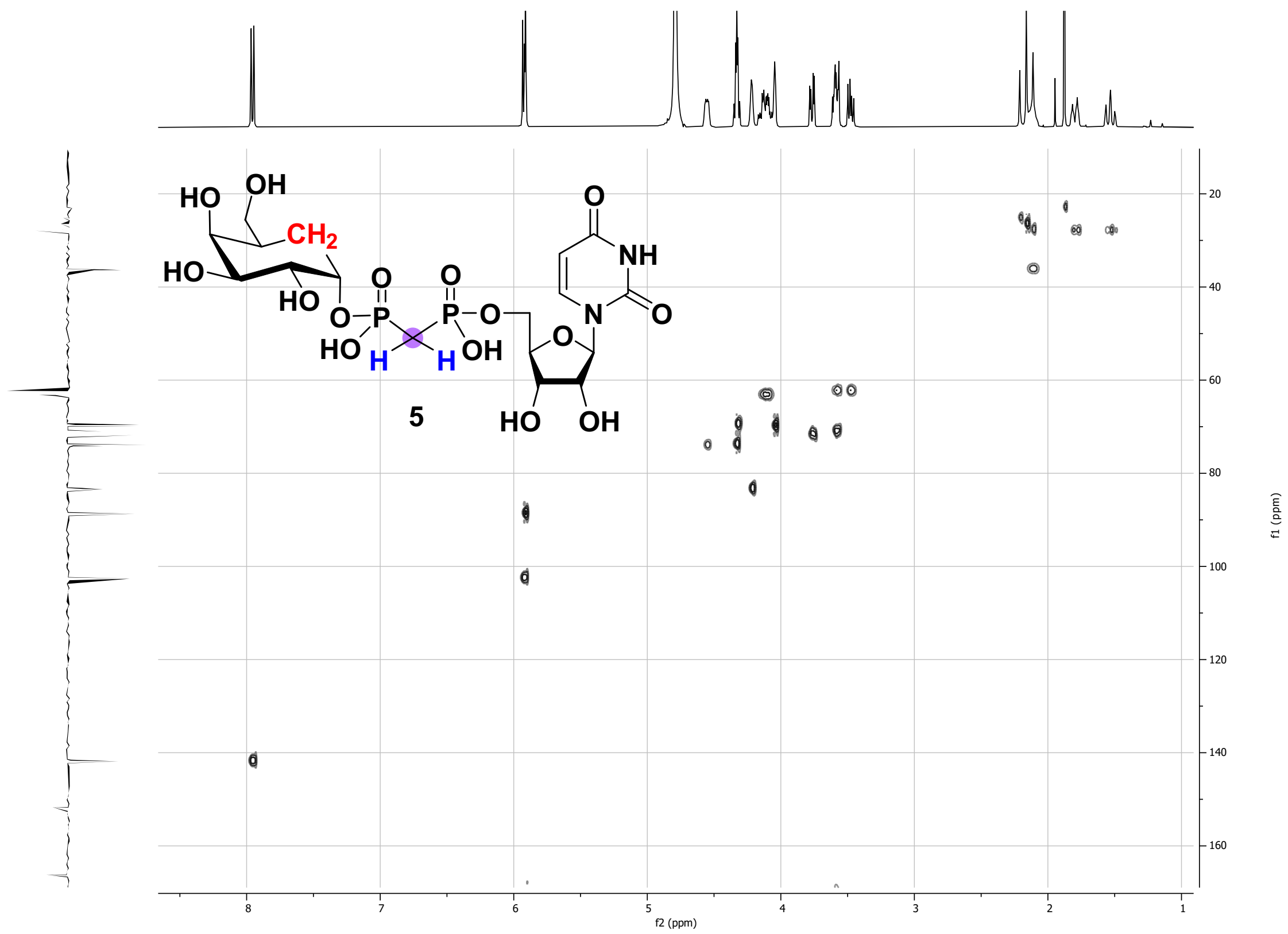

^{1}H-^{13}C HSQC (400 MHz, CDCl$_3$) of compound **5**

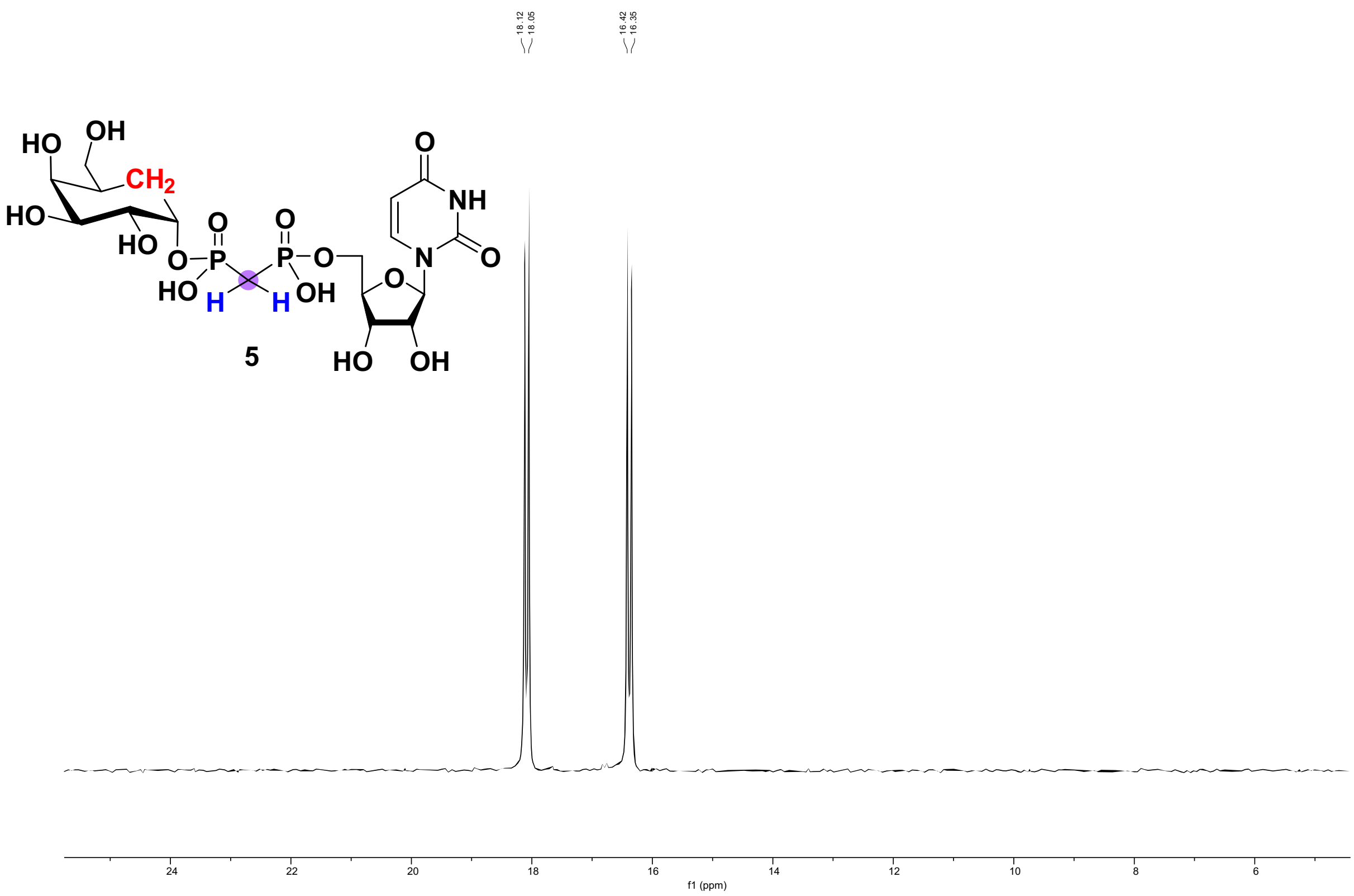

^{31}P NMR (162 MHz, $CDCl_3$) of compound **5**

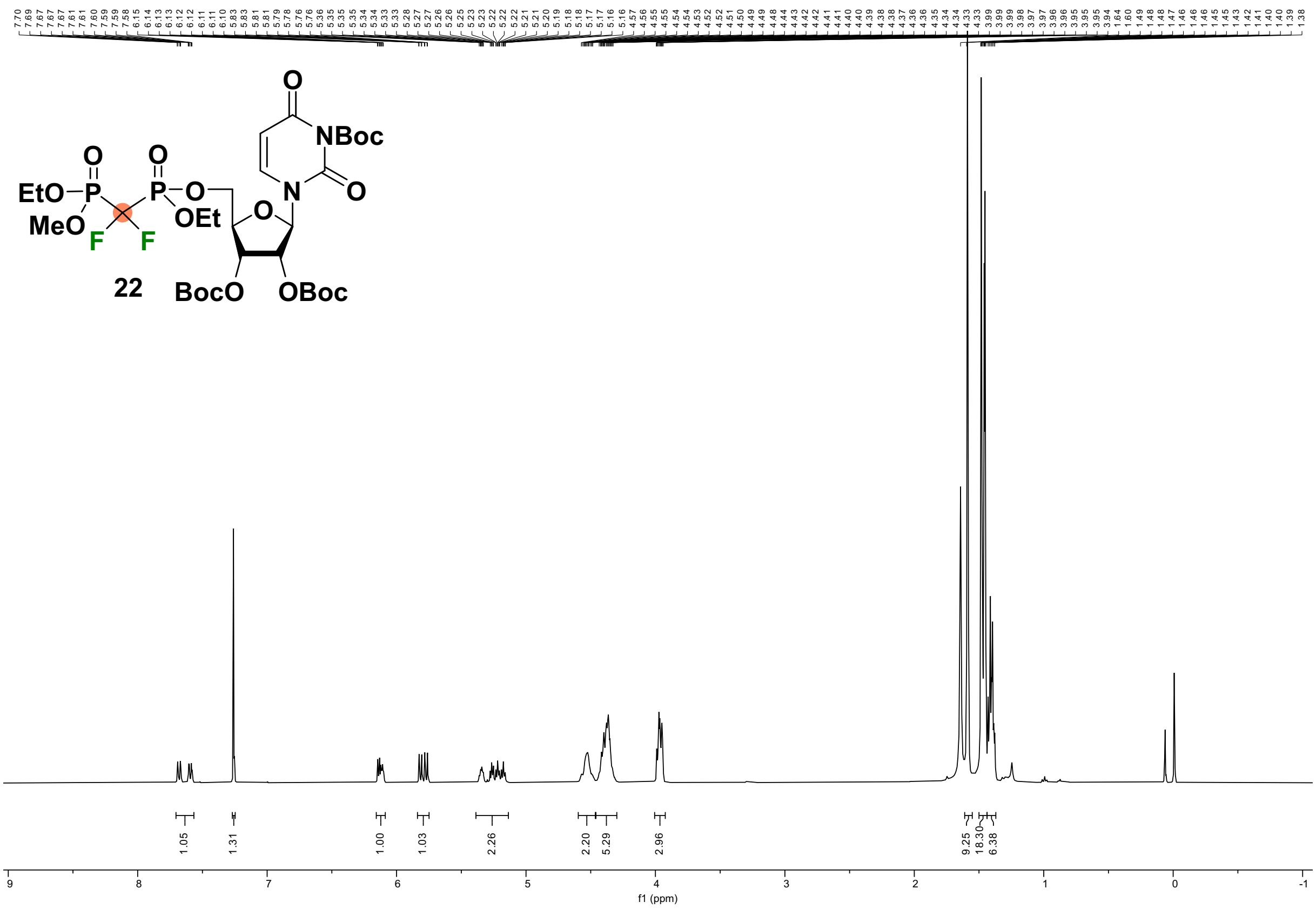

^{1}H NMR (400 MHz, CDCl$_3$) of compound **22**

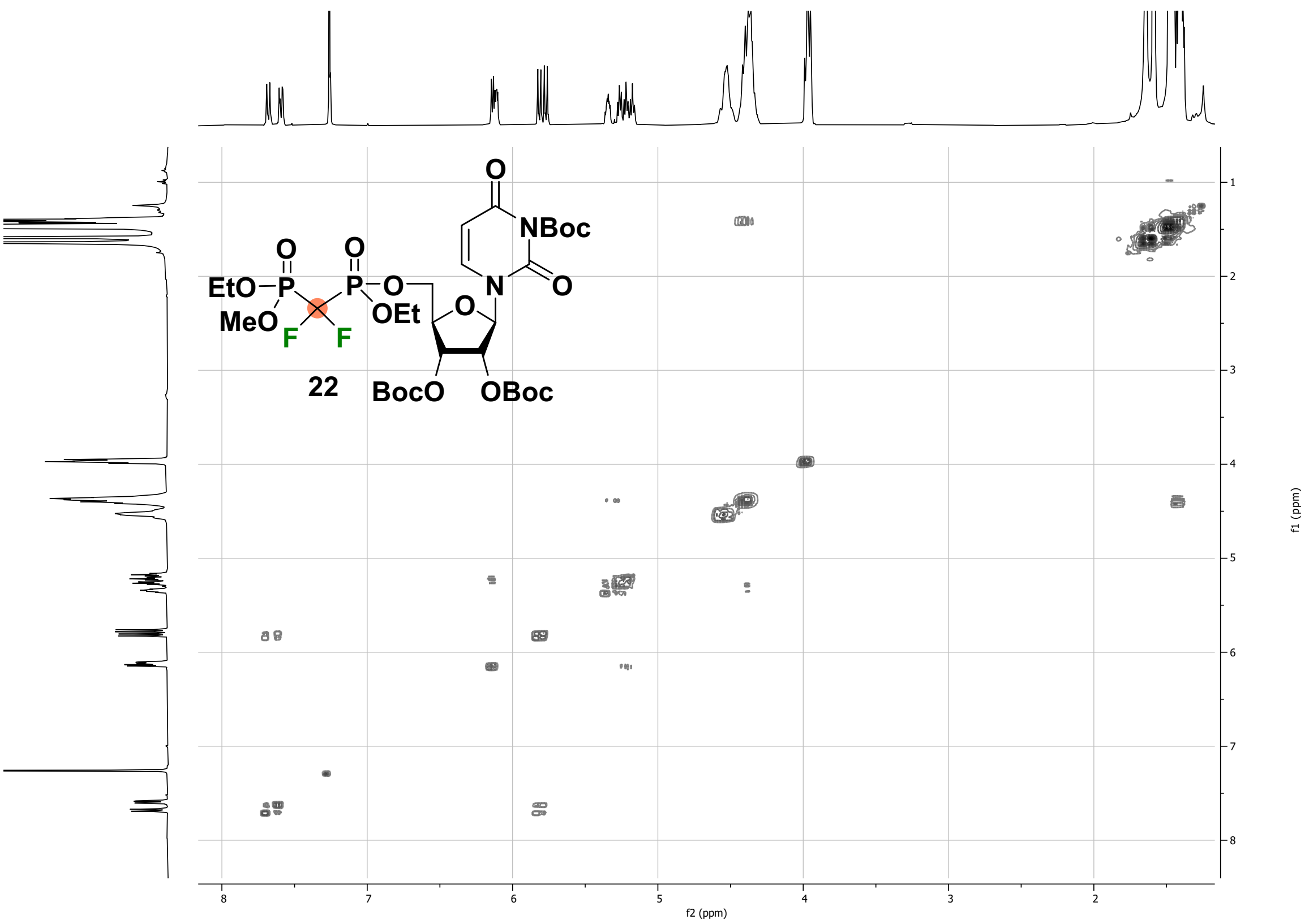

^{1}H-^{1}H COSY (400 MHz, CDCl$_3$) of compound **22**

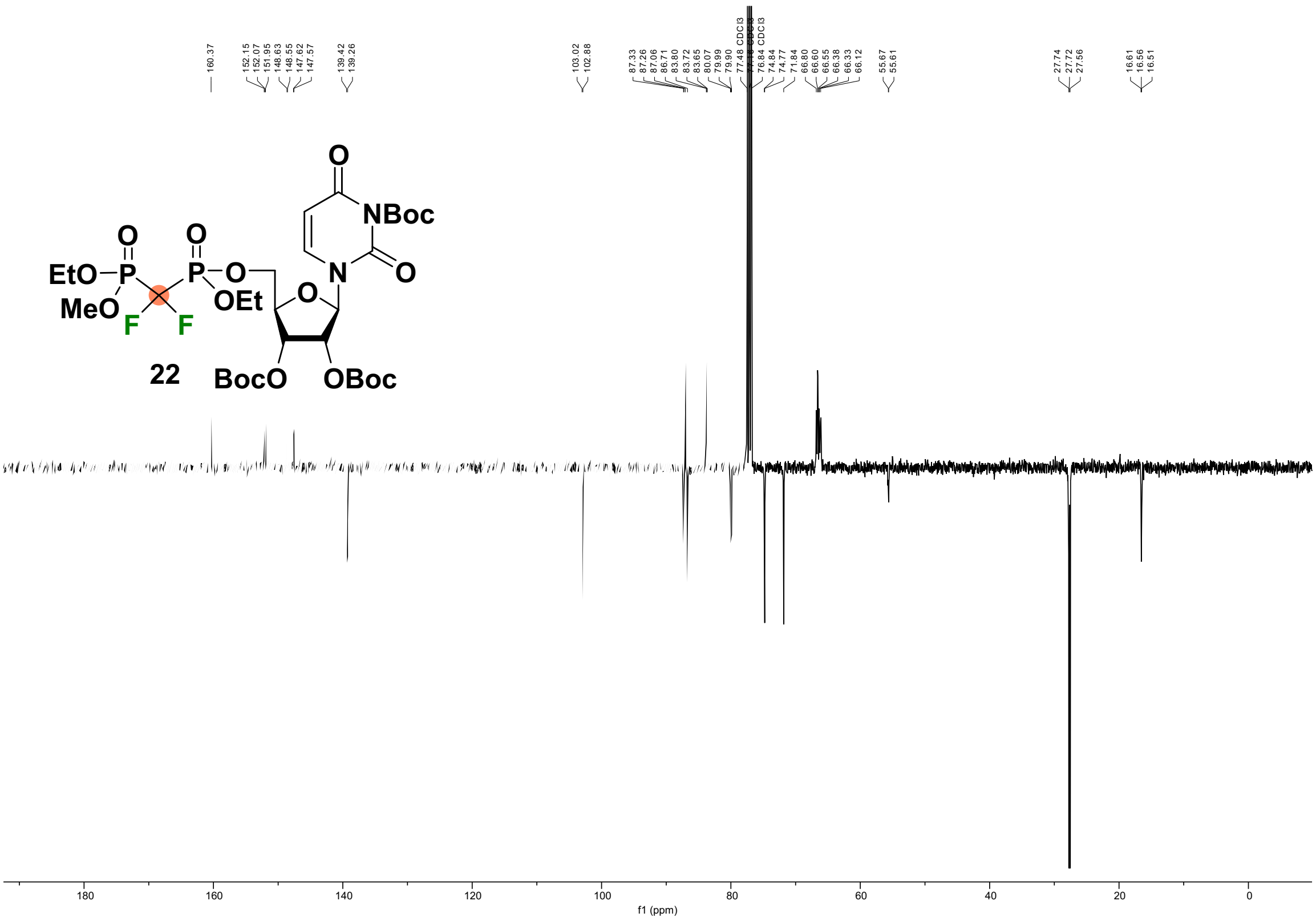

^{13}C NMR (101 MHz, $CDCl_3$) of compound **22**

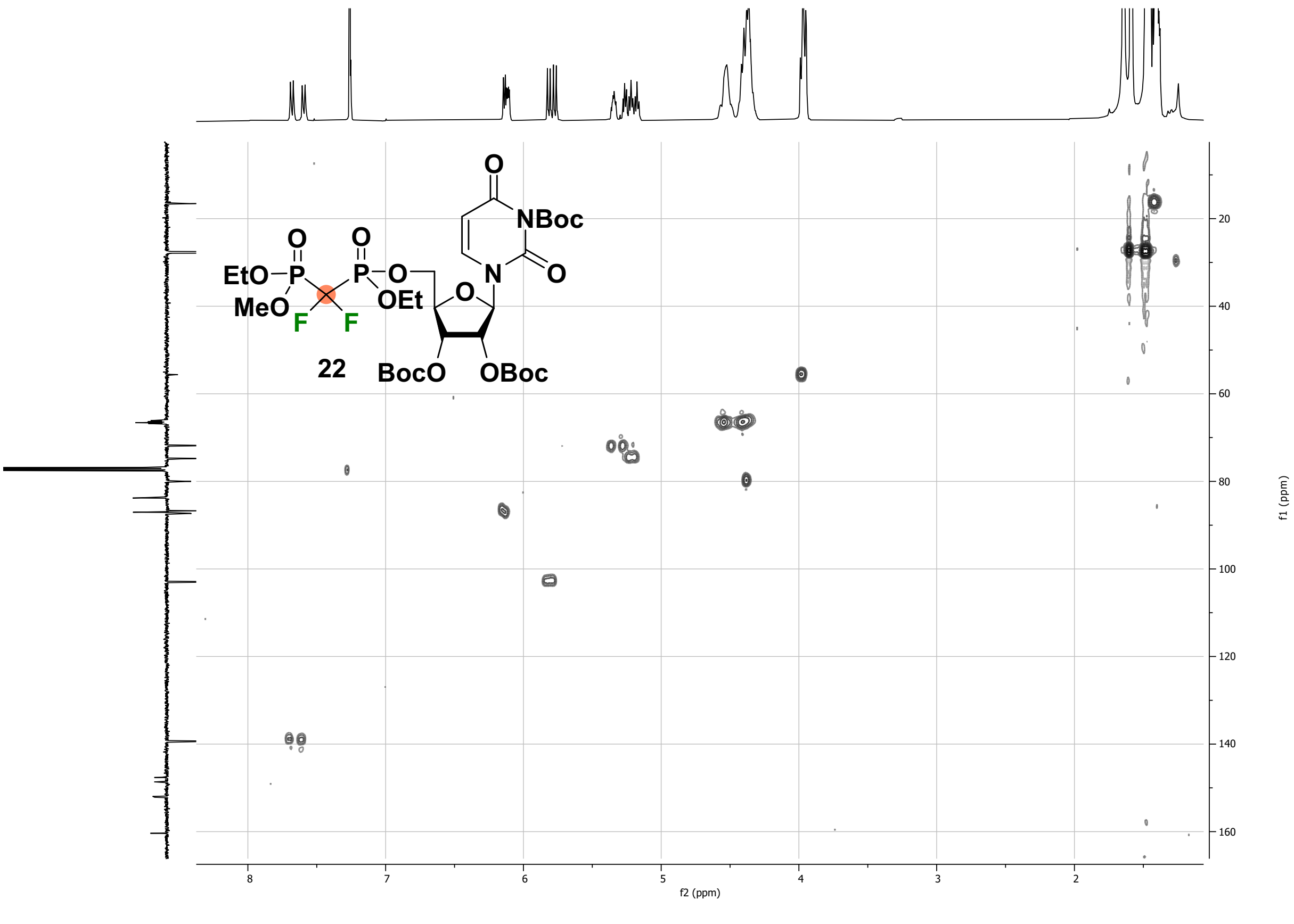

^{1}H-^{13}C HSQC (400 MHz, $CDCl_3$) of compound **22**

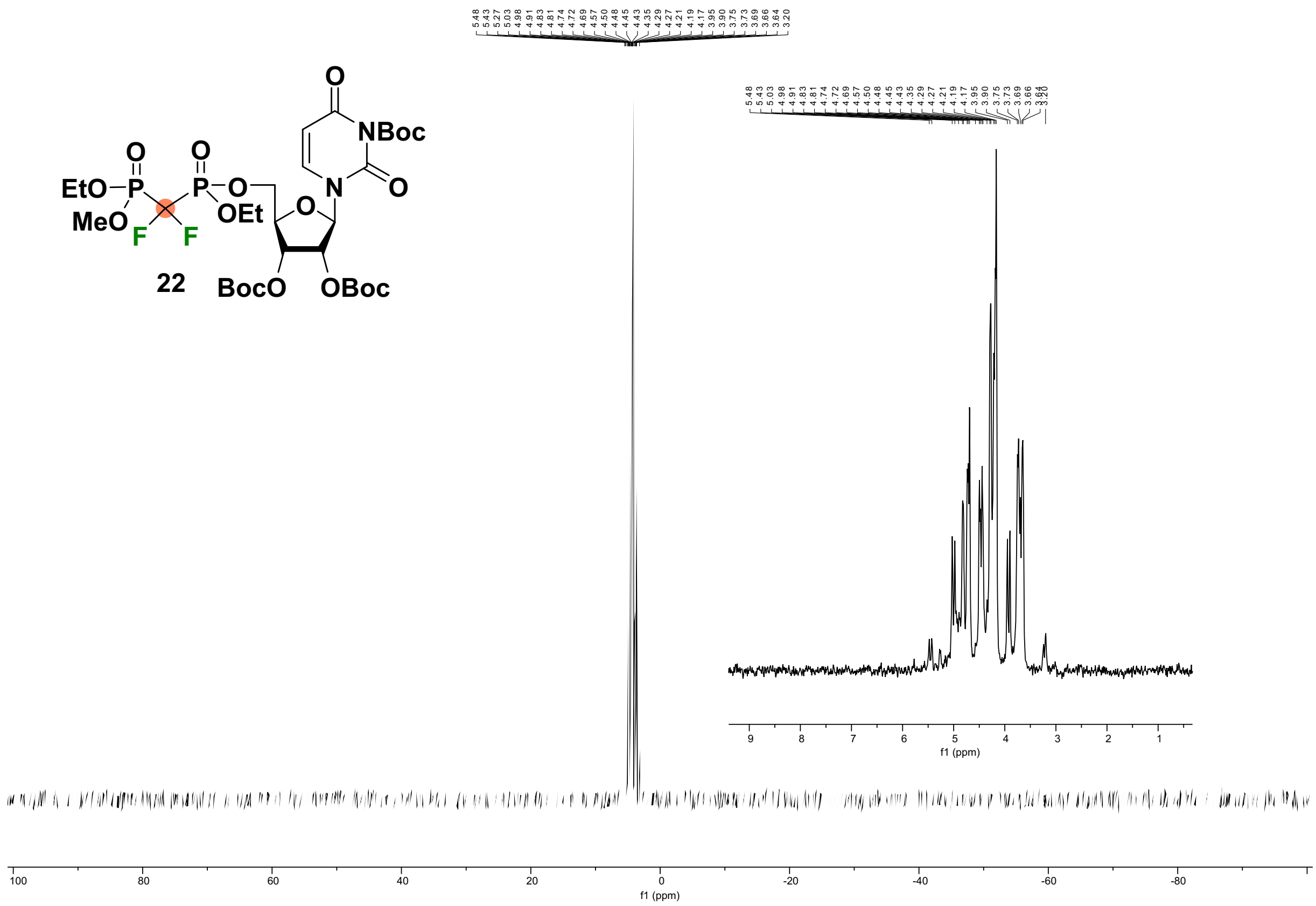

^{31}P NMR (162 MHz, $CDCl_3$) of compound **22**

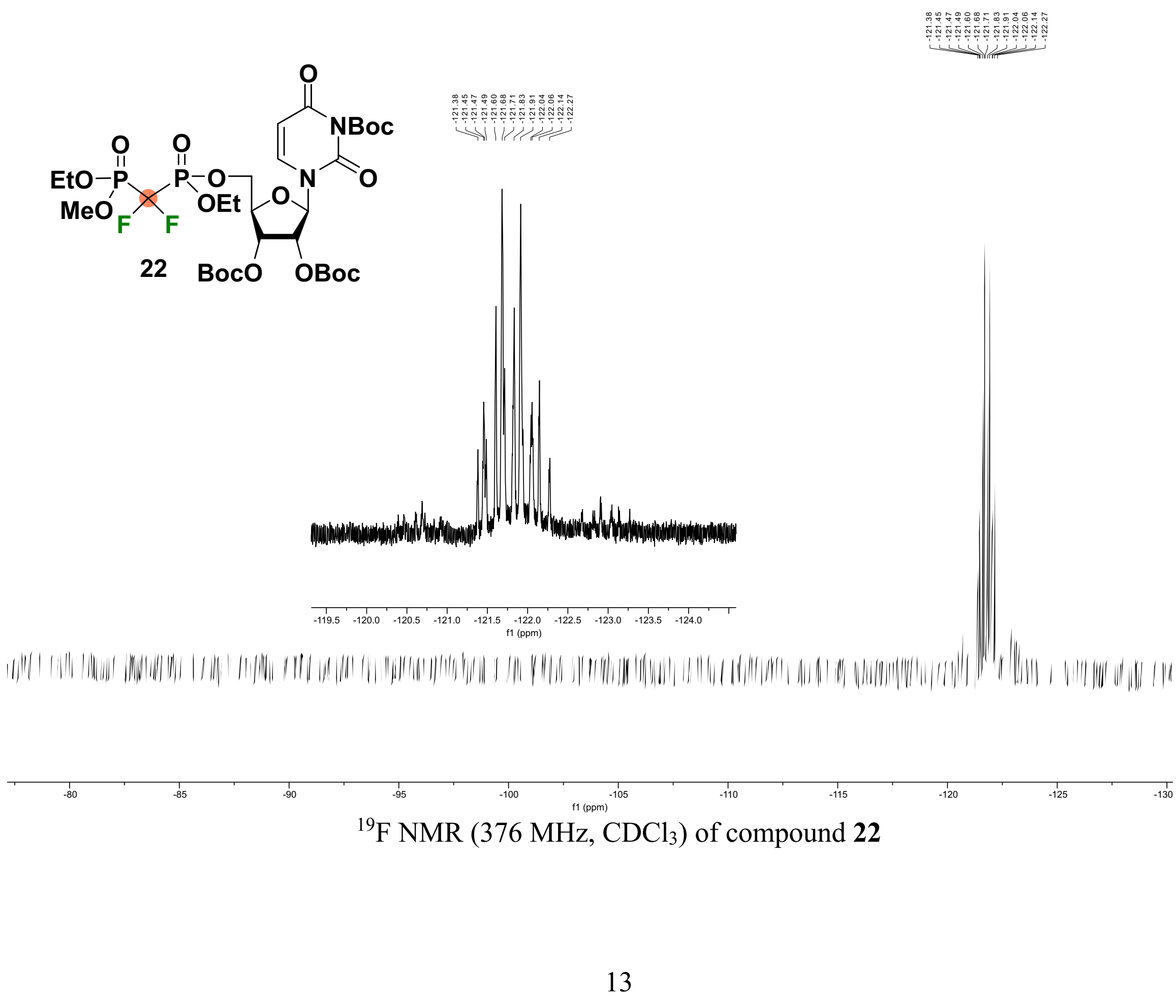

^{19}F NMR (376 MHz, $CDCl_3$) of compound **22**

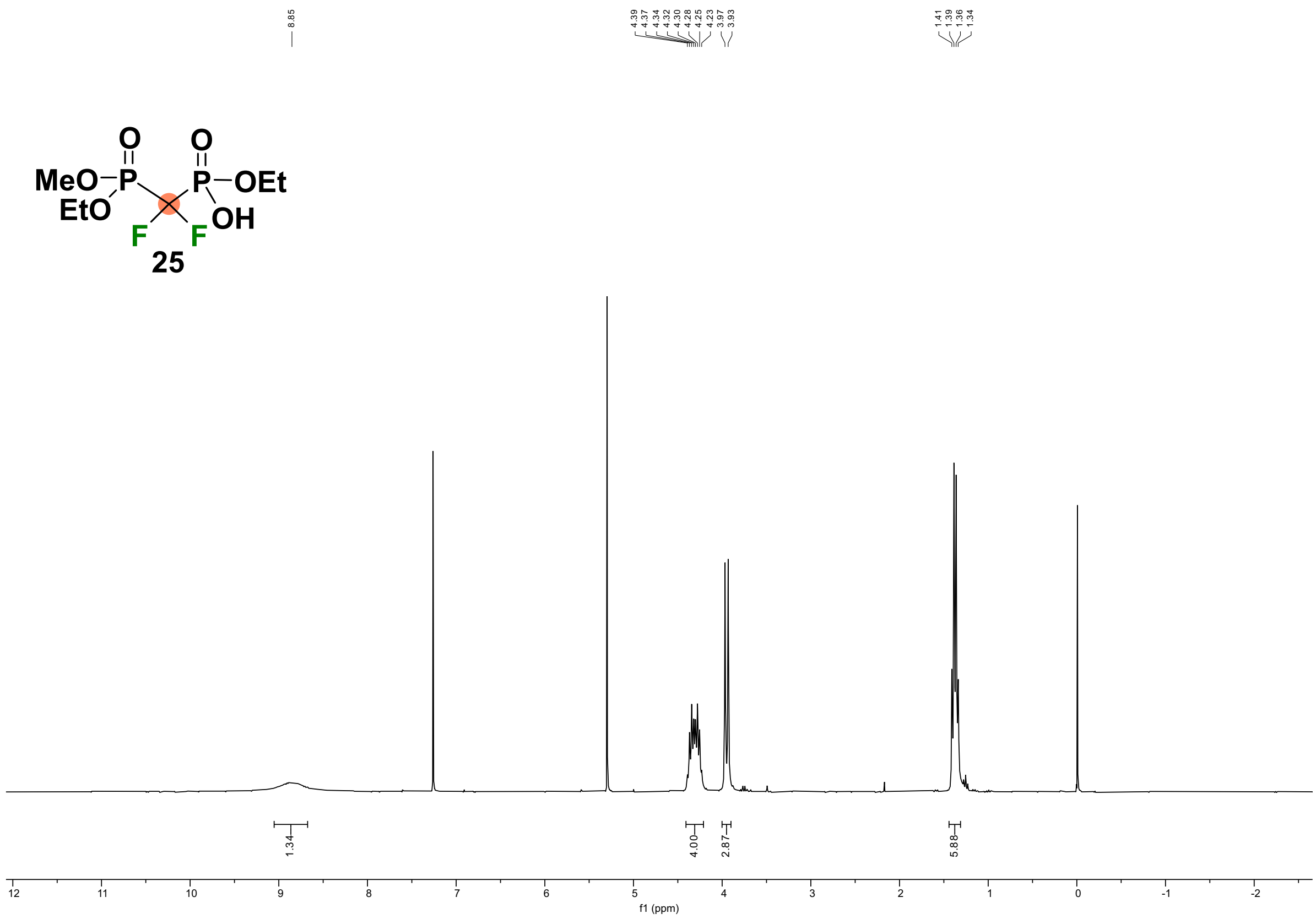

^{1}H NMR (300 MHz, $CDCl_3$) of compound **25**

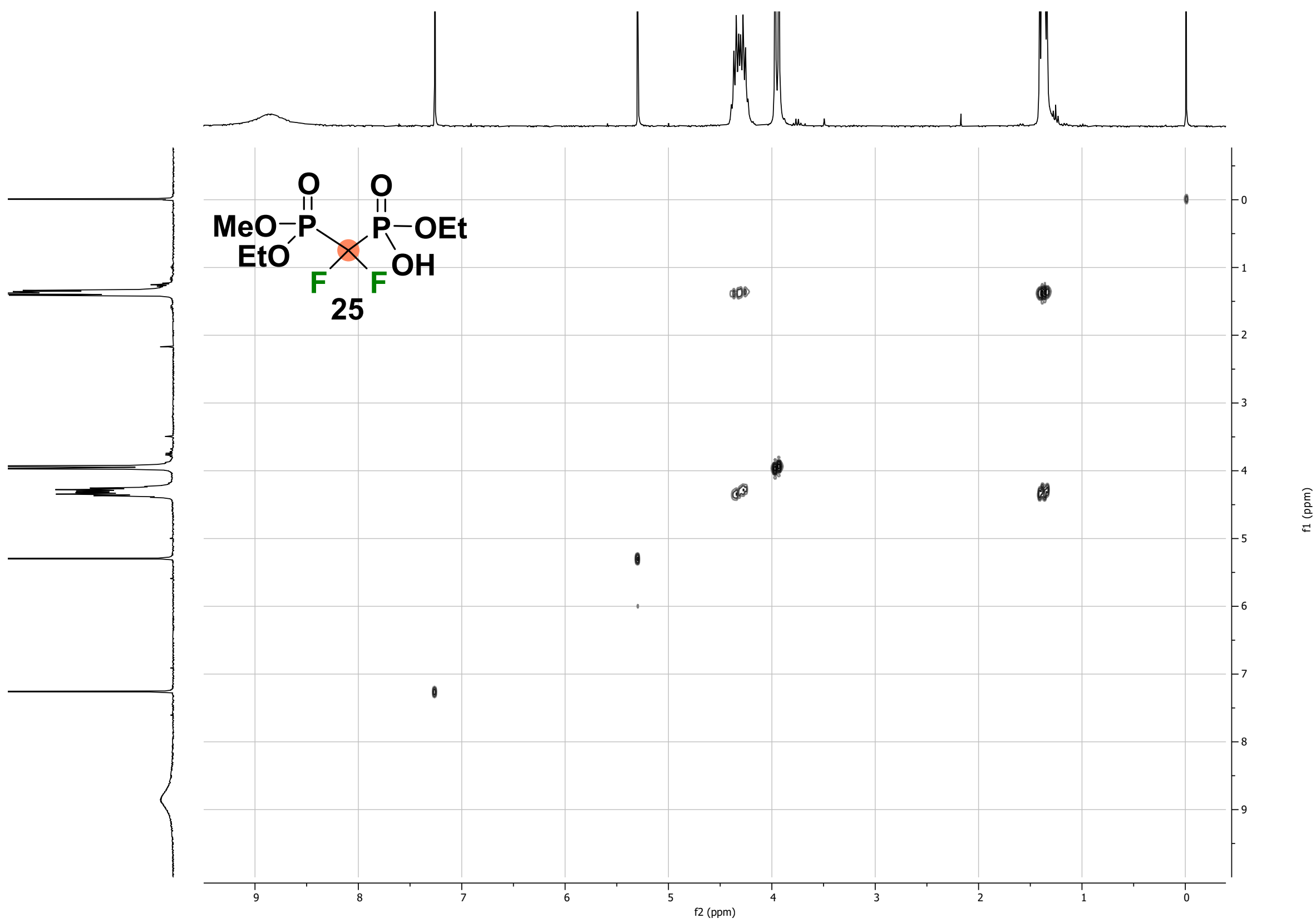

^{1}H-^{1}H COSY (300 MHz, $CDCl_3$) of compound **25**

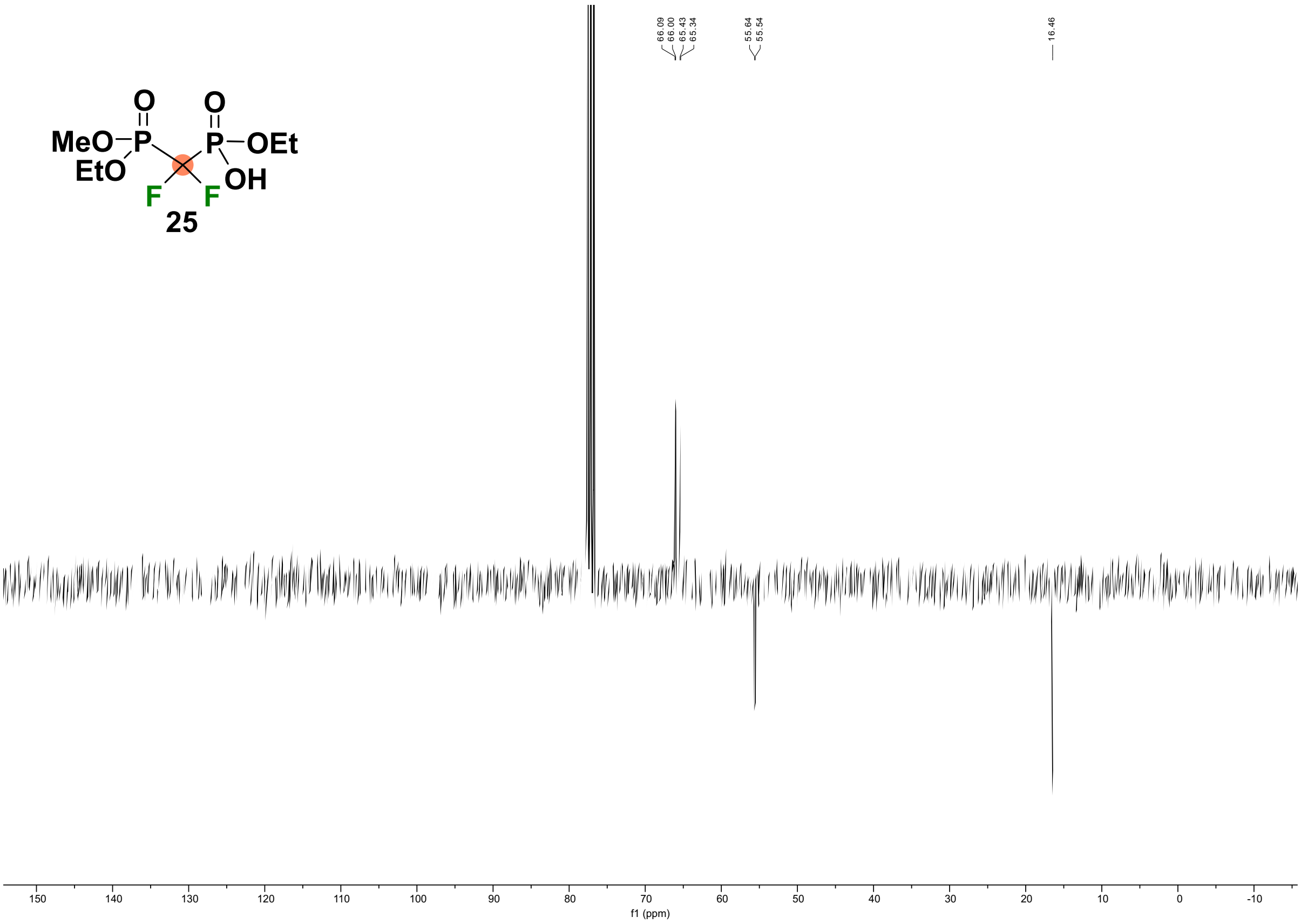

^{13}C NMR (75 MHz, CDCl$_3$) of compound **25**

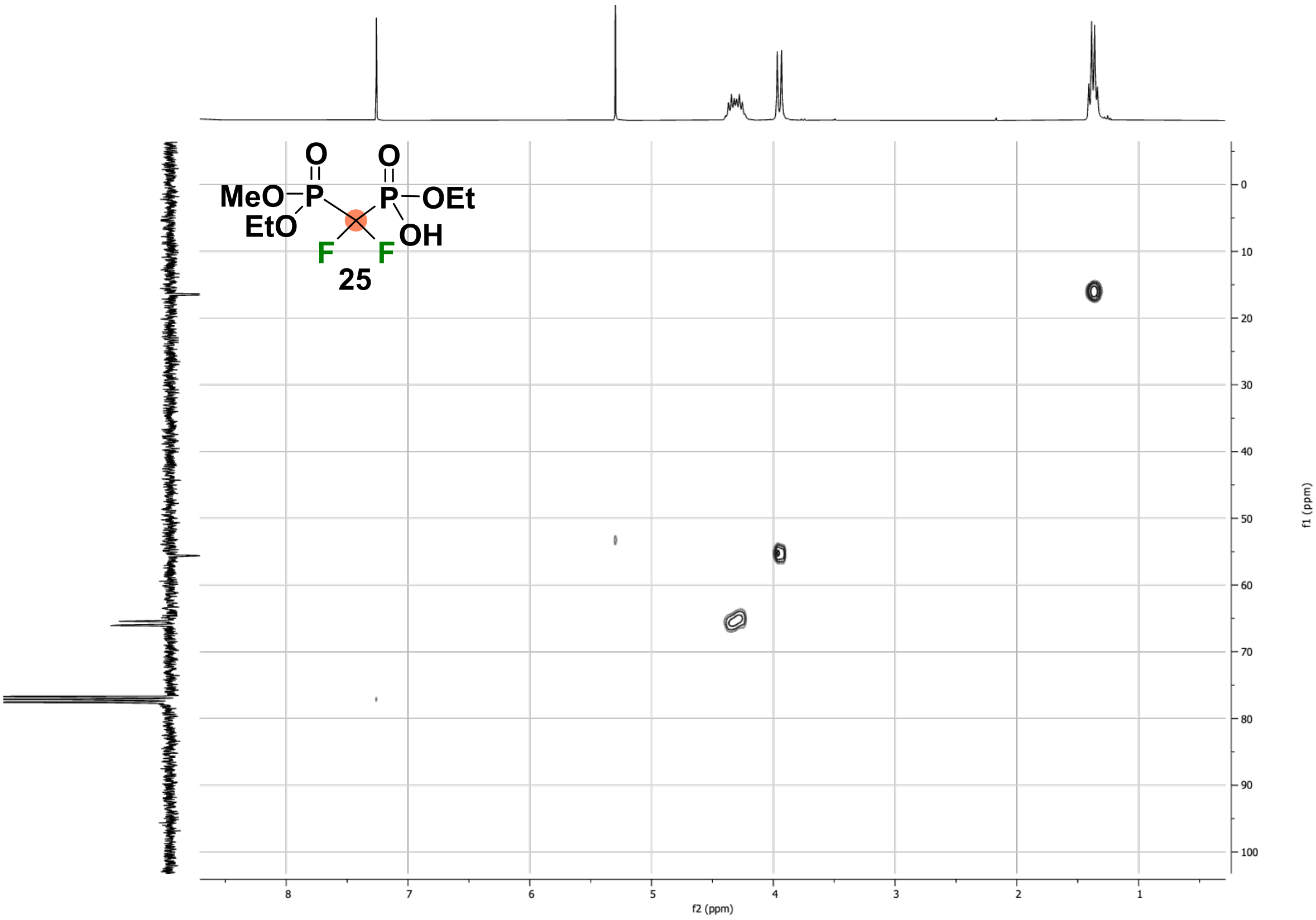

^{1}H-^{13}C HSQC (300 MHz, CDCl$_3$) of compound **25**

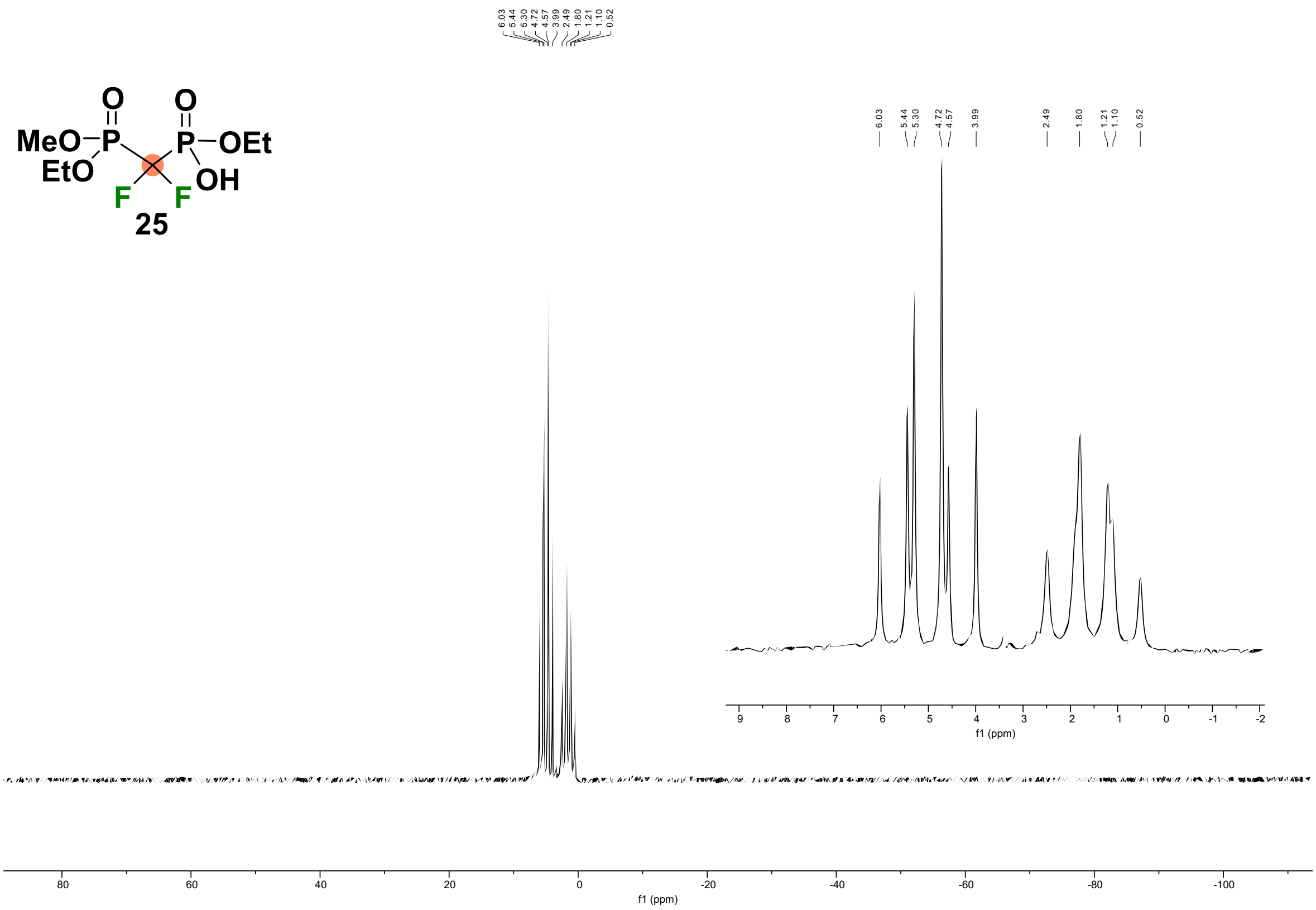

^{31}P NMR (121 MHz, $CDCl_3$) of compound **25**

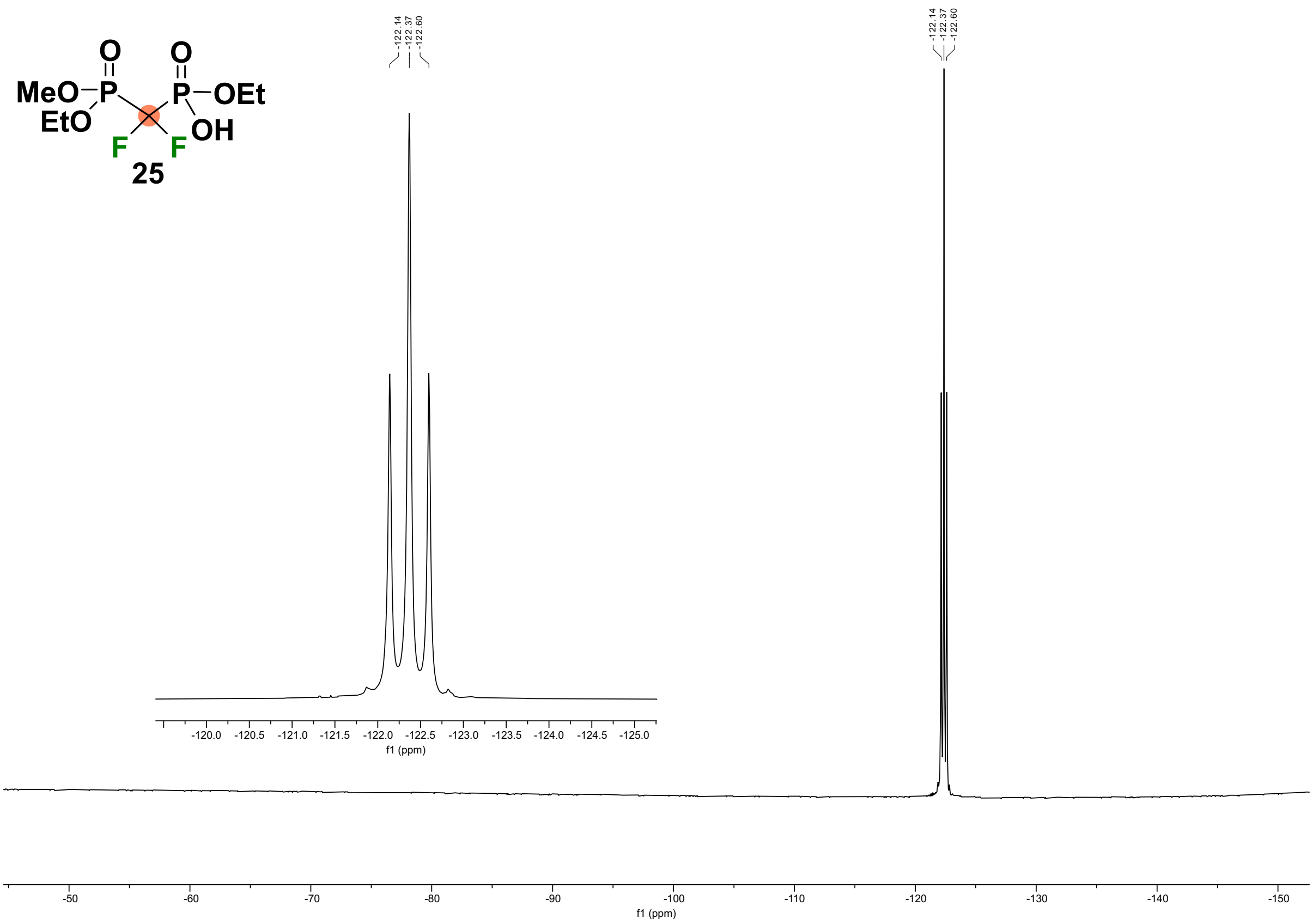

^{19}F NMR (376 MHz, $CDCl_3$) of compound **25**

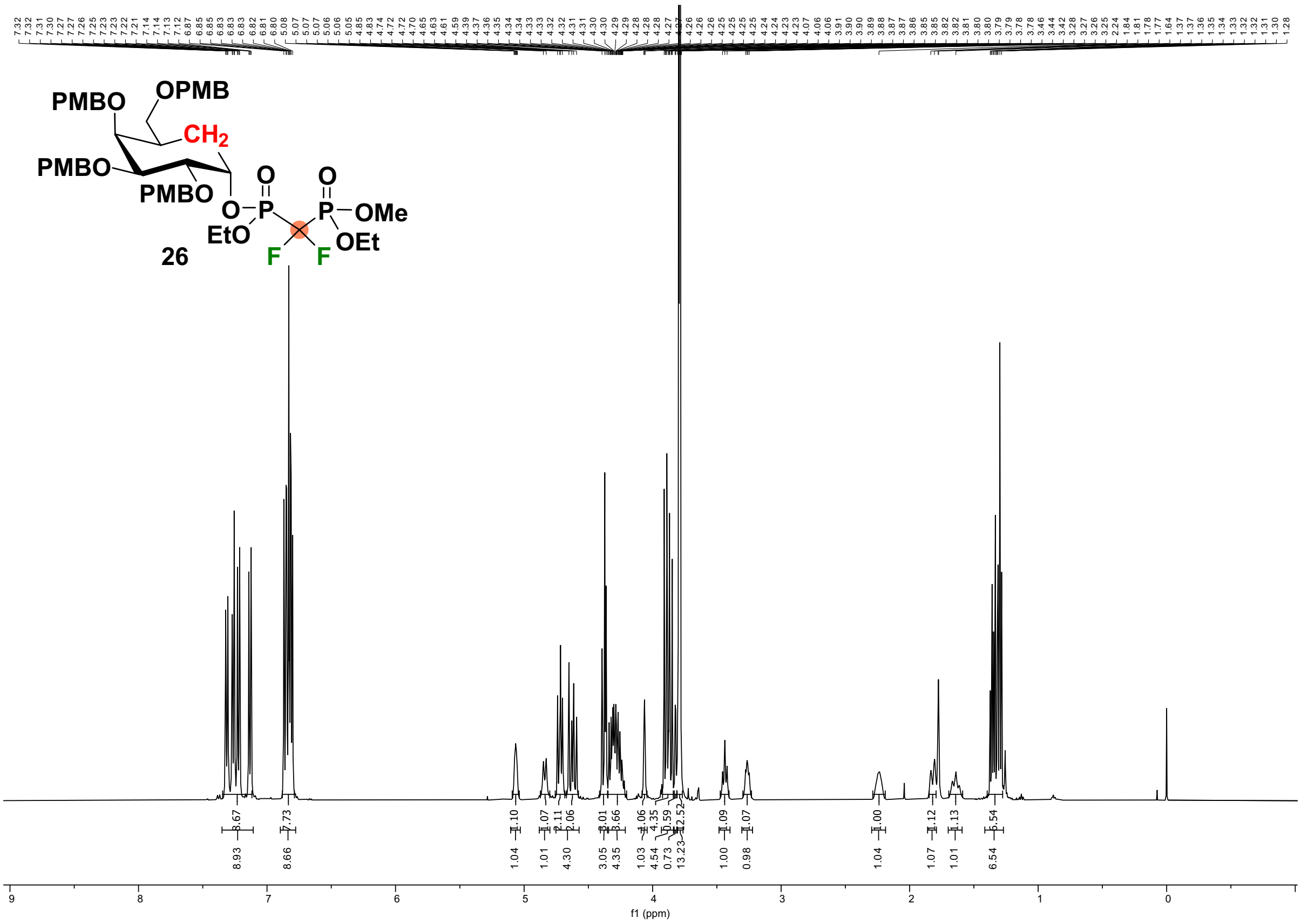

^{1}H NMR (500 MHz, $CDCl_3$) of compound **26**

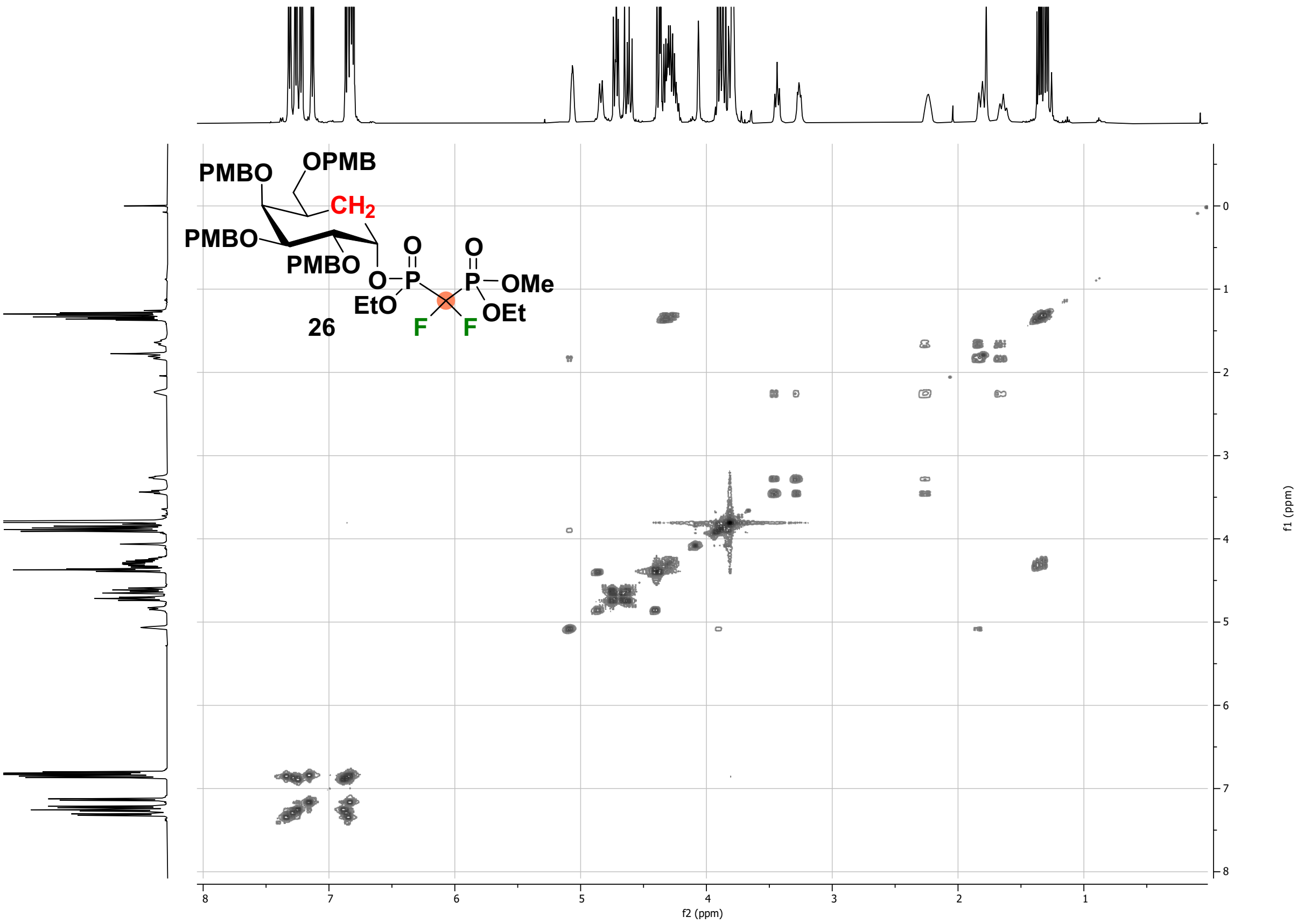

^{1}H-^{1}H COSY (500 MHz, $CDCl_3$) of compound **26**

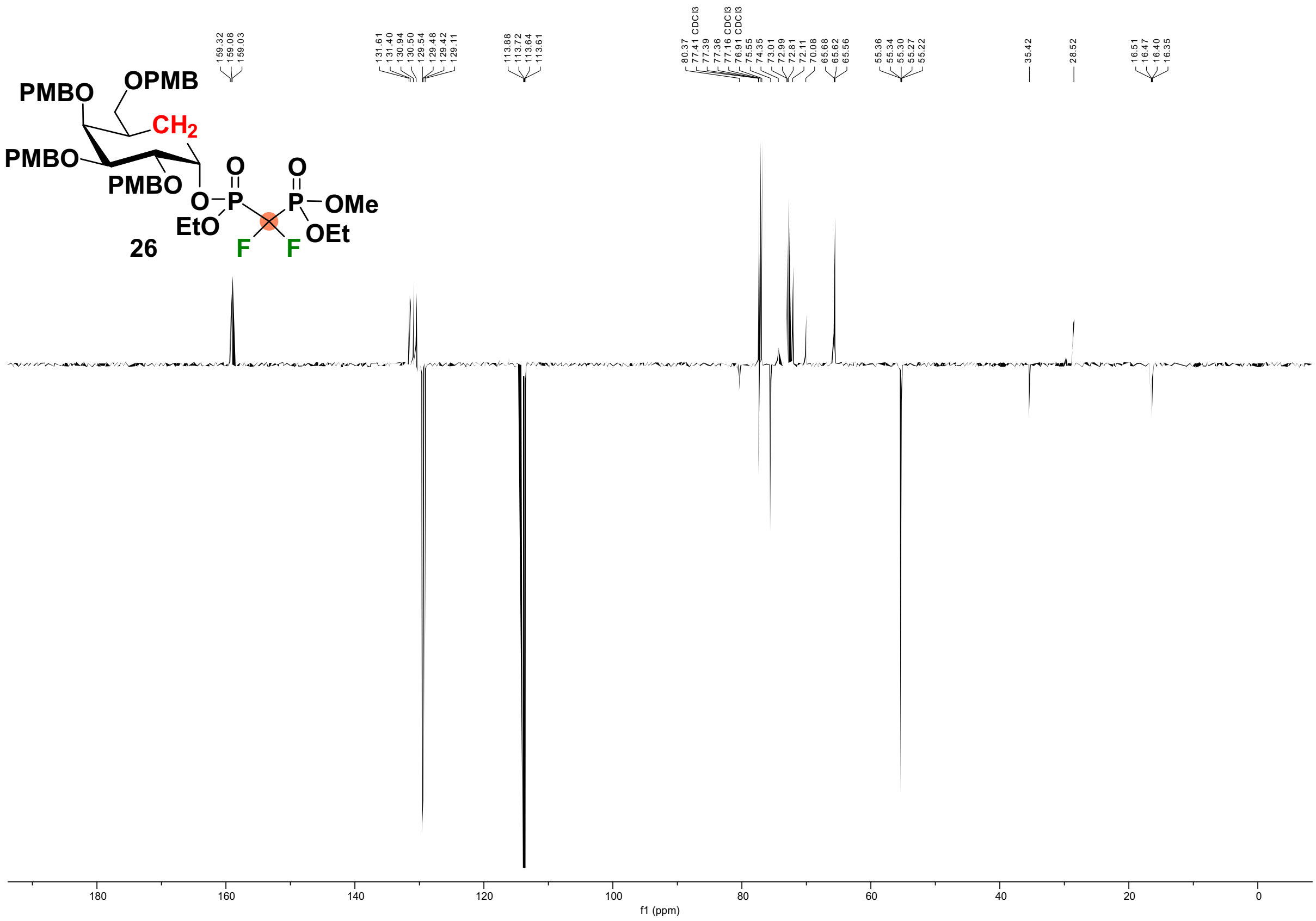

^{13}C NMR (126 MHz, CDCl$_3$) of compound **26**

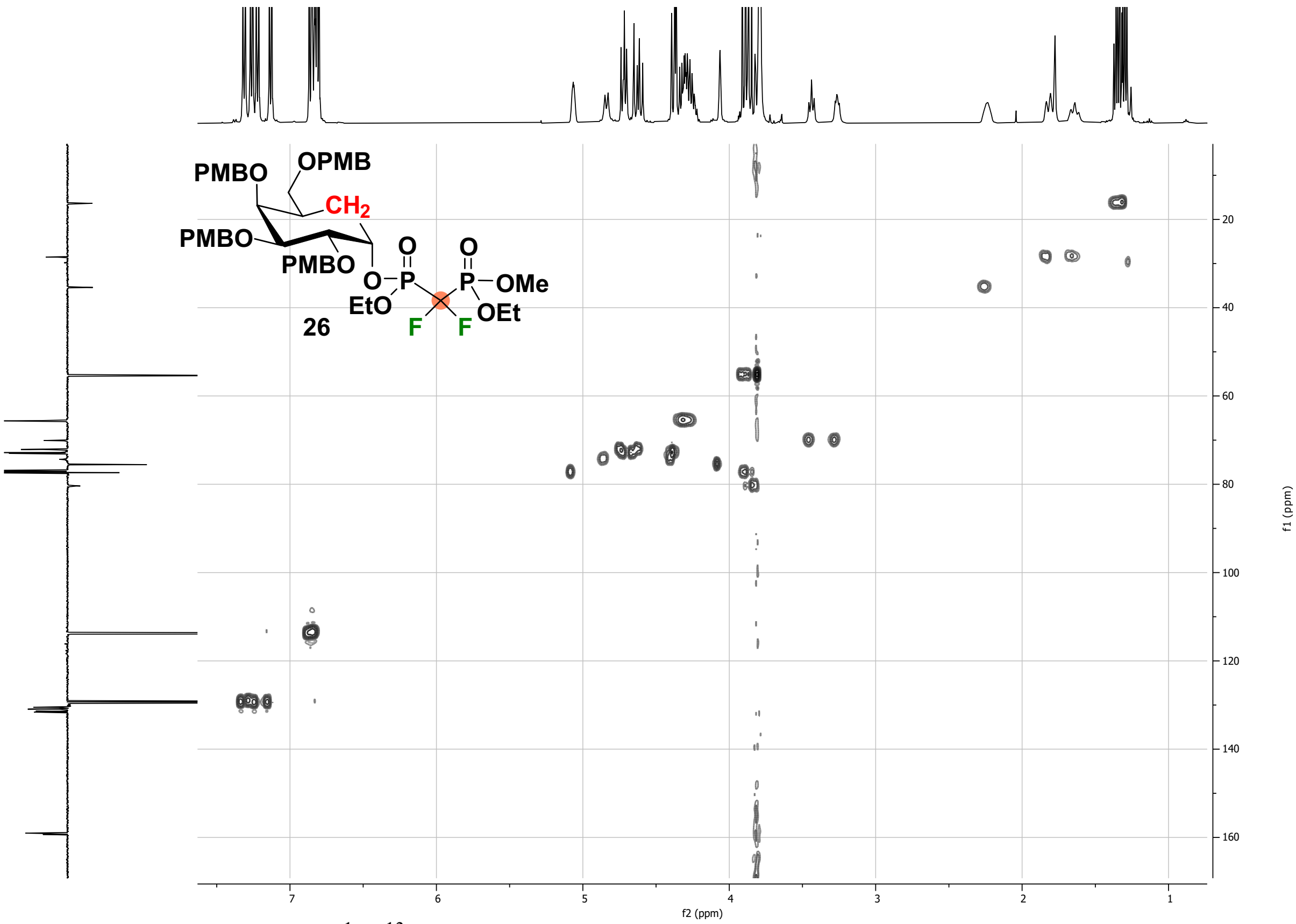

^{1}H-^{13}C HSQC (500 MHz, CDCl$_3$) of compound **26**

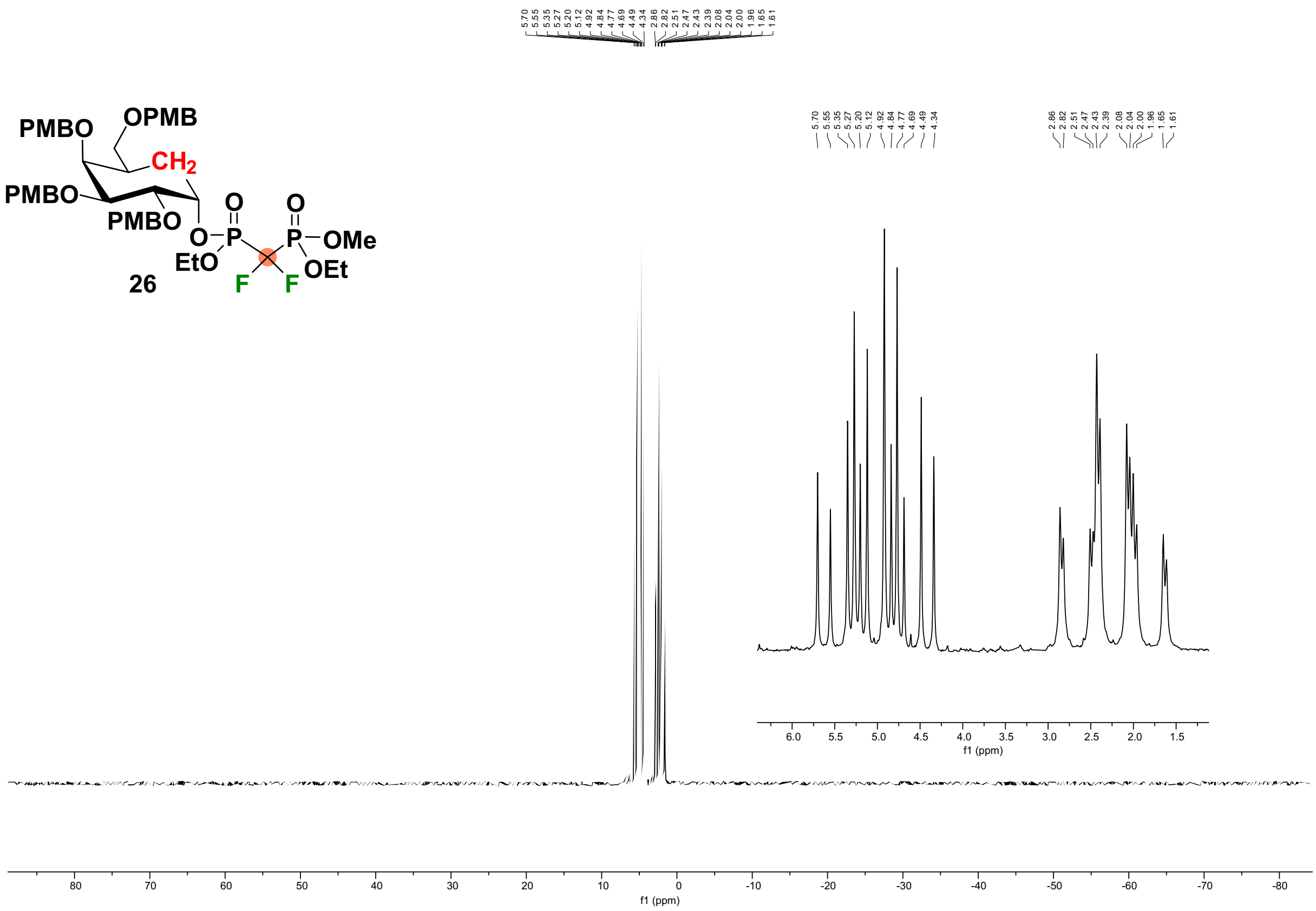

^{31}P NMR (202 MHz, $CDCl_3$) of compound **26**

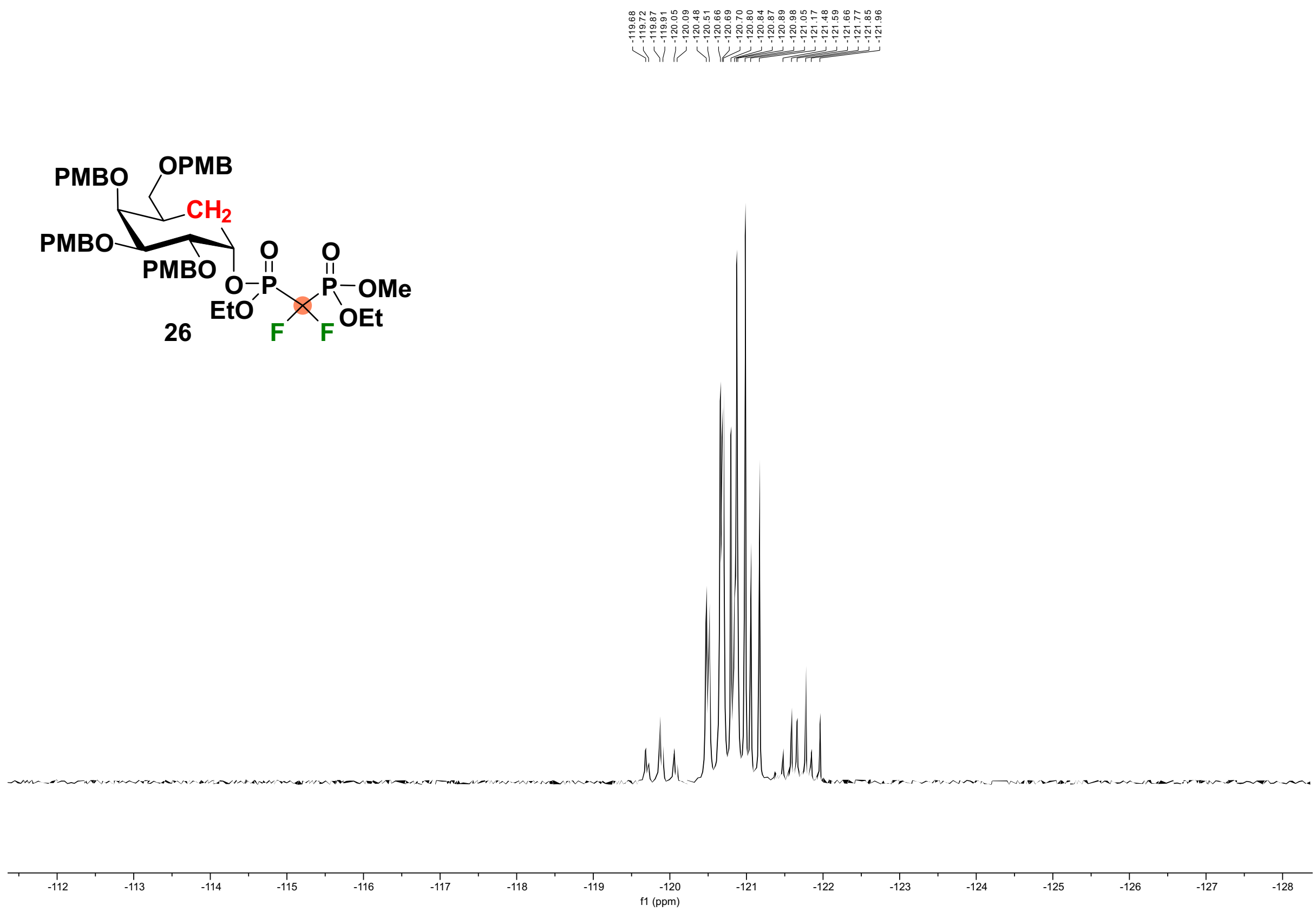

^{19}F NMR (471 MHz, $CDCl_3$) of compound **26**

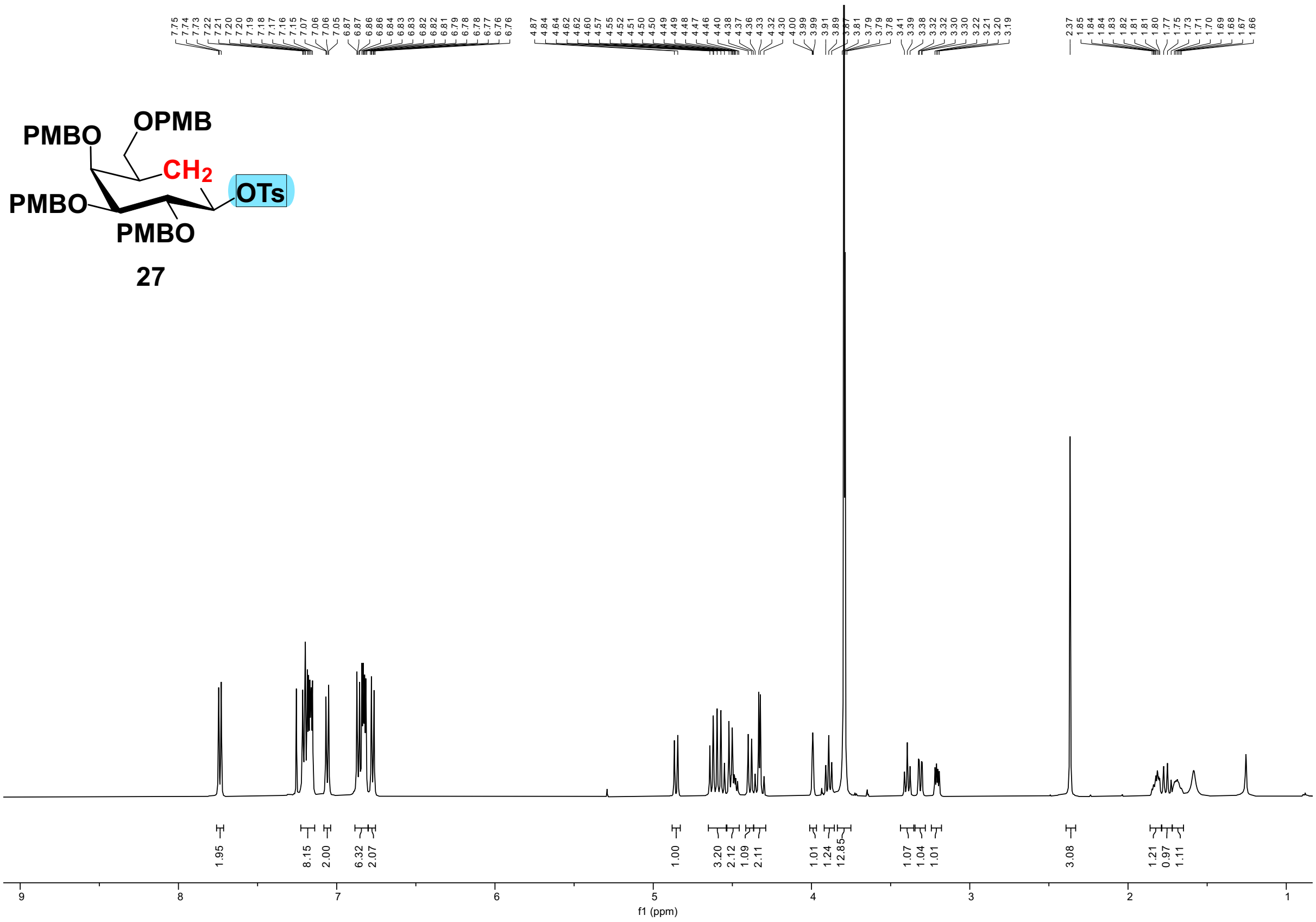

^{1}H NMR (500 MHz, $CDCl_3$) of compound **27**

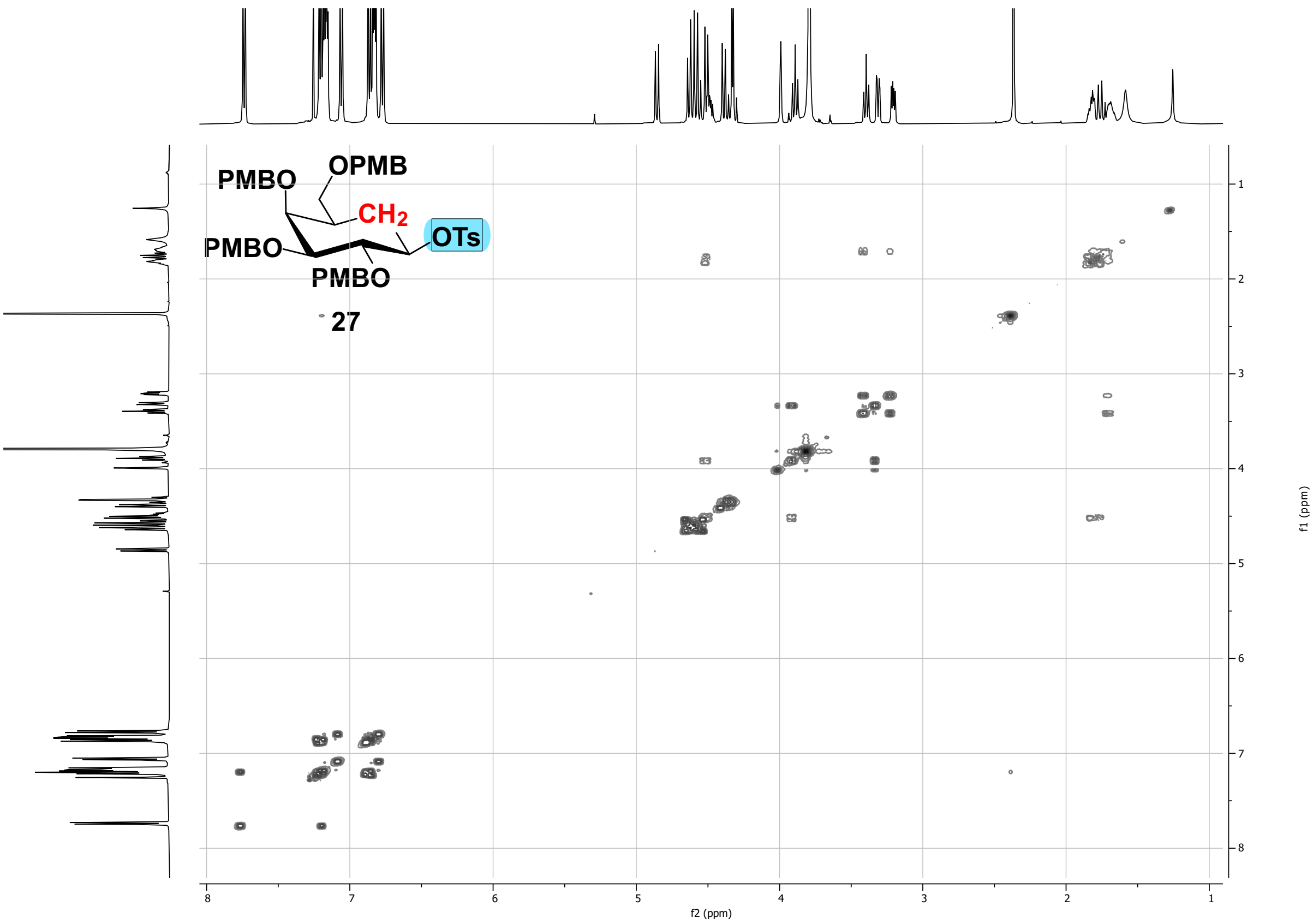

^{1}H-^{1}H COSY (500 MHz, $CDCl_3$) of compound **27**

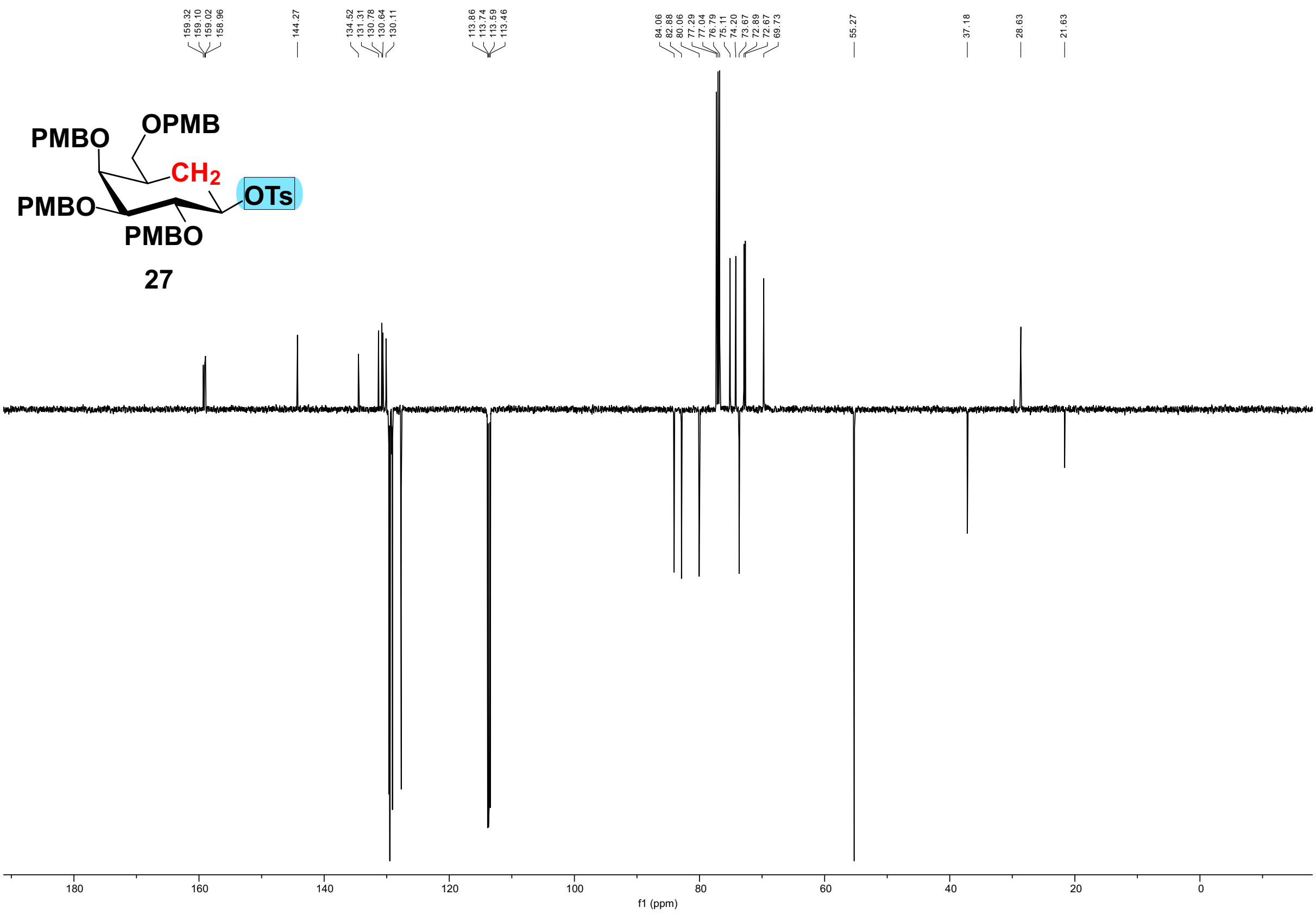

^{13}C NMR (126 MHz, $CDCl_3$) of compound **27**

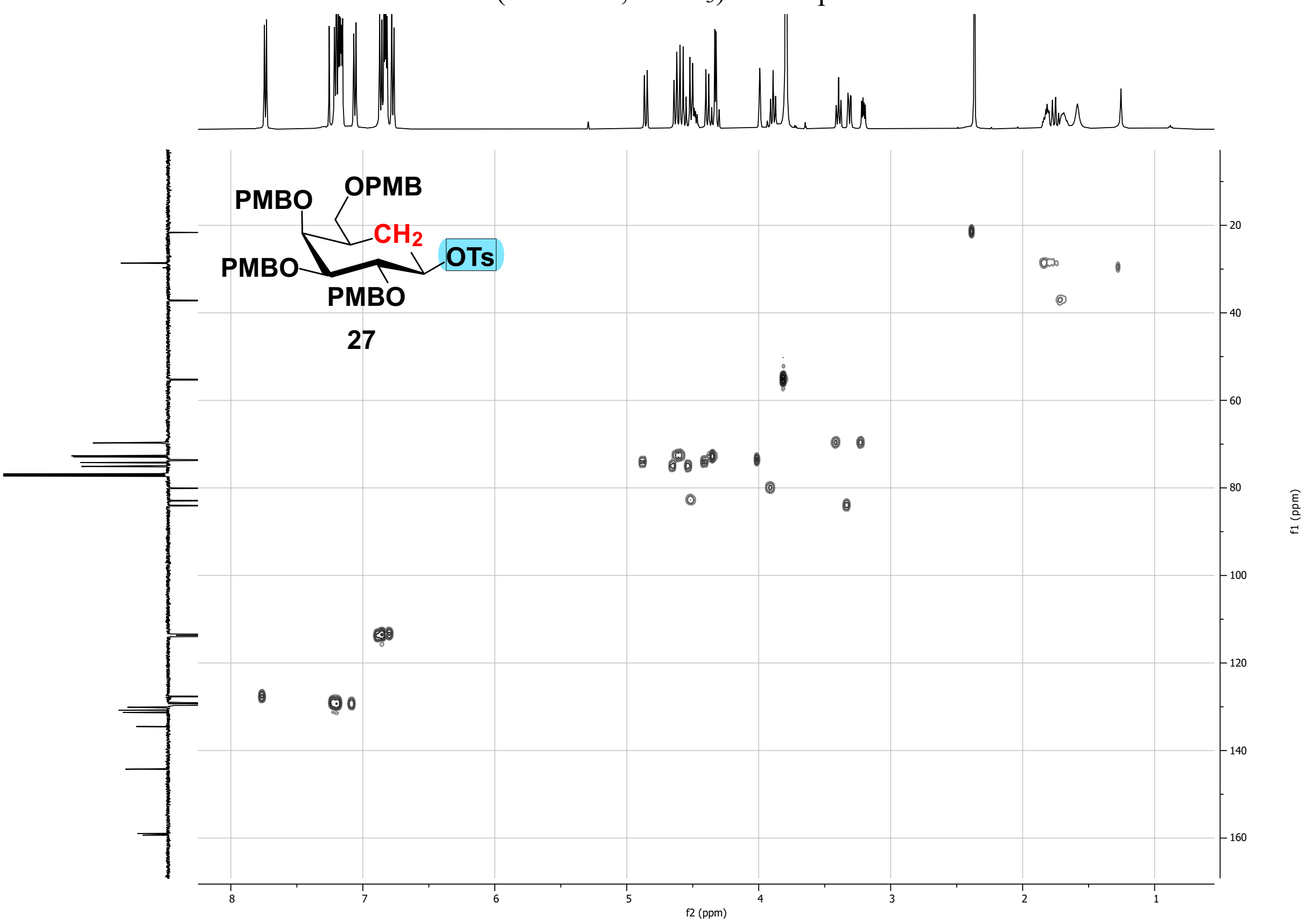

^{1}H-^{13}C HSQC (500 MHz, $CDCl_3$) of compound **27**

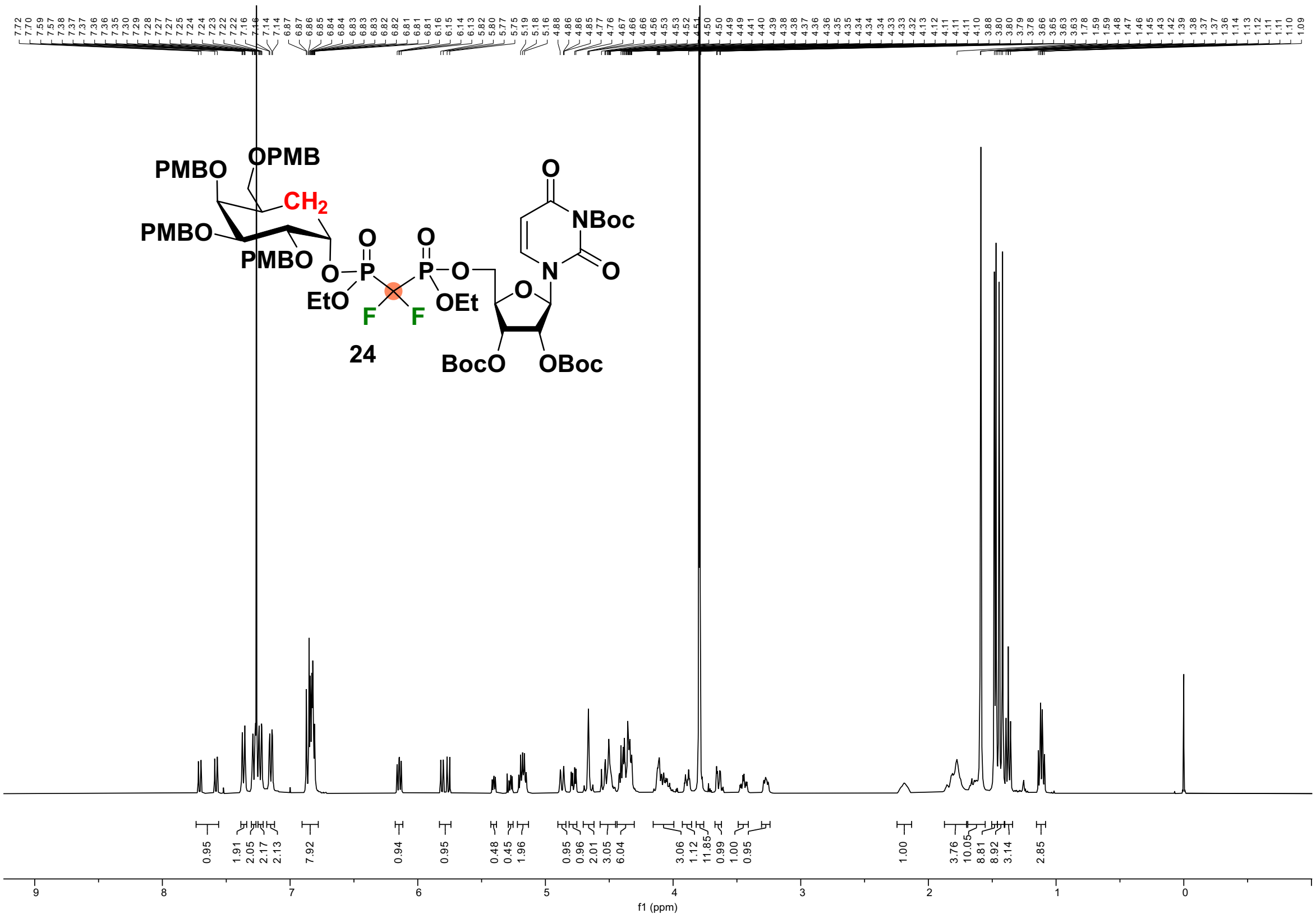

^{1}H NMR (400 MHz, $CDCl_3$) of compound **24**

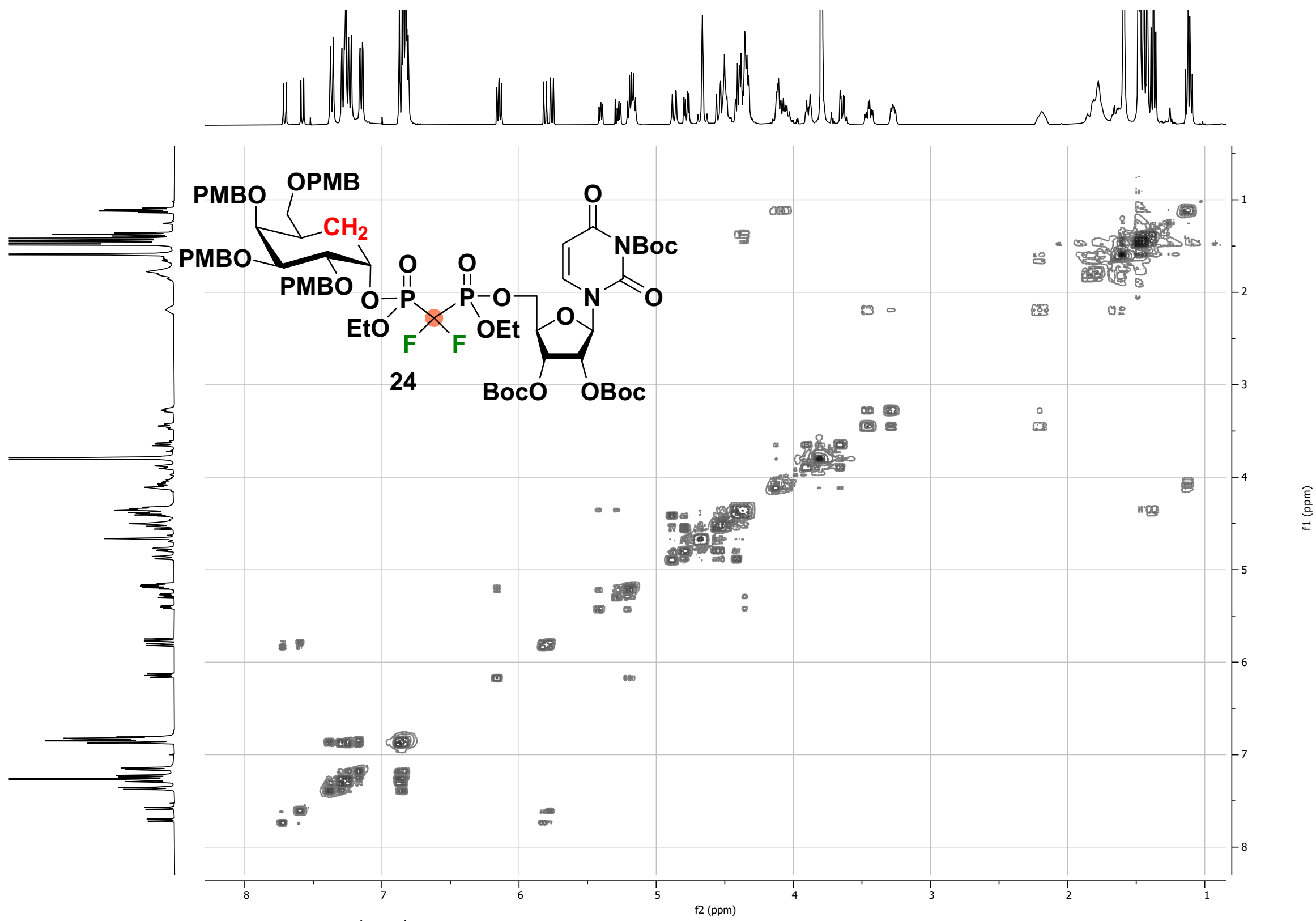

^{1}H-^{1}H COSY (400 MHz, $CDCl_3$) of compound **24**

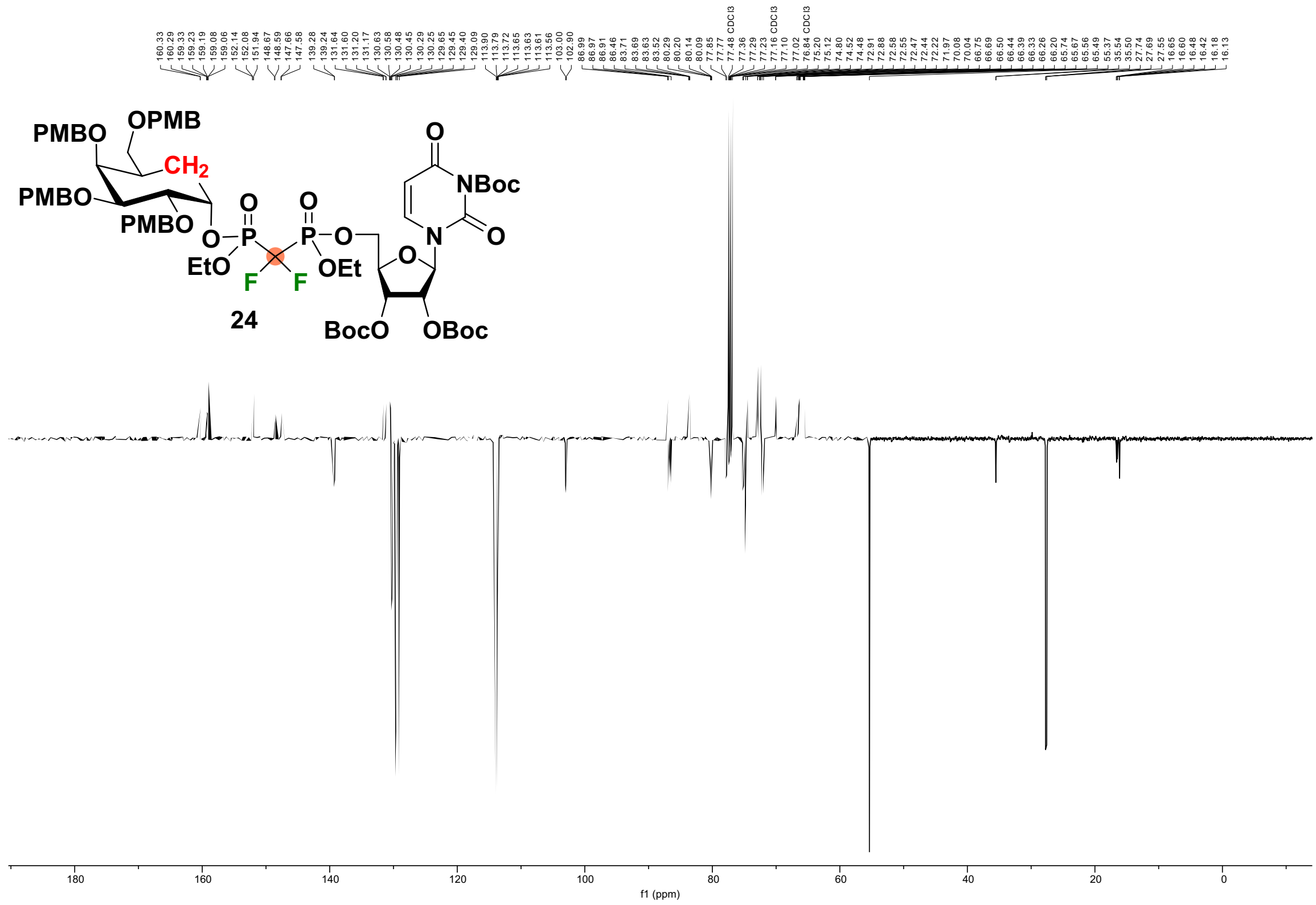

^{13}C NMR (101 MHz, $CDCl_3$) of compound **24**

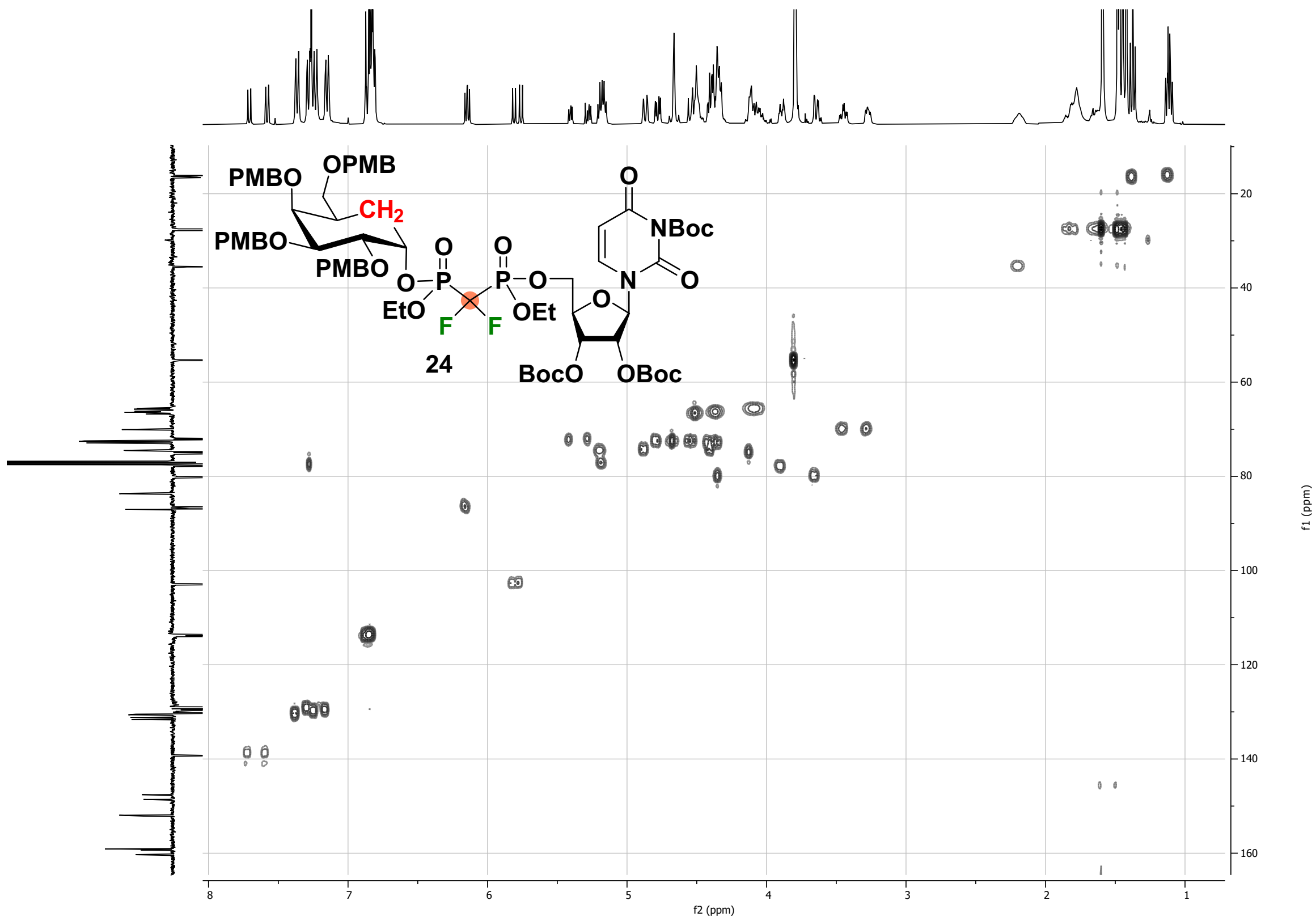

^{1}H-^{13}C HSQC (400 MHz, $CDCl_3$) of compound **24**

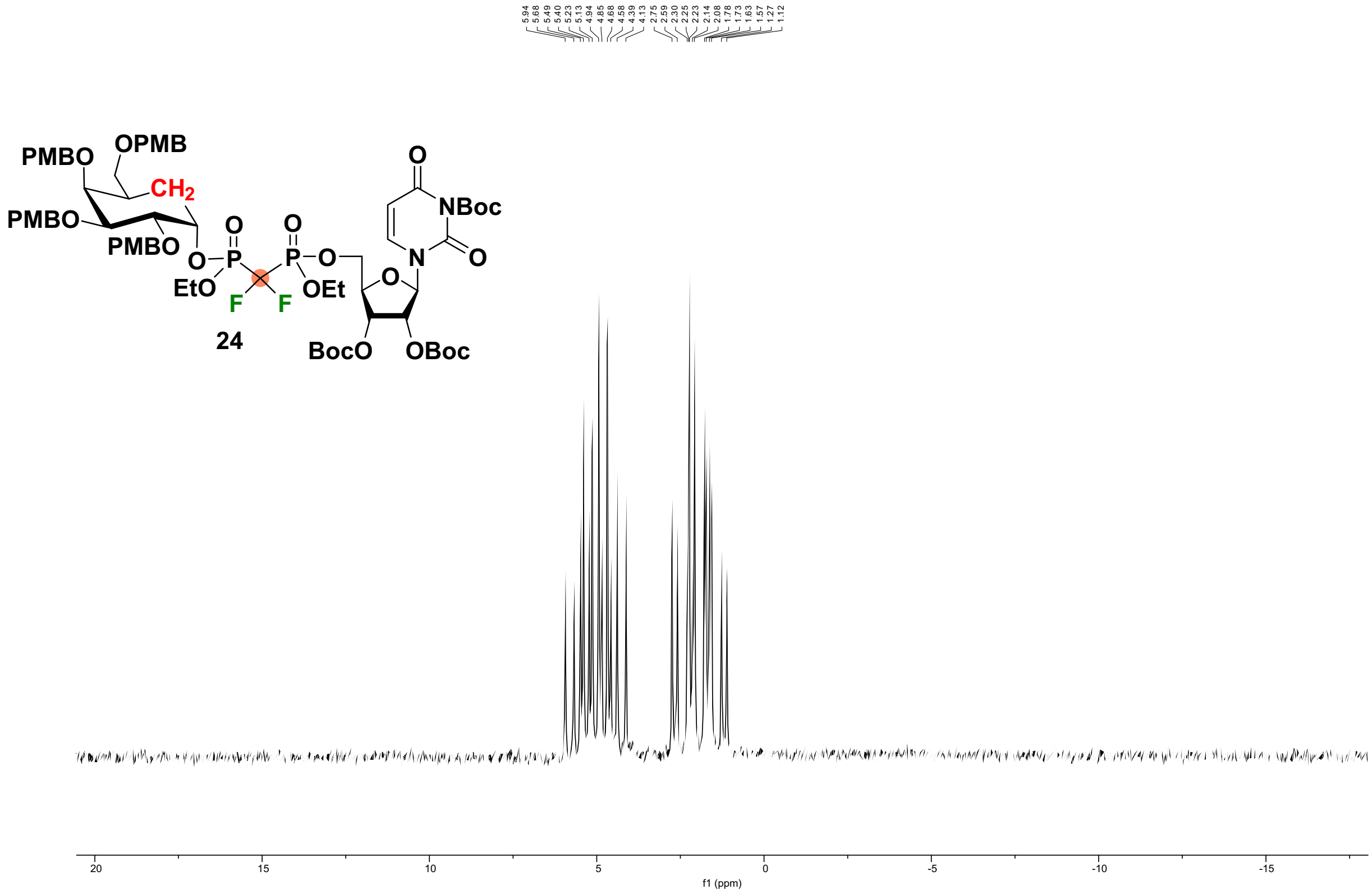

^{31}P NMR (162 MHz, $CDCl_3$) of compound **24**

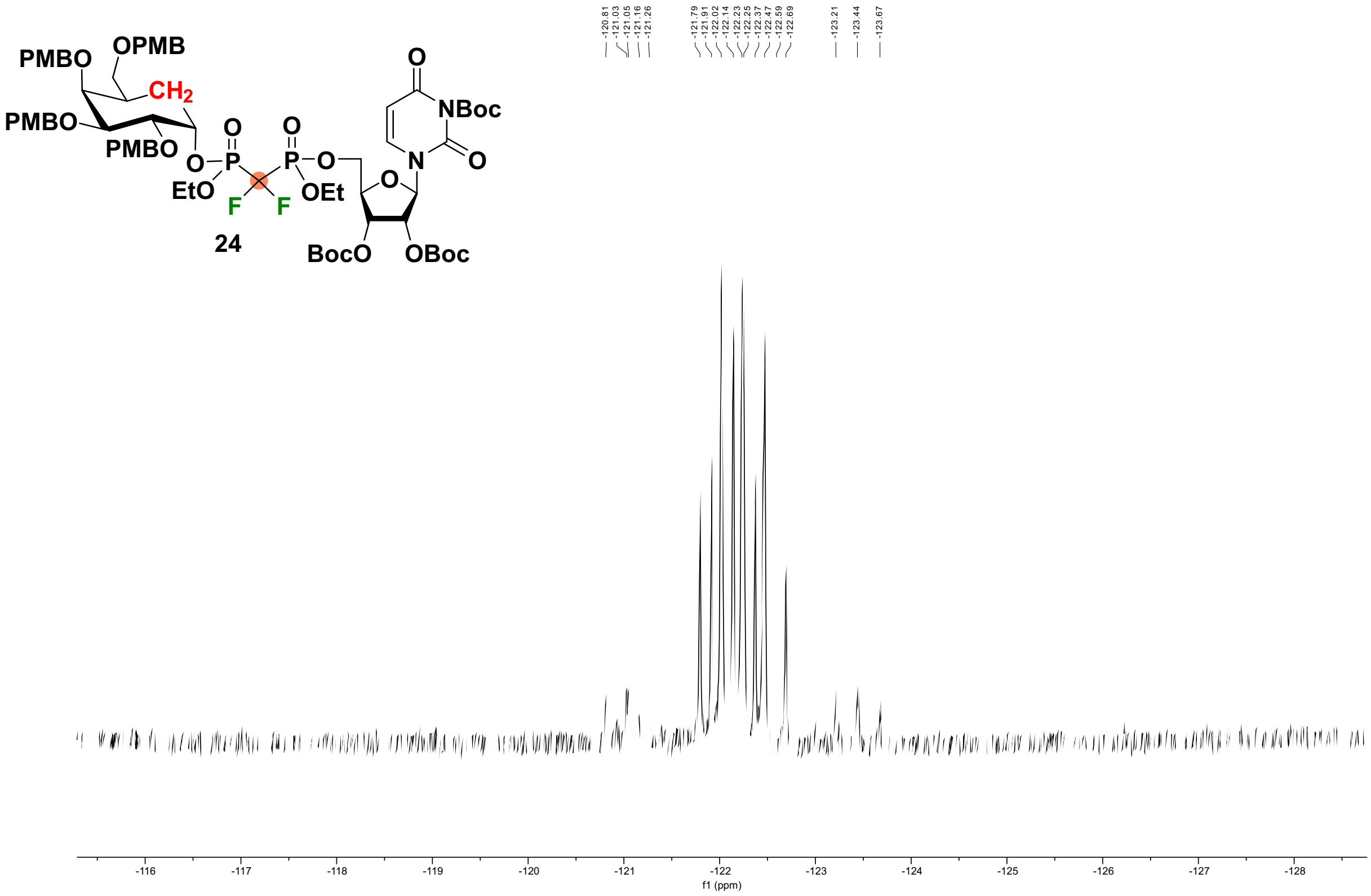

^{19}F NMR (376 MHz, $CDCl_3$) of compound **24**

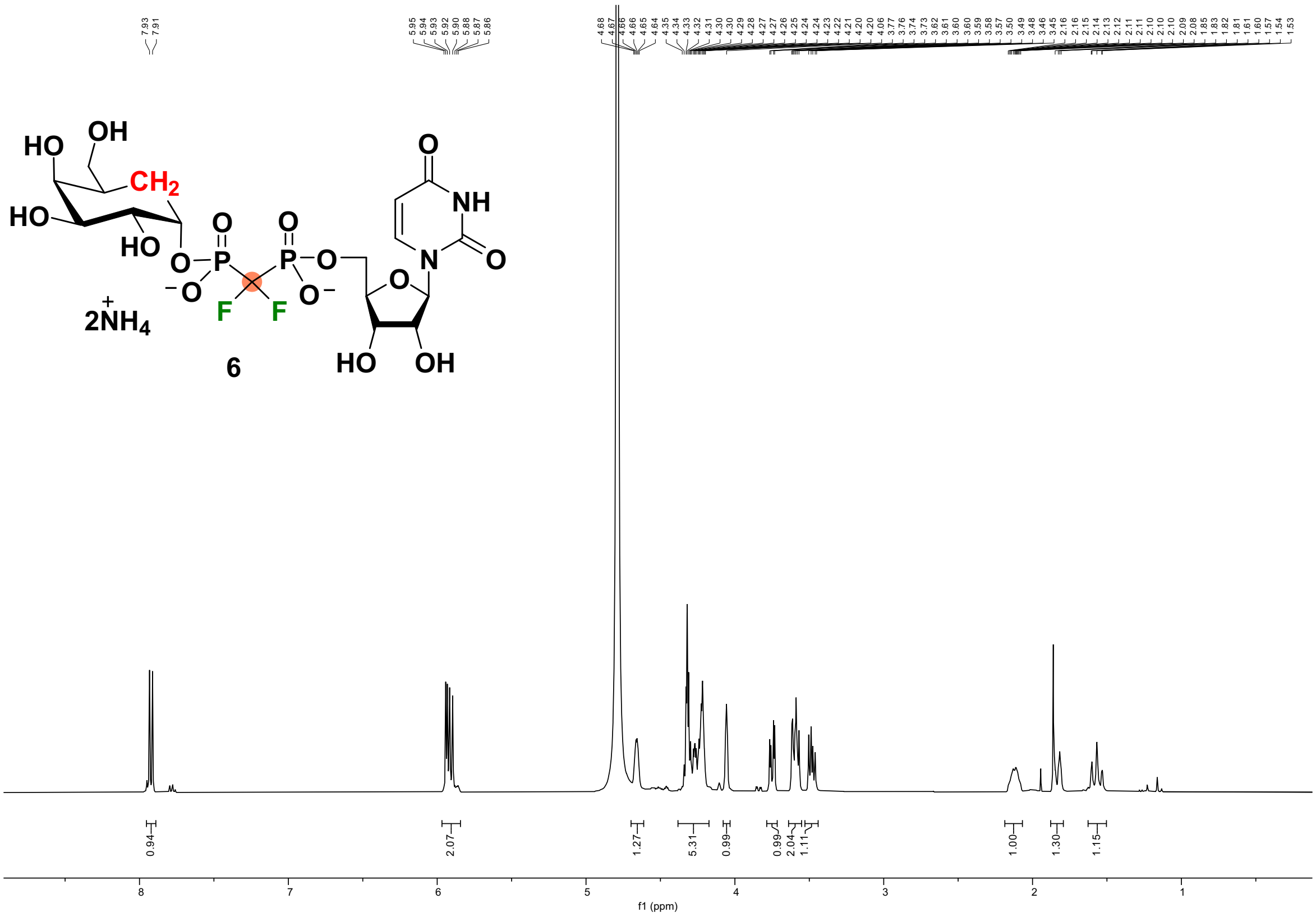

^{1}H NMR (400 MHz, $CDCl_3$) of compound **6**

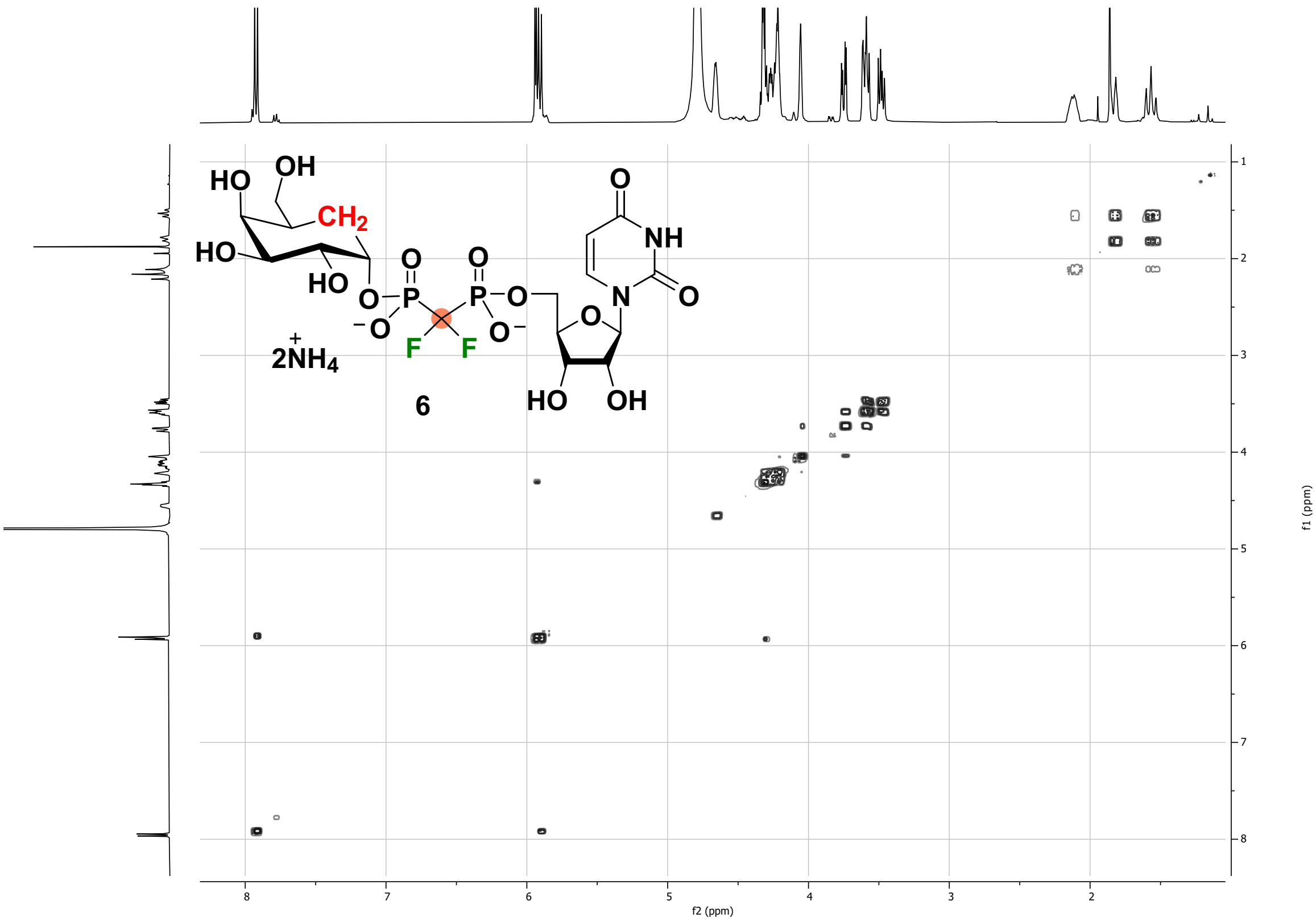

^{1}H-^{1}H COSY (400 MHz, $CDCl_3$) of compound **6**

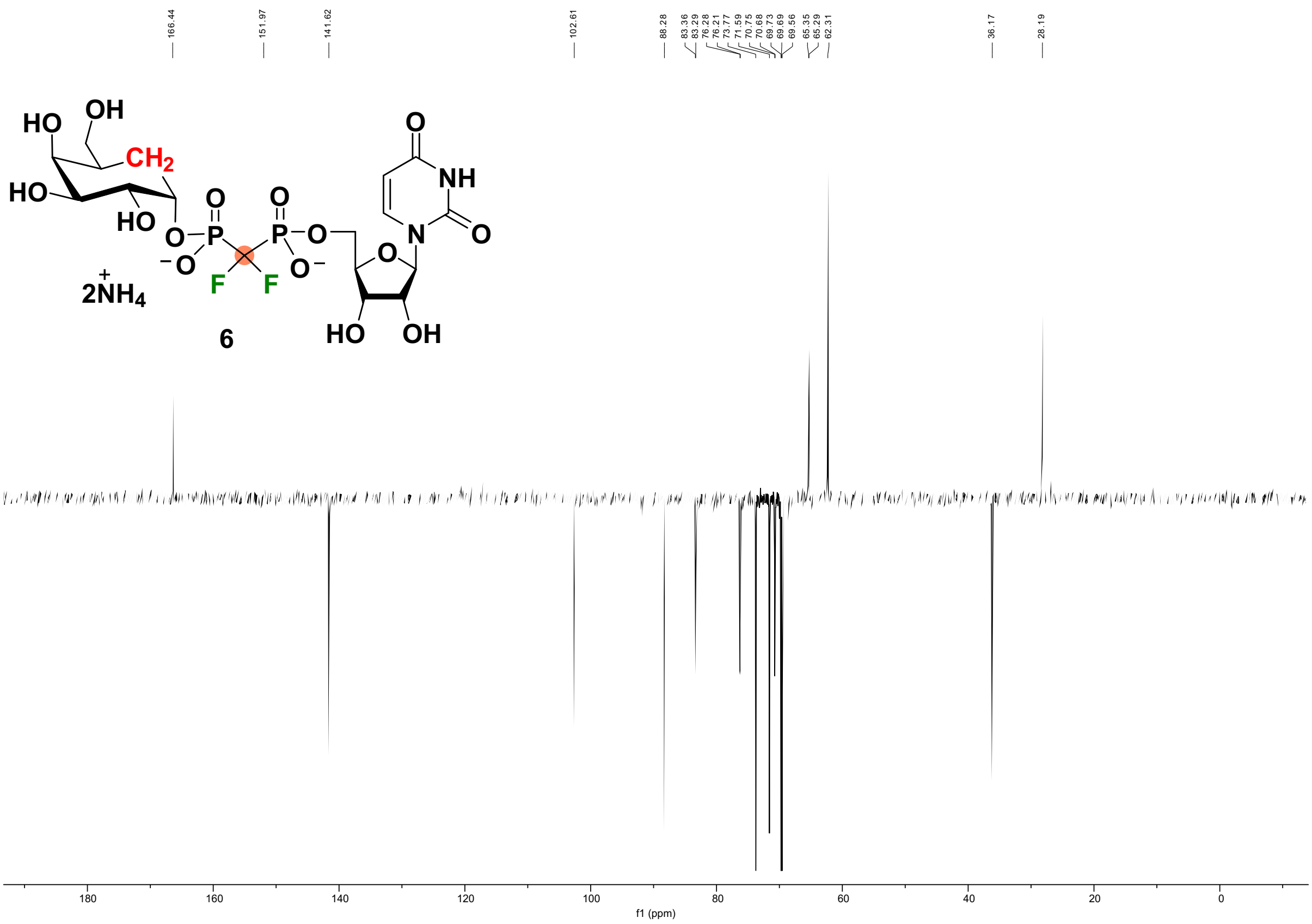

^{13}C NMR (101 MHz, $CDCl_3$) of compound **6**

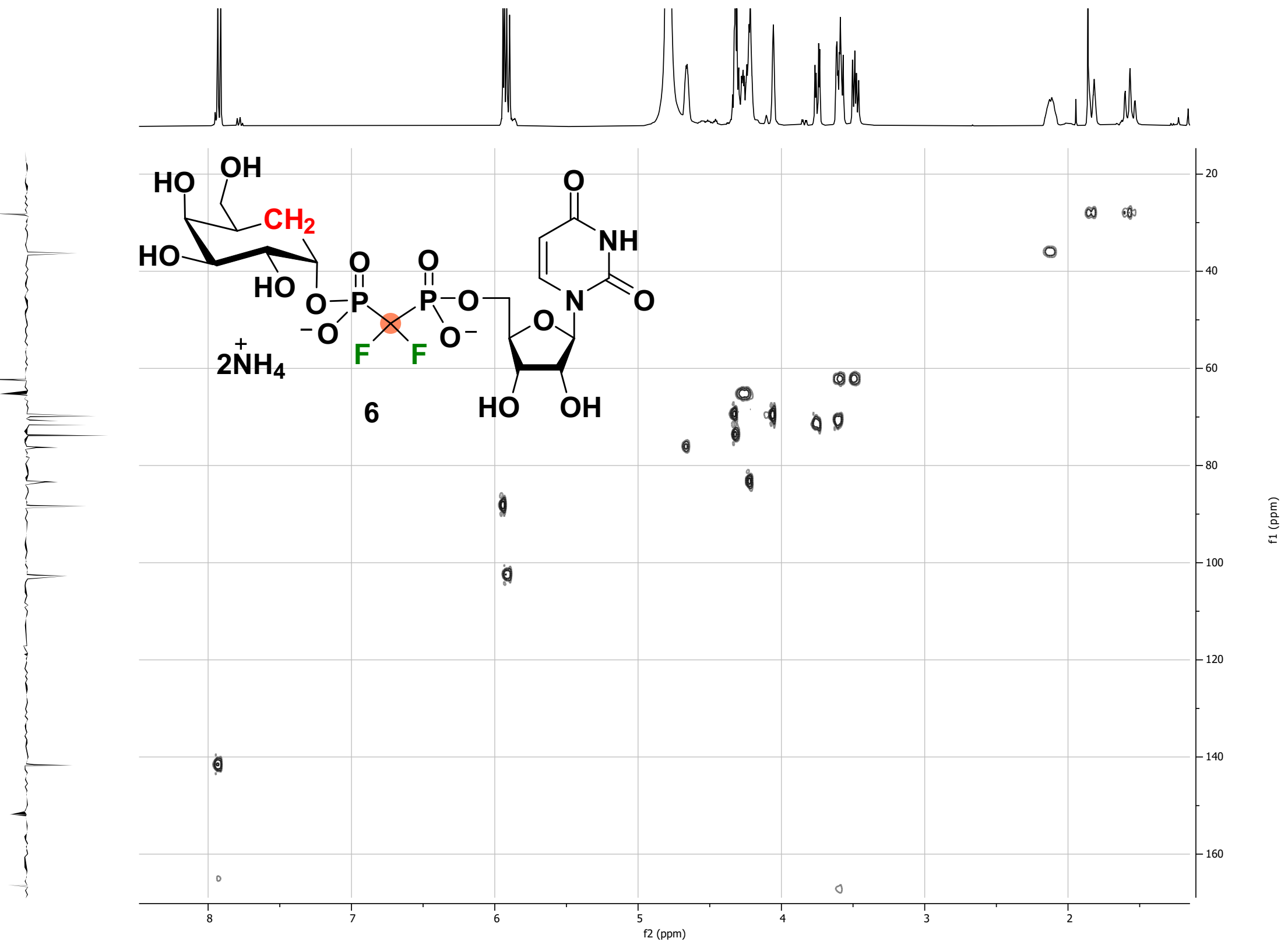

^{1}H-^{13}C HSQC (400 MHz, $CDCl_3$) of compound **6**

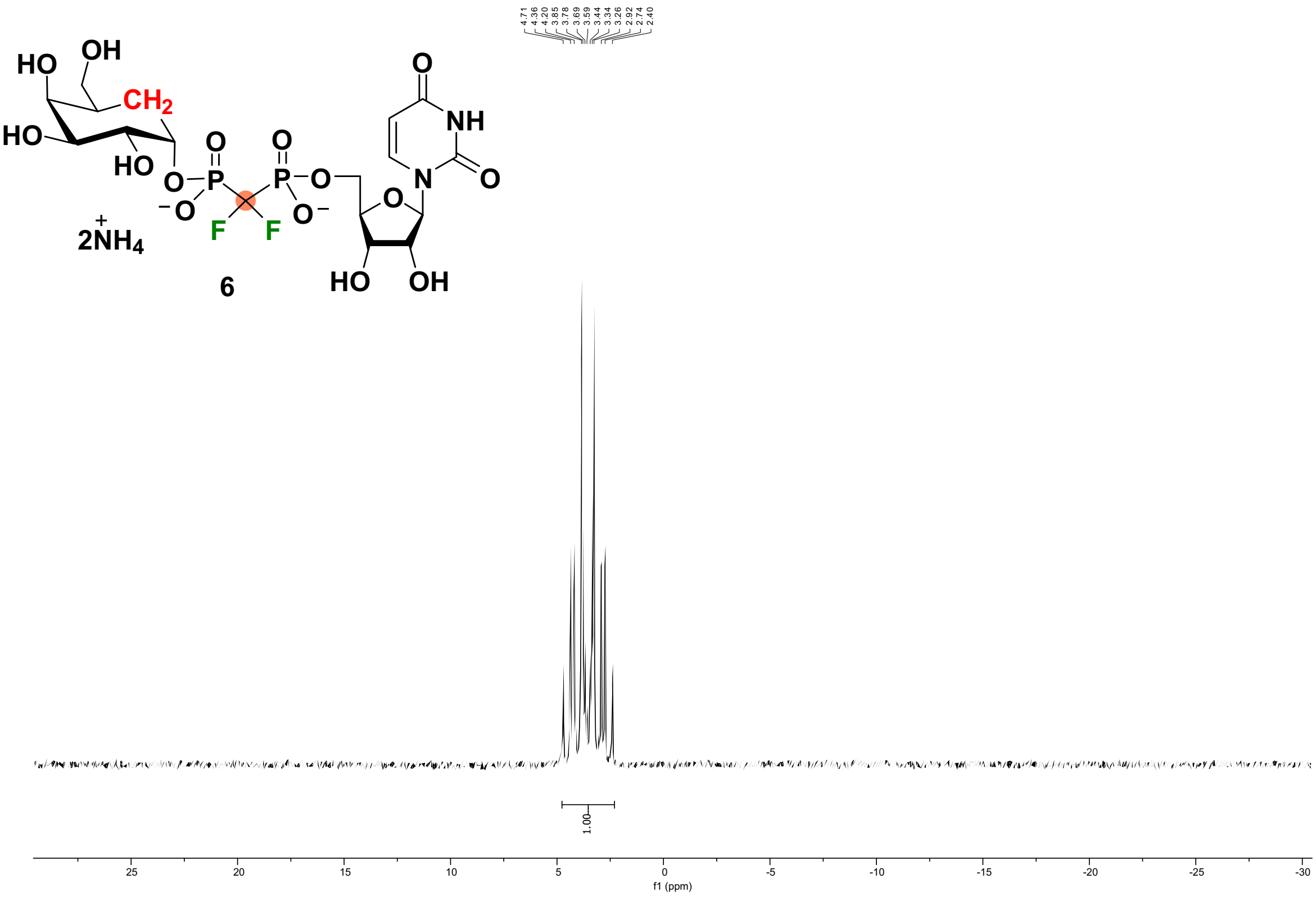

^{31}P NMR (162 MHz, $CDCl_3$) of compound **6**

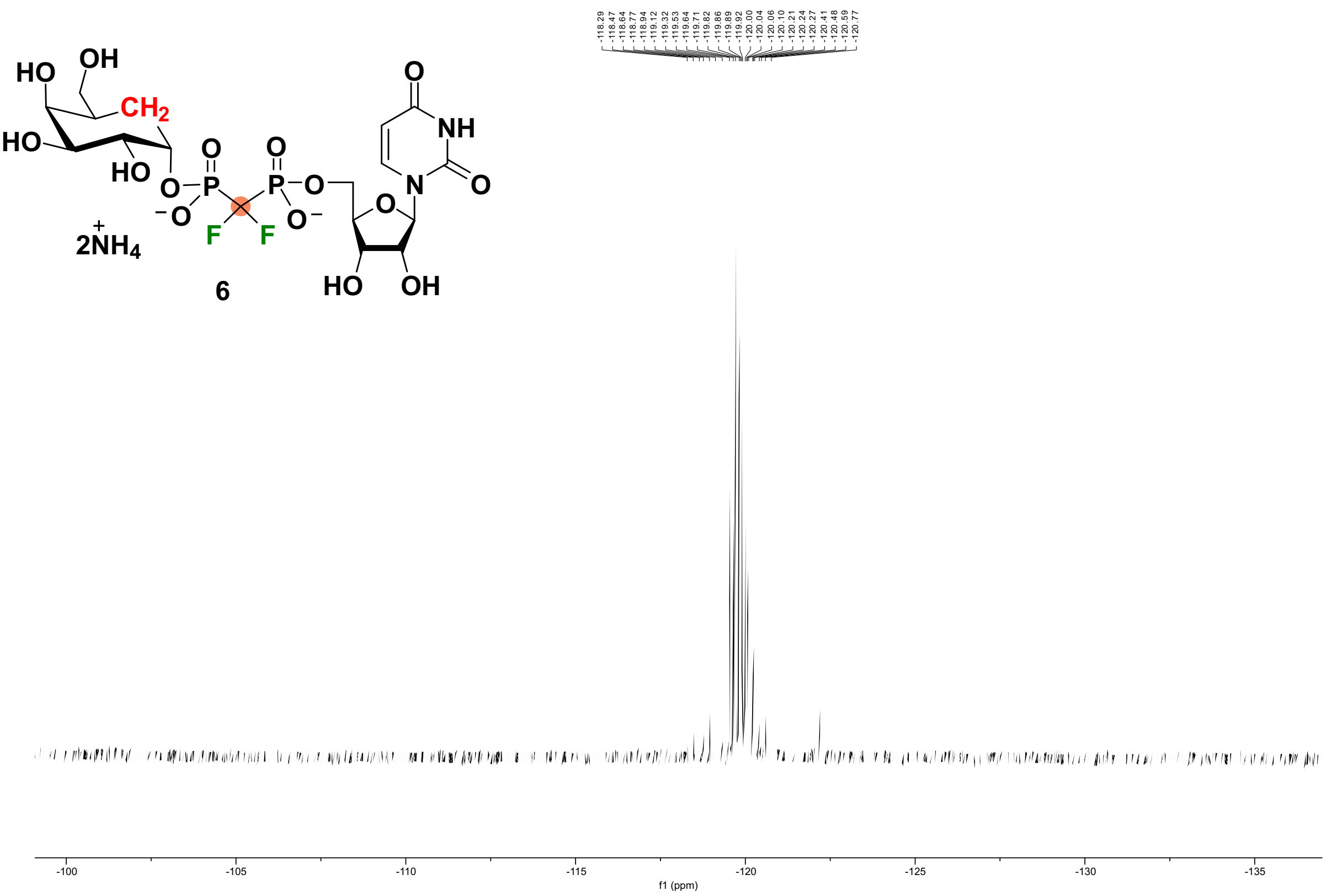

^{19}F NMR (376 MHz, $CDCl_3$) of compound **6**
